# Cytotoxic Drimane-Type Sesquiterpenes from Co-Culture of the Marine-Derived Fungi *Aspergillus carneus* KMM 4638 and *Beauveria felina* (=*Isaria felina*) KMM 4639

**DOI:** 10.3390/md20090584

**Published:** 2022-09-19

**Authors:** Olesya I. Zhuravleva, Elena B. Belousova, Galina K. Oleinikova, Alexandr S. Antonov, Yuliya V. Khudyakova, Anton B. Rasin, Roman S. Popov, Ekaterina S. Menchinskaya, Phan Thi Hoai Trinh, Anton N. Yurchenko, Ekaterina A. Yurchenko

**Affiliations:** 1 G.B. Elyakov Pacific Institute of Bioorganic Chemistry, Far Eastern Branch of the Russian Academy of Sciences, Prospect 100-letiya Vladivostoka, 159, Vladivostok 690022, Russia; 2 Institute of High Technologies and Advanced Materials, Far Eastern Federal University, 10 Ajax Bay, Russky Island, Vladivostok 690922, Russia; 3 Department of Marine Biotechnology, Nhatrang Institute of Technology Research and Application, Vietnam Academy of Science and Technology, Nha Trang 650000, Vietnam

**Keywords:** marine-derived fungi, *Aspergillus carneus*, *Beauveria felina* (*Isaria felina*), coculture, secondary metabolites, drimane sesquiterpenes, cytotoxicity

## Abstract

Chemical investigation of a coculture of the marine-derived fungi *Beauveria felina* KMM 4639 and *Aspergillus carneus* KMM 4638 led to the identification of three new drimane-type sesquiterpenes, asperflavinoids B, D and E (**2**, **4**, **5**), and nine previously reported related compounds. The structures of these compounds were established using spectroscopic methods and by comparison with known analogues. We also investigated the cytotoxic activity of the isolated compounds against several cancer and normal cell lines. Asperflavinoid C (**3**) and ustusolate E (**9**) exerted a significant effect on human breast cancer MCF-7 cell viability, with IC_50_ values of 10 µM, and induced in caspase-dependent apoptosis and arrest of the MCF-7 cell cycle in the G2/M phase in these cells.

## 1. Introduction

Recent studies show that one of the factors causing the induction of the biosynthesis of new bioactive metabolites from marine fungi is coculture with other microorganisms, including fungi. The essence of this approach is to simulate, to a certain extent, the natural microbial complex, whereby microorganisms produce bioactive secondary metabolites necessary for survival in a competitive environment. Interspecies microbial control can have a significant impact on secondary metabolites produced for habitat protection or as chemical signals [1]. Mixed cultivation can activate silent genes that are inactive in monocultures [2]. Joint cultivation can result in an increase in the yields of previously isolated bioactive compounds, obtaining their derivatives, as well as the synthesis of novel metabolites of various chemical classes. For example, the new meroterpenoid derivative chermebilaene A, with potent inhibitory activities against *Ceratobasidium cornigerum* (MIC 0.5 µg/mL), and *Edwardsiella tarda* (MIC 0.25 µg/mL) were characterized from a coculture of the marine-derived fungal isolates of *Penicillium bilaiae* MA-267 and *Penicillium chermesinum* EN-480 [3]. The new alkaloid aluminumneohydroxyaspergillin was isolated from a coculture of the marine-derived fungi *Aspergillus sclerotiorum* and *Penicillium citrinum* and showed significant and selective cytotoxicity against the human histiocytic lymphoma U937 cell line (IC_50_ = 4.2 μM) [4].

Marine-sediment-derived fungus *Beauveria* *felina* KMM 4639 was previously identified as a producer of highly oxygenated chromene derivatives oxirapentyns B–K, pyran polyketide, isariketide A, and benzofuran acremine S [5,6,7]. It was suggested that this strain includes a complex of oxygenases to produce such metabolites.

Coculture of this fungal strain with *Aspergillus sulphureus* KMM 4640 resulted in the isolation of a number of new prenylated indole alkaloids and a new diorcinol J [8,9]. Oxidative prenylated compounds were not produced by a monoculture of *A. sulphureus* but were usual for the *B. felina* strain. Thus, prenylation and further oxygenation may be the result of *B. felina*’s prenyltransferase and oxygenase action.

With the aim of obtaining new bioactive compounds, the fungus *Beauveria* *felina* KMM 4639 was cocultured with another *Aspergillus* genus fungus, *Aspergillus carneus* KMM 4638, which is known as a producer of drimane sesquiterpenoids, prenylated indole alkaloids, carneamides A–C, quinazoline derivatives, carnequinazolines A–D, oxepin-containing alkaloids, and dihydrocinereain [10,11].

**Figure 1:**
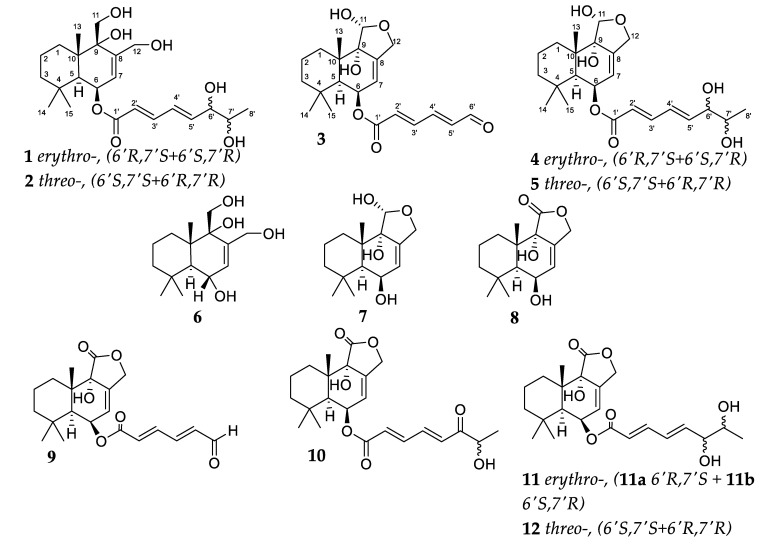
The structure of isolated compounds **1**–**12**.

Herein, we report the isolation and structural elucidation of three new drimane-type sesquiterpenes, asperflavinoids B (**2**), D (**4**) and E (**5**) (Figure 1), together with the known compounds asperflavinoid A (**1**) [12], sesquiterpene ester (**3**) [13], 12-hydroxyalbrassitriol (**6**) [14], pereniporin A (**7**) [15], strobilactone A (**8**) [16], ustusolate E (**9**) [17], (6-strobilactone A) ester of (*E*,*E*)-6-carbonyl-7-hydroxy-2,4-octadienoic acid (**10**) [18], and (6-strobilactone A) esters of (*E*,*E*)-6,7-dihydroxy-2,4-octadienoic acid (**11**, **12**) [17]. Moreover, we evaluated the cytotoxic activity of isolated compounds toward a number of cell lines.

## 2. Results

### 2.1. Isolation and Identification of Compounds

**Figure 2:**
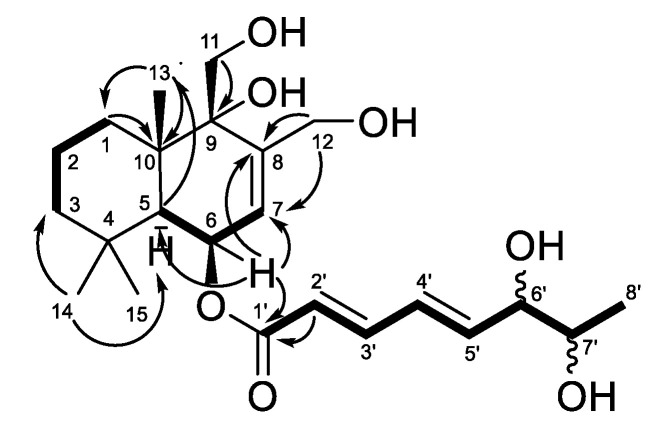
Key HMBC (arrows) and ^1^H-^1^H COSY (bold lines) correlations of compound **1**.

Compound **1** was isolated as an amorphous solid, and the HRESIMS provided a molecular formula of C_23_H_36_O_7_, as confirmed by ^13^C NMR data. The signals of 1D NMR spectra of compound **1** (Table 1 and Table 2 and Figures S15 and S16), as well as HMBC and COSY correlations (Figure 2), undoubtedly established the planar structure of compound **1**. The structure of compound **1** and the chemical shifts in its ^1^H NMR spectrum registered in DMSO-d_6_ (Figure S21) fully correspond to those of recently reported asperflavinoid A [12].

The relative stereochemistry of the side chain of compound **1** was determined using its acetonide derivative (**1a**). The small coupling constant (*J*_6′,7′_ = 6.0 Hz) and dissimilar magnetic environment of acetonide methyls (Δ = 0.12 ppm) (Figure S53) indicate an *erythro* configuration of the diol group at positions C-6′ and C-7′ [19]. The absolute configurations of the chiral centers of the drimane core in compound **1** were defined as 5*S*,6*R*,9*S*,10*S* based on biogenetic considerations. An analysis of the literature data indicates that the configurations of the C-5, C-9, and C-10 asymmetric centers remain unchanged, enabling the use of X-ray diffraction data [20,21], as well as the CD spectral data [22] of known drimanes in projection to asperflavinoid A.

The splitting of a number of ^1^H and ^13^C NMR signals (assigned to the octadiene side chain) indicated that compound **1** is a mixture of two compounds in a 1:1 ratio. A similar case was previously reported for asperflavinoid A; therefore, compound **1** is obviously a mixture of stereoisomers at the hydroxy groups in the side chain: 5*S,*6*R,*9*S,*10*S,*6′*R,*7′*S* and 5*S,*6*R,*9*S,*10*S,*6′*S,*7′*R.*

Xu et al. reported asperflavinoid A as a mixture of three stereoisomers at positions C-6 and C-7 (without specifying which ones), which, upon separation, quickly reverted to the original mixture [12]. This was not confirmed by our experimental data or by the data reported other authors who described drimanes with the same side chain [22,23]. Although we were unable to separate the two stereoisomers of compound **1**, compound **11**, which has an identical side chain, was separated into two individual diastereomers, asperienes C (**11a**) and D (**11b**). After two days, these compounds remained unchanged.

The molecular formula of compound **2** was determined as C_23_H_36_O_7_ according to the HRESIMS peak at *m*/*z* 447.2347 [M + Na]^+^, in accordance with the ^13^C NMR data. A close inspection of the ^1^H and ^13^C NMR data (Table 1 and Table 2 and Figures S22–S25) of compound **2** by DEPT and HSQC revealed the presence of a drimane sesquiterpene skeleton with an open C ring and fatty acyl moiety with two hydroxy functionalities. The main ^1^H–^1^H COSY and HMBC correlations (Figures S24 and S26) indicated that compound **2** has the same planar structure as compound **1**.

The acetonide derivative (**2a**) of compound **2** was prepared for further investigation of the stereochemistry at the diol position in fatty acid. The large coupling constant (*J*_6′,7′_ = 8.0 Hz) and the almost identical magnetic environment of the acetonide methyls (Δ = 0.00 ppm) (Figure S54) indicate a *threo* configuration of the diol group at positions C-6′ and C-7′ [19].

The absolute configurations of the chiral centers of the drimane core in compound **2** were defined based on a combination of ROESY (Figure S27) correlations (H-5 (δ_H_ 2.09)/H_3_-15 (δ_H_ 0.97), H-6 (δ_H_ 6.64); H-6/H_3_-15; H_3_-13 (δ_H_ 1.23)/H_2_-11 (δ_H_ 3.70, 3.75)) and biogenetic considerations, such as 5*S*6*R*9*S*10*S*.

Like compound **1**, the NMR spectra of compound **2** indicated the presence of a mixture of two diastereomers in solution. Obviously, compound **2** is a mixture of stereoisomers 5*S,*6*R,*9*S,*10*S,*6′*S,*7′*S* and 5*S,*6*R,*9*S,*10*S,*6′*R,*7′*R* and was named asperflavinoid B.

The molecular formula of compound **3** was determined as C_21_H_28_O_6_ according to the HRESIMS peak at *m*/*z* 399.1772 [M + Na]^+^, in accordance with the ^13^C NMR data. The ^1^H and ^13^C NMR (Table 1 and Table 2 and Figures S28–S32), as well as the DEPT and HSQC spectra, showed many similarities with NMR data of known pereniporin A (**7**) [15] but with upfielded methyl signals, downfielded CH-5 and CH-6 signals, four additional olefinic methine groups (δ_H_ 6.29, δ_C_ 130.1; δ_H_ 6.43, δ_C_ 137.4; δ_H_ 7.38, δ_C_ 141.1; δ_H_ 7.17, and δ_C_ 147.0), one aldehyde (δ_H_ 9.68 and δ_C_ 192.9), and one carboxyl group (δ_C_ 164.9).

**Figure 3:**
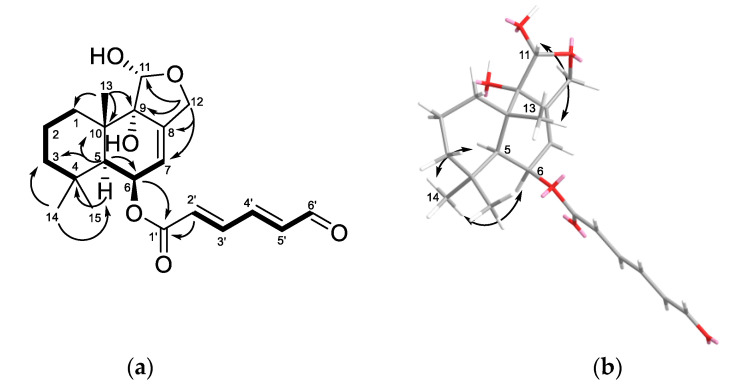
(**a**) Key HMBC (arrows), ^1^H-^1^H COSY (bold lines), and (**b**) ROESY correlations of compound **3**.

The HMBC and COSY correlations (Figure 3 and Figure S33) proved the presence of a pereniporin A drimane moiety in compound **3**.

The ^1^H–^1^H COSY correlation system from H-2′ to H-6′, in combination with HMBC correlations H-6 (δ_H_ 5.75)/C-1′ (δ_C_ 164.9); H-2′ (δ_H_ 6.29)/C-1′ indicated the presence of a 6-oxohexadienoic acid moiety in compound **3** and its location at position C-6 (δ_C_ 68.1). Large coupling constants (*J*_2′,3′_ = 15.2 Hz, *J*_4′,5′_ = 15.4 Hz) indicated an *E* configuration for both double bonds of this moiety.

The absolute configurations of the chiral centers of the drimane core in compound **3** were defined based on ROESY (Figure 3 and Figure S34) correlations H_3_-14 (δ_H_ 1.01)/H-5 (δ_H_ 2.11), H-6 (δ_H_ 5.75), and H-11 (δ_H_ 5.38)/H_3_-13 (δ_H_ 1.24), as well as biogenetic considerations, such as 5*S,*6*R,*9*S,*10*S,*11*R*. Esterification of compound **3** with (*S*)- and (*R*)-MTPA-Cl led to destruction of the compound. Compound **3** was named asperflavinoid C. An unnamed compound with the same structure was previously reported in [13], but the report did not include any detailed NMR data that can be used for identification and comparison.

The HRESIMS of compounds **4** and **5** showed [M–H]^–^ peaks at *m*/*z* 445.2190 and *m*/*z* 445.2195, respectively. These data, coupled with ^13^C NMR spectroscopic data (DEPT), established the molecular formula of compounds **4** and **5** as C_23_H_34_O_7_ for both compounds. The ^1^H and ^13^C NMR data (Table 1 and Table 2, Figures S35–S47) observed for these metabolites closely resembled those of asperflavinoid C (**3**), with the exception of the proton and carbon signals for the fatty acyl moiety. The correlations observed in the ^1^H–^1^H COSY and HMBC spectra of compounds **4** and **5** (Figures S38, S40, S44, and S46) established the presence of a dihydroxyoctadienoic acid residue attached to the C-6 carbon atom of the drimane core in their molecules.

The chemical shifts of C-6′ (δ_C_ 75.5 and 77.3) and C-7′ (δ_C_ 70.3 and 70.8) indicated the location of hydroxyl groups in these atoms and large coupling constants an *E* configuration of double bonds. The NMR data of the side chain of compound **4** ((δ_C_ 17.8 (C-8′), δ_H_ 4.23 (H-6′), and δ_H_ 3.94 (H-7′)) suggested the compound to be the *erythro* isomer, whereas those of compound **5** ((δ_C_ 19.3 (C-8′), δ_H_ 4.01 (H-6′), and δ_H_ 3.70 (H-7′)) suggested the presence of a *threo* isomer [17]. Attempts to obtain an acetonide of compounds **4** and **5** were unsuccessful. Esterification of compounds **4** and **5** with (*S*)- and (*R*)-MTPA-Cl led to destruction of the compounds, making it impossible to establish the absolute configuration using the modified Mosher method. The absolute configurations of the chiral centers of the drimane core in compounds **4** and **5** were defined based on a combination of ROESY (Figure S47) correlations and biogenetic considerations, as for asperflavinoid C (**3**). Thus, compounds **4** and **5** were named asperflavinoids D and E, respectively. As described above for compounds **1** and **2**, the NMR spectra of compound **4** and **5** indicated that they are mixtures of the stereoisomers 5*S,*6*R,*9*S,*10*S,*11*R,*6′*R,*7′*S* + 5*S,*6*R,*9*S,*10*S,*11*R*, 6′*S,*7′*R* and 5*S,*6*R,*9*S,*10*S,*11*R,*6′*S,*7′*S* + 5*S,*6*R,*9*S,*10*S,*11*R*, 6′*R,*7′*R,* respectively.

The structures of known compounds 12-hydroxyalbrassitriol (**6**) [14]; pereniporin A (**7**) [15]; strobilactone A (**8**) [16]; ustusolate E (**9**) [17]; (6-strobilactone A) ester of (*E,E*)-6-carbonyl-7-hydroxy-2,4-octadienoic acid (**10**) [18]; and asperienes C (**11a**), D (**11b**), and B (**12**) [22,23] were determined based on HRESIMS and NMR data, as well as comparison with literature data.

**Figure 4:**
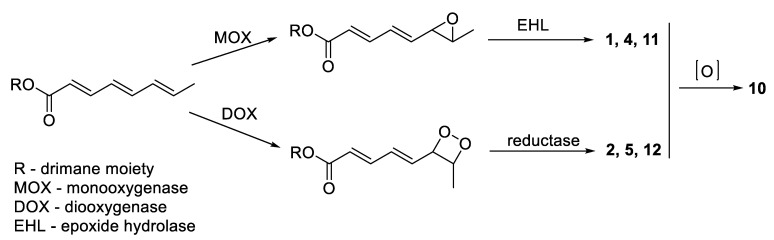
Proposed biosynthesis of the oxygenated side chains in the isolated compounds.

Although all isolated compounds are closely related, and many of them were previously described as fungal metabolites; only strobilactone A (**8**) was previously isolated from a monoculture of the fungus *A. carneus* KMM 4638 [10,11]. Other drimane derivatives may be products of the influence of coculture with *B. felina*. Moreover, compounds **1**, **2**, **4**, **5**, and **10**–**12** are probably the results of the action of oxygenases from *B. felina* on an octatrienoic acid residue (Figure 4) [7], in accordance with the previously obtained results of the mixed cultivation of *B. felina* KMM 4639 with *Aspergillus sulphureus* KMM 4640 [9]. On the other hand, the hydroxylation of the double bond of the side chain may be non-enzymatic. Thus, further research is required to confirm these assumptions.

### 2.2. Biological Activity of Compounds

Isolated compounds **1**–**5** and **8**–**12** were tested for cytotoxic activity against a number of cell lines, including rat glioblastoma C6, human breast cancer MCF-7, human prostate cancer PC-3, and human lymphoma Raji, as well as normal rat cardiomyocyte H9c2 cell lines (Table 3). Compounds **6** and **7** were isolated in quantities insufficient for bioassays.

Strobilactone A (**8**) did not influence the viability of the tested used cells up to a concentration of 100 µM. Stereoisomers **1**–**2**, **4**–**5**, and **11**–**12** caused 50% viability inhibition of various cells at concentrations of 54.1–97.5 µM or were nontoxic.

Compounds **3** and **9** decreased the viability of H9c2, C6, and Raji cells, with IC_50_ values of 54.3–88.1 µM. Compound **3** was more toxic against MCF-7 cells, with an IC_50_ of 33.8 µM and against PC-3 cells, with an IC_50_ of 50.8 µM. Compound **9** inhibited MCF-7 and PC-3 cell viability by 50% at concentrations of 57.8 µM and 50.1 µM, respectively.

MCF-7 cells were more sensitive to the toxic influence of the investigated compounds than other cancer cell lines tested in our experiments. Therefore, subsequent experiments were carried out using the MCF-7 cell line.

**Figure 5:**
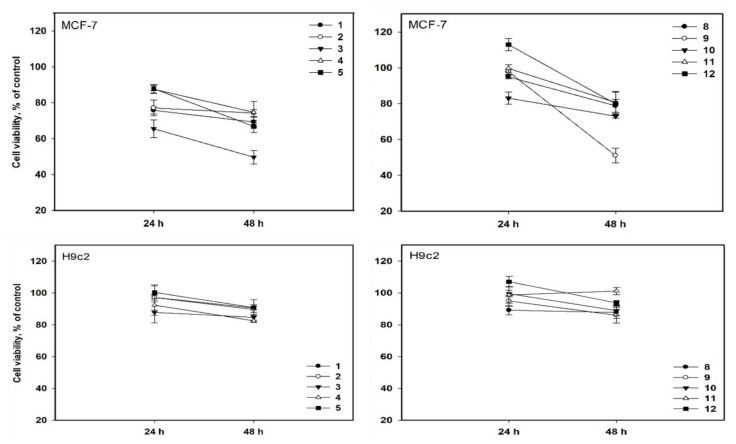
Cytotoxic activity of compounds **1**–**5** and **8**–**12** at a concentration of 10 µM against MCF-7 and H9c2 cells. All experiments were carried out in three independent replicates, and data are presented as mean ± standard error of the mean (SEM).

The prolonged incubation (48 h) of MCF-7 cells with compounds **1**–**5** and **8**–**12** at concentrations up to 10 µM resulted in an increase in the cytotoxic effect of the compounds. Nonetheless, only compounds **3** and **9** caused a decrease in MCF-7 cell viability of nearly 50%, IC_50_ values calculated as 10.0 ± 0.8 µM and 10.1 ± 0.5 µM, respectively. Furthermore, the toxicity of all compounds against the normal cardiomyocyte H9c2 line was lower, with a viability of H9c2 cells of more than 80% when treated for 48 h (Figure 5).

To further investigate the cytotoxic activity of the most effective compounds, we investigated the MCF-7 cell cycle, apoptosis level, and caspase activity after incubation with **3** and **9** at a concentration of 10 µM for 48 h.

**Figure 6:**
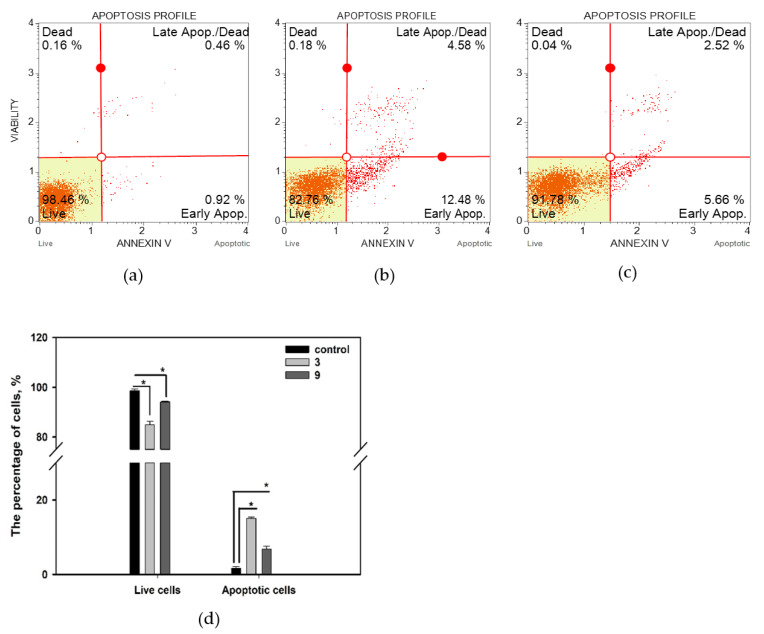
Influence of compounds **3** and **9** at a concentration of 10 µM for 48 h on MCF-7 cell apoptosis profiles. (**a**) Untreated cells; (**b**) cells treated with compound **3**; (**c**) cells treated with compound **9**; (**d**) summary graph, with early apoptotic and late apoptotic cells presented as “apoptotic cells”. All experiments were carried out in three independent replicates, and data are presented as mean ± standard error of the mean (SEM). * indicates significant differences; *p* < 0.05.

We found that compounds **3** and **9** significantly changed the apoptotic profile of MCF-7 cells (Figure 6). Control MCF-7 cells comprised only 1.7% early and late apoptotic cells, and treating the cells with compounds **3** and **9** caused an increased the amount of apoptotic cells to 15.0% and 6.8%, respectively. Accordingly, the amount of living cells was decreased from 98.6% in the control population to 84.9% and 94.1% in cells treated with compounds **3** and **9**, respectively.

**Figure 7:**
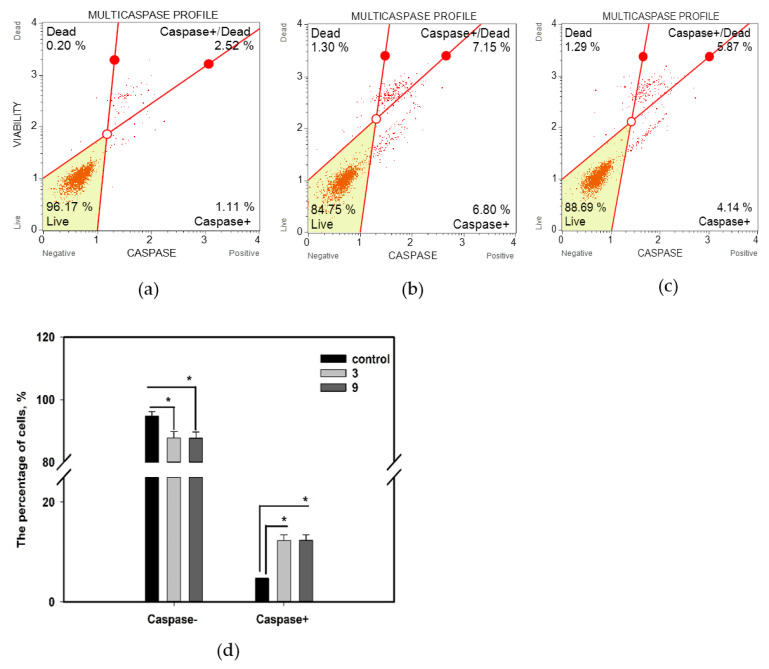
Influence of compounds **3** and **9** at a concentration of 10 µM for 48 h on total caspase activity in MCF-7 cells. (**a**) Untreated cells; (**b**) cells treated with compound **3**; (**c**) cells treated with compound **9**; (**d**) summary graph. All experiments were carried out in three independent replicates, and data are presented as mean ± standard error of the mean (SEM). * indicates significant differences; *p* < 0.05.

Changes in the apoptosis profile of MCF-7 cells after treatment with compounds **3** and **9** were accompanied by an increase in total caspase activity (Figure 7).

The influence of the compounds on the percentage of live MCF-7 cells (caspase−/7-AAD−), live MCF-7 cells with caspase activity (caspase+/7-AAD−), dead MCF-7 cells with caspase activity (caspase+/7-AAD−), and dead MCF-7 cells (caspase−/7-AAD+) was measured by a Muse^®^ multicaspase kit (Luminex, Austin, TX, USA). The kit utilizes a VAD-peptide derivatized with a fluorescent group called fluorescent-labeled inhibitor of caspases (FLICA) [24]. The peptide binds to activated caspases, resulting in a fluorescent signal proportional to the number of active caspases in the cell. Fluorescent dye 7-AAD was used as a dead cell marker in this assay.

Both compounds **3** and **9** significantly increased the percentage of living cells exhibiting caspase activity and 7-AAD-labeled cells exhibiting caspase activity to 12.3% and 12.4%, respectively, whereas control cell population comprised only 4.7% of cells exhibiting caspase activity. The effect of compounds **3** and **9** on caspase activity in MCF-7 cells was the same, in contract their influence on the cell apoptotic profile.

**Figure 8:**
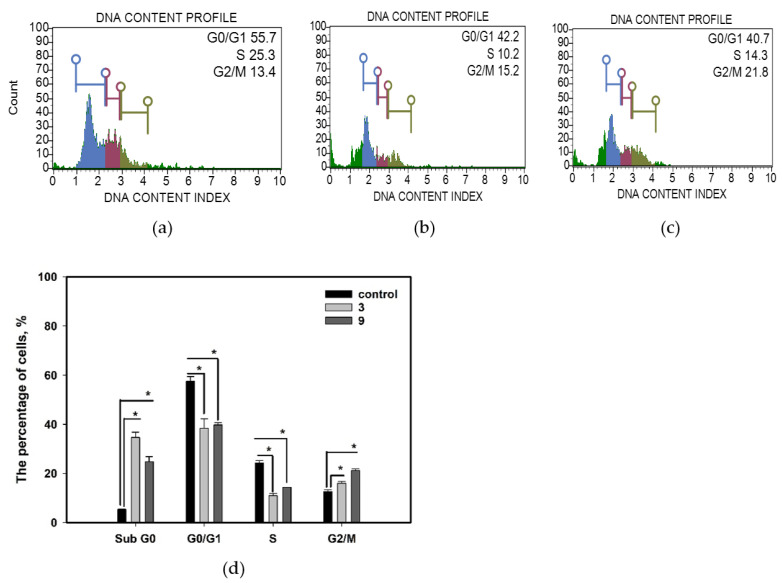
Influence of compounds **3** and **9** at a concentration of 10 µM for 48 h on the MCF-7 cells cycle. (**a**) Untreated cells; (**b**) cells treated with compound **3**; (**c**) cells treated with compound **9**; (**d**) summary graph. All experiments were carried out in three independent replicates, and data are presented as a mean ± standard error of the mean (SEM). * indicates significant differences; *p* < 0.05.

The influence of compounds **3** and **9** on the MCF-7 cell cycle is presented in Figure 8. Compounds **3** and **9** significantly reduced the percentage of cells in the G0/G1 phase by 30.9% and 33.3%, respectively. Moreover, we observed a decrease in the percentage of cells in the S phase due to the action of compounds **3** and **9** by 54.5% and 41.2%, respectively. Furthermore, the percentage of cells in the G2/M phase was significantly increased by 25.2% in the case of compound **3** and by 67.3% in the case of compound **9**. Additionally, after treatment with compounds **3** and **9**, the subG0 population of cells was dramatically increased by 6.4 and 4.6 times, respectively. The control cells comprised only 5.4% in the subG0 zone, whereas cells treated with compounds **3** and **9** comprised 34.6% and 24.7% subG0 cells, respectively. The decrease in cells in the G0/G1 and S phases and accumulation in subG0 phase were apparently caused by cell death after incubation with compounds **3** and **9**, whereas cell cycle arrest was observed in the G2/M phase after incubation with compounds **3** and **9**.

Thus, compounds **3** and **9** significantly decreased MCF-7 cell viability via the apoptosis caspase-dependent pathway, as well as cell cycle arrest in the G2/M phase. The effect of both compounds on MCF-7 cell viability was similar, but compound **3** was a stronger inducer of apoptosis than compound **9**, whereas compound **9** arrested the MCF-7 cell cycle more than compound **3**. These compounds are interesting targets for future investigations as anticancer agents, as they affected in vitro breast cancer MCF-7 cells but not normal cardiomyocyte H9c2 cells.

The cytotoxic activity of some drimane sesquiterpenoids was previously reported by several research groups. Additionally, the cytotoxic effect of asperflavinoid A (**1**) against human hepatocarcinoma HepG2 and gastric cancer MKN-45 cells was previously reported, with IC_50_ values of 84.4 and 63.2 μM, respectively [12]. Moreover, it was reported that compound **10** decreased the viability of murine leukemia P388 cells, with an IC_50_ value of 8.7 µM [18], and compound **11**(**12**) at a concentration of 120 μM decreased the viability of human leukemia MOLT-4 cells by 72% [25]. However, in our experiments, both of these compounds exhibited very weak toxicity against human leukemia Raji cells. Ustusolate E (**9**) was previously reported to significantly decrease the viability of human leukemia HL-60 cells, with an IC_50_ of 8 µM, but was nontoxic against human lung cancer A549 cells [26]. Moreover, compound **9** decreased the viability of lymphoma L5178Y, PC 12, and HeLa cells, with IC_50_ values of 1.6, 19.3, and 15.8 μM, respectively [17]. In our experiment, we did not observe a selective effect of compound **9** on human leukemia Raji cells in comparison with other investigated cell lines. Obviously, the chemical structure of isolated drimane sesquiterpenoids exerts a significant influence on their anticancer activity. Both of the most active isolated compounds, **3** and **9**, have aldehyde functionalities in their side chains, which probably play a key role in the observed activity.

Compound **3** was previously patented as an inhibitor of PTP1B and SHP2 enzyme activity only in cell-free assays, with IC_50_ values of 2.8 and 3.0 µg/mL, respectively. Compound **9** also was found to be an inhibitor of PTP1B and SHP2 enzymes, with IC_50_ values of 50 and 40 µg/mL, respectively [13]. It has been suggested that inhibition of the PTP1B enzyme in breast cancer cells can result in delayed tumor formation via several pathways [27]. PTP1B inhibitors, such as docosahexaenoic acid [28] and cinnamaldehyde [29], affected human breast cancer MCF-7 cell viability. Inhibition of PTP1B results in decreased cell adhesion, loss of extracellular matrix attachment and apoptosis, AMPK activation, and autophagy [28].

Thus, PTP1B inhibition may be one of the pathways explaining the anticancer activity of compounds **3** and **9** toward MCF-7 cells in our experiments. Nevertheless, compounds **3** and **9** have a similar effect on MCF-7 cell viability, although their reported effect on PTP1B activity differed by ten times, perhaps indicating that other pathways also participate in the cytotoxic action of compounds **3** and **9**. Our flow cytometry data evidence that apoptosis in MCF-7 cells treated with compounds **3** and **9** is activated via a caspase-dependent pathway. Moreover, MCF-7 cell cycle arrest was detected in the G2/M phase as a result of the action of compounds **3** and **9**.

We observed that compounds **3** and **9** at a concentration of 10 µM for 48 h decreased MCF-7 cell viability by nearly 50% in MTT tests, whereas 7-AAD/annexin V straining resulted in an increase in the amount of apoptotic cells to 15.0% and 6.8%, respectively. MTT reagent is metabolized by various cellular enzymes, and inhibition of its activity results in a decrease in formazan production, which is reflected by a decrease in the viability of cells. Nonetheless, the decrease in formazan production could be a result of not only cell apoptosis but also a consequence of cell death via other mechanisms or inhibition of cell adhesion.

## 3. Materials and Methods

### 3.1. General Experimental Procedures

Optical rotations were measured on a Perkin-Elmer 343 polarimeter (Perkin Elmer, Waltham, MA, USA). UV spectra were recorded on a Shimadzu UV-1601PC spectrometer (Shimadzu Corporation, Kyoto, Japan) in methanol. CD spectra were measured with a Chirascan-Plus CD spectrometer (Leatherhead, UK) in methanol. NMR spectra were recorded in CDCl_3_, acetone-*d*_6_, and DMSO-*d*_6_ on a Bruker DPX-300 (Bruker BioSpin GmbH, Rheinstetten, Germany), a Bruker Avance III-500 (Bruker BioSpin GmbH, Rheinstetten, Germany), and a Bruker Avance III-700 (Bruker BioSpin GmbH, Rheinstetten, Germany) spectrometer, using TMS as an internal standard. HRESIMS spectra were measured on a Maxis impact mass spectrometer (Bruker Daltonics GmbH, Rheinstetten, Germany). Microscopic examination and photography of fungal cultures were performed with an Olympus CX41 microscope equipped with an Olympus SC30 digital camera. Detailed examination of the ornamentation of the fungal conidia was performed using and EVO 40 scanning electron microscope (SEM).

Low-pressure liquid column chromatography was performed using silica gel (60/100 μm, Imid Ltd., Krasnodar, Russia) and ODS-A gel (12 nm, S—75 um, YMC Co., Ishikawa, Japan). Plates precoated with silica gel (5–17 μm, 10 cm × 10 cm, Imid Ltd., Krasnodar, Russia) and 60 RP-18 F_254_S silica gel (20 cm × 20 cm, Merck KGaA, Darmstadt, Germany) were used for thin-layer chromatography. Preparative HPLC was carried out on an Agilent 1100 chromatograph (Agilent Technologies, Santa Clara, CA, USA) with an Agilent 1100 refractometer (Agilent Technologies, Santa Clara, CA, USA) and a Shimadzu LC-20 chromatograph (Shimadzu USA Manufacturing, Canby, OR, USA) with a Shimadzu RID-20A refractometer (Shimadzu Corporation, Kyoto, Japan) using YMC ODS-AM (YMC Co., Ishikawa, Japan) (5 µm, 10 mm × 250 mm), YMC ODS-A (YMC Co., Ishikawa, Japan) (5 µm, 4.6 mm × 250 mm) and Hydro-RP (Phenomenex, Torrance, CA, USA) (4 μm, 250 mm × 10 mm) columns.

### 3.2. Fungal Strain

The *A. carneus* fungal strain was isolated from superficial mycobiota of the brown alga *Laminaria sachalinensis* (Miyabe) collected on Kunashir Island and was identified based on morphological evaluation by Dr. Mikhail V. Pivkin from the Pacific Institute of Bioorganic Chemistry (PIBOC). The strain is stored in the Collection of Marine Microorganisms, PIBOC, Vladivostok, Russia, under the code KMM 4638.

The *Beauveria felina* fungal strain was isolated from marine sediments collected at a depth of 10 m (Van Phong Bay, the South China Sea, Vietnam) during 34th expedition of r/v “Akademik Oparin” and was identified based on morphological evaluation by Dr. Natalya N. Kirichuk from the Pacific Institute of Bioorganic Chemistry (PIBOC). The strain is stored in the Collection of Marine Microorganisms, PIBOC, Vladivostok, Russia, under the code KMM 4639.

### 3.3. Cultivation of Fungus

The fungi *A. carneus* and *B. felina* were cultivated separately at 22 °C for 7 days in Erlenmeyer flasks (500 mL), each containing 20 g of rice, 20 mg of yeast extract, 10 mg of KH_2_PO_4_, and 40 mL of natural sea water. *B. felina* mycelium was inoculated into 20 flasks with *A. carneus* culture. The fungus was inoculated with three pieces of mycelium (~0.5 × 0.5 mm) in each flask. Then, fungal cultures were cocultivated for 14 days.

### 3.4. Extraction and Isolation

At the end of the incubation period, the mycelium and medium were homogenized and extracted with EtOAc (4 L). The extract was concentrated to dryness. The residue was dissolved in 20% EtOH–H_2_O (1 L) and was extracted with *n*-hexane (200 mL × 3), EtOAc (200 mL × 3), and *n*-BuOH (150 mL × 2). After evaporation of the EtOAc layer, the residual material (6 g) was passed over silica columns (4 × 20 cm), which was eluted first with *n*-hexane (2 L), followed by a step gradient from 5% to 100% EtOAc in *n*-hexane (total volume 10 L). Fractions of 200 mL were collected and combined on the basis on TLC (silica gel, toluene–isopropanol 6:1, *v*/*v*).

The *n*-hexane–EtOAc (90:10) eluate (708 mg) was separated on a Gel ODS-A column (1.5 × 8 cm), which was eluted using a step gradient from 40% to 80% CH_3_OH in H_2_O (total volume 1 L) to yield subfraction F1. Subfraction F1 (60% CH_3_OH, 150 mg) was purified by RP HPLC on a YMC ODS-AM column eluted with CH_3_CN-H_2_O (60:40) to yield individual compound **9** (8 mg) and fractions F1-1 (62 mg) and F1-2 (14 mg). Fraction F1-1 was purified by RP HPLC on a hydro-RP column eluted with CH_3_CN-H_2_O (40:60) to yield compounds **11** (7.7 mg) and **12** (6.5 mg). Fraction F1-2 was purified by RP HPLC on a hydro-RP column eluted with CH_3_CN-H_2_O (50:50) to yield compound **10** (3.7 mg).

The *n*-hexane-EtOAc (80:20, 646 mg) fraction was separated on an ODS-A gel column (1.5 × 8 cm), which was eluted using a step gradient from 40% to 80% CH_3_OH in H_2_O (total volume 1 L) to yield subfraction E1. Subfraction E (60% CH_3_OH, 205 mg) was purified by RP HPLC on a YMC ODS-AM column eluted with CH_3_OH-H_2_O (80:20) to yield fractions E1-2 (125.5 mg) and E1-2 (6.0). Fraction E1-1 was purified by RP HPLC on a YMC ODS-A column eluted with CH_3_CN-H_2_O (40:60), then with CH_3_OH-H_2_O (10:90) to yield compounds **4** (4.0 mg), **5** (2.3 mg), and **8** (6.5 mg). Fraction E1-2 was purified by RP HPLC on a hydro-RP column eluted with CH_3_CN-H_2_O (60:40) to yield compound **3** (3.0 mg).

The *n*-hexane-EtOAc (70:30, 690 mg) fraction was separated on an ODS-A gel column (1.5 × 8 cm), which was eluted by a step gradient from 40% to 80% CH_3_OH in H_2_O (total volume 1 L) to yield subfractions A1 and A2. Subfraction A1 (60% CH_3_OH, 282 mg) was purified by RP HPLC on a YMC ODS-AM column eluted with CH_3_OH-H_2_O (70:30) and then on a YMC ODS-A column eluted with CH_3_CN-H_2_O (40:60) to yield compound **7** (1.0 mg). Subfraction A2 (80% CH_3_OH, 328 mg) was purified by RP HPLC on a YMC ODS-AM column eluted with CH_3_OH-H_2_O (80:20) and then on a hydro-RP column eluted with CH_3_CN-H_2_O (40:60) to yield compounds **1** (1.3 mg), **2** (1.2 mg), and **6** (2.0 mg).

### 3.5. Spectral Data

Asperflavinoid A (**1**): amorphous solid; [α]_D_^20^ –149.6 (*c* 0.13, MeOH); CD (6.6 × 10^−4^, MeOH), λ_max_ (∆*ε*) 199 (−14.33), 261 (−2.69) nm (Figure S1); UV (CH_3_OH) *λ*_max_ (log *ε*) 261 (4.41), 225 (3.96), 196 (4.32) nm (Figure S8); ^1^H and ^13^C NMR data (Table 1 and Table 2, Figures S15–S21); HRESIMS *m*/*z* 423.2392 [M – H]^–^ (calcd. for C_23_H_35_O_7_, 423.2388, Δ 0.8 ppm), 447.2350 [M + Na]^+^ (calcd. for C_23_H_36_O_7_Na, 447.2353, Δ 0.8 ppm).

Asperflavinoid B (**2**): amorphous solid; [α]_D_^20^ –167.1 (*c* 0.08, MeOH); ^1^H and ^13^C NMR data, see Table 1 and Table 2, (Figures S22–S27); HRESIMS *m*/*z* 423.2389 [M – H]^–^ (calcd. for C_23_H_35_O_7_, 423.2388, Δ 0.1 ppm), 447.2347 [M + Na]^+^(calcd. for C_23_H_36_O_7_Na, 447.2353, Δ 1.4 ppm).

Asperflavinoid C (**3**): amorphous solid; [α]_D_^20^ –258.7 (*c* 0.08, MeOH); CD (*c* 7.1 ×10^−4^, CH_3_OH), λ_max_ (∆ε) 207 (−11.98), 270 (−3.43) nm, (Figure S2); UV (CH_3_OH) *λ*_max_ (log *ε*) 270 (4.23), 228 (3.74), 197 (3.96) nm, (Figure S9); ^1^H and ^13^C NMR data, see Table 1 and Table 2, (Figures S28–S34); HRESIMS *m*/*z* 375.1812 [M – H]^–^ (calcd. For C_21_H_27_O_6_, 375.1813, Δ 0.2 ppm), 399.1772 [M + Na]^+^(calcd. For C_21_H_28_O_6_Na, 399.1778, Δ 1.5 ppm).

Asperflavinoid D (**4**): amorphous solid; [α]_D_^20^ –187.8 (*c* 0.09, MeOH); CD (*c* 5.3 × 10^−4^, CH_3_OH), λ_max_ (∆ε) 208 (−11.04), 262 (−5.13) nm, (Figure S3); UV (CH_3_OH) *λ*_max_ (log *ε*) 262 (4.30), 222 (3.66), 210 (3.74), 197 (2.91) nm, (Figure S10); ^1^H and ^13^C NMR data, see Table 1 and Table 2, (Figures S35–S40); HRESIMS *m*/*z* 421.2239 [M – H]^–^ (calcd. For C_23_H_33_O_7_, 421.2232, Δ 1.7 ppm), 445.2190 [M + Na]^+^(calcd. For C_23_H_34_O_7_Na, 445.2197, Δ 1.6 ppm).

Asperflavinoid E (**5**)*:* amorphous solid; [α]_D_^20^ –190.0 (*c* 0.13, MeOH); CD (*c* 2.5 × 10^−4^, CH_3_OH), λ_max_ (∆ε) 208 (−11.62), 262 (−4.63) nm, (Figure S4); UV (CH_3_OH) *λ*_max_ (log *ε*) 261 (4.13), 224 (3.75), 196 (4.07) nm, (Figure S11); ^1^H and ^13^C NMR data, see Table 1 and Table 2, (Figures S41–S47); HRESIMS *m*/*z* 421.2234 [M – H]^–^ (calcd. for C_23_H_33_O_7_, 421.2232, Δ 0.5 ppm), 445.2195 [M + Na]^+^(calcd. for C_23_H_34_O_7_Na, 445.2197, Δ 0.4 ppm).

### 3.6. Cell Lines and Culture Conditions

The human prostate cancer PC-3, human breast cancer MCF-7, human lymphoma Berkitta Raji cells, and rat glioblastoma C6 cells were purchased from ATCC (Manassas, VA, USA). Rat cardiomyocyte H9c2 cells were kindly provided by Prof. Dr. Gunhild von Amsberg from Martini-Klinik Prostate Cancer Center, University Hospital Hamburg-Eppendorf, Hamburg, Germany.

PC-3, MCF-7, C6, and H9c2 cells were cultured in DMEM medium (Biolot, St. Petersburg, Russia) containing 10% fetal bovine serum (Biolot, St. Petersburg, Russia) and 1% penicillin/streptomycin (Biolot, St. Petersburg, Russia) at 37 °C in a humidified atmosphere with 5% (*v*/*v*) CO_2_. The Raji cells were cultured in RPMI-1640 medium (Biolot, St. Petersburg, Russia) containing 10% fetal bovine serum (Biolot, St. Petersburg, Russia) and 1% penicillin/streptomycin (Biolot, St. Petersburg, Russia) under the same conditions.

Initially, cells were incubated in culture flasks until subconfluent (~80%). For testing, the cells were seeded at concentrations of 5 × 10^3^ cells/well (PC-3, MCF-7, Raji, and C6 cells) or 3 × 10^3^ cells/well (H9c2 cells), and experiments were started after 24 h.

### 3.7. In Vitro MTT-Based Cytotoxicity Assay

The in vitro cytotoxicity of individual substances was determined by the MTT method (3-(4,5-dimethylthiazol-2-yl)-2,5-diphenyltetrazolium bromide), according to the manufacturer’s instructions (Sigma-Aldrich, St. Louis, MO, USA).

Investigated compounds were dissolved in DMSO at a concentration of 10 mM. This solution was used to obtain the required concentration of compounds in the cell suspension so that the concentration of DMSO in the cell suspension did not exceed 1%.

The cells were treated with the investigated compounds for 24 h or 48 h, and MTT reagent was added to each well of the plate. The vehicle with DMSO at a concentration of 1% was used as a control. The absorbance of formed formazan was measured at λ = 570 nm using a Multiskan FC microplate photometer (Thermo Scientific, Waltham, MA, USA) and expressed in optical units (o.u.). The results are presented as % of viable cells relative to vehicle, and 50% inhibition concentration of cell viability (IC_50_) was calculated.

### 3.8. Flow Cytometry

#### 3.8.1. Apoptosis

After incubation, culture media were collected; then, cells were washed twice with cold PBS and incubated with trypsin-EDTA solution for 1 min. The cell suspension was washed twice by centrifugated at 250× *g* for 4 min with cold PBS and used for apoptosis detection by a Muse^®^ annexin V and dead cell kit in accordance with manufacturer’s instructions (Luminex, Austin, TX, USA). Fluorescence was measured with a Muse^®^ cell analyzer (Luminex, Austin, TX, USA), and data were processed by Muse 1.5 analysis software (Luminex, Austin, TX, USA). The proportion of apoptotic cells was expressed as a percentage.

#### 3.8.2. Total Caspase Activity

After incubation, culture media were collected, and cells were washed twice with cold PBS and incubated in trypsin-EDTA solution for 1 min. The cell suspension was washed twice by centrifugation at 250× *g* for 4 min with cold PBS and then used for caspase activity detection by a Muse^®^ multicaspase kit (caspases 1, 3, 4, 5, 6, 7, 8, and 9) in accordance with manufacturer’s instructions (Luminex, Austin, TX, USA). Fluorescence was measured with a Muse^®^ cell analyzer (Luminex, Austin, TX, USA), and data were processed by Muse 1.5 analysis software (Luminex, Austin, TX, USA). The proportion of the cells exhibiting caspase activity was expressed as a percentage.

#### 3.8.3. Cell Cycle

After incubation, cells were trypsinized, harvested, washed with PBS, and fixed with ice-cold 70% ethanol in a dropwise manner prior to storage at −20 °C overnight. The cells were then washed with PBS, incubated with 200 μg/mL RNAse (PanReac, AppliChem, Darmstadt, Germany) and 20 μg/mL of propidium iodide (Sigma-Aldrich, St. Louis, MO, USA) for 30 min at 37 °C, and the DNA content was analyzed with a Muse^®^ cell analyzer (Luminex, Austin, TX, USA). The data were processed by Muse 1.5 analysis software (Luminex, Austin, TX, USA). The proportion of cells in each phase of the cell cycle was expressed as a percentage.

### 3.9. Statistical Data Evaluation

All results are presented as mean ± standard error of the mean (SEM). General statistical analysis was performed using Student’s *t*-test employed with the aid of SigmaPlot 14.0 (Systat Software Inc., San Jose, CA, USA). Differences were considered statistically significant at *p* < 0.05.

## 4. Conclusions

Chemical investigation of a coculture of the marine-derived fungi *Beauveria felina* KMM 4639 and *Aspergillus carneus* KMM 4638 led to the identification of three new drimane-type sesquiterpenes, asperflavinoids B, D, and E (**2**, **4**, **5**), and nine previously reported related compounds. Only strobilactone A (**8**) was previously isolated from a monoculture of *Aspergillus carneus* KMM 4638. Compounds **1**, **2**, **4**, **5**, and **10**–**12** are likely the result of the action of oxygenases from *B. felina* on octatrienoic acid residue. Compounds **3** and ustusolate E (**9**) exerted a significant effect on human breast cancer MCF-7 cell viability, with an IC_50_ value of 10 µM, and induced caspase-dependent apoptosis and arrest of the MCF-7 cell cycle in the G2/M phase. Therefore, the cytotoxic activity of asperflavinoid C (**3**) and ustusolate E (**9**) against human breast cancer MCF-7 cells was reported here for the first time, with promising prospects for future investigations.

## Figures and Tables

**Table 1:** ^1^H NMR data (*δ* in ppm, *J* in Hz) for compounds **1**–**5**.

Position	1 ^a^	2 ^a^	3 ^b^	4 ^c^	5 ^c^
1	a: 1.48, d (14.5) b: 2.05, m	a: 1.48, d (14.5) b: 2.05, m	a: 1.41, m b: 1.92, td (13.1, 4.3)	a: 1.39, dt (13.1, 3.0) b: 1.92, td (13.4, 3.7)	a: 1.40, dt (13.4, 3.6) b: 1.91, td (13.2, 3.0)
2	a: 1.51, m b: 1.69, dt (13.5, 2.8)	a: 1.50, m b: 1.69, m	a: 1.53, m b: 1.66, m	a: 1.52, dt (13.6, 3.3) b: 1.66, qt (13.5, 2.6)	a: 1.53, dt (13.6, 3.6) b: 1.66, qt (13.6, 3.0)
3	a: 1.25, m b: 1.34, d (13.0)	a: 1.28, m b: 1.34, d (12.8)	a: 1.32, m b: 1.41, m	a: 1.30, td (13.3, 2.3) b: 1.39, brd (13.0)	a: 1.29, td (13.0, 2.5) b: 1.39, dt (13.4, 3.6)
5	2.09, d (4.3)	2.09, d (4.3)	2.11, d (4.4)	2.07, d (4.3)	2.07, d (4.3)
6	5.64, t (4.7)	5.64, t (4.8)	5.75, m	5.72, brs	5.72, m
7	5.86, d (5.3)	5.86, d (5.3)	5.72, m	5.71, brs	5.71, m
11	a: 3.71, d (11.7) b: 3.76, d (11.6)	a: 3.70, d (11.6) b: 3.75, d (11.6)	5.38, brs	5.37, s	5.37, s
12	a: 4.23, d (13.5) b: 4.29, d (13.4)	a: 4.23, d (13.6) b: 4.29, d (13.4)	a: 4.25, d (13.4) b: 4.62, d (13.4)	a: 4.25, m b: 4.61, d (13.3)	a: 4.24, d (13.4) b: 4.61, dt (13.3, 1.9)
13	1.23, s	1.23, s	1.24, s	1.24, s	1.23, s
14	0.97, s	0.97, s	1.01, s	1.00, s	1.00, s
15	1.13, s	1.13, s	1.14, s	1.14, s	1.14, brs
1′					
2′	5.92, d (15.3)	5.93, d (15.3)	6.29, d (15.2)	5.90, d (15.3)	5.91, d (15.3)
3′	7.28, dd (15.2, 11.0)/7.27, dd (15.2, 11.0)	7.28, dd (14.7, 11.1)/7.26, dd (14.6, 11.1)	7.38, dd (15.2, 11.3)	7.24, dd (15.3, 11.2)/7.23, dd (15.3, 11.1)	7.24, dd (15.4, 10.7)/7.22, dd (15.7, 11.0)
4′	6.52, m/6.51, m	6.55, m/6.54 m	7.17, dd (15.4, 11.2)	6.47, dd (15.2, 11.0)/6.46, dd (15.4, 10.6)	6.48, ddd (15.6, 11.0, 8.1)
5′	6.34, dd (9.1, 5.4)/6.31, dd (9.1, 5.4)	6.29, dd (15.6, 10.3)/6.26, dd (15.6, 10.6)	6.43, dd (15.4, 7.6)	6.15, dd (15.3, 5.6)/6.13, dd (15.3, 6.4)	6.10, m
6′	4.09, t (5.2)/4.07, t (5.2)	4.02, t (5.2)/4.00, t (5.6), m	9.68, d (7.6)	4.23, m	4.01, d (6.5)/3.99, d (6.5)
7′	3.71, m	3.65, m/3.64, m		3.94, m	3.70, quint (6.3)
8′	1.12, d (6.3)	1.11, d (6.4)/1.10, d (6.4)		1.16, d (6.5)	1.221, d (6.3)/1.219, d (6.3)

^a^ Chemical shifts were measured at 700.13 MHz in acetone-d_6_. ^b^ Chemical shifts were measured at 300.13 MHz in CDCl_3_. ^c^ Chemical shifts were measured at 700.13 MHz in CDCl_3_.

**Table 2:** ^13^C NMR data (*δ* in ppm) for compounds **1**–**5**.

Position	1 ^a^	2 ^a^	3 ^b^	4 ^c^	5 ^c^
1	32.9, CH_2_	32.9, CH_2_	32.0, CH_2_	32.0, CH_2_	32.0, CH_2_
2	19.4, CH_2_	19.4, CH_2_	18.1, CH_2_	18.1, CH_2_	18.1, CH_2_
3	45.3, CH_2_	45.3, CH_2_	44.8, CH_2_	44.8, CH_2_	44.8, CH_2_
4	34.4, C	34.4, C	33.9, C	33.5, C	33.8, C
5	46.1, CH	46.1, CH	45.8, CH	45.9, CH	45.9, CH
6	67.4, CH	67.4, CH	68.1, CH	67.3, CH	67.2, CH
7	124.6, CH	124.6, CH	119.6, CH	120.2, CH	120.0, CH
8	144.61/144.58, C	144.5, C	142.2, C	141.6, C	141.6, C
9	76.3, C	76.5, C	77.4, C	75.6 C	78.0, C
10	41.4, C	41.4, C	38.6, C	38.5, C	38.5, C
11	63.0, CH_2_	63.0, CH_2_	98.2, CH	98.3, CH	98.3, CH
12	64.4, CH_2_	64.4, CH_2_	66.7, CH_2_	66.7, CH_2_	66.7, CH_2_
13	19.2, CH_3_	19.2, CH_3_	19.0, CH_3_	19.0, CH_3_	18.9, CH_3_
14	33.3, CH_3_	33.3, CH_3_	33.0, CH_3_	33.0, CH_3_	33.0, CH_3_
15	25.2, CH_3_	25.2, CH_3_	24.9, CH_3_	24.8, CH_3_	24.8, CH_3_
1′	166.63/166.62, C	166.6, C	164.9, C	166.2, C	166.14/166.12, C
2′	122.21/122.15, CH	122.4/122.3, CH	130.1, CH	122.43/122.38, CH	122.62/122.56, CH
3′	145.40/145.38, CH	145.3/145.2, CH	141.1, CH	144.1, CH	144.02/143.99, CH
4′	129.0/128.8, CH	129.3/129.2, CH	147.0, CH	130.1/129.9, CH	130.0/129.8, CH
5′	144.61/144.58, CH	144.32/144.26, CH	137.4, CH	140.2, CH	141.3/141.2, CH
6′	76.4, CH	76.7, CH	192.9, CH	75.5, C	77.3, C
7′	70.96/70.92, CH	70.9, CH		70.3, CH	70.8, CH
8′	18.90/18.87, CH_3_	19.0, CH_3_		17.8, CH_3_	19.3, CH_3_

^a^ Chemical shifts were measured at 176.04 MHz in acetone-d_6_. ^b^ Chemical shifts were measured at 75.47 MHz in CDCl_3_.^c^ Chemical shifts were measured at 176.04 MHz in CDCl_3_.

**Table 3:** Cytotoxic activity of compounds **1**–**5** and **8**–**12**.

Compound	Cell Lines				
	H9c2	C6	PC-3	Raji	MCF-7
	IC_50_, µM				
**1**	84.2 ± 3.3	102.2 ± 1.7	85.3 ± 2.9	>100	59.0 ± 4.4
**2**	74.4 ± 4.4	>100	75.2 ± 3.8	>100	54.1 ± 2.4
**3**	55.6 ± 2.6	56.0 ± 1.2	50.8 ± 1.6	82.3 ± 2.9	33.8 ± 2.2
**4**	59.3 ± 1.7	>100	57.4 ± 2.4	85.7 ± 6.0	75.0 ± 6.2
**5**	63.9 ± 1.6	>100	59.1 ± 0.8	94.4 ± 2.3	80.6 ± 3.5
**8**	>100	>100	>100	>100	>100
**9**	66.9 ± 3.0	54.3 ± 3.3	50.1 ± 1.8	88.1 ± 1.3	57.8 ± 0.5
**10**	61.9 ± 0.5	>100	57.8 ± 1.1	95.3 ± 0.8	55.1 ± 1.4
**11**	>100	>100	>100	96.9 ± 1.6	83.5 ± 1.7
**12**	>100	>100	>100	97.5 ± 0.9	81.7 ± 0.9

The cells were incubated with the isolated compounds for 24 h, and the viability of cells was measured by MTT assay. All experiments were carried out in three independent replicates, and data are presented as mean ± standard error of the mean (SEM).

## Data Availability

Not applicable.

## Supplementary Materials

The following are available online at https://www.mdpi.com/article/10.3390/md20090584/s1, Figures S1–S7: ECD spectra of compounds **1**, **3–5**, **10–12**, Figures S9–S14: UV spectra of compounds **1**, **3–5**, **10–12**, Figures S15–S54: NMR spectra of compounds **1**–**5, 9–12**.
