# Supplementary material for: Cytotoxic Drimane-Type Sesquiterpenes from Co-Culture of the Marine-Derived Fungi Aspergillus carneus KMM 4638 and Beauveria felina (=Isaria felina) KMM 4639
Source: Mar Drugs. 2022 Sep 19;20(9):584. doi: 10.3390/md20090584 (PMC9504587; doi:10.3390/md20090584)
Supplement: Supplementary file 1 [file marinedrugs-20-00584-s001.zip › marinedrugs-1889253-supplementary.pdf]

SUPPLEMENTARY MATERIAL

Cytotoxic Drimane-type Sesquiterpenes from Co-Culture Marine-Derived Fungi *Aspergillus carneus* KMM 4638 and *Beauveria felina (=Isaria felina)* KMM 4639

Olesya I. Zhuravleva[1,2], Elena B. Belousova[1,2], Galina K. Oleinikova[1], Alexandr S. Antonov[1], Yuliya V. Khudyakova[1], Anton B. Rasin[1], Roman S. Popov[1], Ekaterina S. Menchinskaya[1], Phan Thi Hoai Trinh[3], Anton N. Yurchenko[1,*] and Ekaterina A. Yurchenko[1,*]

1 G.B. Elyakov Pacific Institute of Bioorganic Chemistry, Far Eastern Branch of the Russian Academy of Sciences, Prospect 100-letiya Vladivostoka, 159, Vladivostok 690022, Russia
2 Institute of High Technologies and Advanced Materials, Far Eastern Federal University, 10 Ajax Bay, Russky Island, Vladivostok 690922, Russia
3 Department of marine biotechnology, Nhatrang Institute of Technology Research and Application, Vietnam Academy of Science and Technology, Nha Trang 650000, Vietnam
* Correspondence: yurchenkoan@piboc.dvo.ru (A.N.Y.); eyurch@piboc.dvo.ru (E.A.Y.); Tel.: +7-423-231-1168 (A.N.Y. & E.A.Y.)

Abstract: Chemical investigation of co-culture marine-derived fungi *Beauveria felina* KMM 4639 and *Aspergillus carneus* KMM 4638 led to the identification of three new drimane-type sesquiterpenes asperflavinoids B, D and E (**2**, **4**, **5**) and nine previously reported related compounds. Their structures were established using spectroscopic methods and by comparison with known analogues. Cytotoxic activity of isolated compounds against several cancer and normal cell lines were investigated. Compounds **3** and ustusolate E (**9**) shown significant effect on human breast cancer MCF-7 cell viability with IC_{50}s of 10 μM and induced in these cells the caspase-dependent apoptosis and arrest of MCF-7 cell cycle in G2/M phase.

Keywords: marine-derived fungi; *Aspergillus carneus*, *Beauveria felina (Isaria felina)*, co-culture; secondary metabolites; drimane sesquiterpenes; cytotoxicity

Content

Figure S1. CD spectrum of **1** 4
Figure S2. CD spectrum of **3** 4
Figure S3. CD spectrum of **4** 5
Figure S4. CD spectrum of **5** 5
Figure S5. CD spectrum of **10** 6
Figure S6. CD spectrum of **11** 6
Figure S7. CD spectrum of **12** 7
Figure S8. UV spectrum of **1** 7
Figure S9. UV spectrum of **3** 8
Figure S10. UV spectrum of **4** 8
Figure S11. UV spectrum of **5** 9
Figure S12. UV spectrum of **10** 9
Figure S13. UV spectrum of **11** 10
Figure S14. UV spectrum of **12** 10
Figure S15. ^{1}H NMR spectrum (700 MHz, acetone-d_6) of **1** 12
Figure S16. ^{13}C NMR spectrum (175 MHz, acetone-d_6) of **1** 17
Figure S17. COSY-45 spectrum (700 MHz, acetone-d_6) of **1** 18
Figure S18. HSQC spectrum (700 MHz, acetone-d_6) of **1** 19
Figure S19. HMBC spectrum (700 MHz, acetone-d_6) of **1** 20
Figure S20. ROESY spectrum (700 MHz, acetone-d_6) of **1** 21
Figure S21. Expanded ^{1}H NMR spectrum (700 MHz, DMSO-d_6) of **1** 22
Figure S22. ^{1}H NMR spectrum (700 MHz, acetone-d_6) of **2** 31
Figure S23. ^{13}C NMR spectrum (175 MHz, acetone-d_6) of **2** 37
Figure S24. COSY-45 spectrum (700 MHz, acetone-d_6) of **2** 38
Figure S25. HSQC spectrum (700 MHz, acetone-d_6) of **2** 39
Figure S26. HMBC spectrum (700 MHz, acetone-d_6) of **2** 40
Figure S27. ROESY spectrum (700 MHz, acetone-d_6) of **2** 41
Figure S28. ^{1}H NMR spectrum (300 MHz, $CDCl_3$) of **3** 42
Figure S29. ^{13}C NMR spectrum (75 MHz, $CDCl_3$) of **3** 47
Figure S30. DEPT-135 NMR spectrum (75 MHz, $CDCl_3$) of **3** 48
Figure S31. COSY-45 spectrum (300 MHz, $CDCl_3$) of **3** 49

Figure S32. HSQC spectrum (300 MHz, $CDCl_3$) of **3** 50
Figure S33. HMBC spectrum (300 MHz, $CDCl_3$) of **3** 51
Figure S34. ROESY spectrum (300 MHz, $CDCl_3$) of **3** 52
Figure S35. ^{1}H NMR spectrum (700 MHz, $CDCl_3$) of **4** 53
Figure S36. ^{13}C NMR spectrum (75 MHz, $CDCl_3$) of **4** 58
Figure S37. DEPT-135 NMR spectrum (75 MHz, $CDCl_3$) of **4** 59
Figure S38. COSY-45 spectrum (700 MHz, $CDCl_3$) of **4** 60
Figure S39. HSQC spectrum (700 MHz, $CDCl_3$) of **4** 61
Figure S40. HMBC spectrum (700 MHz, $CDCl_3$) of **4** 62
Figure S41. ^{1}H NMR spectrum (700 MHz, $CDCl_3$) of **5** 63
Figure S42. ^{13}C NMR spectrum (75 MHz, $CDCl_3$) of **5** 68
Figure S43. DEPT-135 NMR spectrum (75 MHz, $CDCl_3$) of **5** 69
Figure S44. COSY-45 spectrum (700 MHz, $CDCl_3$) of **5** 70
Figure S45. HSQC spectrum (700 MHz, $CDCl_3$) of **5** 71
Figure S46. HMBC spectrum (700 MHz, $CDCl_3$) of **5** 72
Figure S47. ROESY spectrum (700 MHz, $CDCl_3$) of **5** 73
Figure S48. Expanded ^{1}H NMR spectrum (700 MHz, $CDCl_3$) of **9** 74
Figure S49. Expanded ^{1}H NMR spectrum (700 MHz, $CDCl_3$) of **10** 79
Figure S50. Expanded ^{1}H NMR spectrum (700 MHz, DMSO-d_6) of **11** 83
Figure S51. Expanded ^{1}H NMR spectrum (700 MHz, DMSO-d_6) of **12** 88
Figure S52. Expanded ^{1}H NMR spectrum (700 MHz, $CDCl_3$) of **12** 93
Figure S53. ^{1}H NMR spectrum (700 MHz, acetone-d_6) of **1a** 99
Figure S54. ^{1}H NMR spectrum (700 MHz, acetone-d_6) of **2a** 100

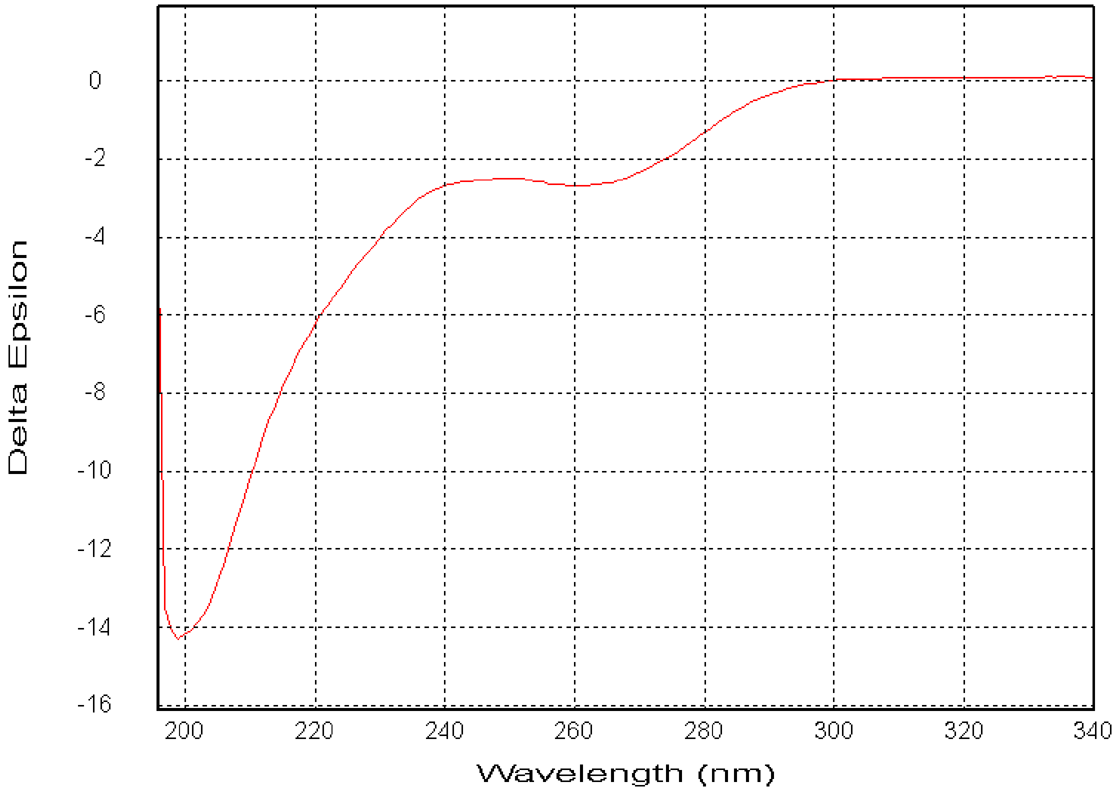

Figure S1. CD spectrum of **1**

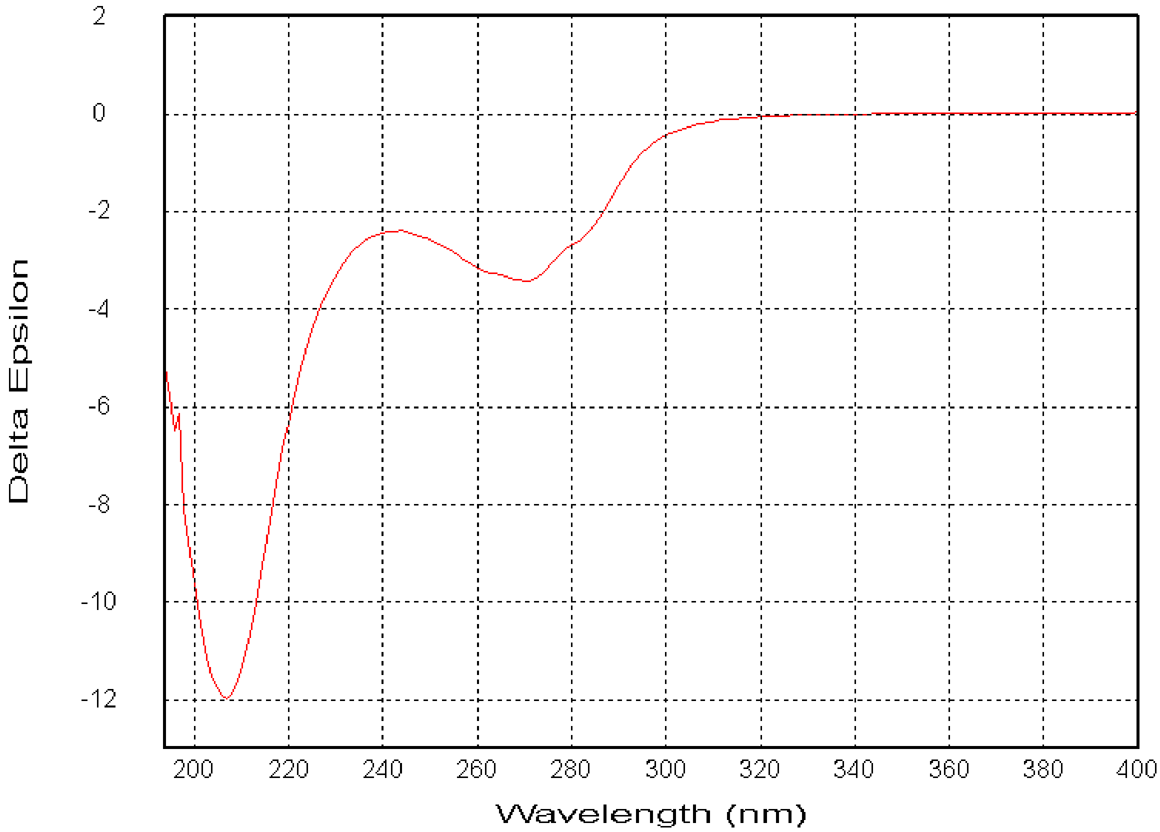

Figure S2. CD spectrum of **3**

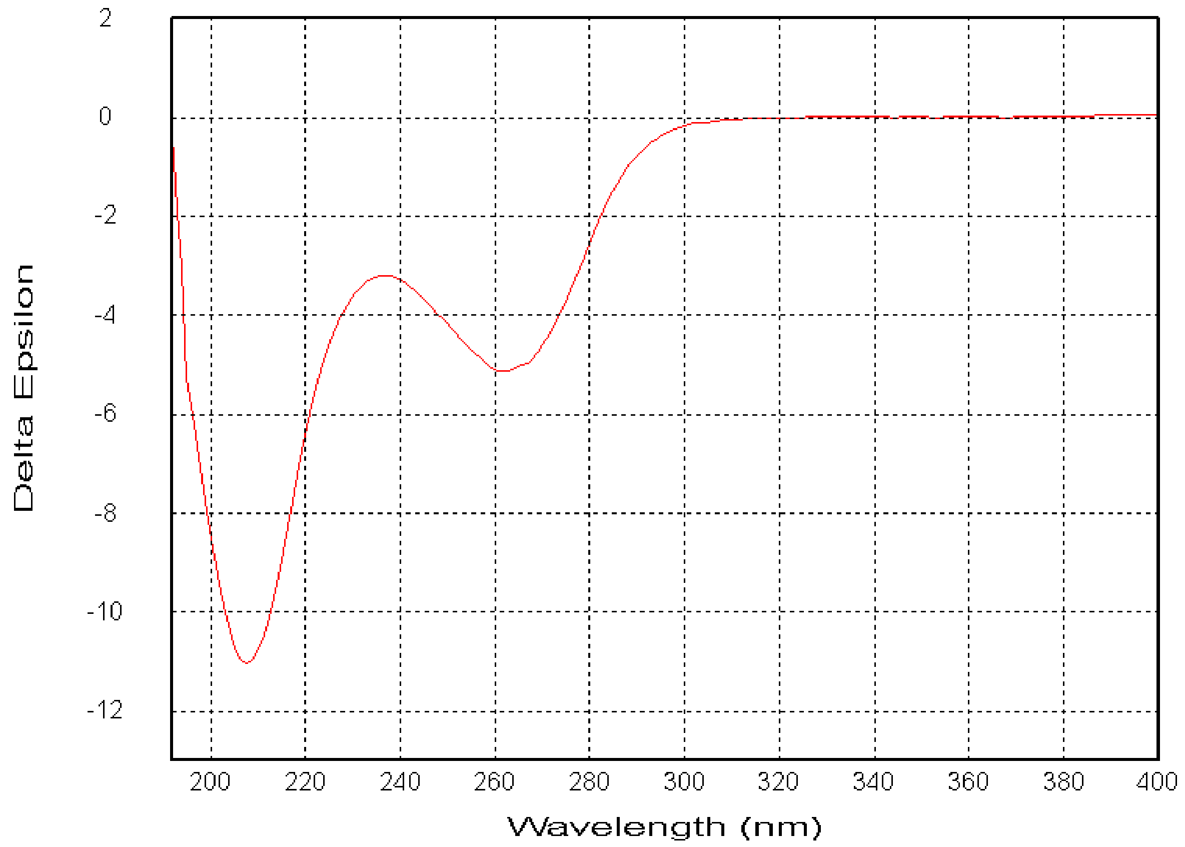

Figure S3. CD spectrum of **4**

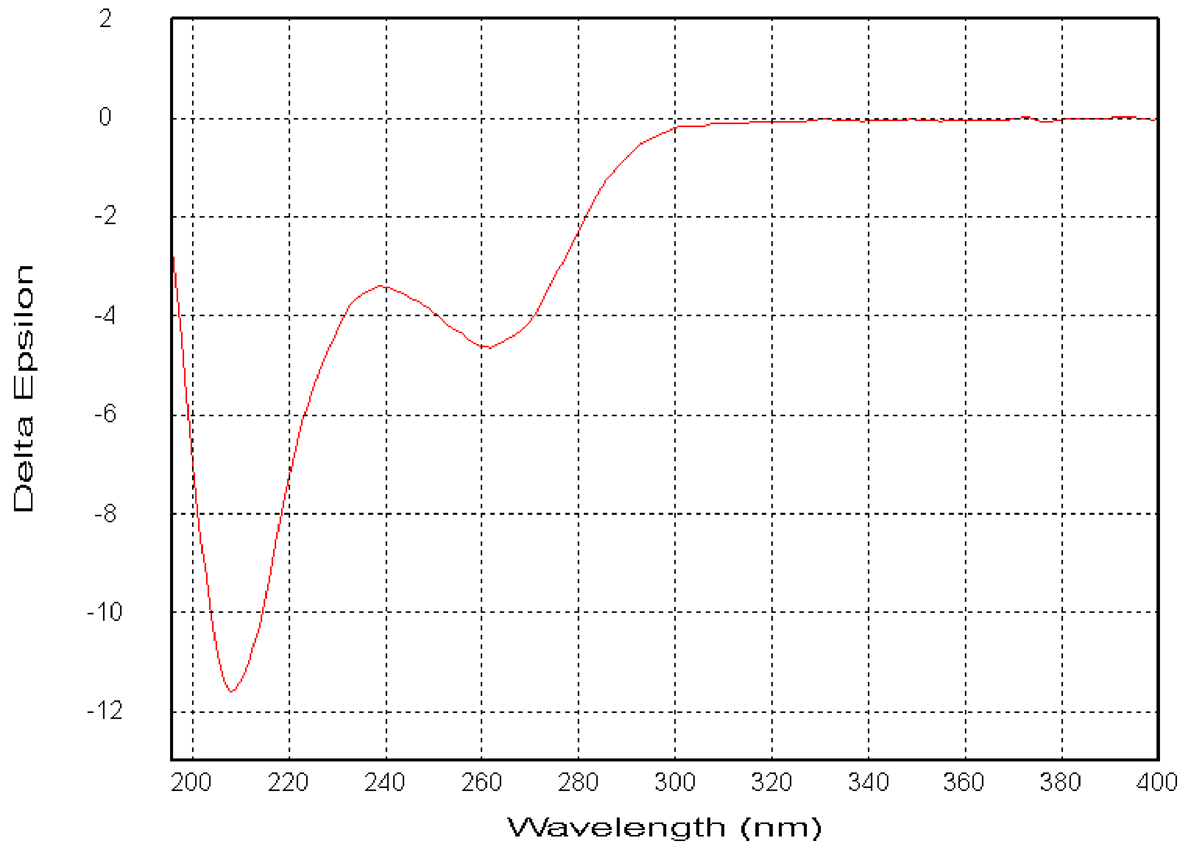

Figure S4. CD spectrum of **5**

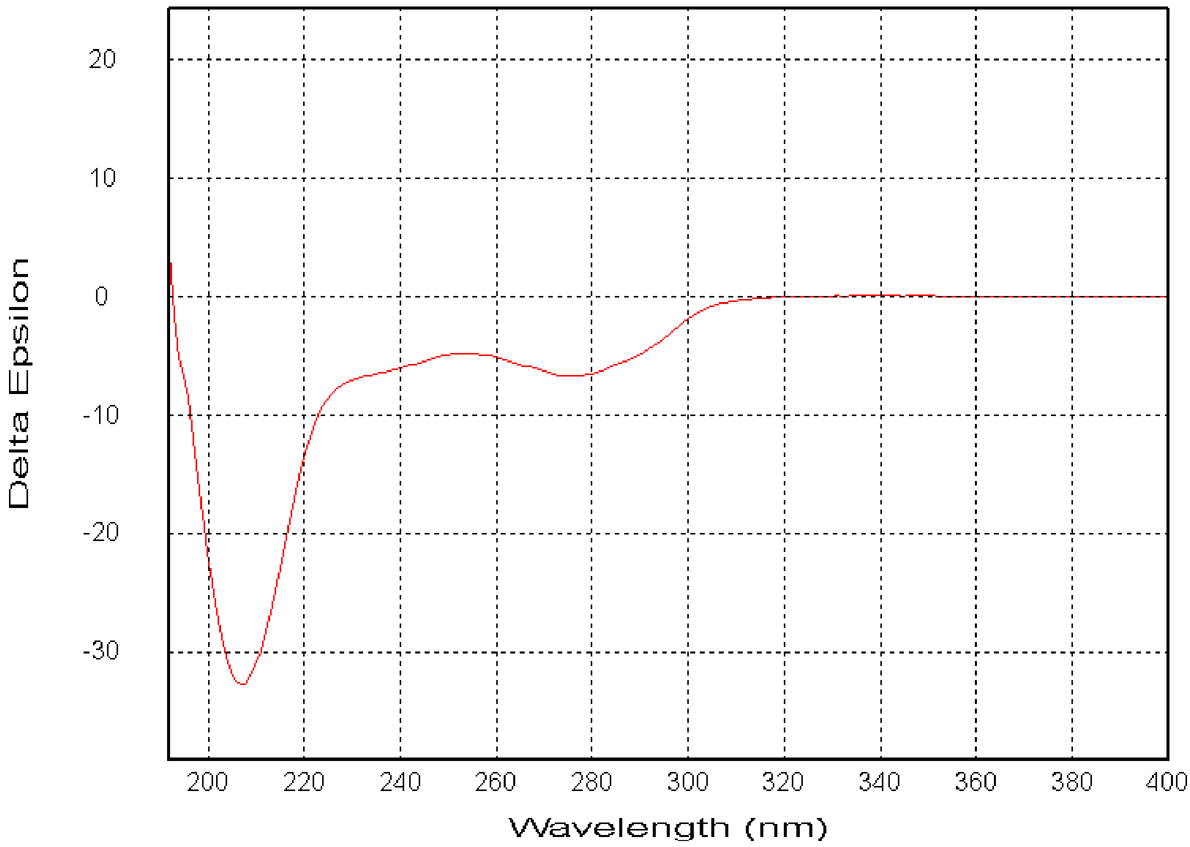

Figure S5. CD spectrum of **10**

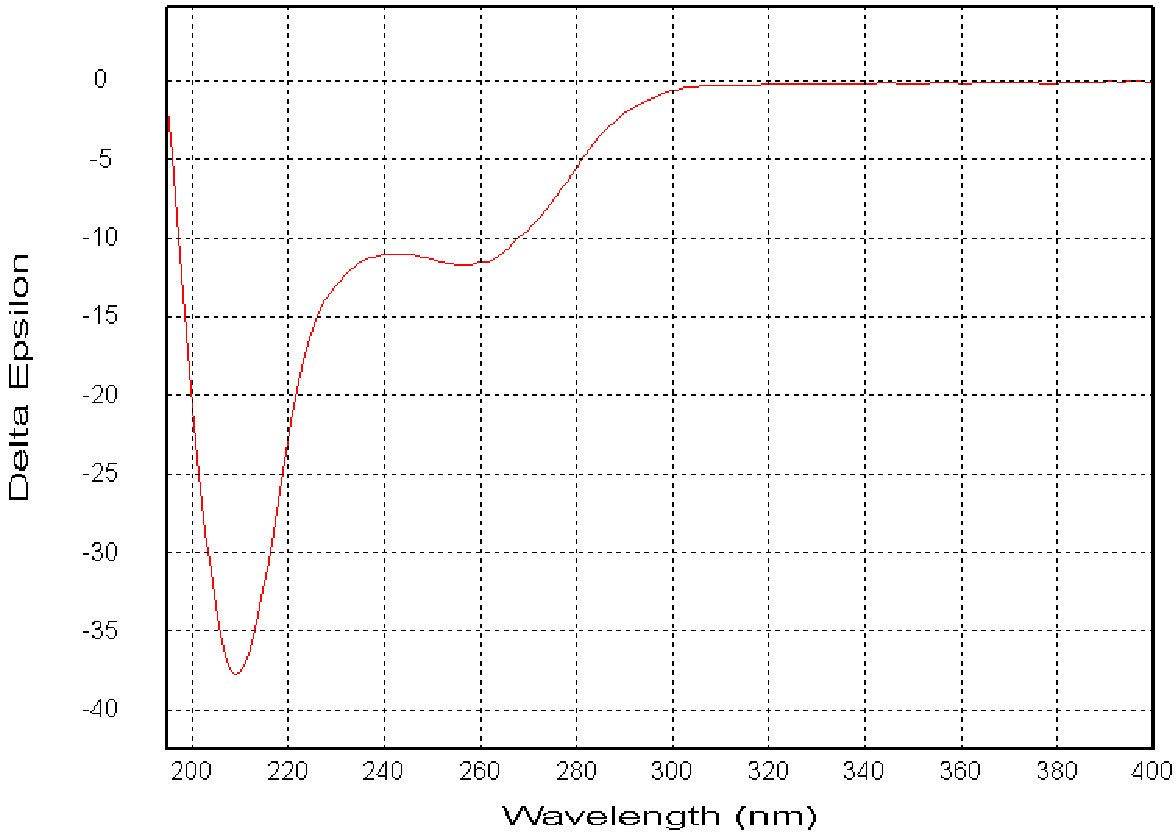

Figure S6. CD spectrum of **11**

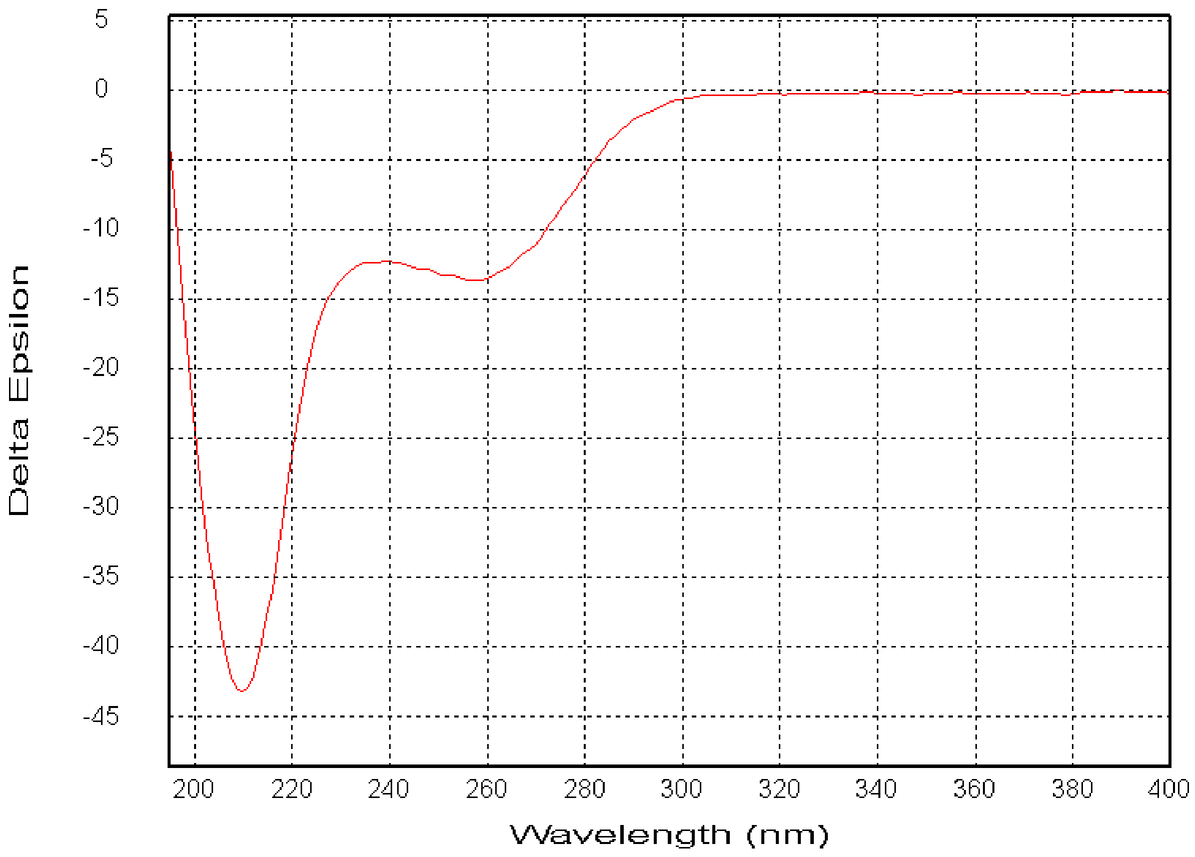

Figure S7. CD spectrum of **12**

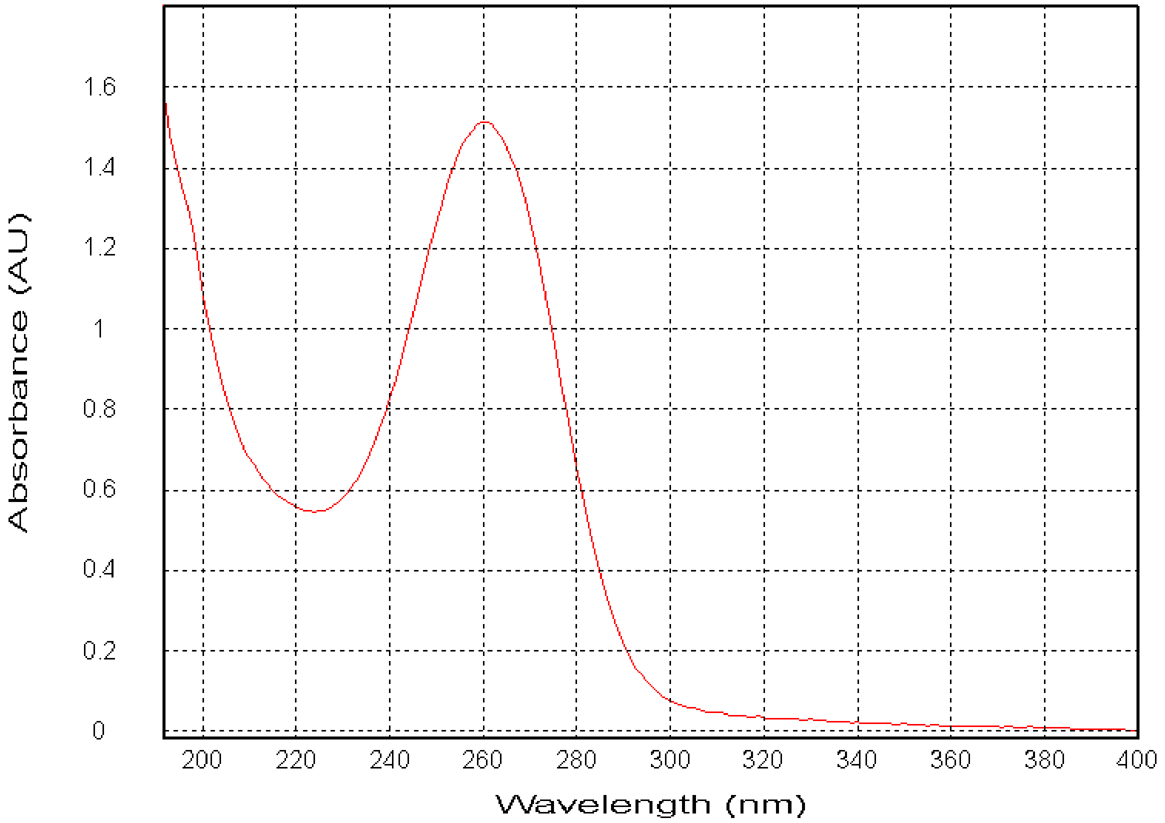

Figure S8. UV spectrum of **1**

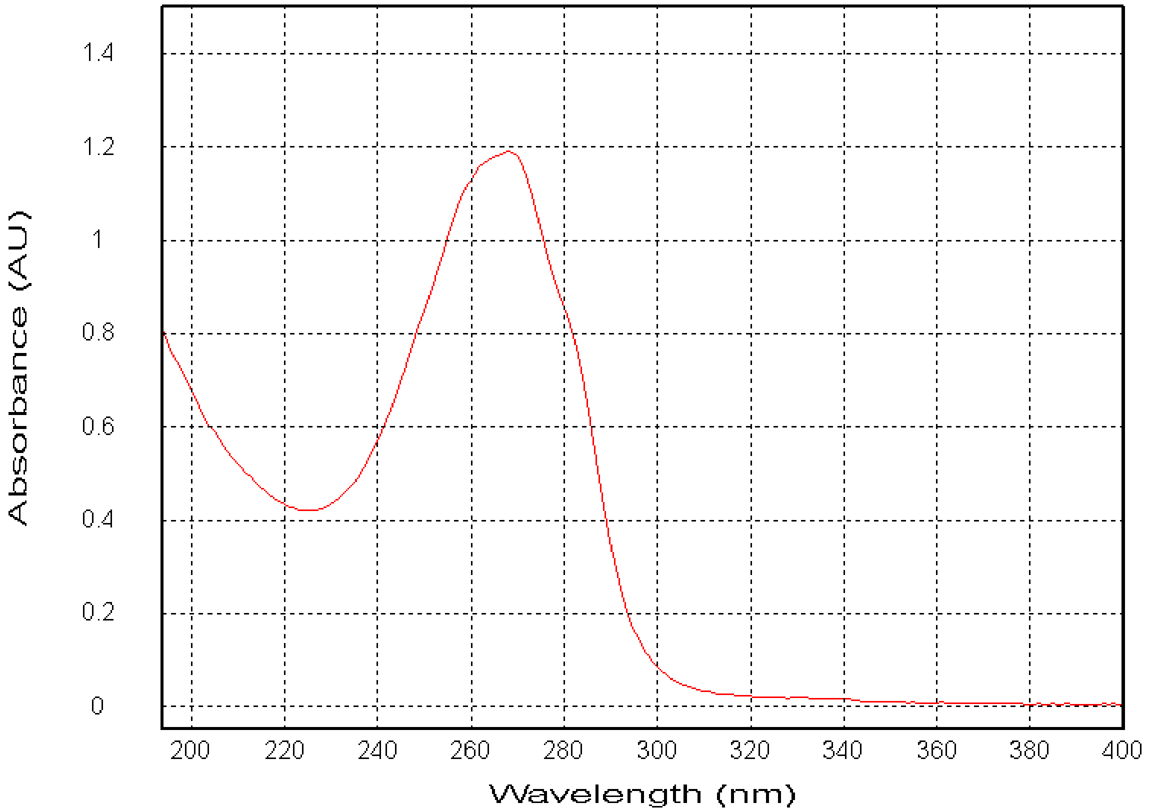

Figure S9. UV spectrum of **3**

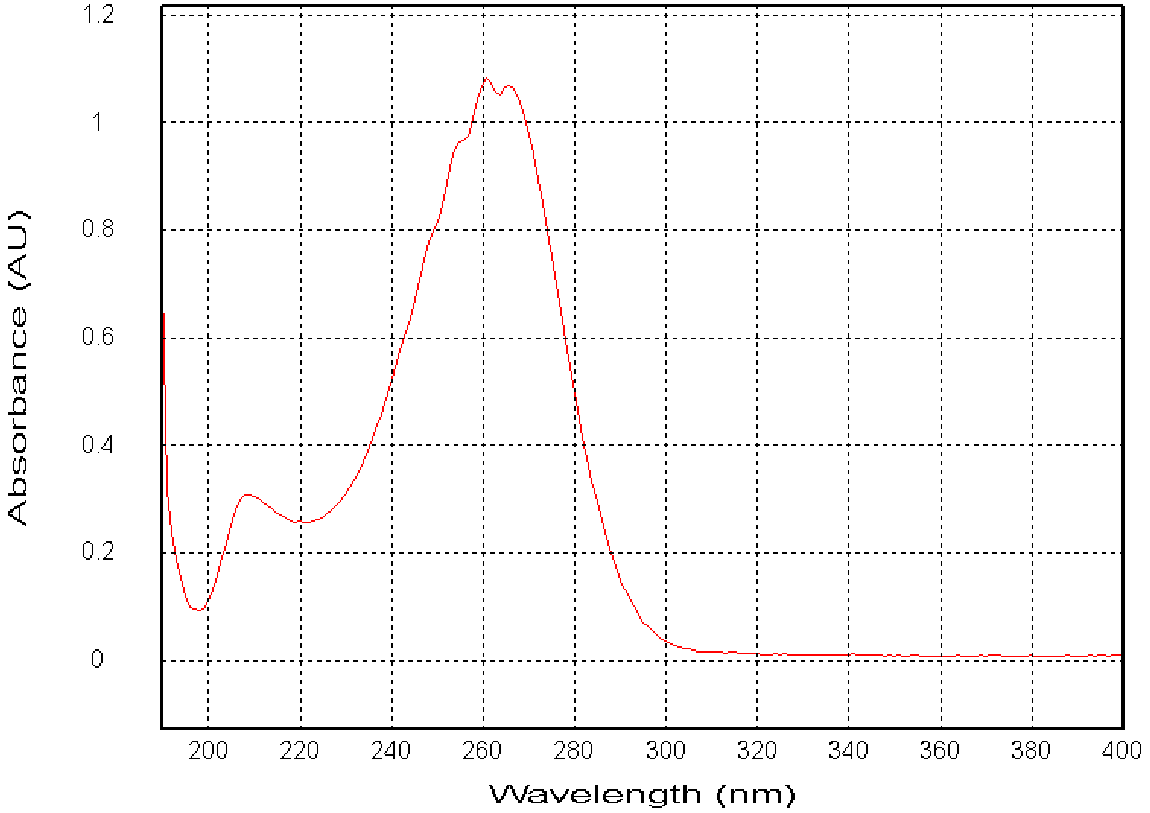

Figure S10. UV spectrum of **4**

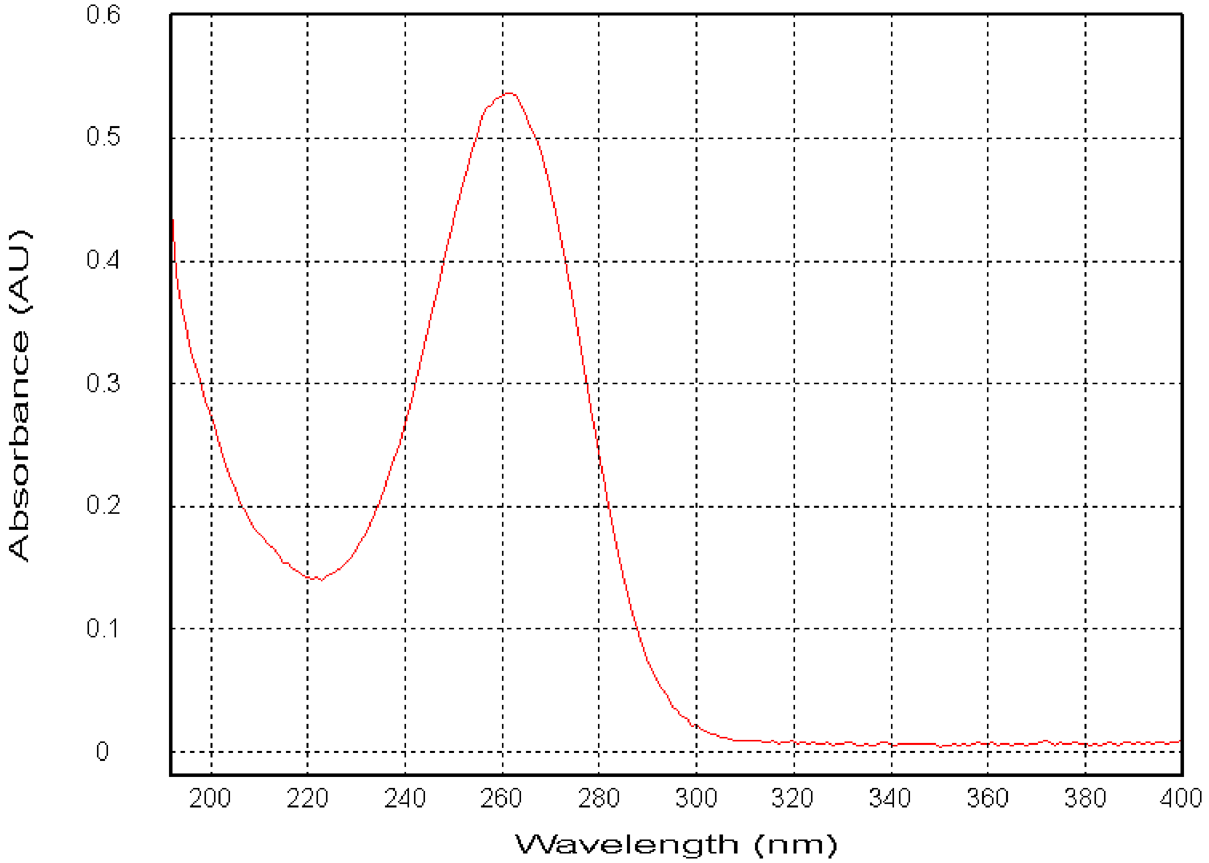

Figure S11. UV spectrum of **5**

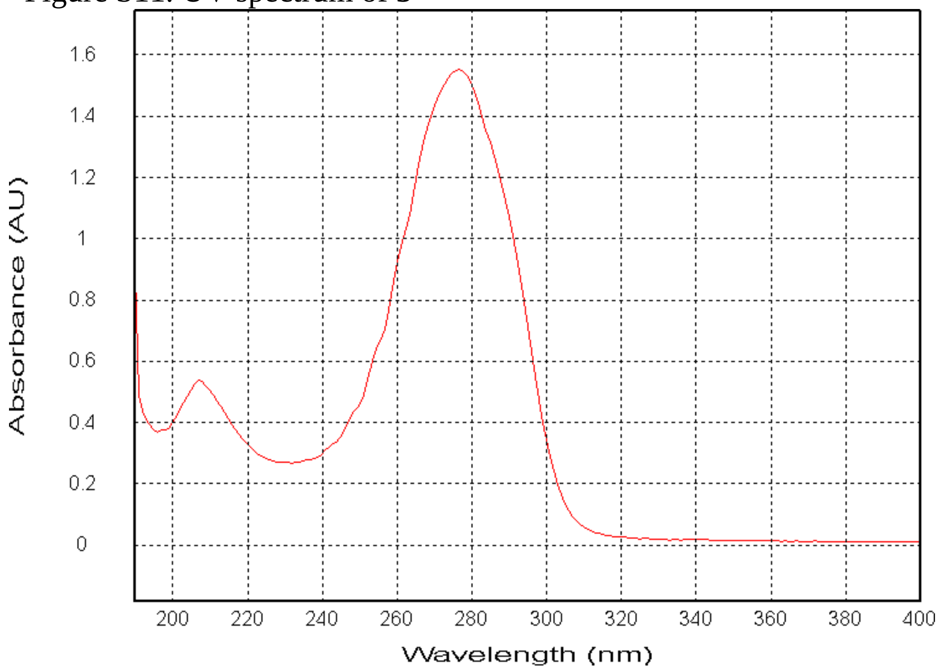

Figure S12. UV spectrum of **10**

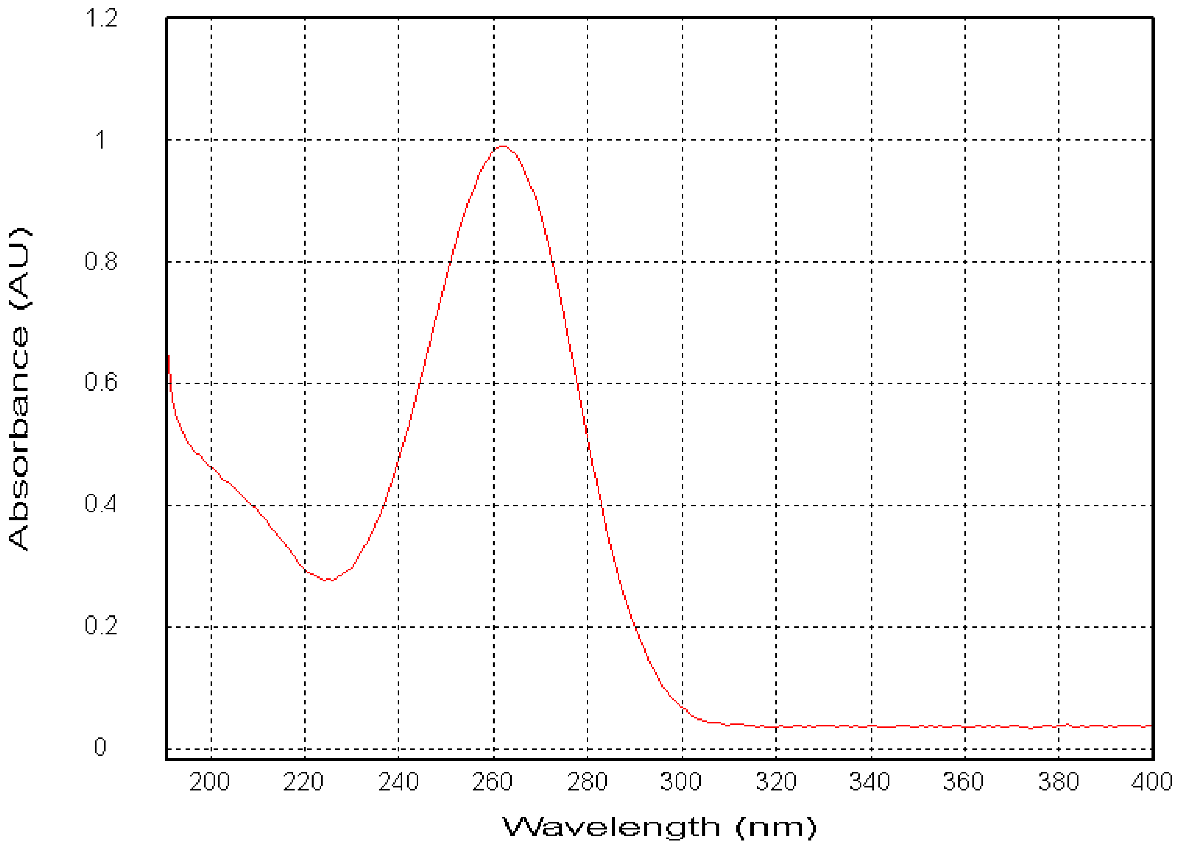

Figure S13. UV spectrum of **11**

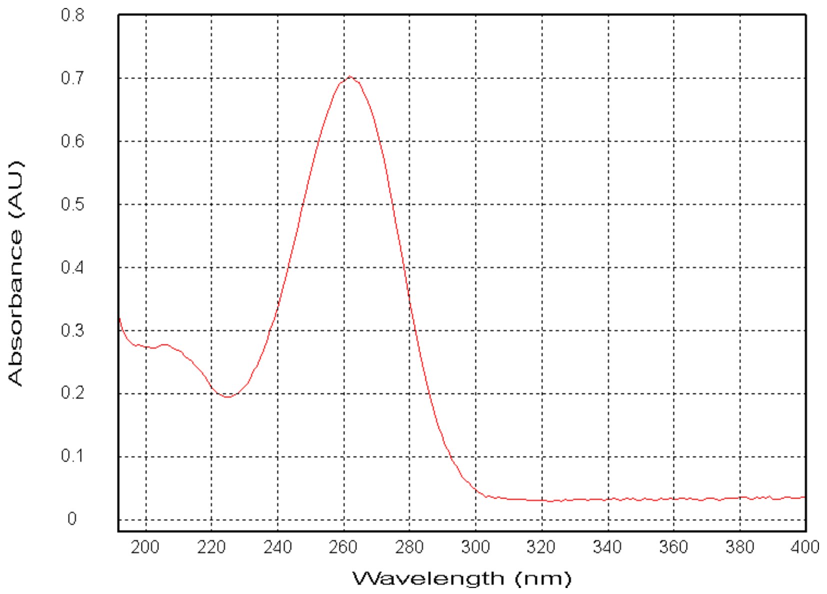

Figure S14. UV spectrum of **12**

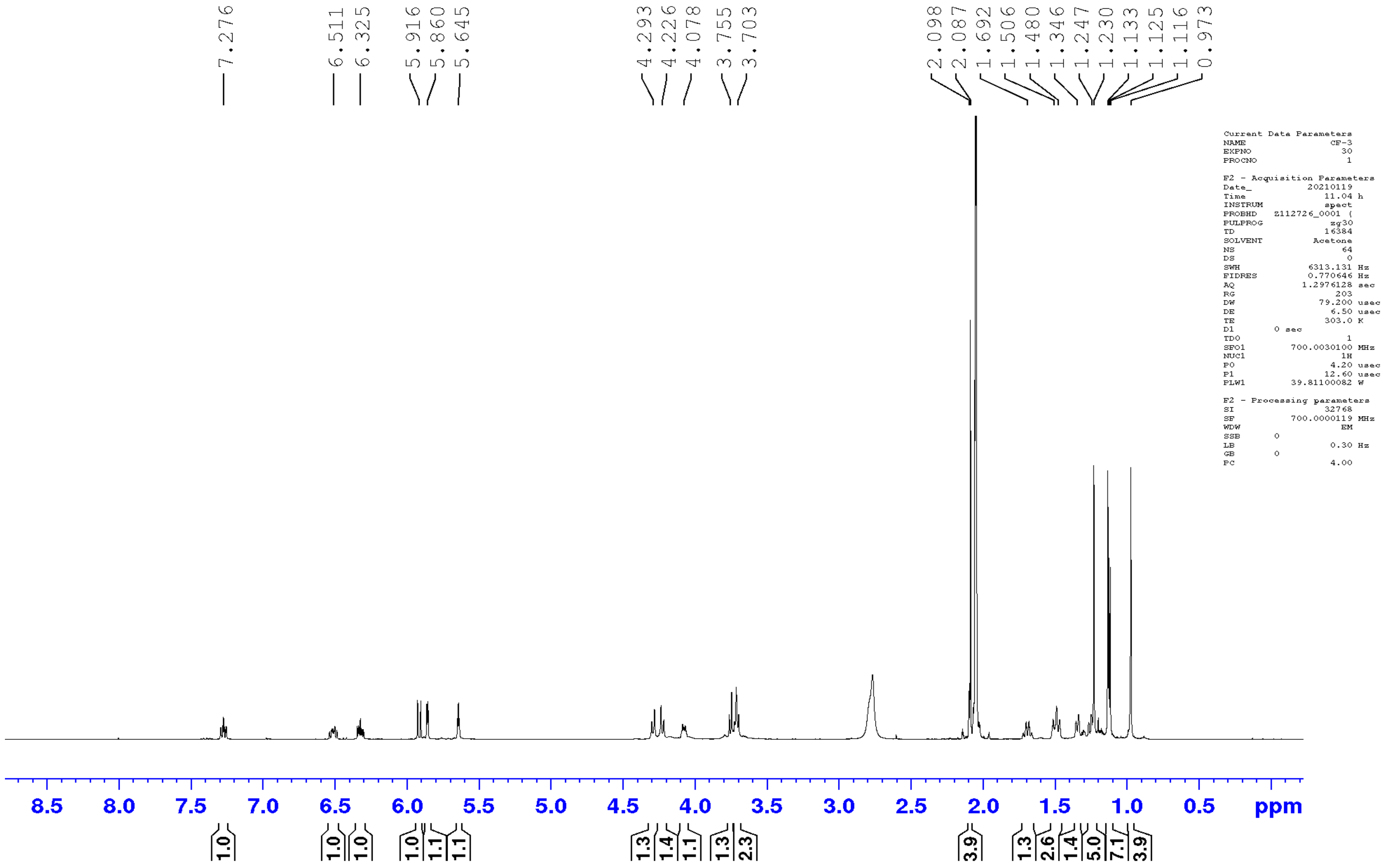

Figure S15. ^{1}H NMR spectrum (700 MHz, acetone-d_6) of **1**

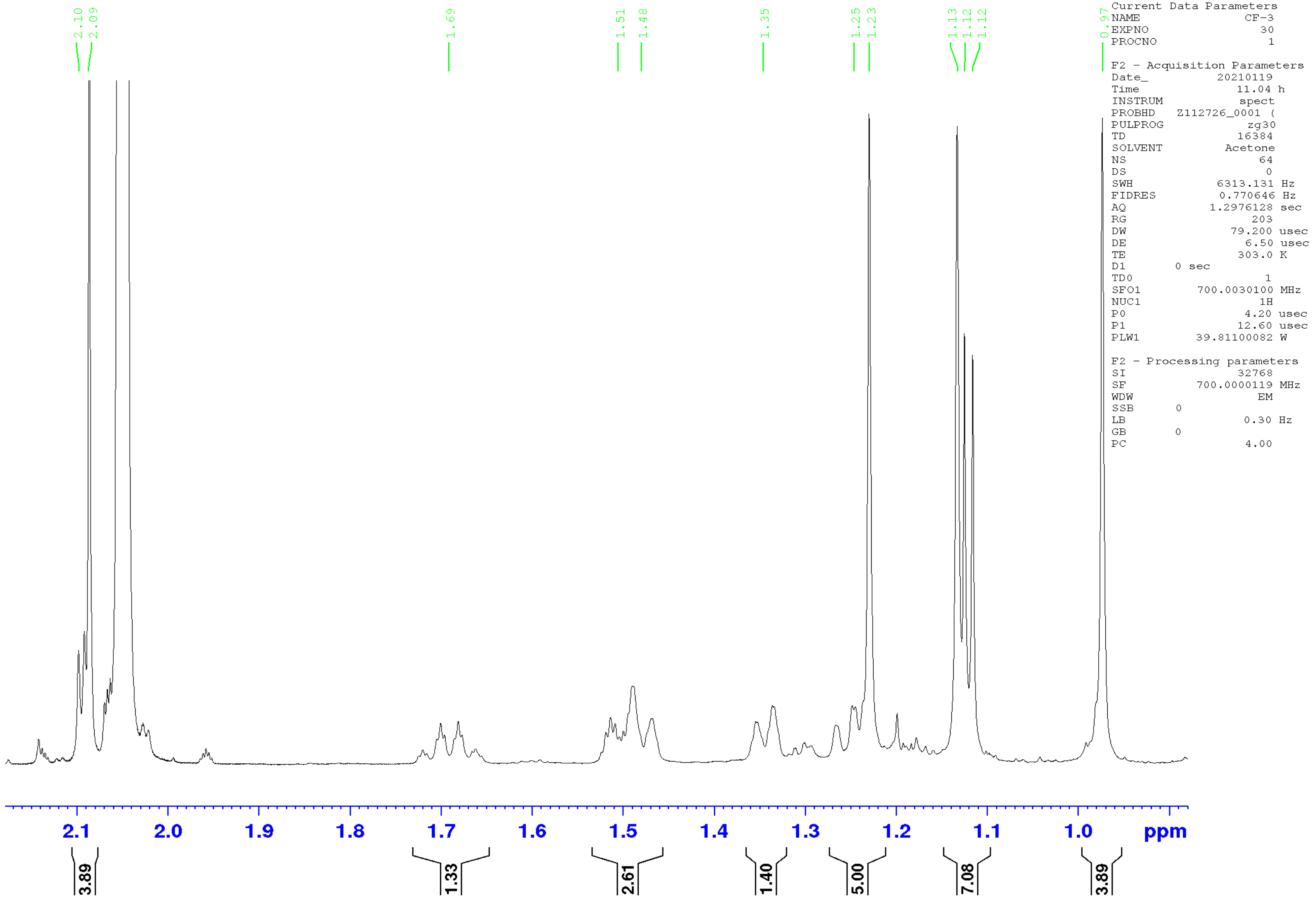

^{1}H NMR spectrum (700 MHz, acetone-d$_6$) of **1**

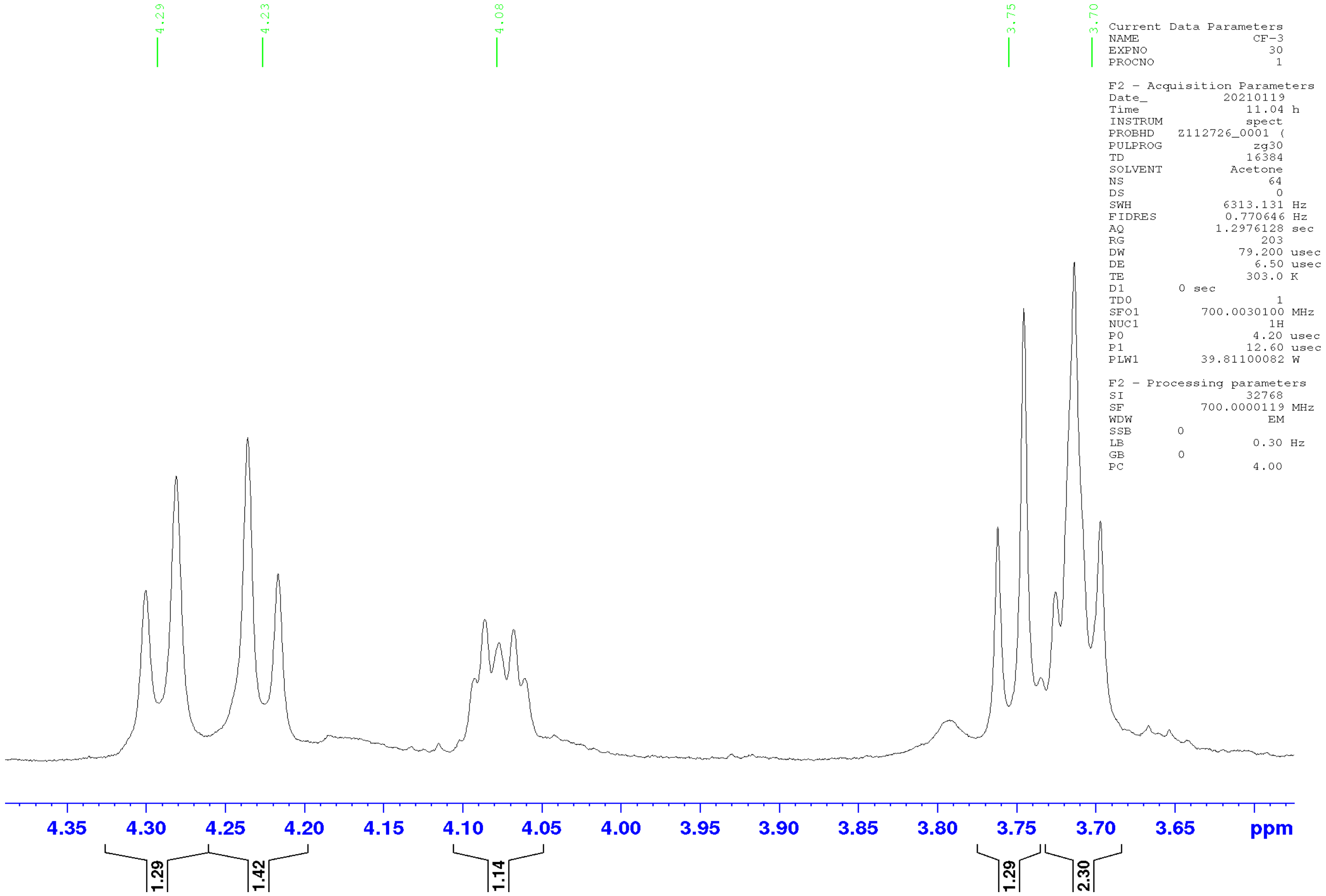

^{1}H NMR spectrum (700 MHz, acetone-d_6) of **1**

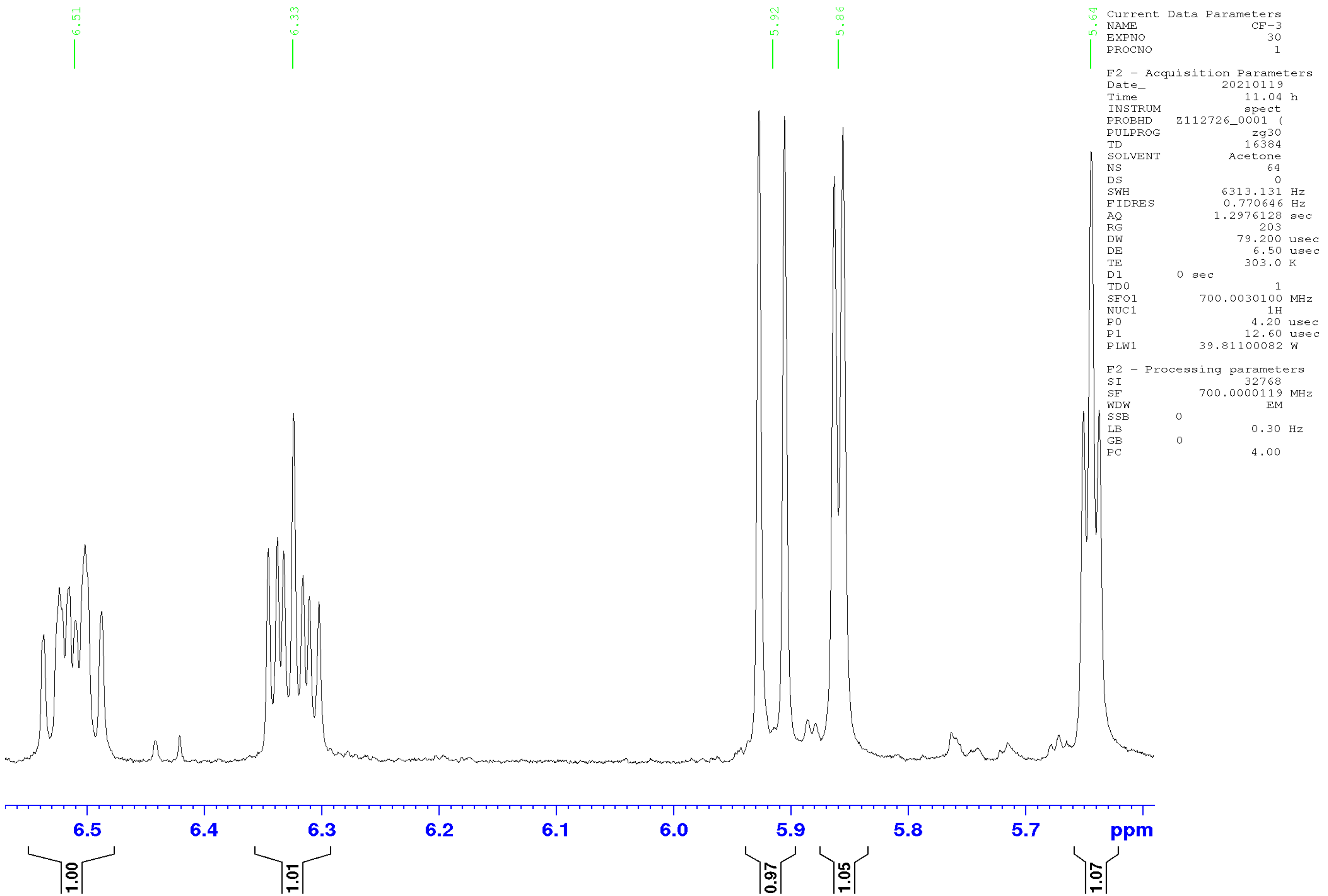

^{1}H NMR spectrum (700 MHz, acetone-d_6) of **1**

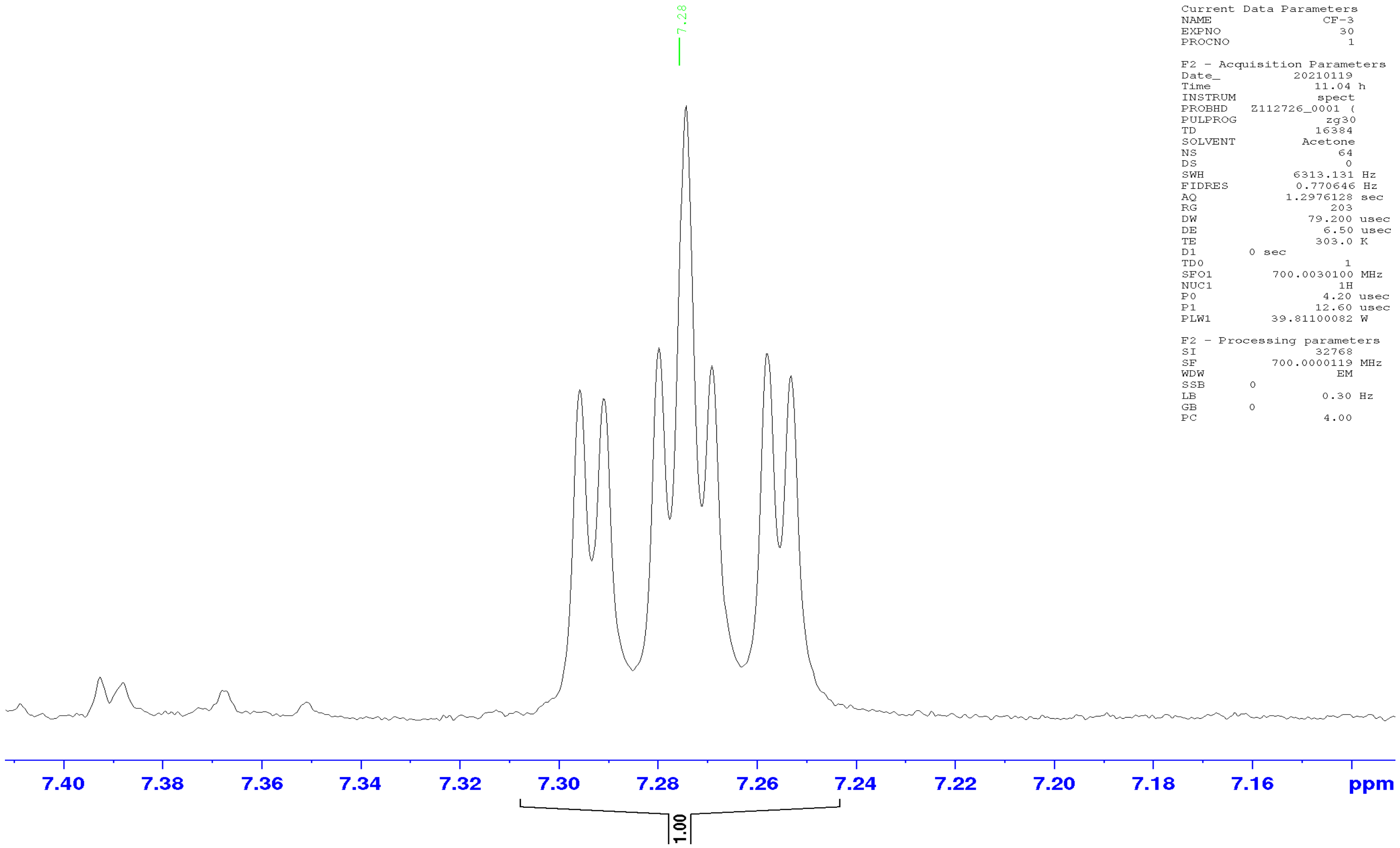

^{1}H NMR spectrum (700 MHz, acetone-d_6) of **1**

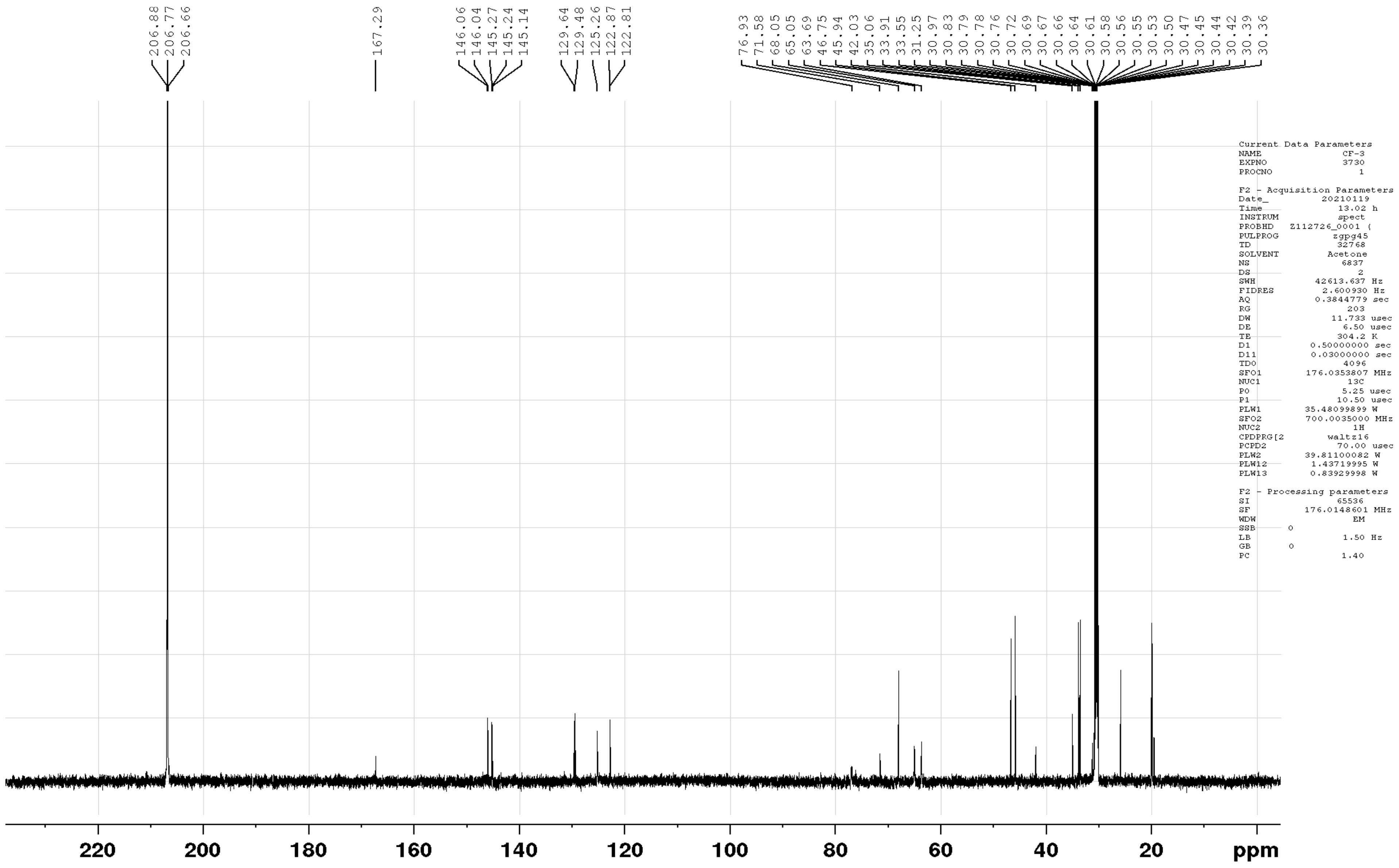

Figure S16. ^{13}C NMR spectrum (175 MHz, acetone-d_6) of **1**

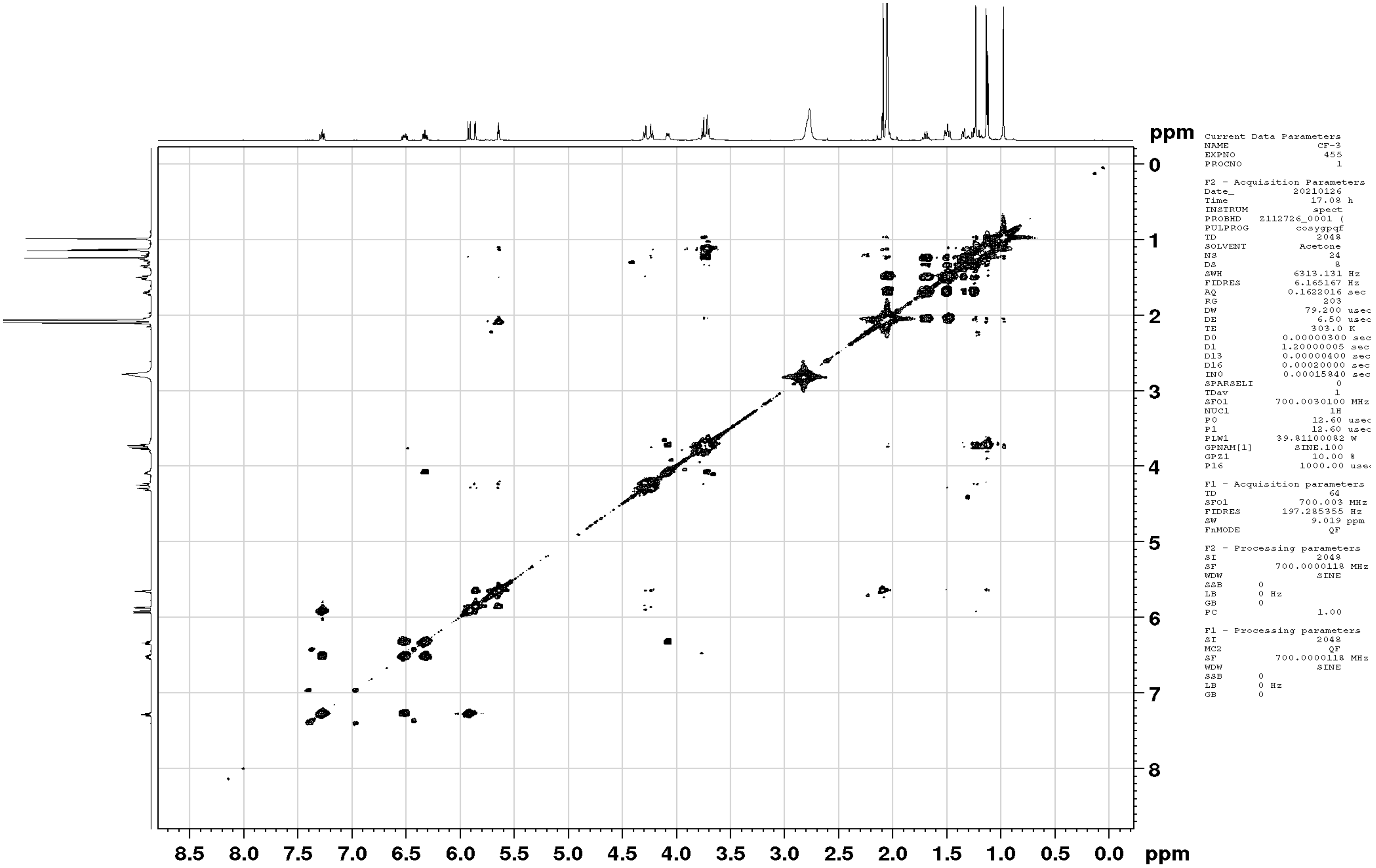

Figure S17. COSY-45 spectrum (700 MHz, acetone-d_6) of **1**

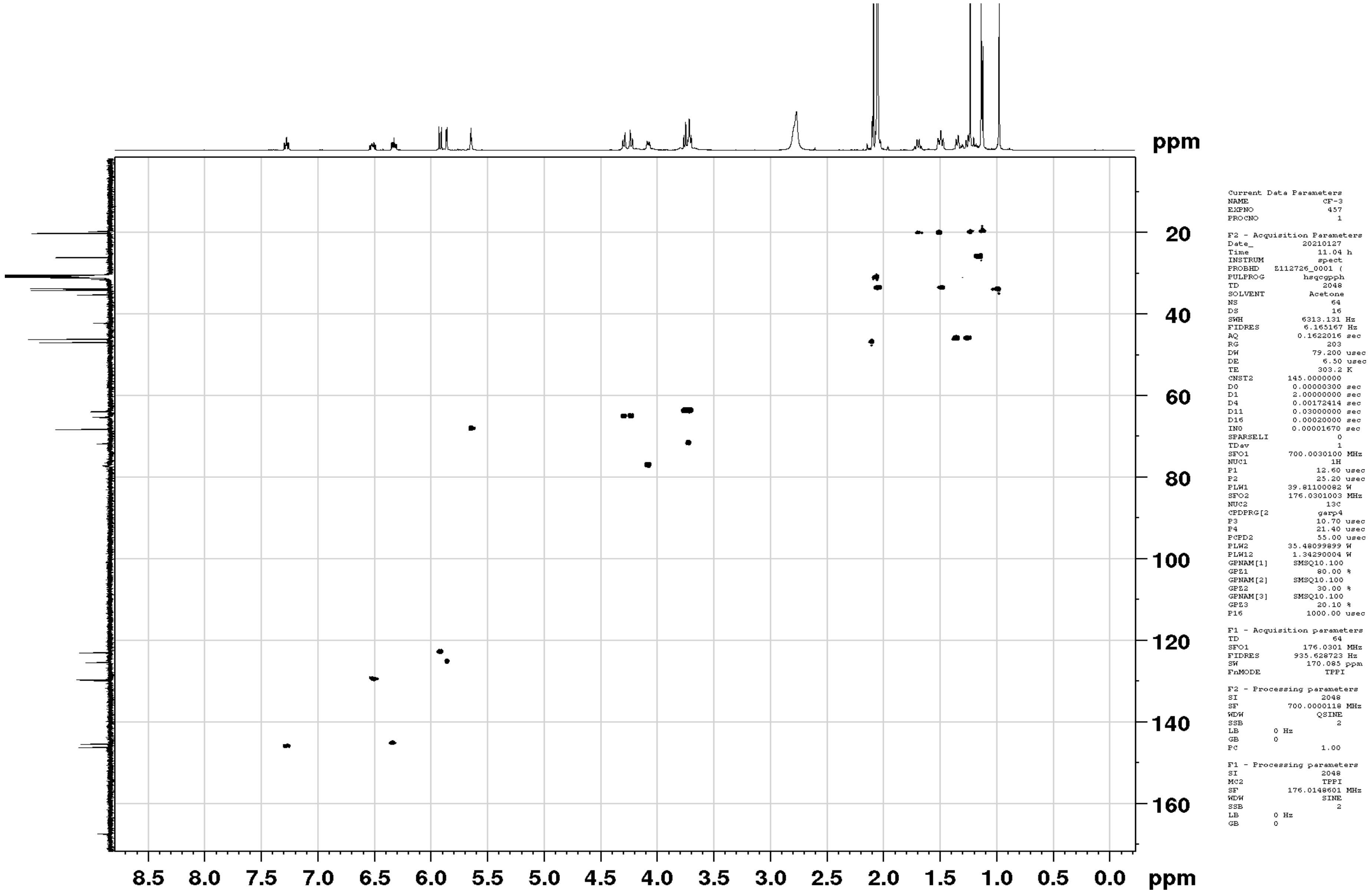

Figure S18. HSQC spectrum (700 MHz, acetone-d_6) of **1**

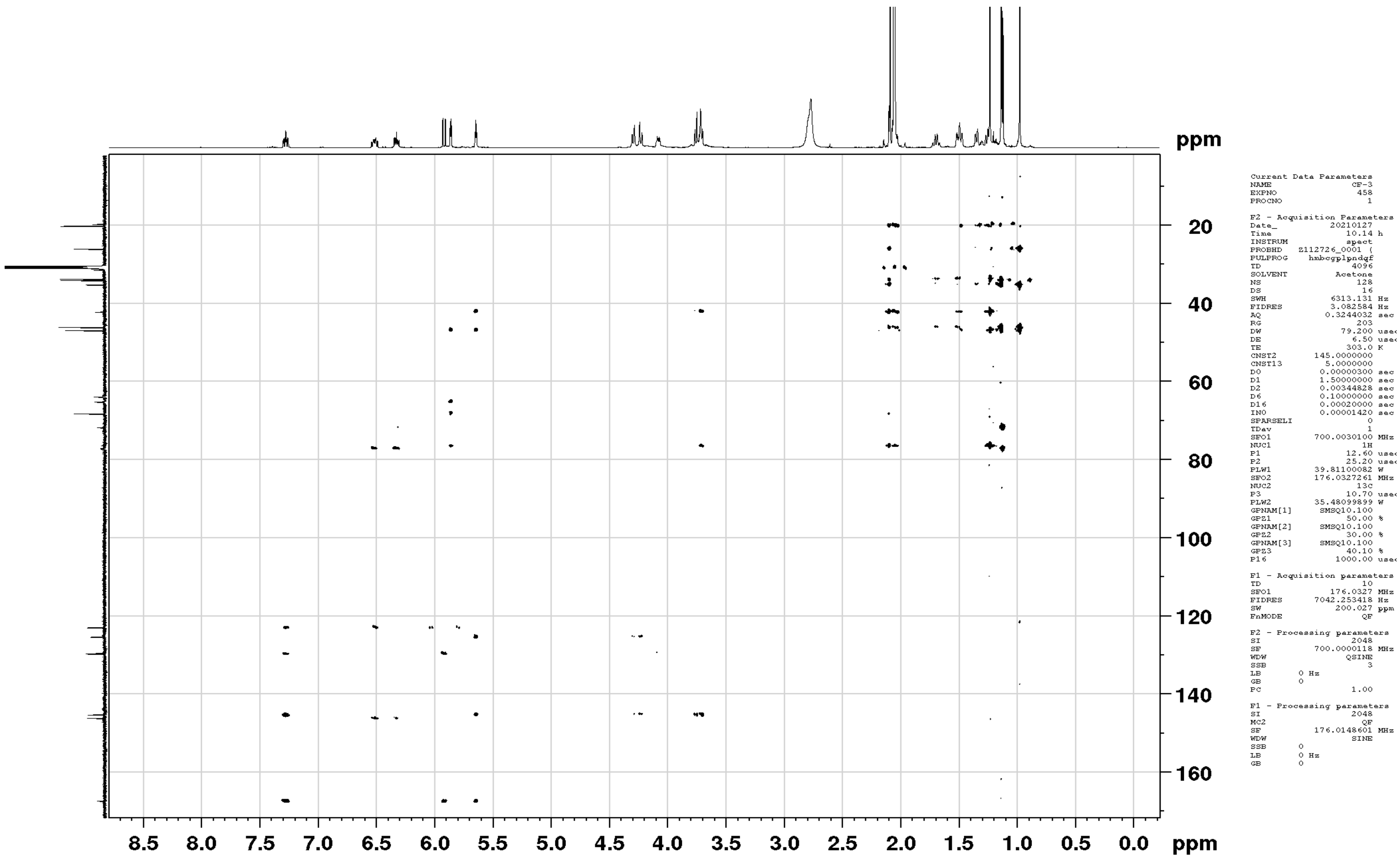

Figure S19. HMBC spectrum (700 MHz, acetone-d_6) of **1**

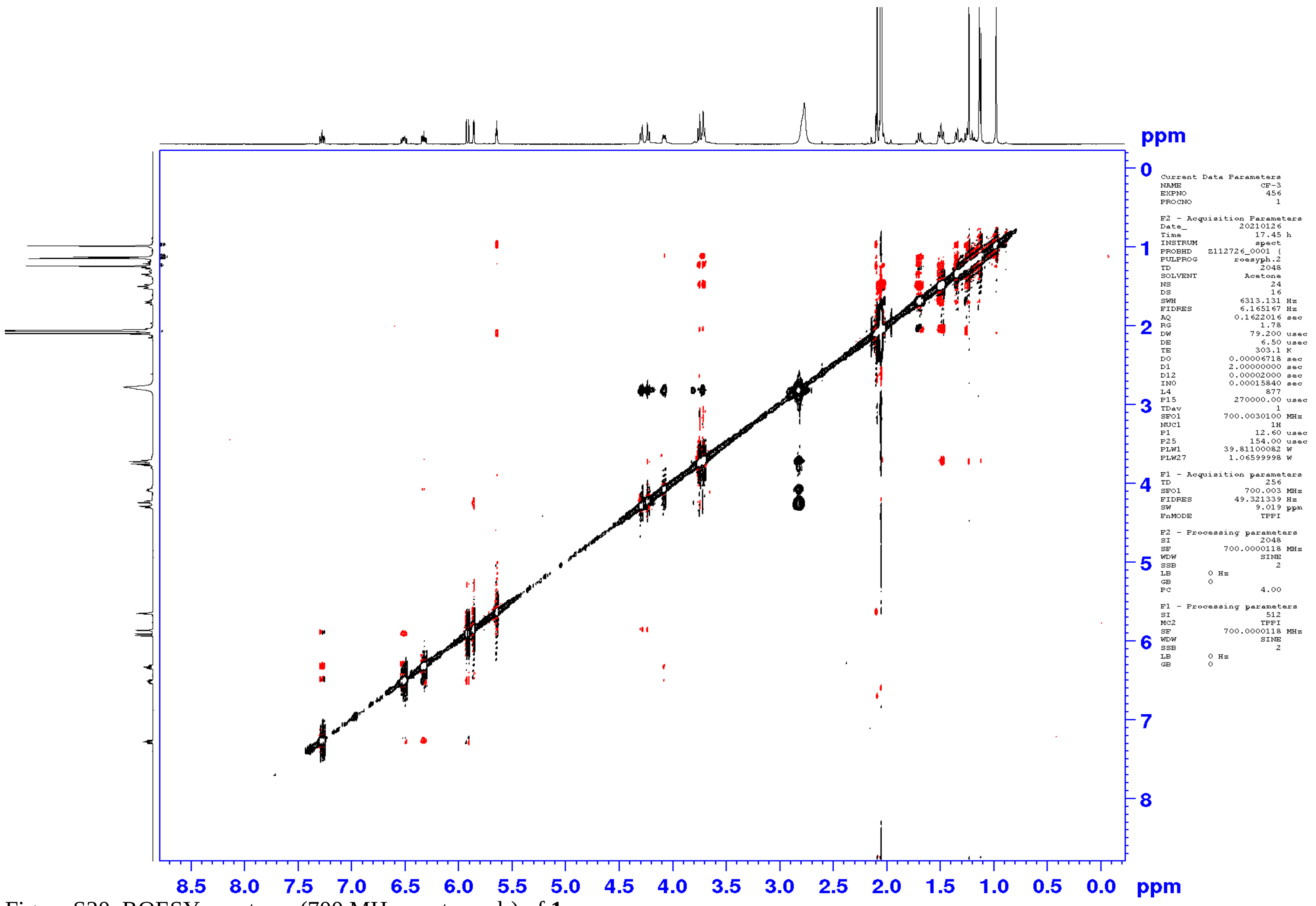

Figure S20. ROESY spectrum (700 MHz, acetone-d_6) of **1**

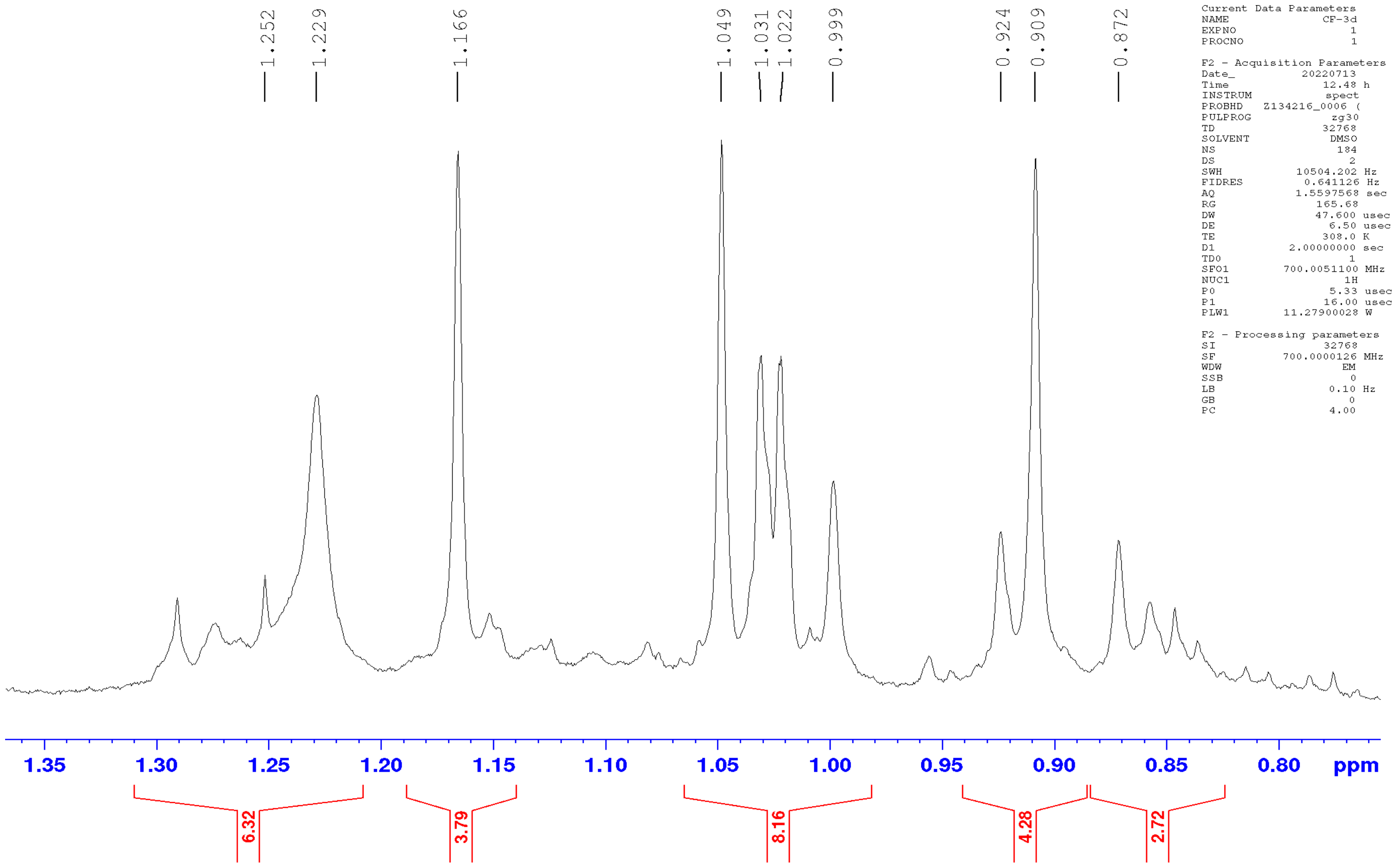

Figure S**21**. Expanded ^{1}H NMR spectrum (700 MHz, DMSO-d_6) of **1**

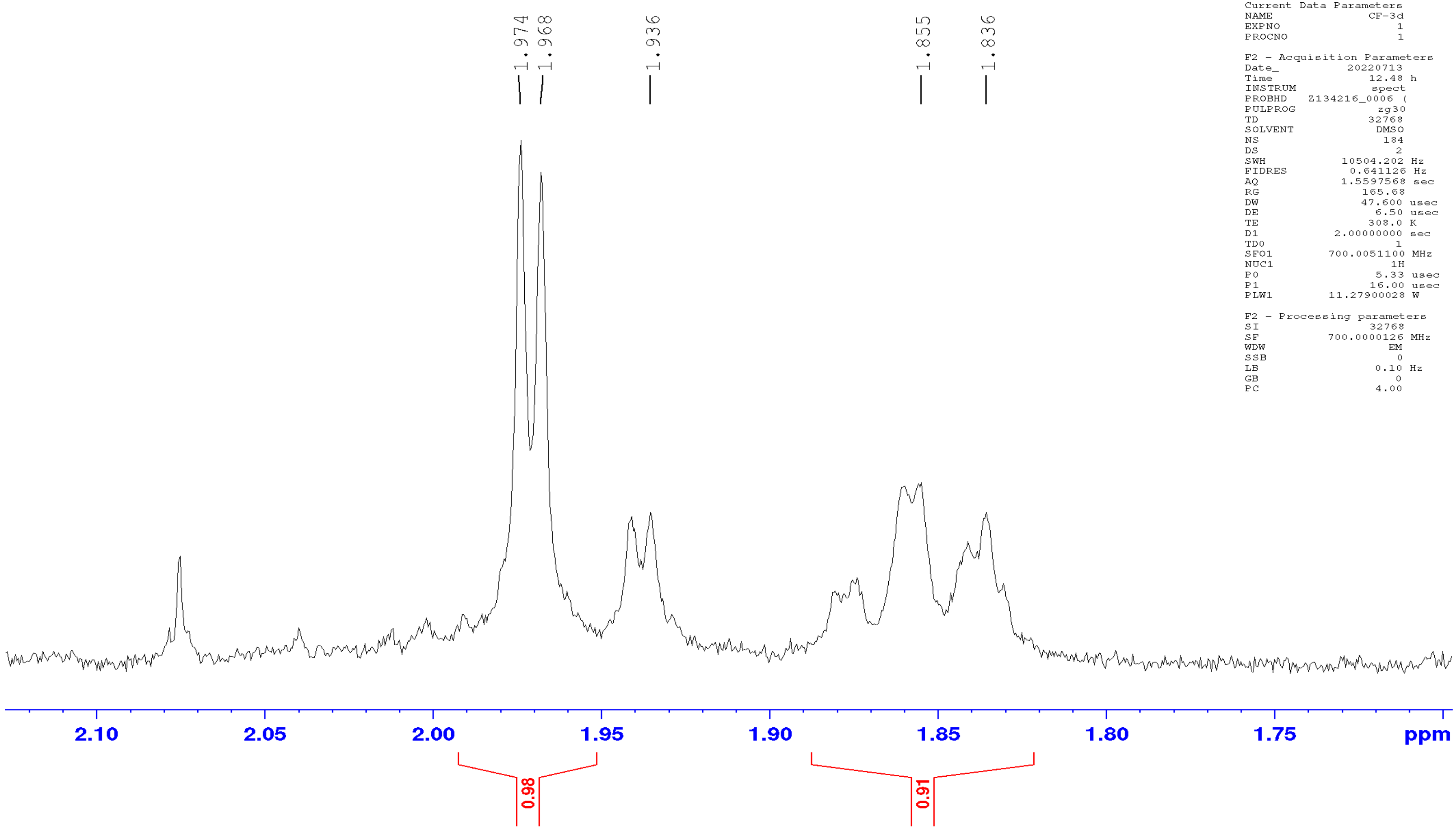

Expanded ^{1}H NMR spectrum (700 MHz, DMSO-d_6) of **1**

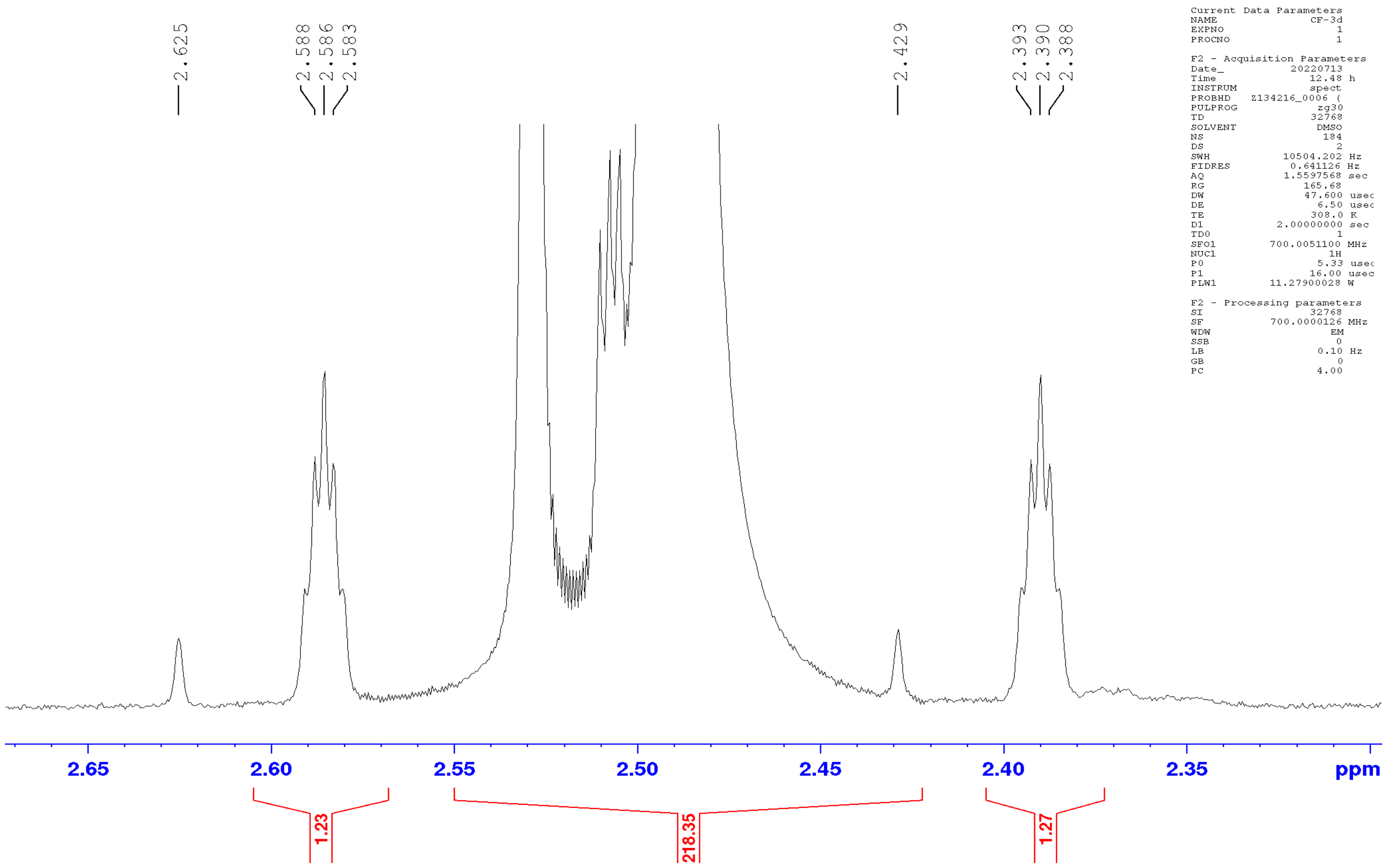

Expanded ^{1}H NMR spectrum (700 MHz, DMSO-d_6) of **1**

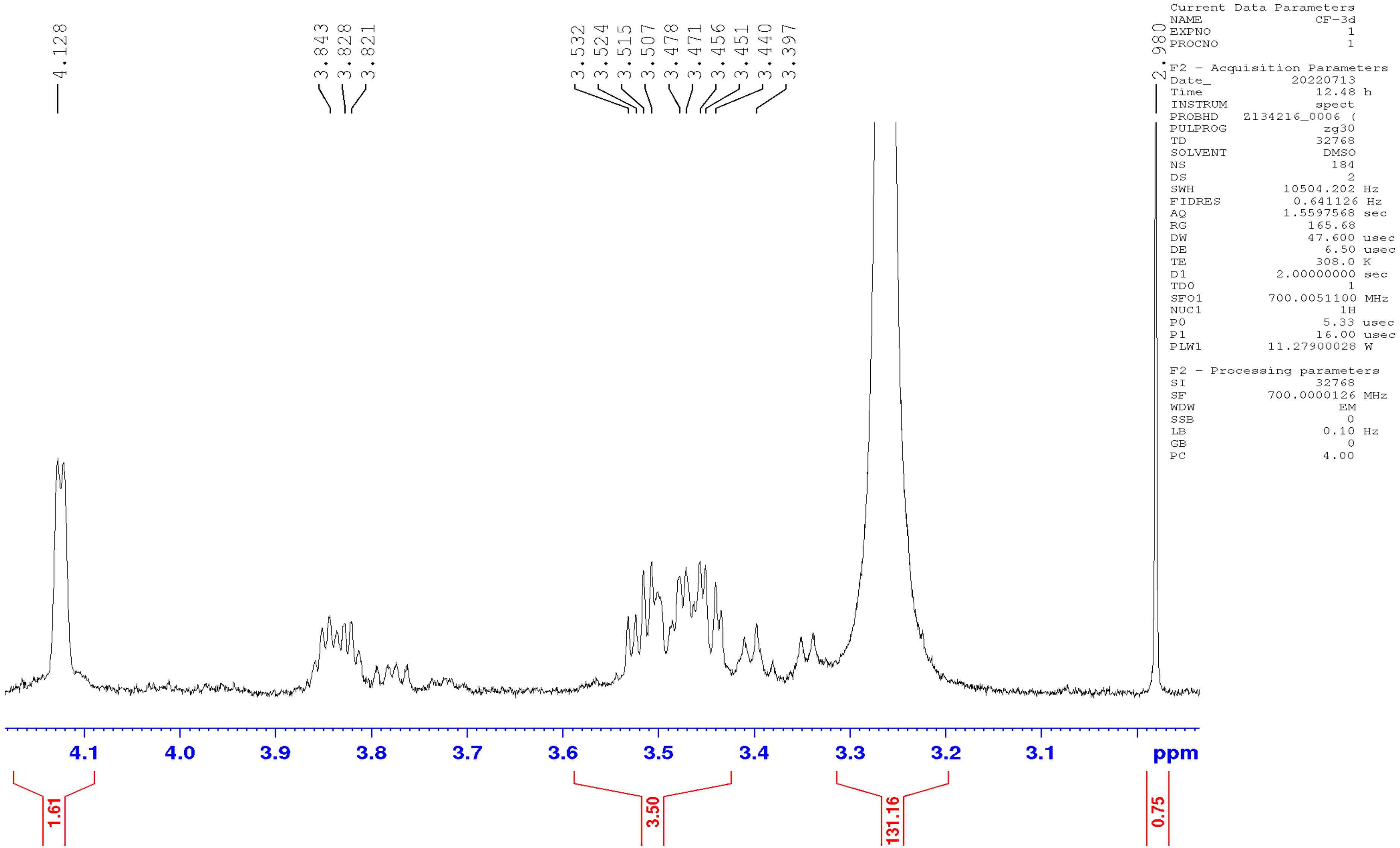

Expanded ^{1}H NMR spectrum (700 MHz, DMSO-d_6) of **1**

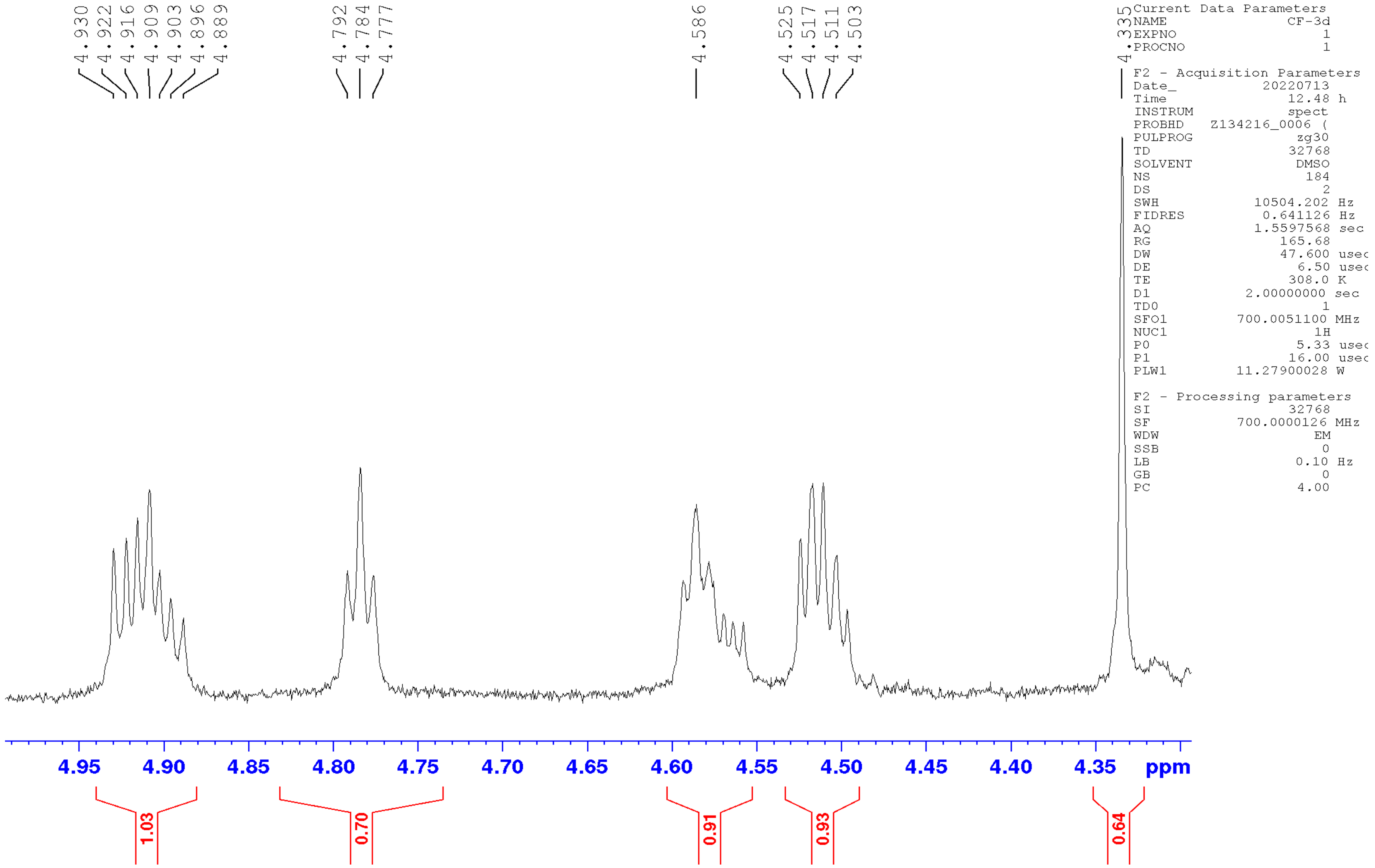

Expanded ^{1}H NMR spectrum (700 MHz, DMSO-d_6) of **1**

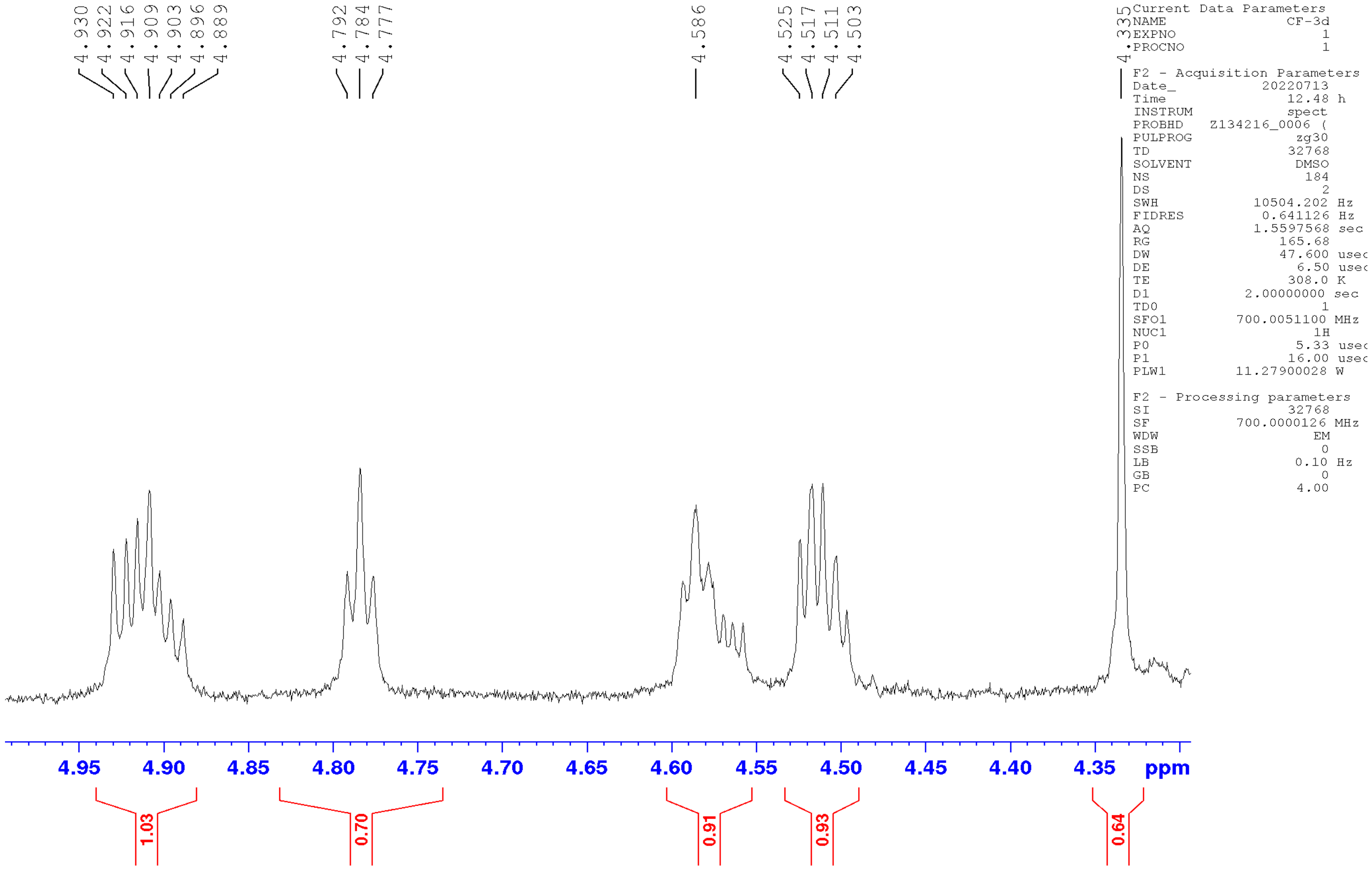

Expanded ^{1}H NMR spectrum (700 MHz, DMSO-d_6) of **1**

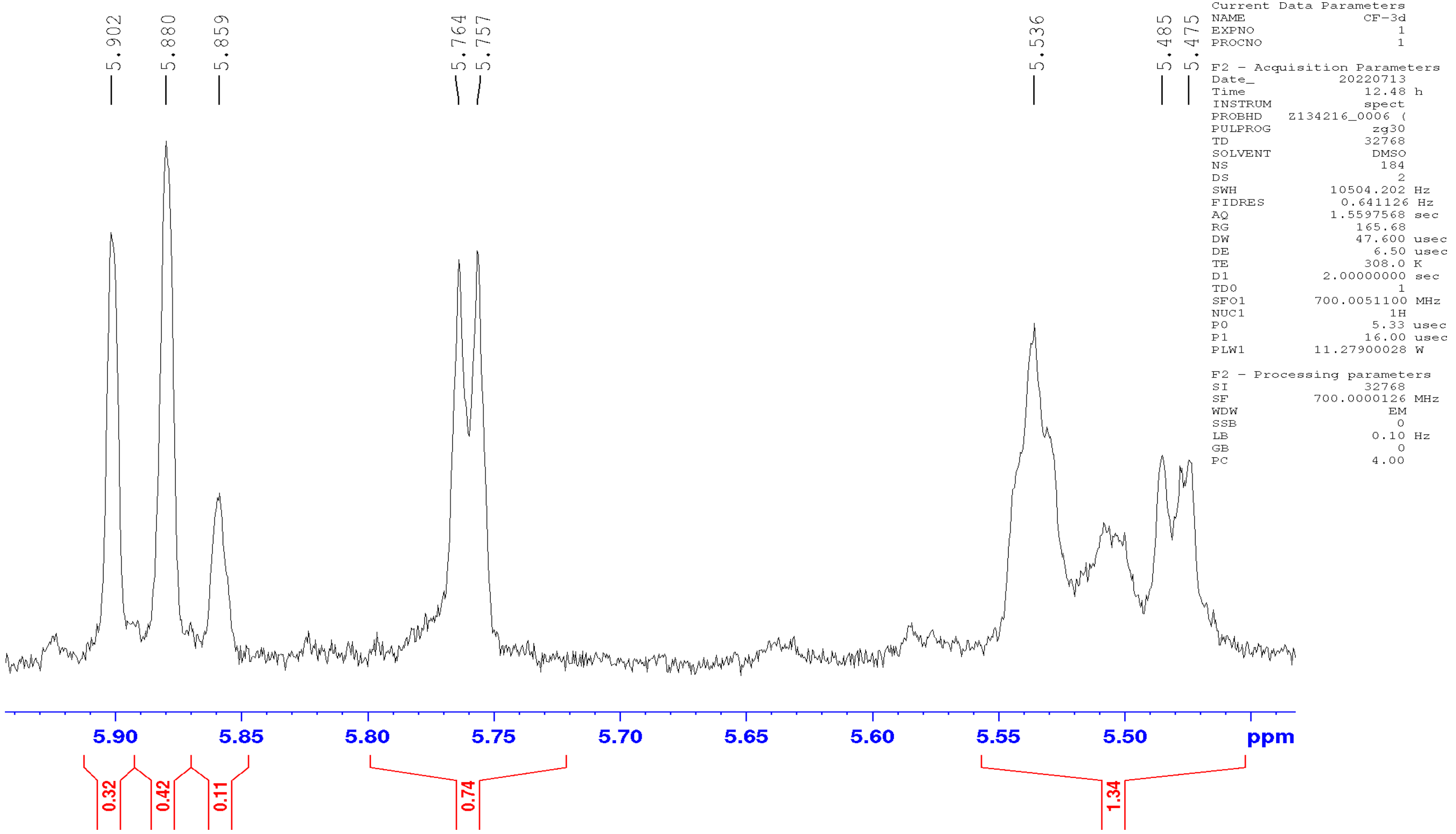

Expanded ^{1}H NMR spectrum (700 MHz, DMSO-d_6) of **1**

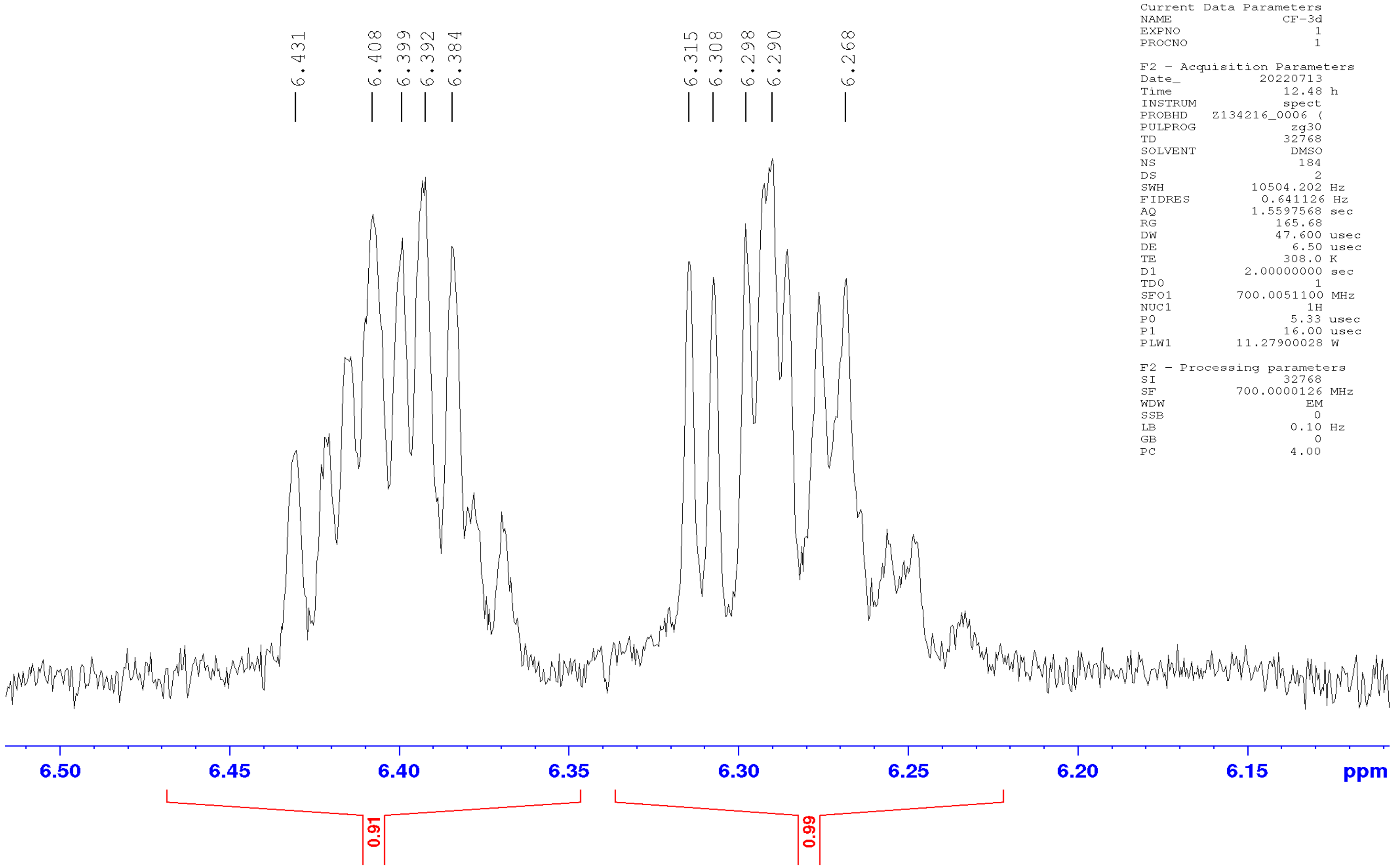

Expanded ^{1}H NMR spectrum (700 MHz, DMSO-d_6) of **1**

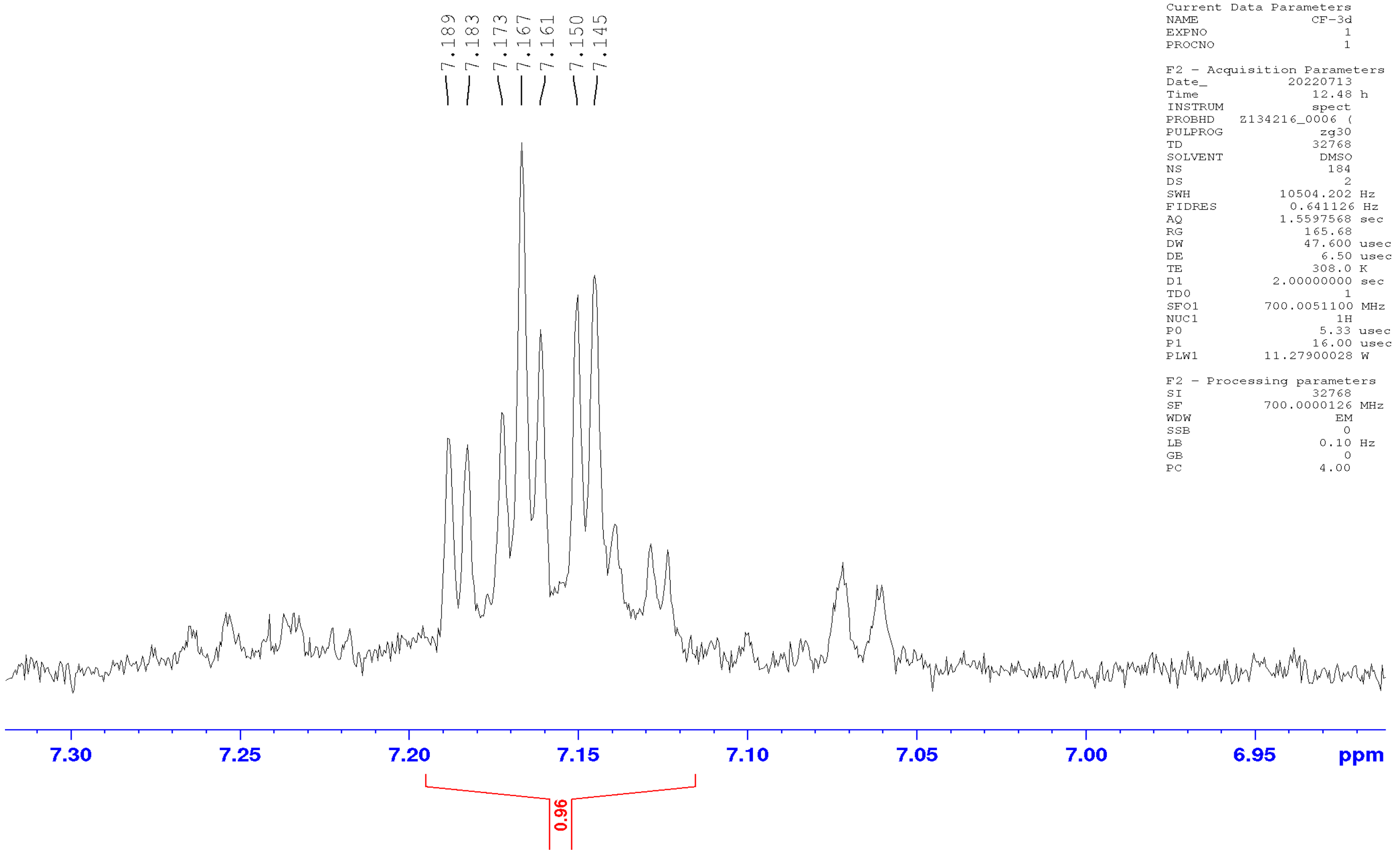

Expanded ^{1}H NMR spectrum (700 MHz, DMSO-d_6) of **1**

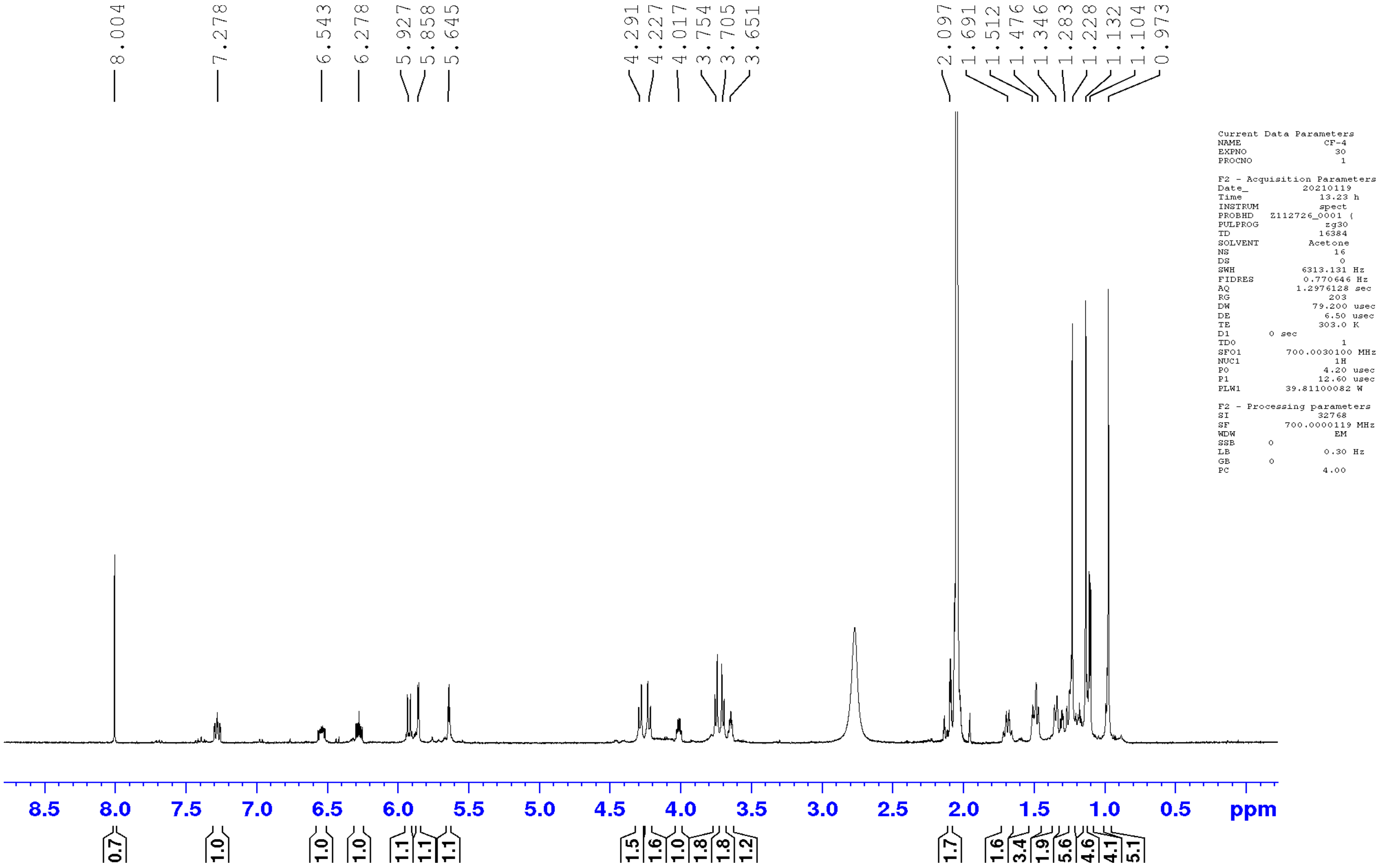

Figure S22. ^{1}H NMR spectrum (700 MHz, acetone-d$_6$) of **2**

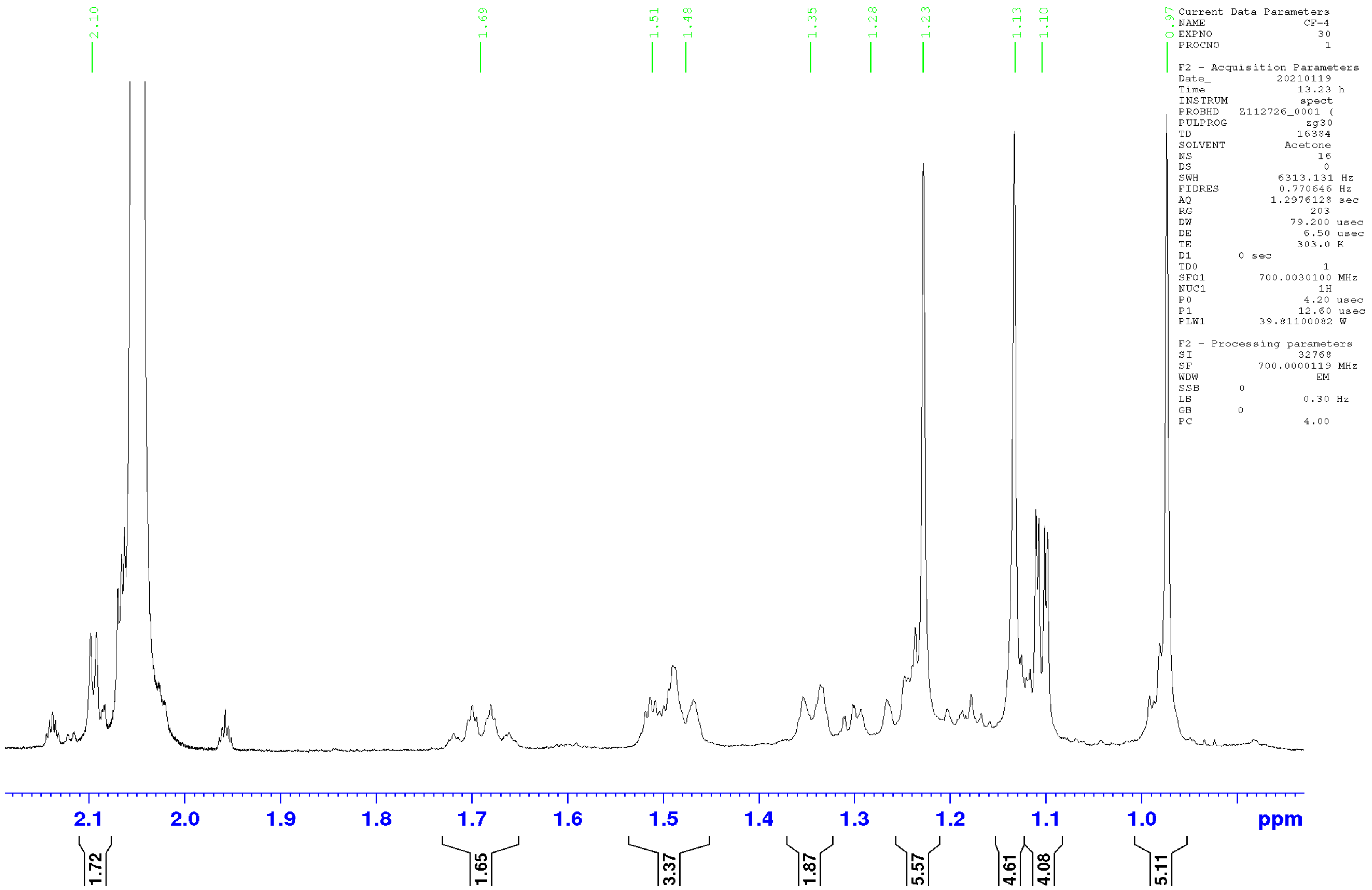

^{1}H NMR spectrum (700 MHz, acetone-d_6) of **2** (expanded)

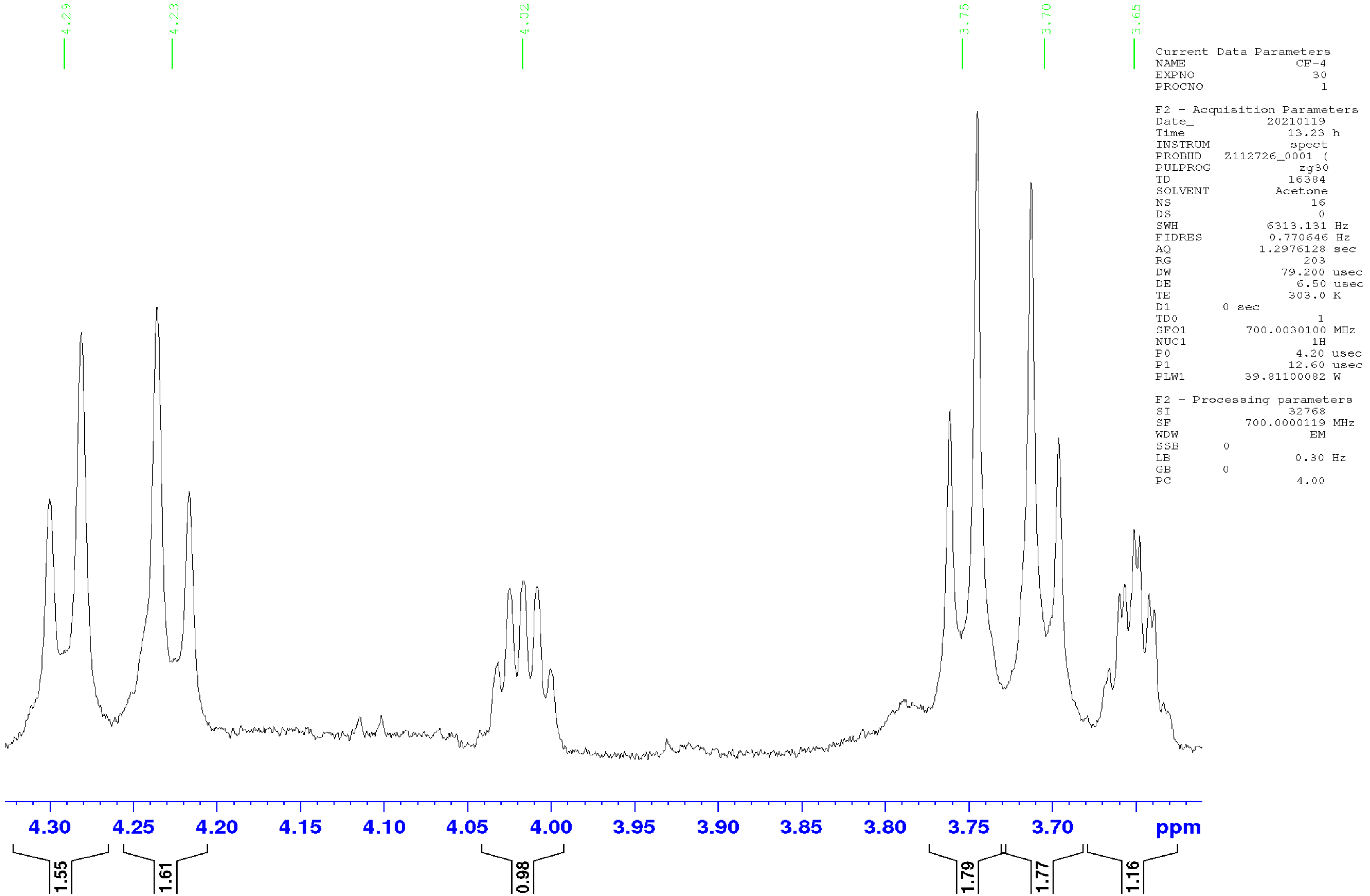

^{1}H NMR spectrum (700 MHz, acetone-d_6) of **2** (expanded)

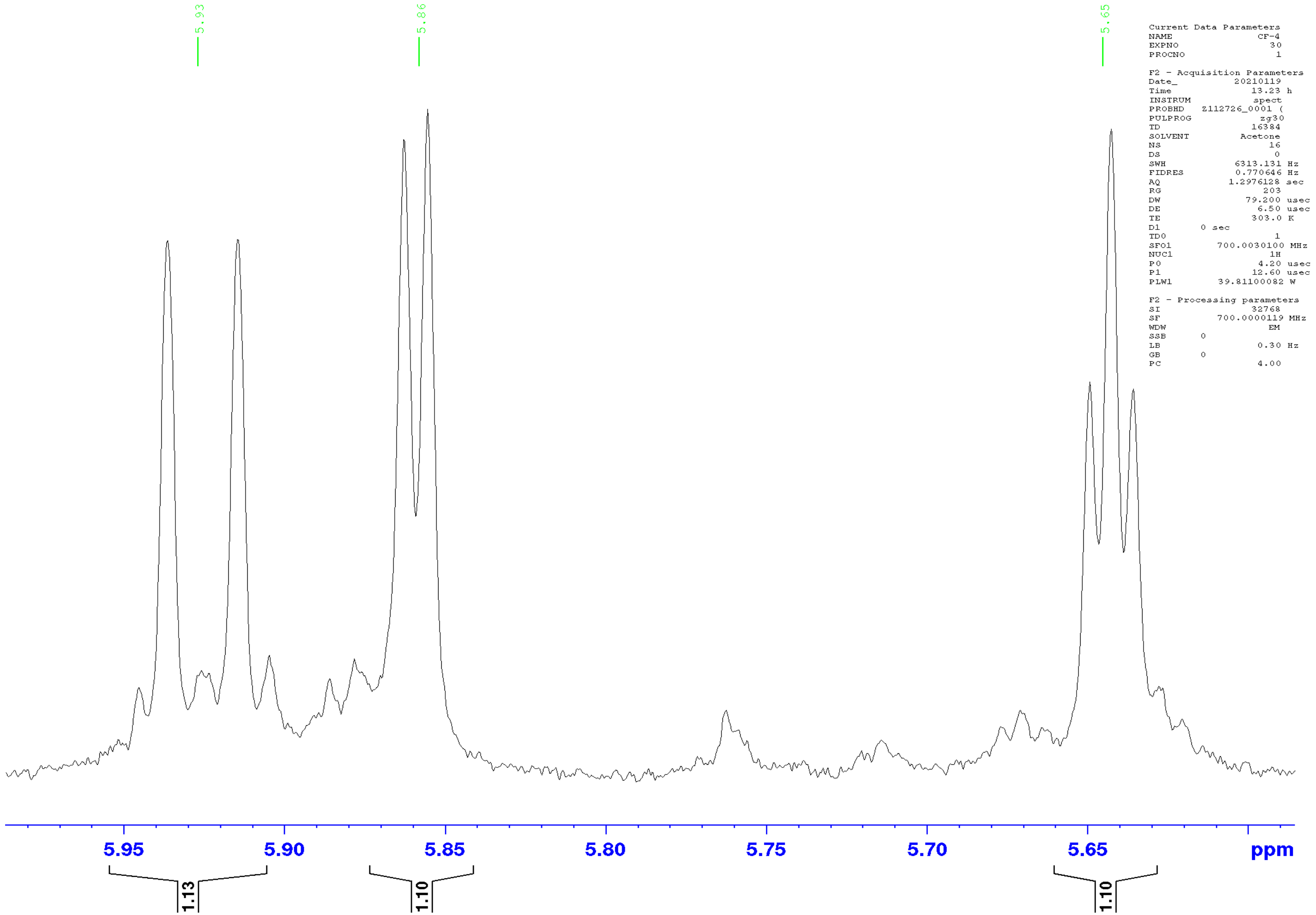

^{1}H NMR spectrum (700 MHz, acetone-d_6) of **2** (expanded)

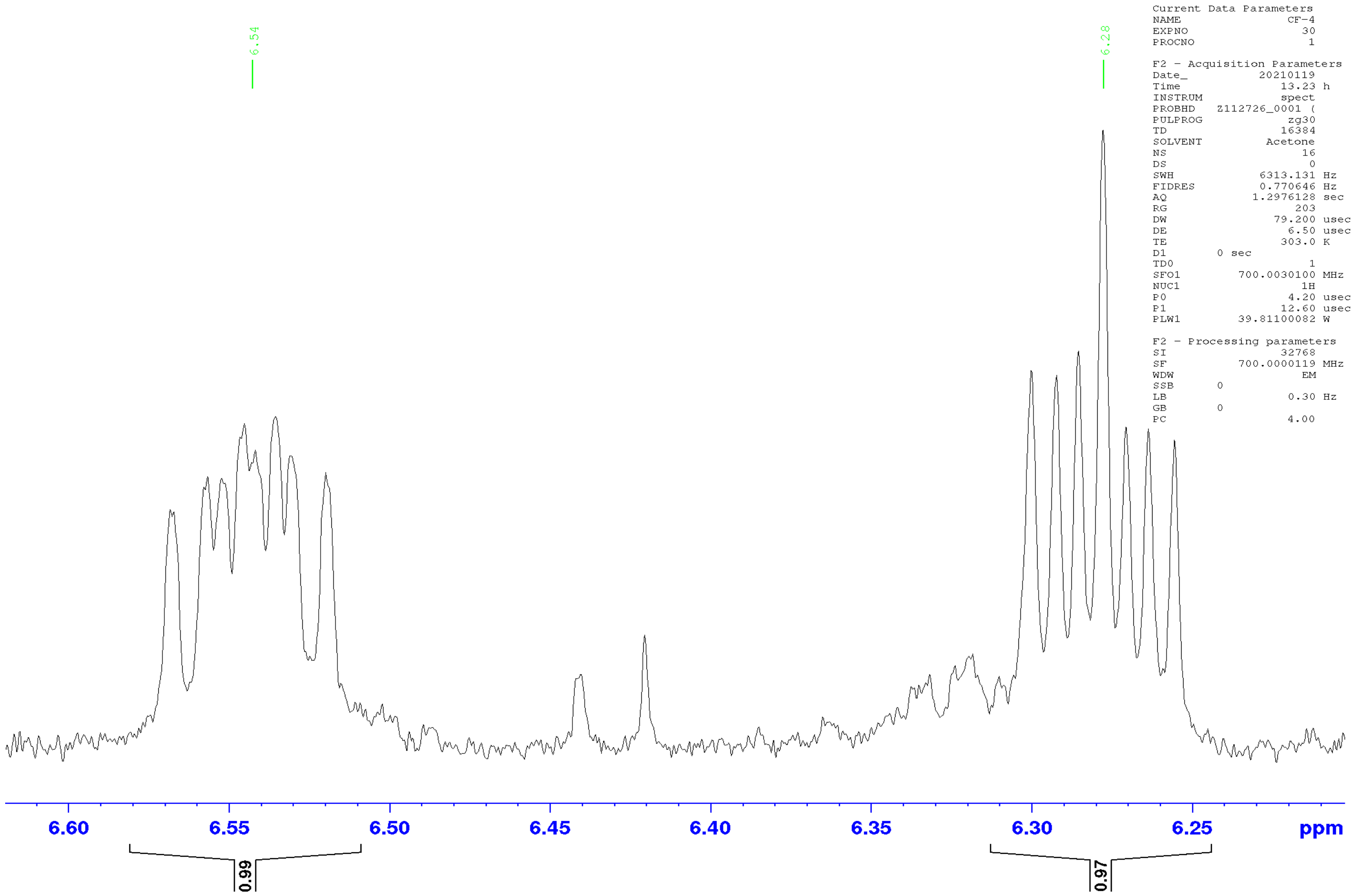

^{1}H NMR spectrum (700 MHz, acetone-d$_6$) of **2** (expanded)

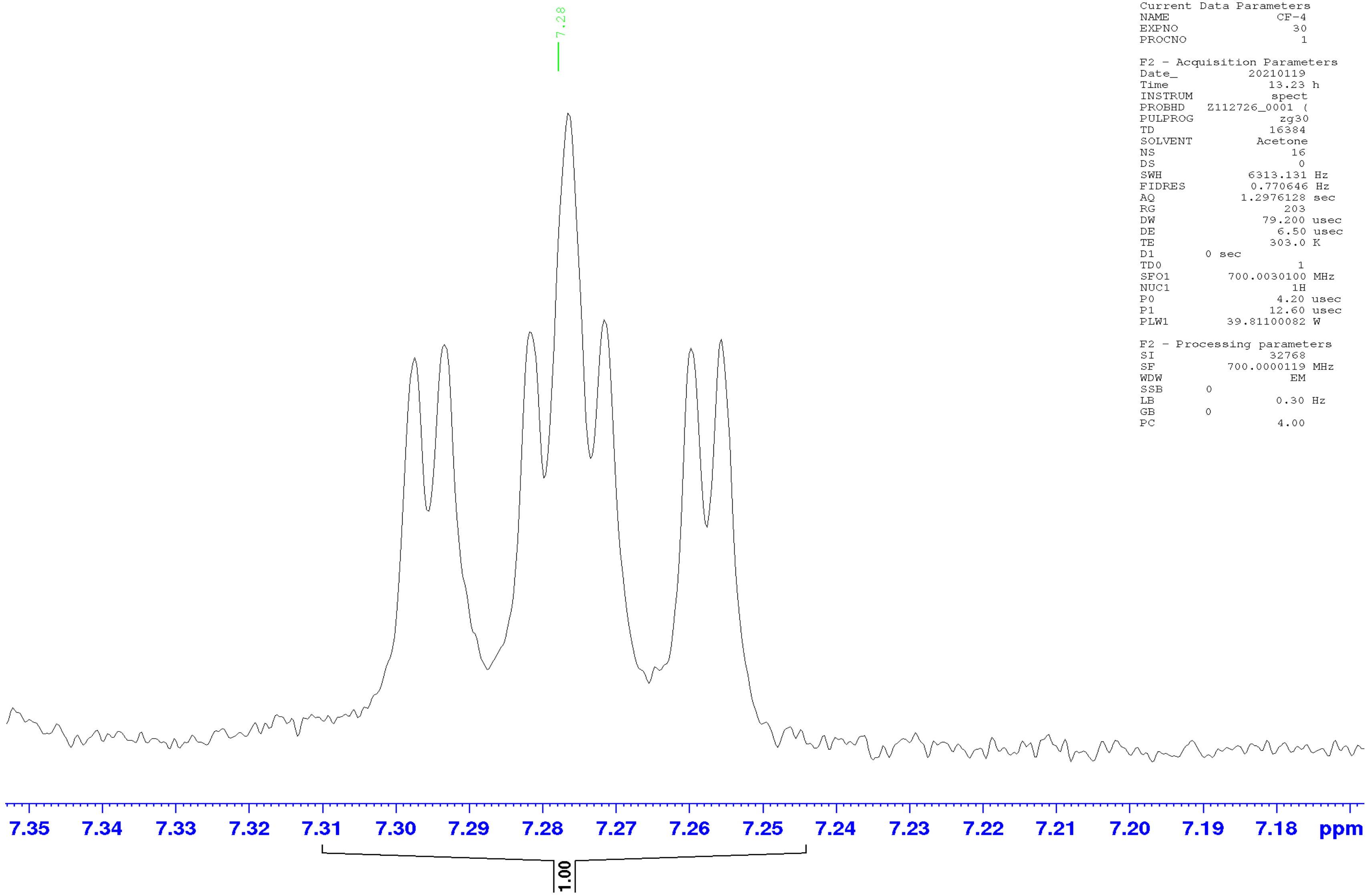

^{1}H NMR spectrum (700 MHz, acetone-d_6) of **2** (expanded)

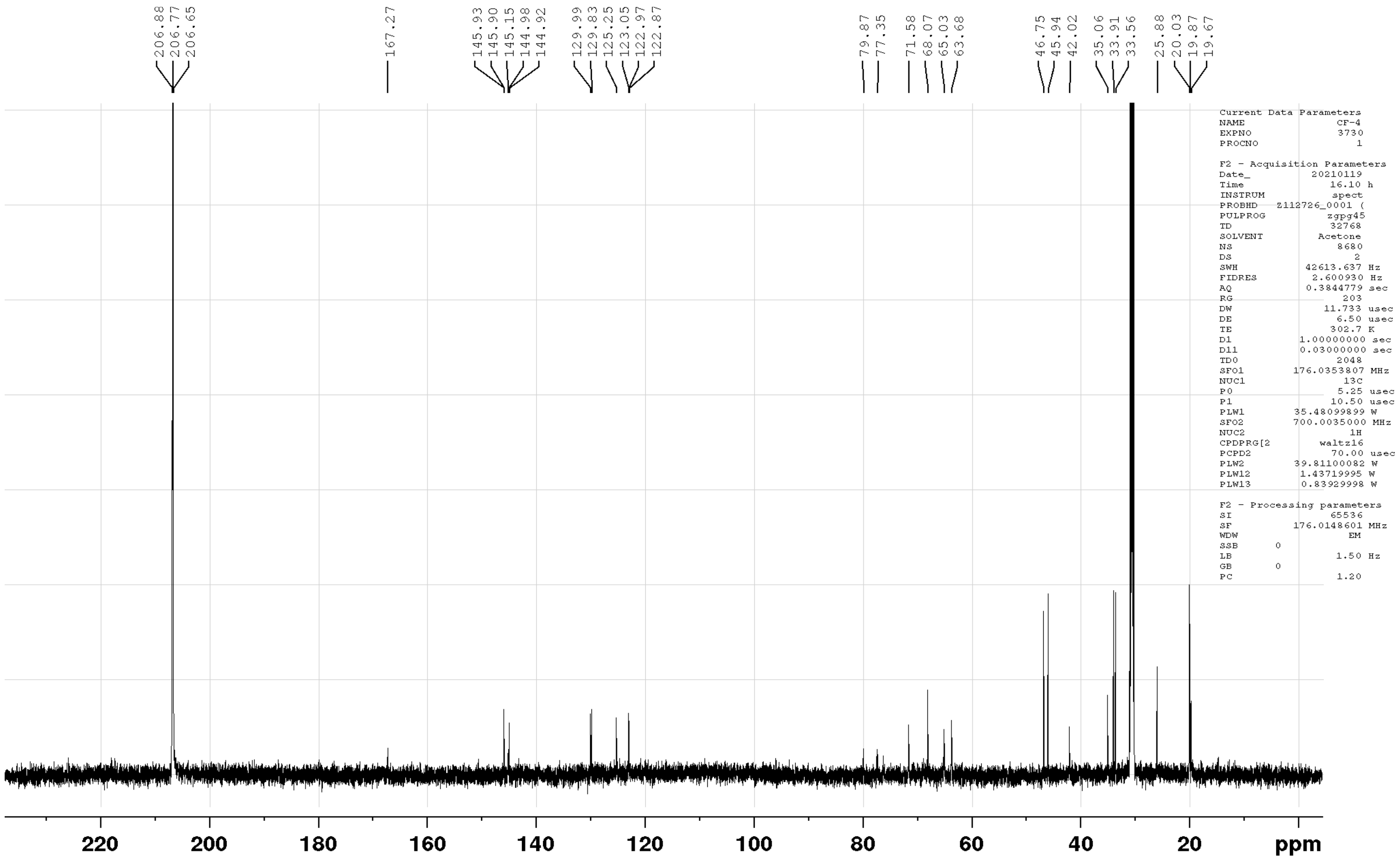

Figure S23. ^{13}C NMR spectrum (175 MHz, acetone-d_6) of **2**

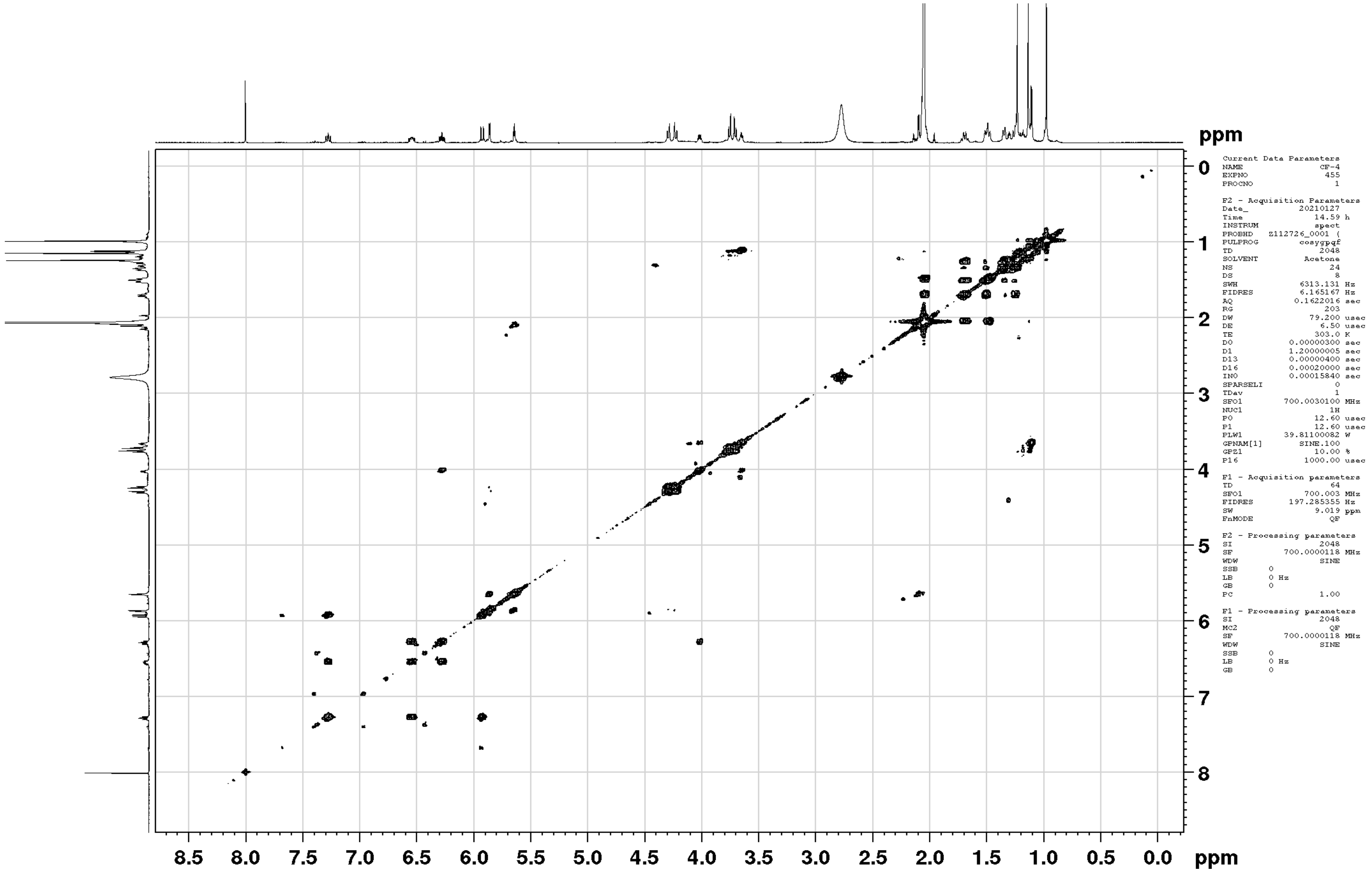

Figure S24. COSY-45 spectrum (700 MHz, acetone-d_6) of **2**

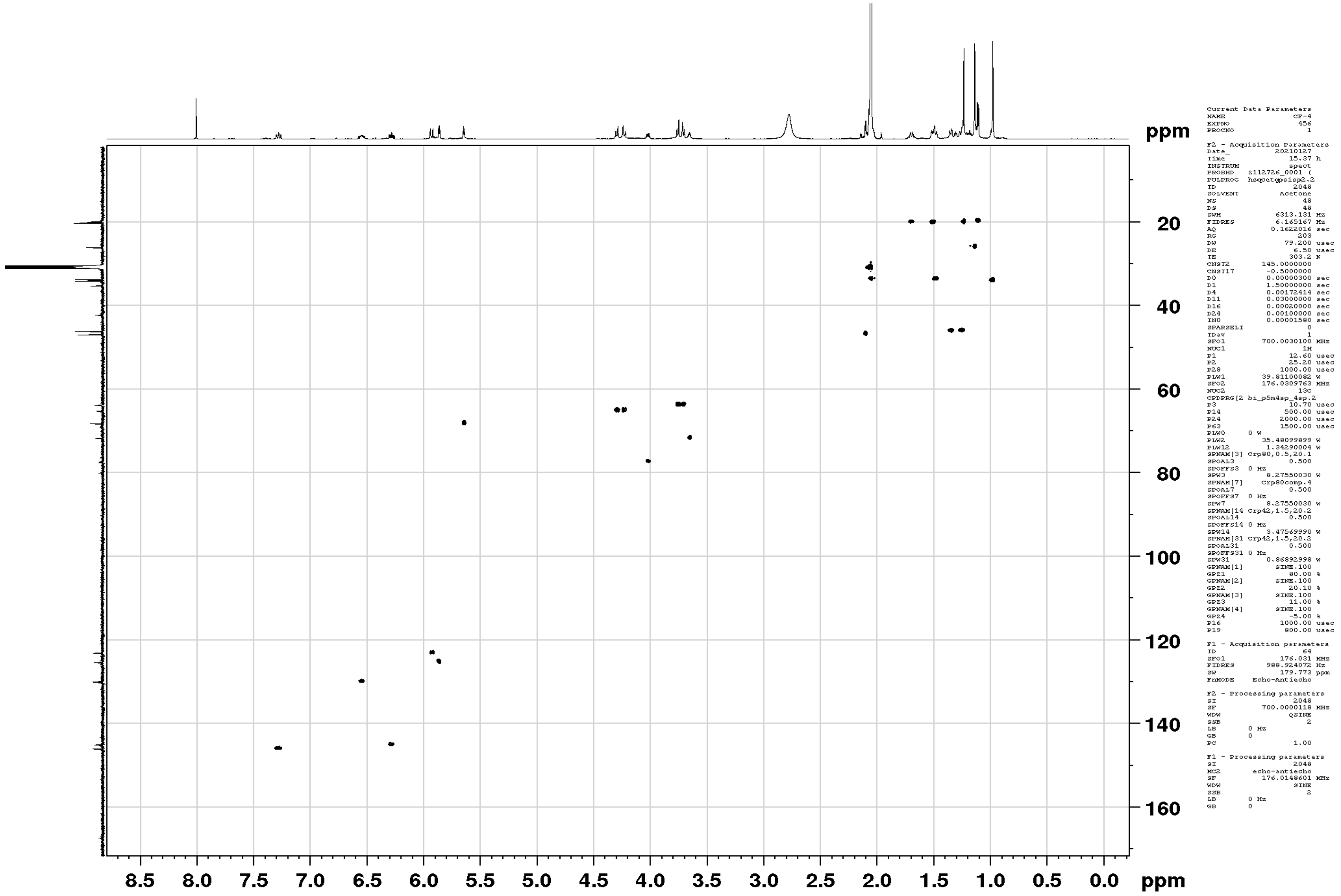

Figure S25. HSQC spectrum (700 MHz, acetone-d_6) of **2**

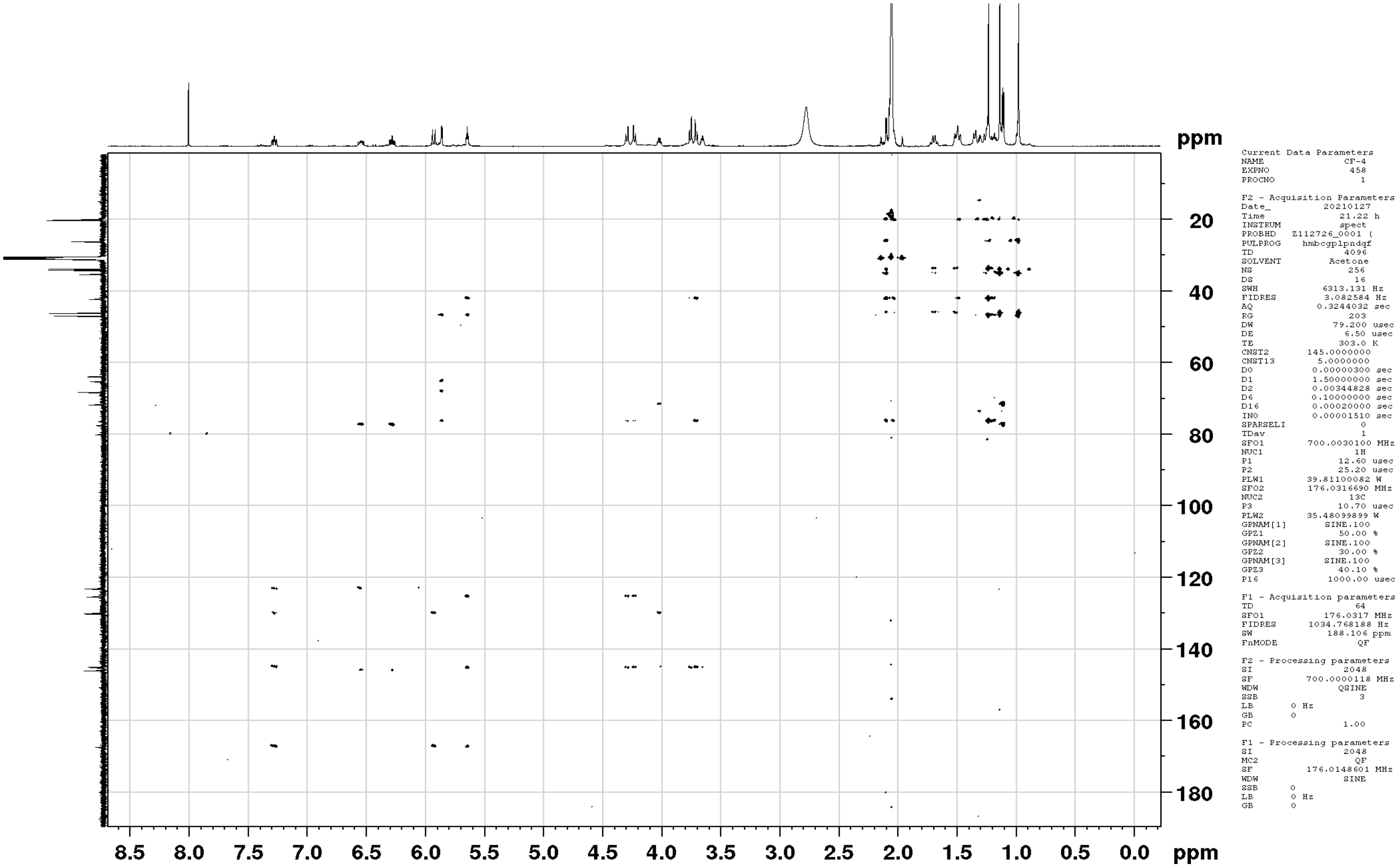

Figure S26. HMBC spectrum (700 MHz, acetone-d_6) of **2**

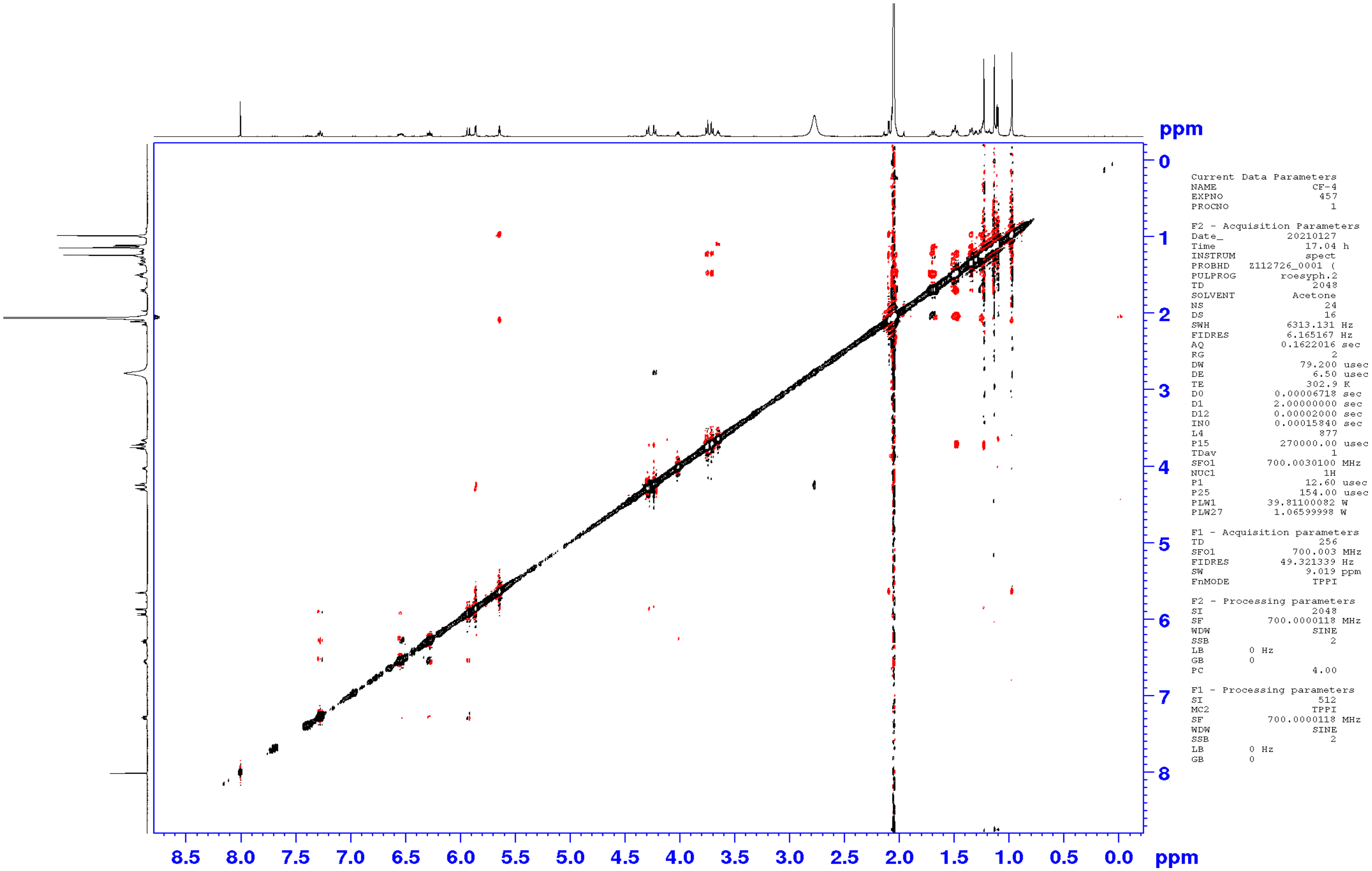

Figure S27. ROESY spectrum (700 MHz, acetone-d_6) of **2**

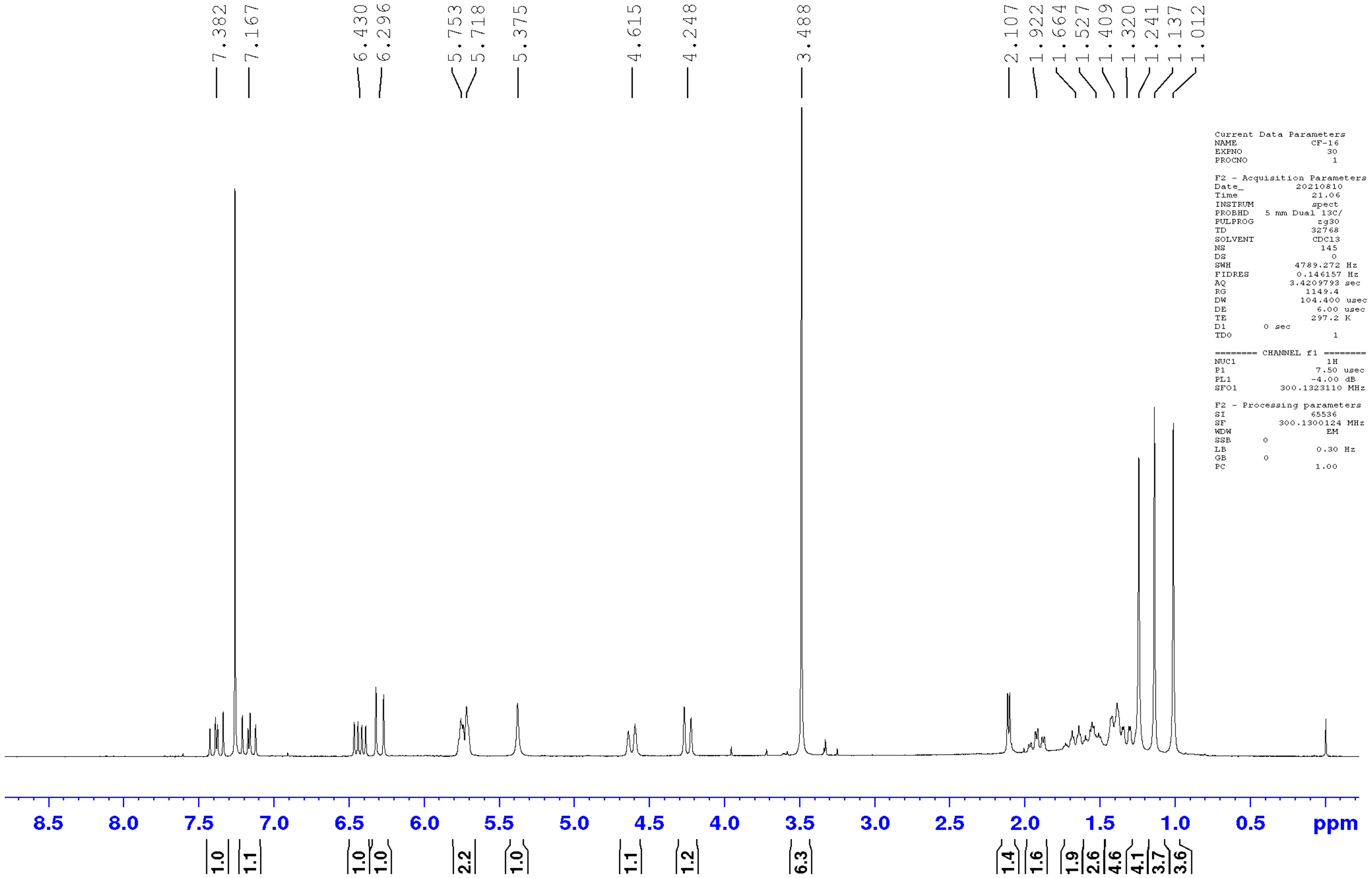

Figure S28. ^{1}H NMR spectrum (300 MHz, $CDCl_3$) of **3**

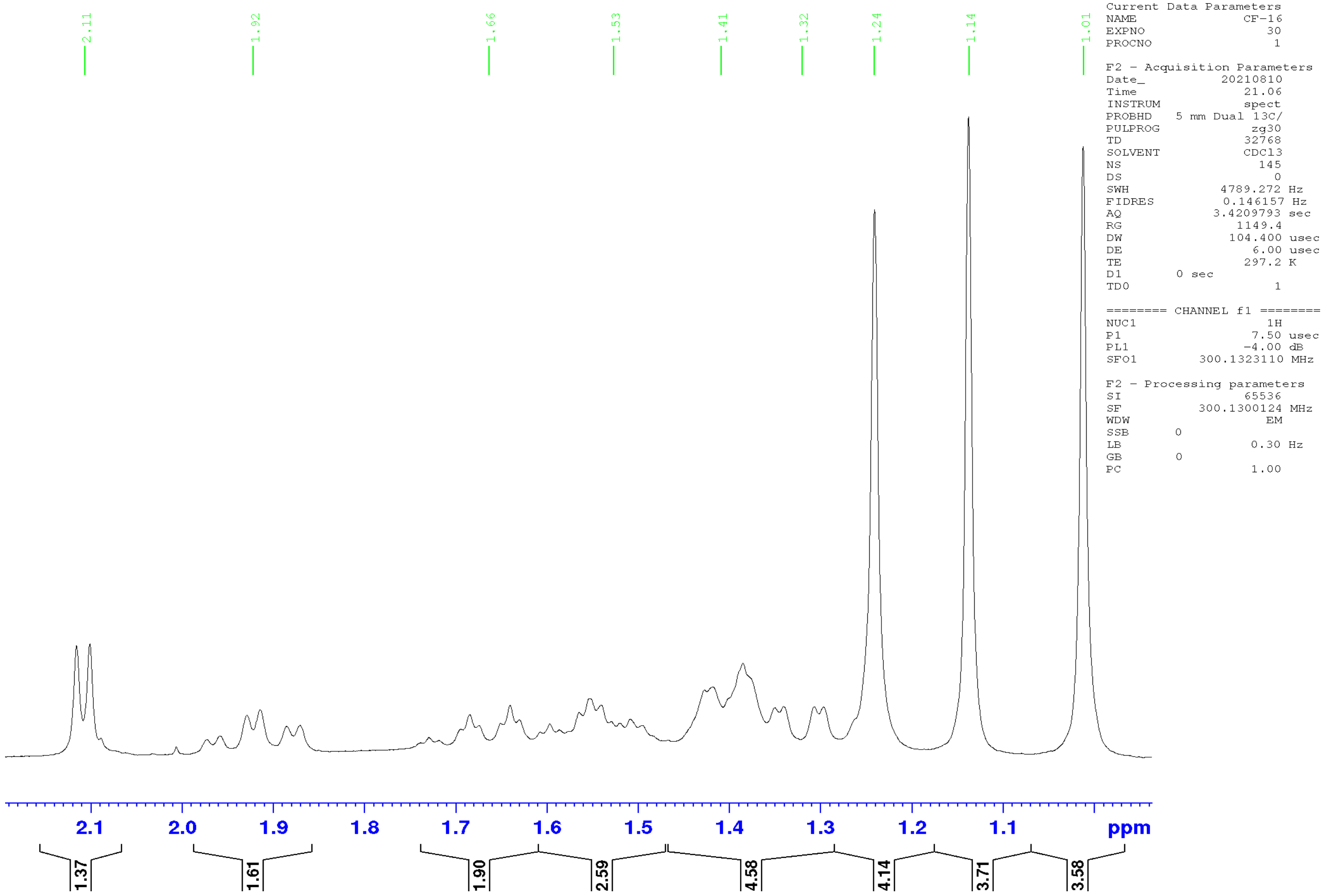

^{1}H NMR spectrum (300 MHz, $CDCl_3$) of **3** (expanded)

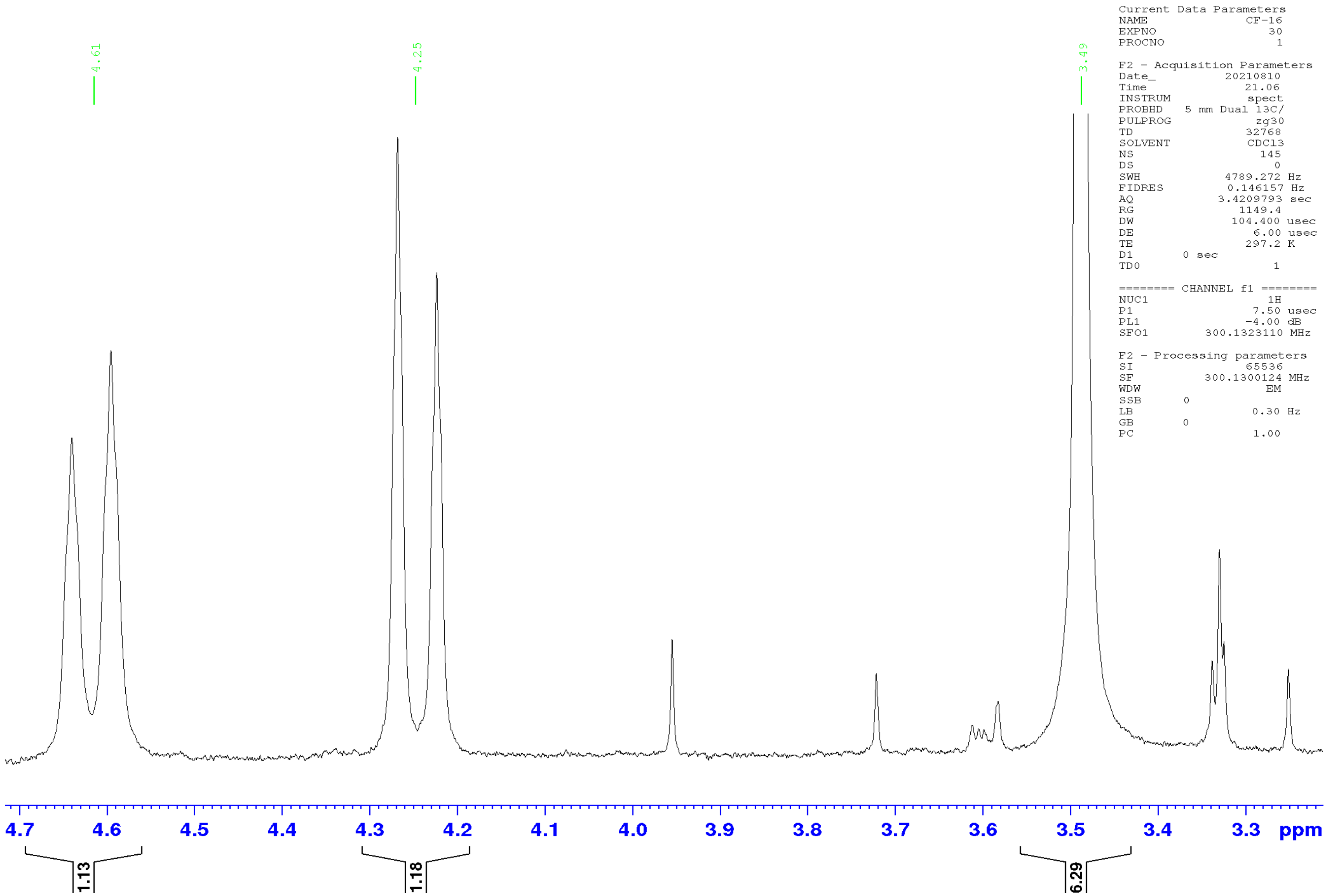

^{1}H NMR spectrum (300 MHz, $CDCl_3$) of **3** (expanded)

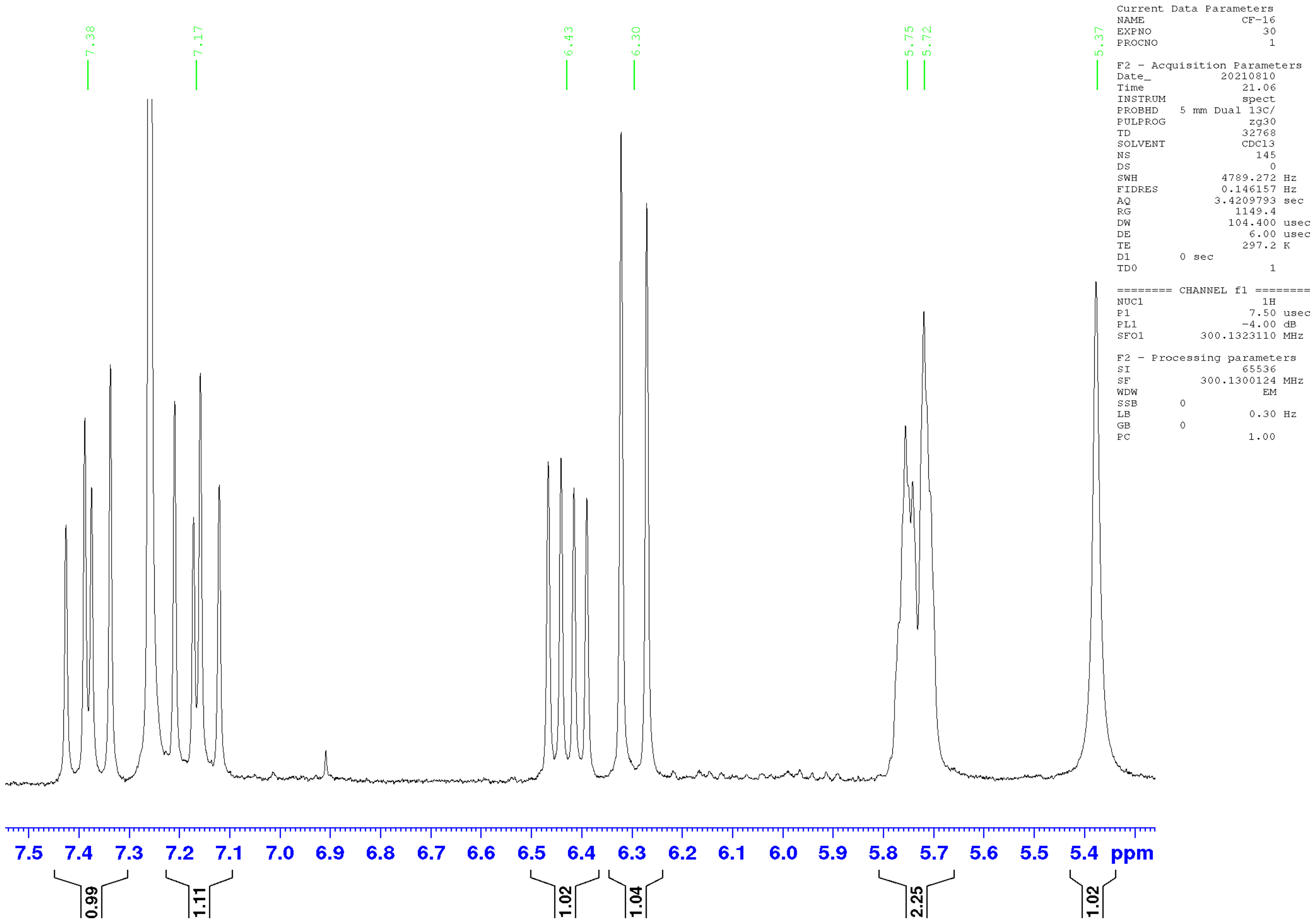

^{1}H NMR spectrum (300 MHz, $CDCl_3$) of **3** (expanded)

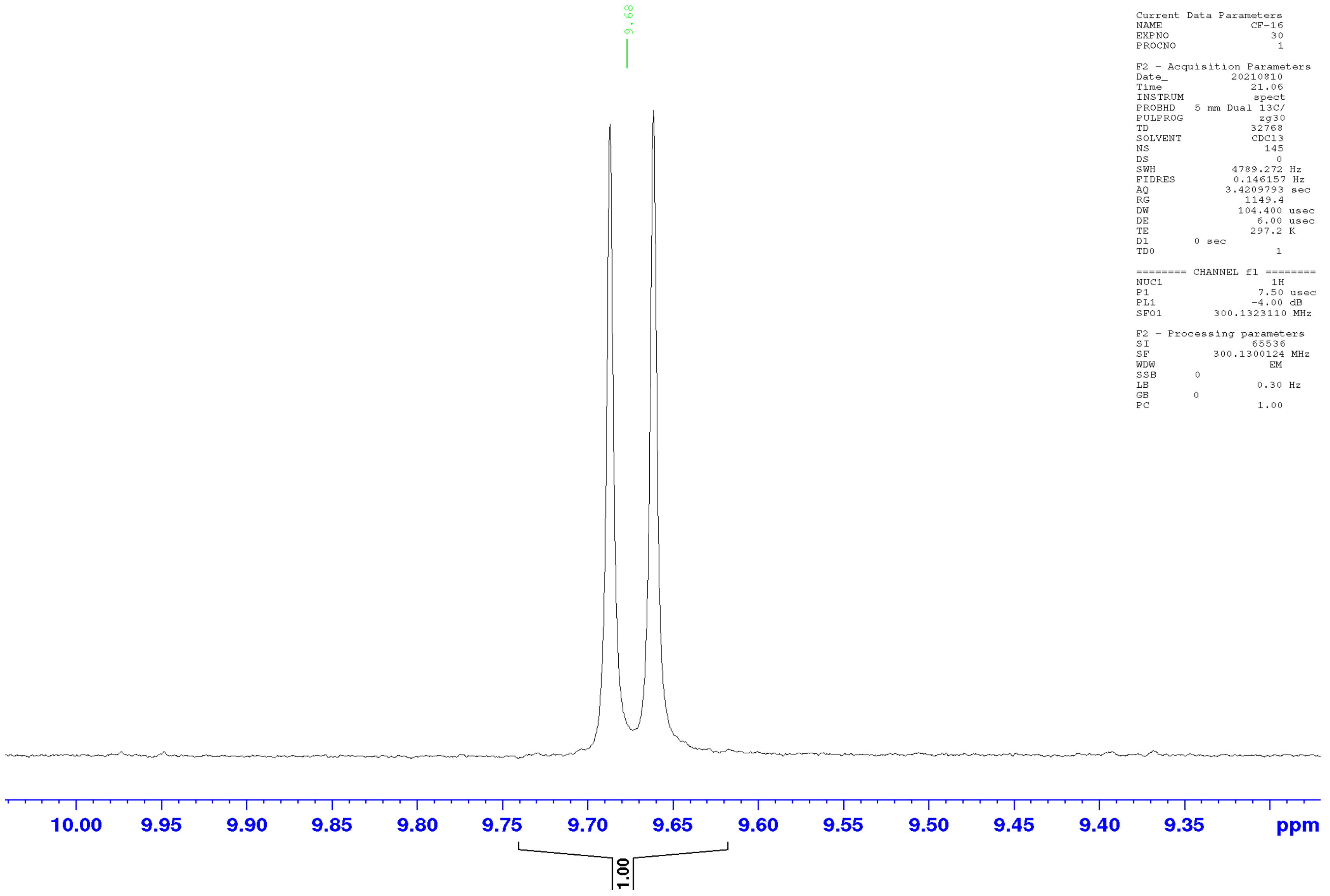

^{1}H NMR spectrum (300 MHz, $CDCl_3$) of **3** (expanded)

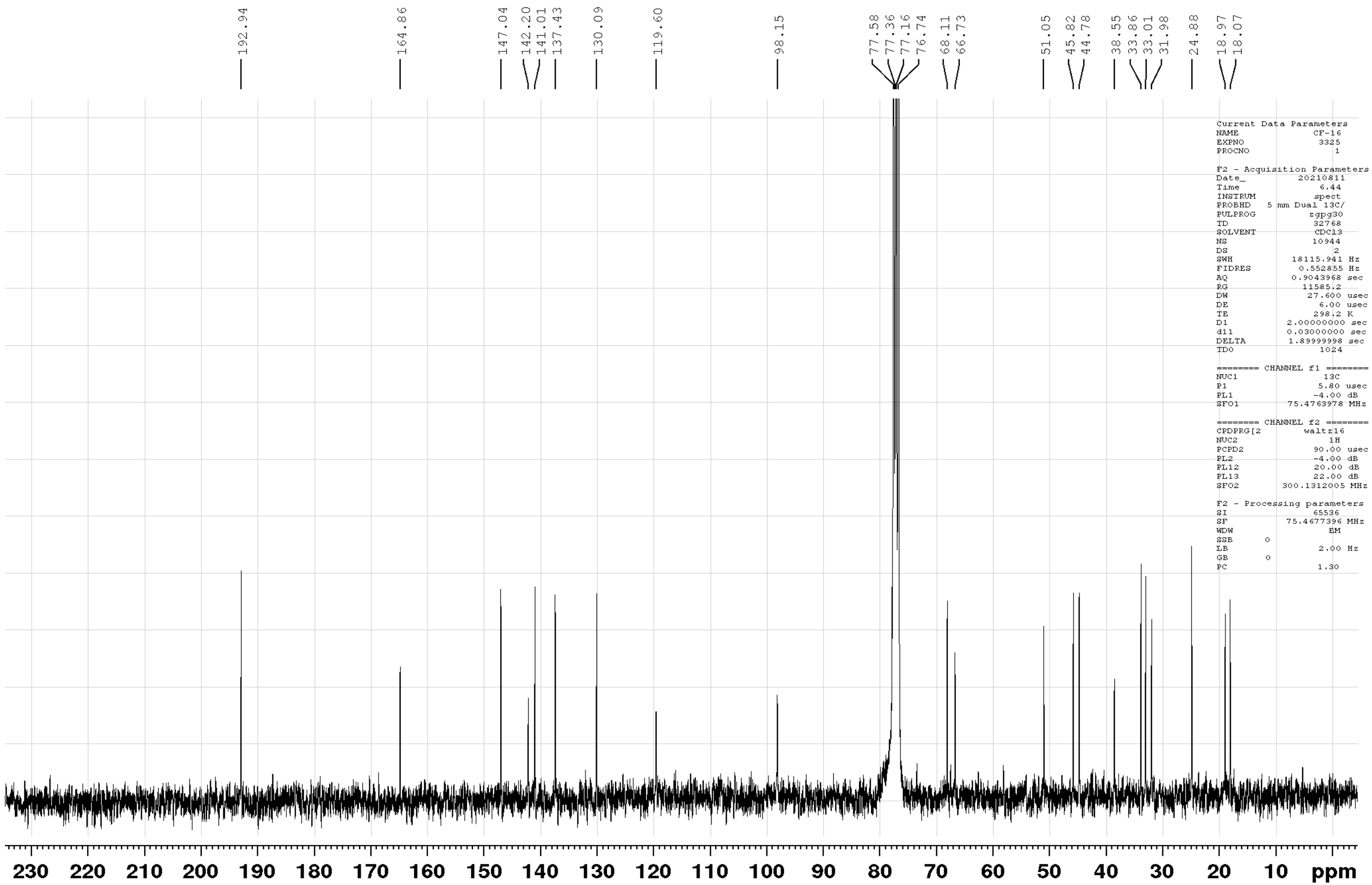

Figure S29. ^{13}C NMR spectrum (75 MHz, $CDCl_3$) of **3**

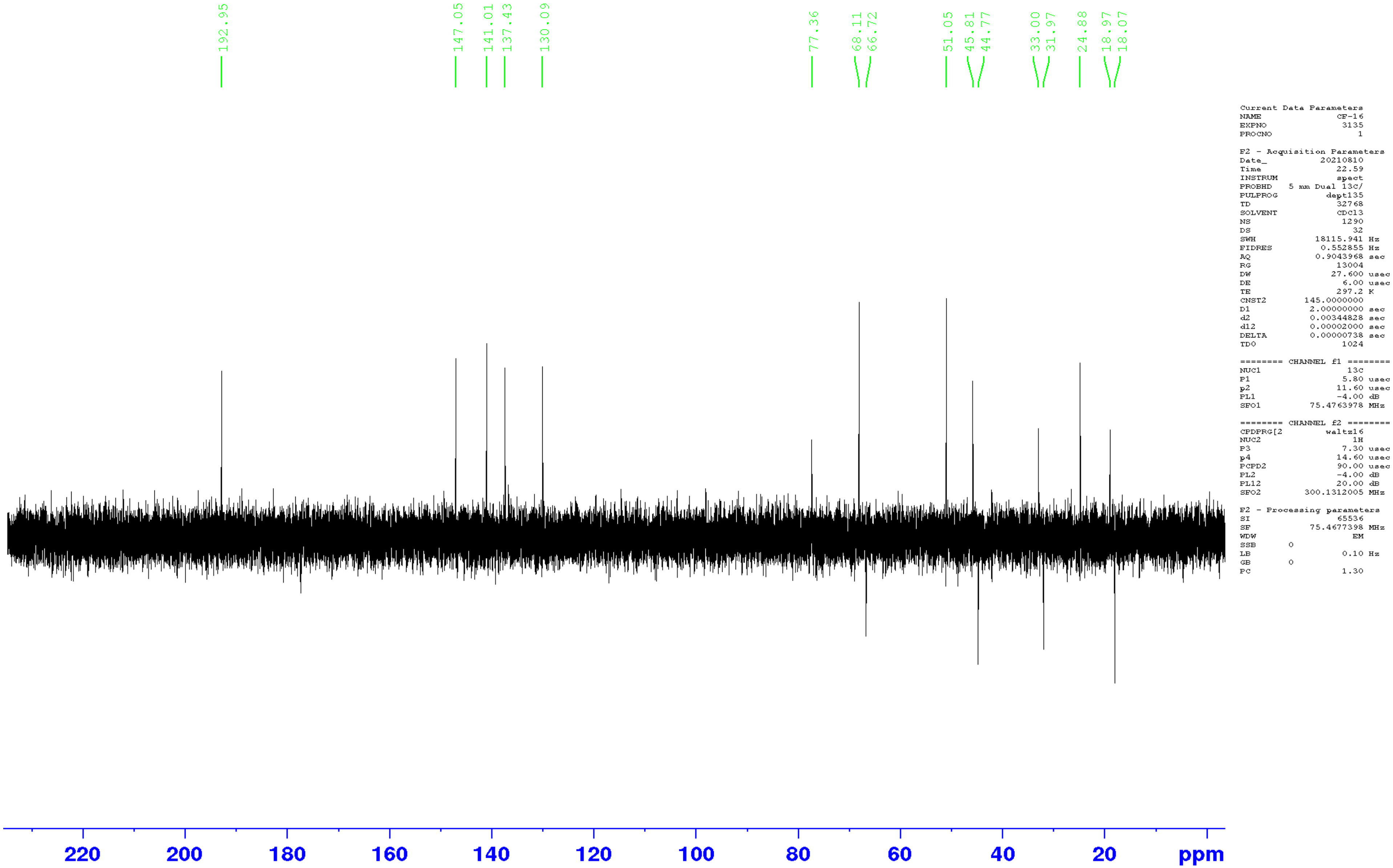

Figure S30. DEPT-135 NMR spectrum (75 MHz, $CDCl_3$) of **3**

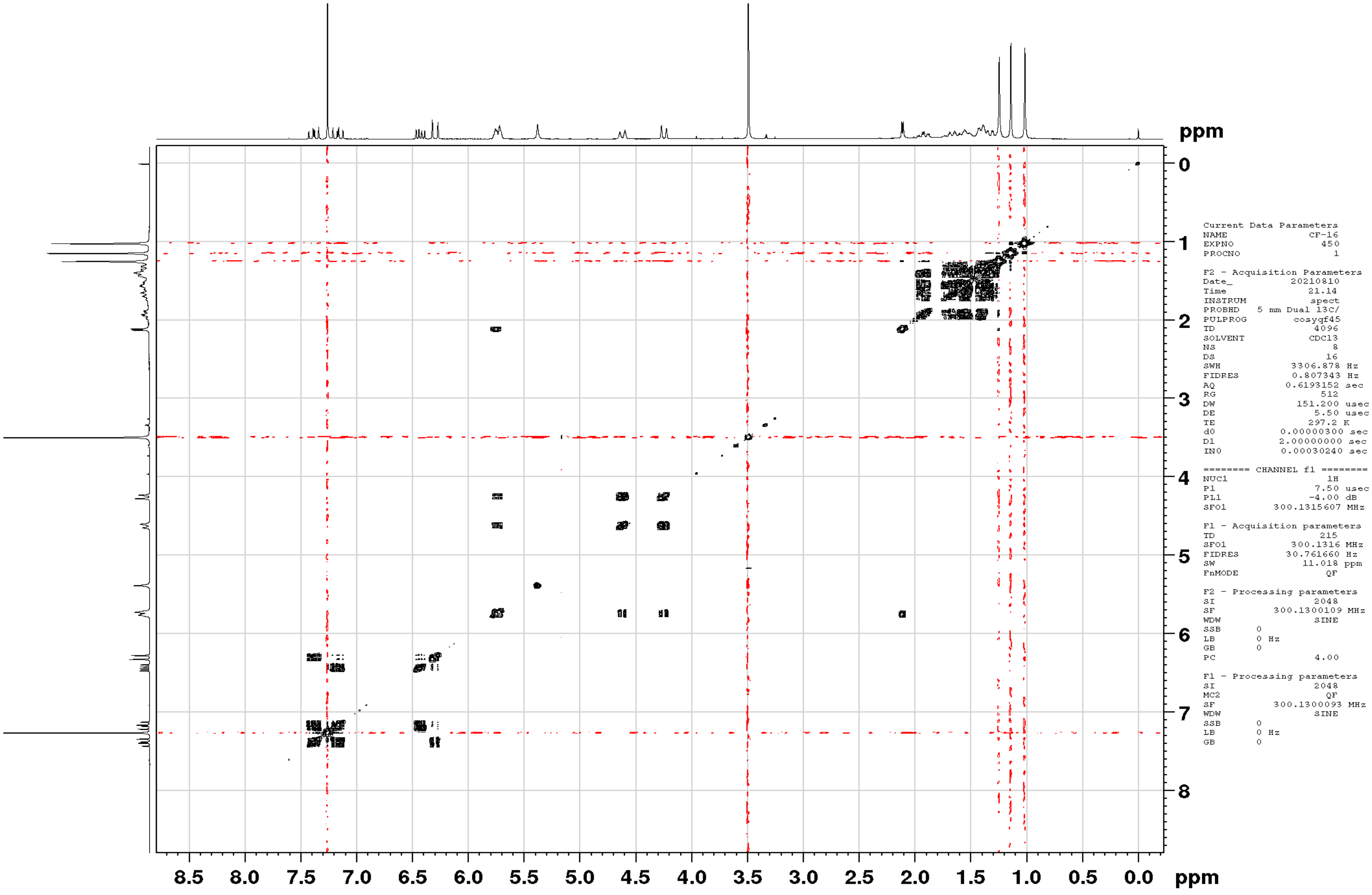

Figure S31. COSY-45 spectrum (300 MHz, $CDCl_3$) of **3**

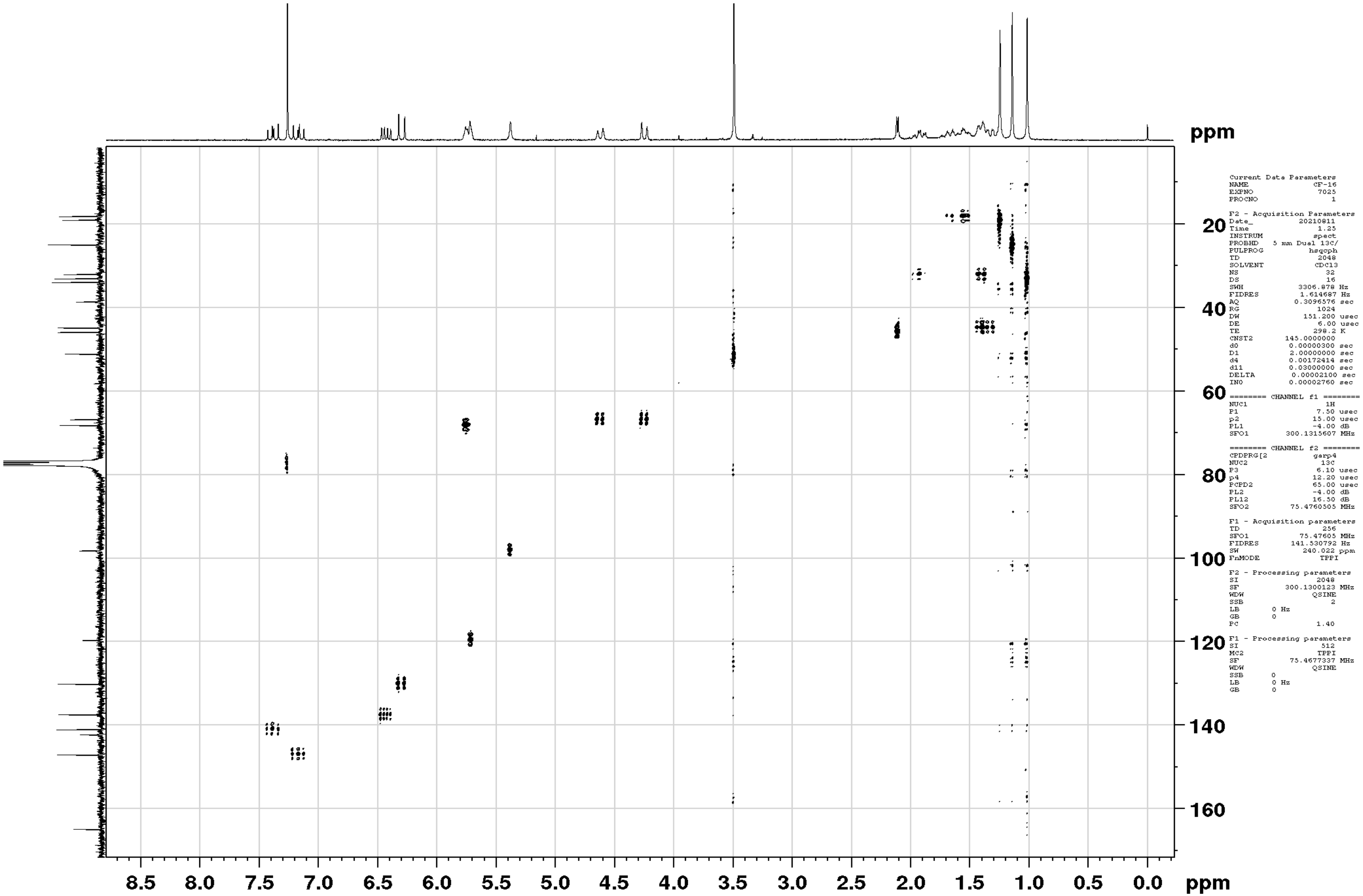

Figure S32. HSQC spectrum (300 MHz, $CDCl_3$) of **3**

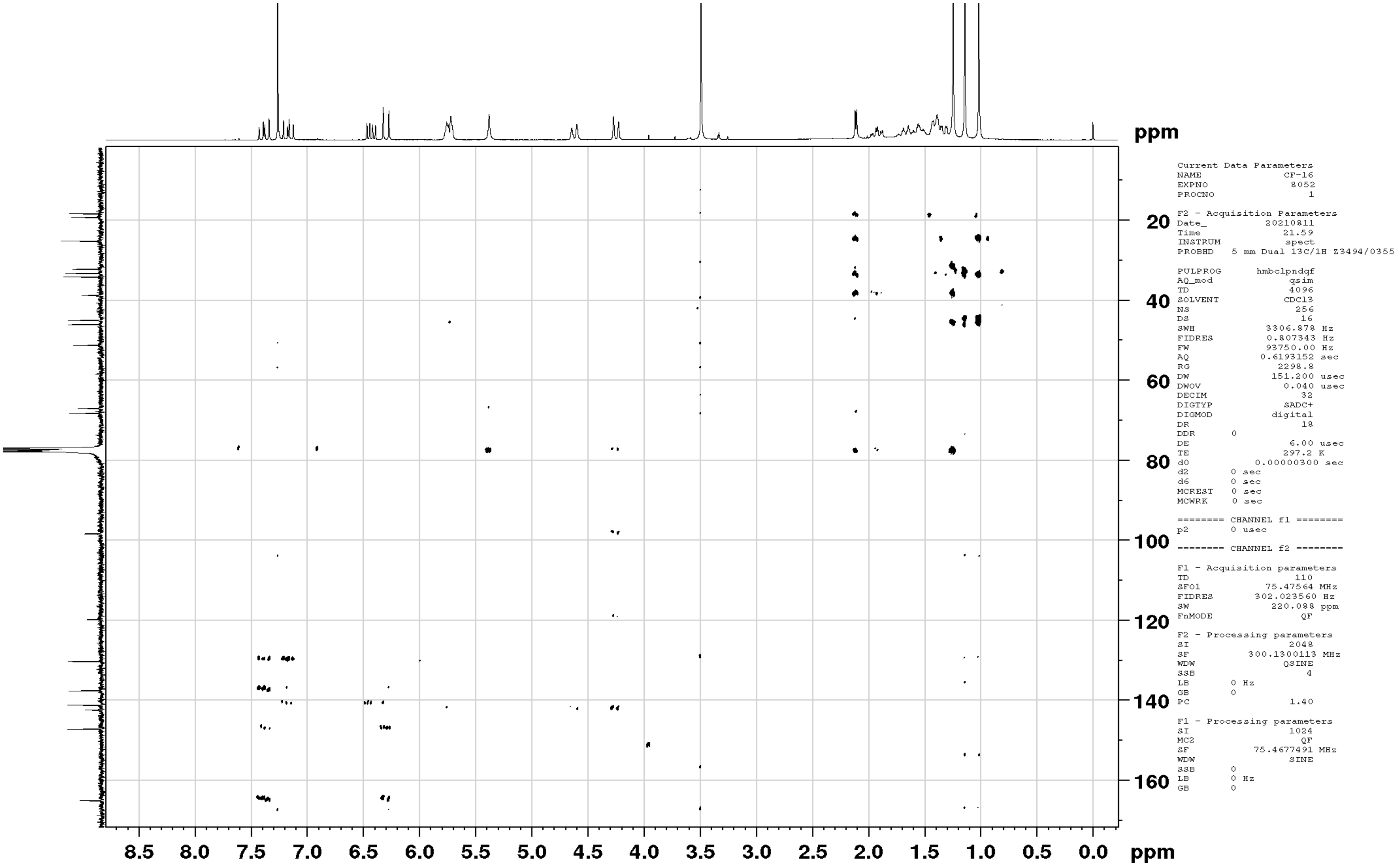

Figure S33. HMBC spectrum (300 MHz, $CDCl_3$) of **3**

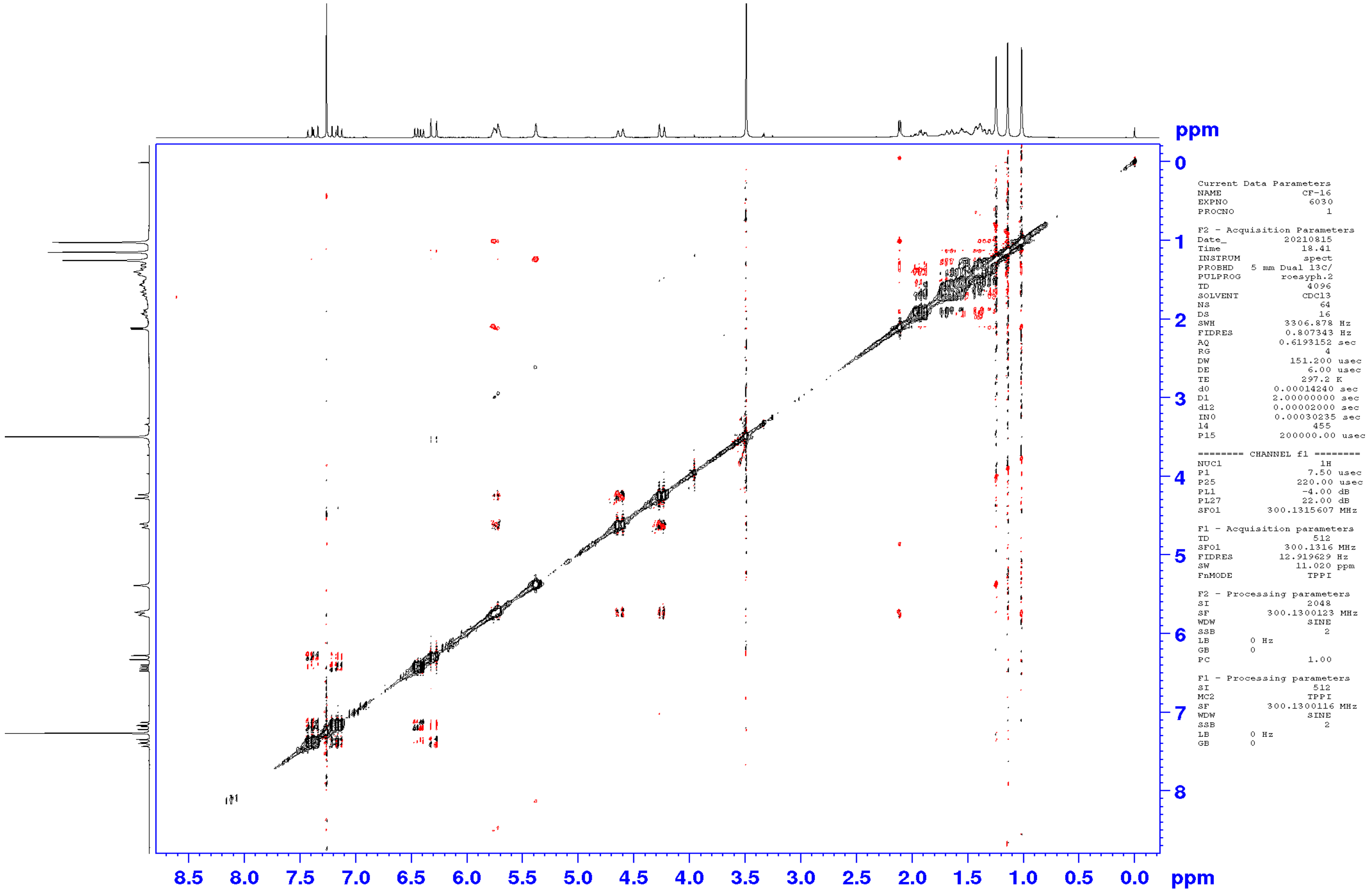

Figure S34. ROESY spectrum (300 MHz, $CDCl_3$) of **3**

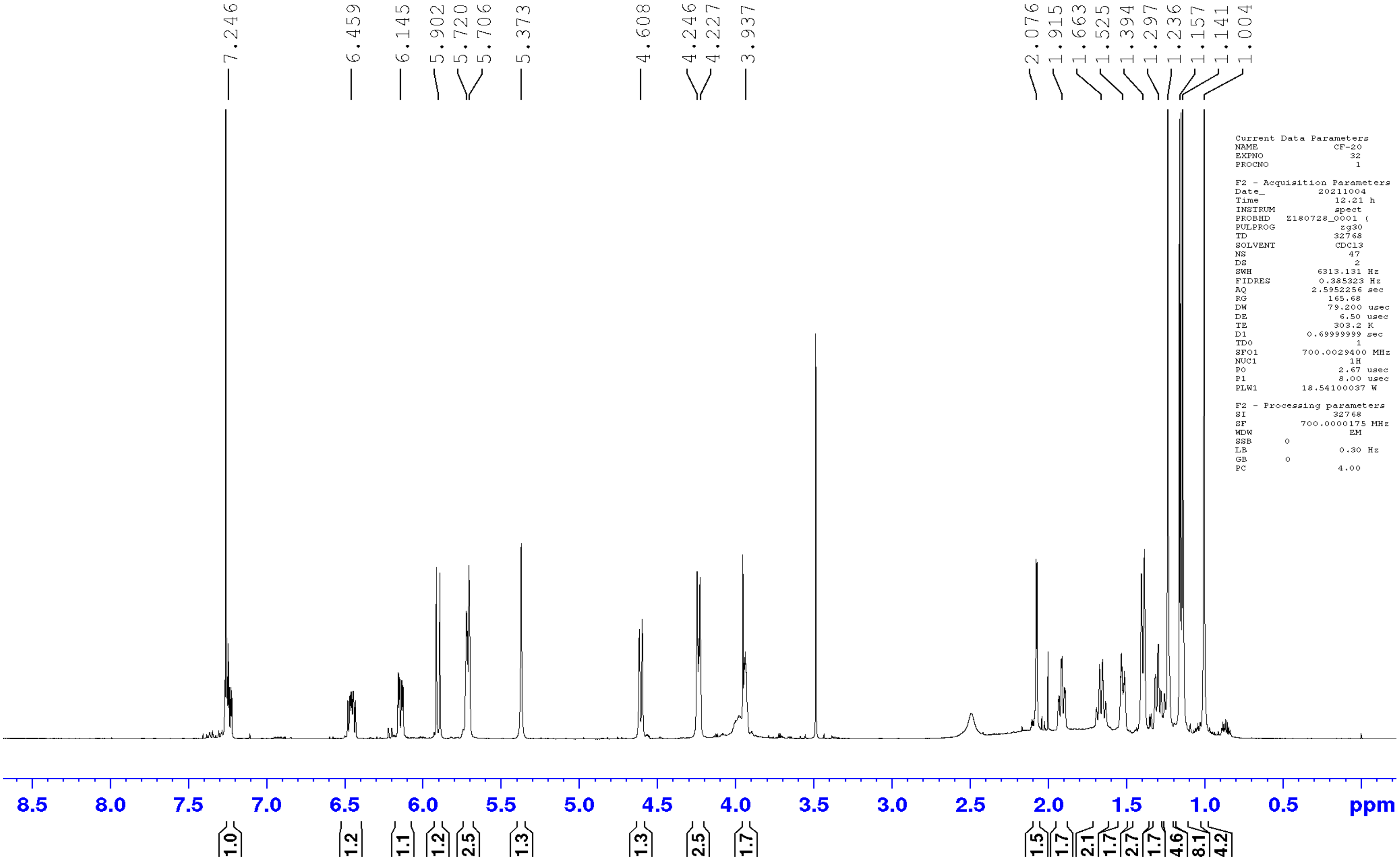

Figure S35. ^{1}H NMR spectrum (700 MHz, $CDCl_3$) of **4**

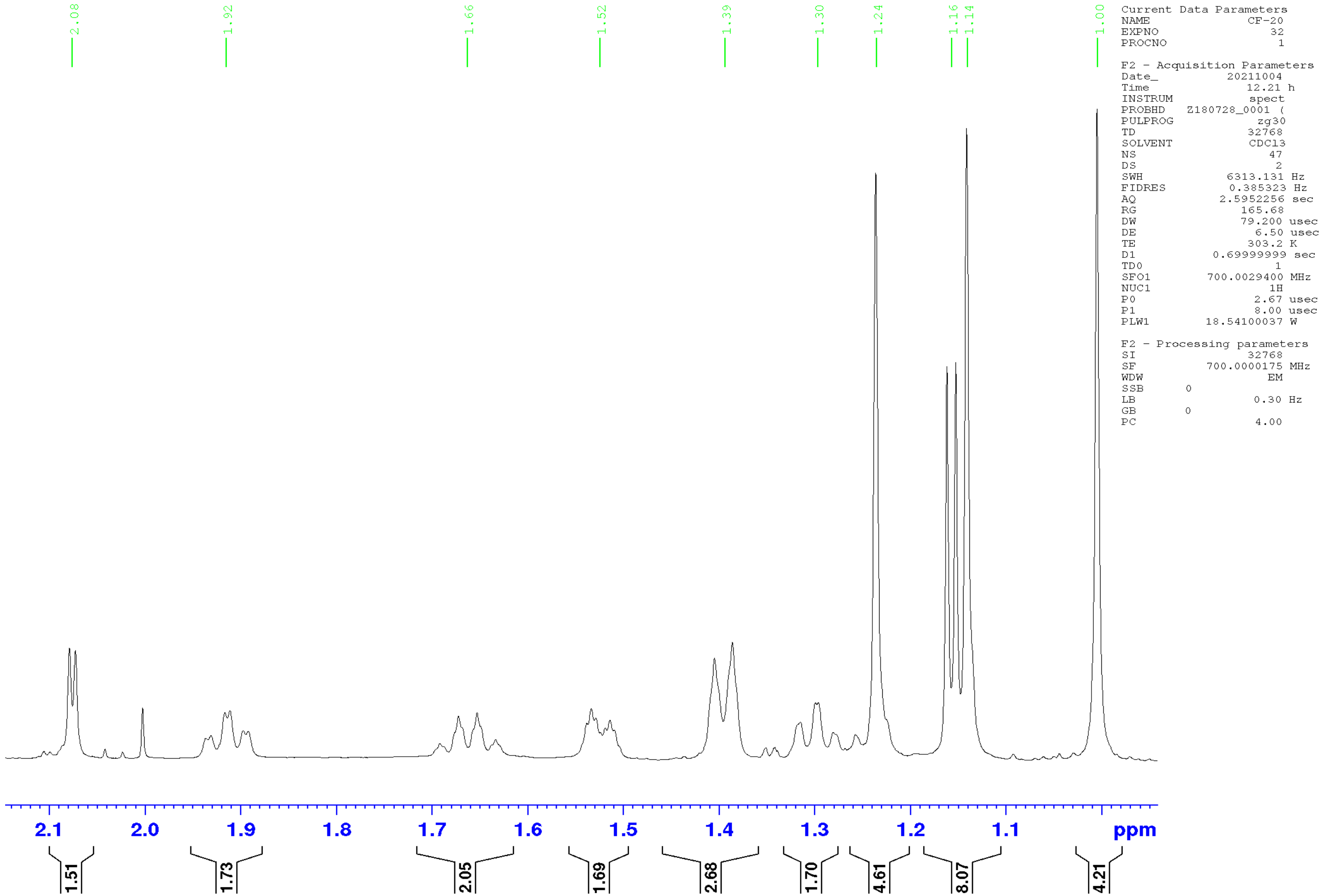

^{1}H NMR spectrum (700 MHz, $CDCl_3$) of **4** (expanded)

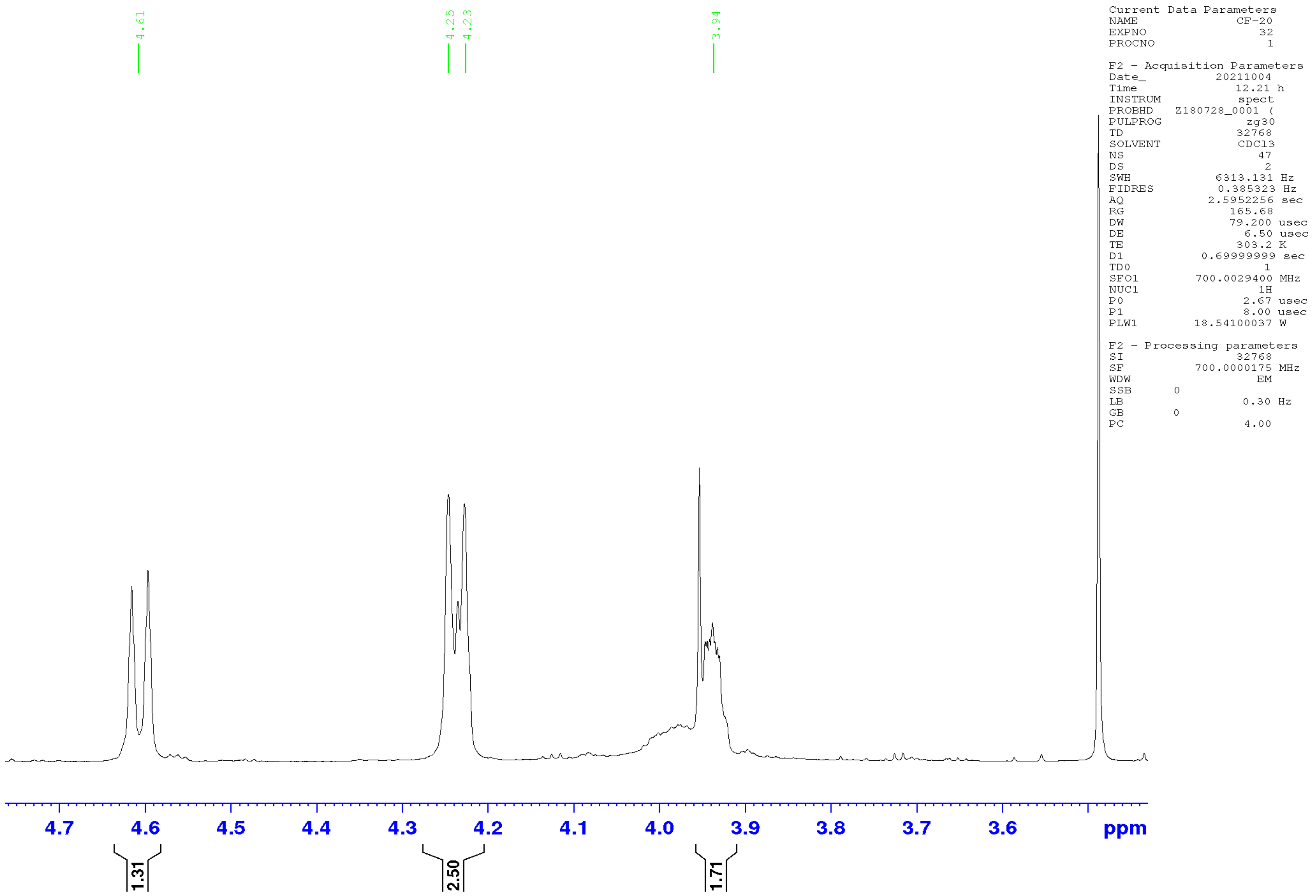

^{1}H NMR spectrum (700 MHz, $CDCl_3$) of **4** (expanded)

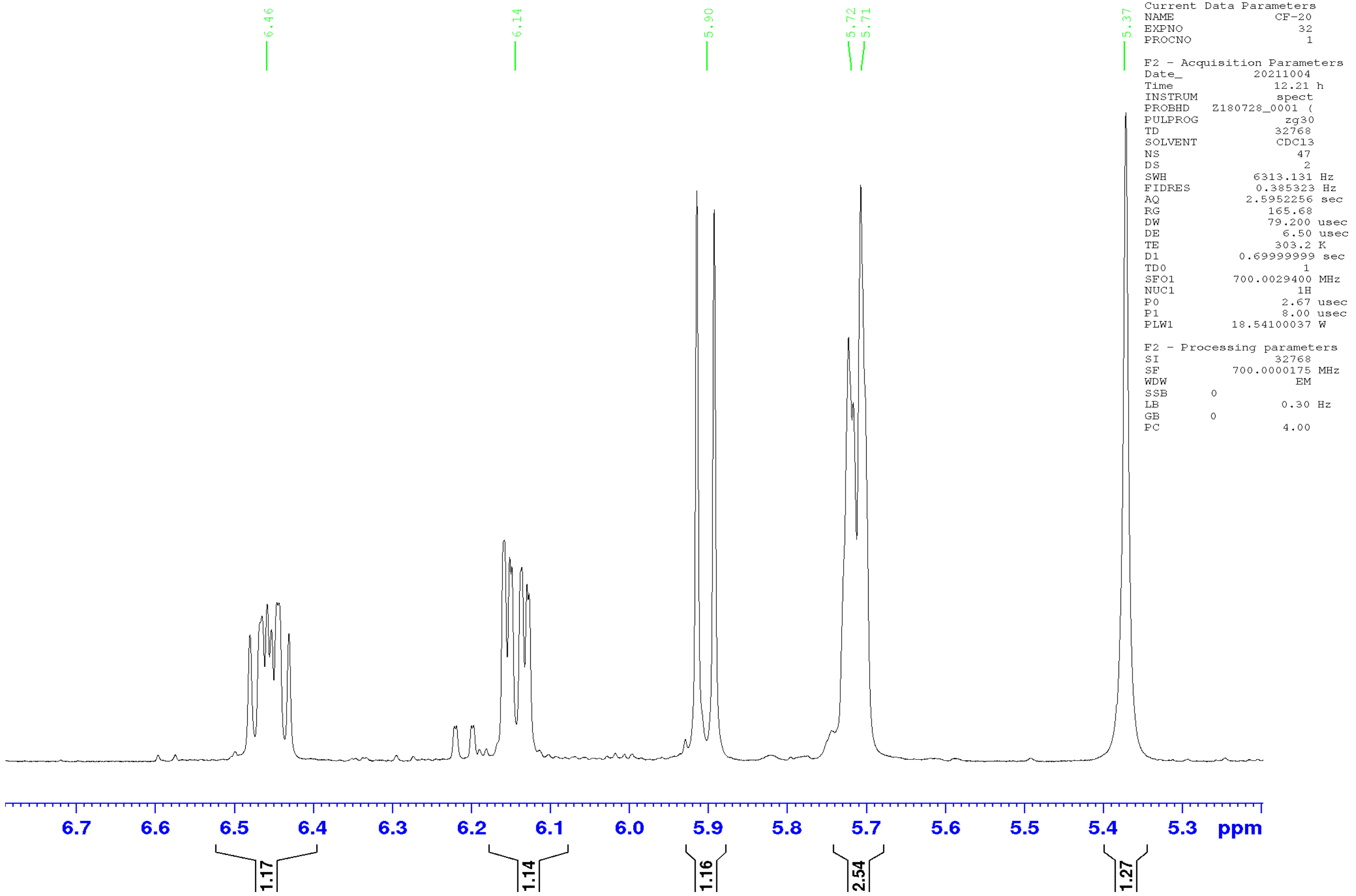

^{1}H NMR spectrum (700 MHz, $CDCl_3$) of **4** (expanded)

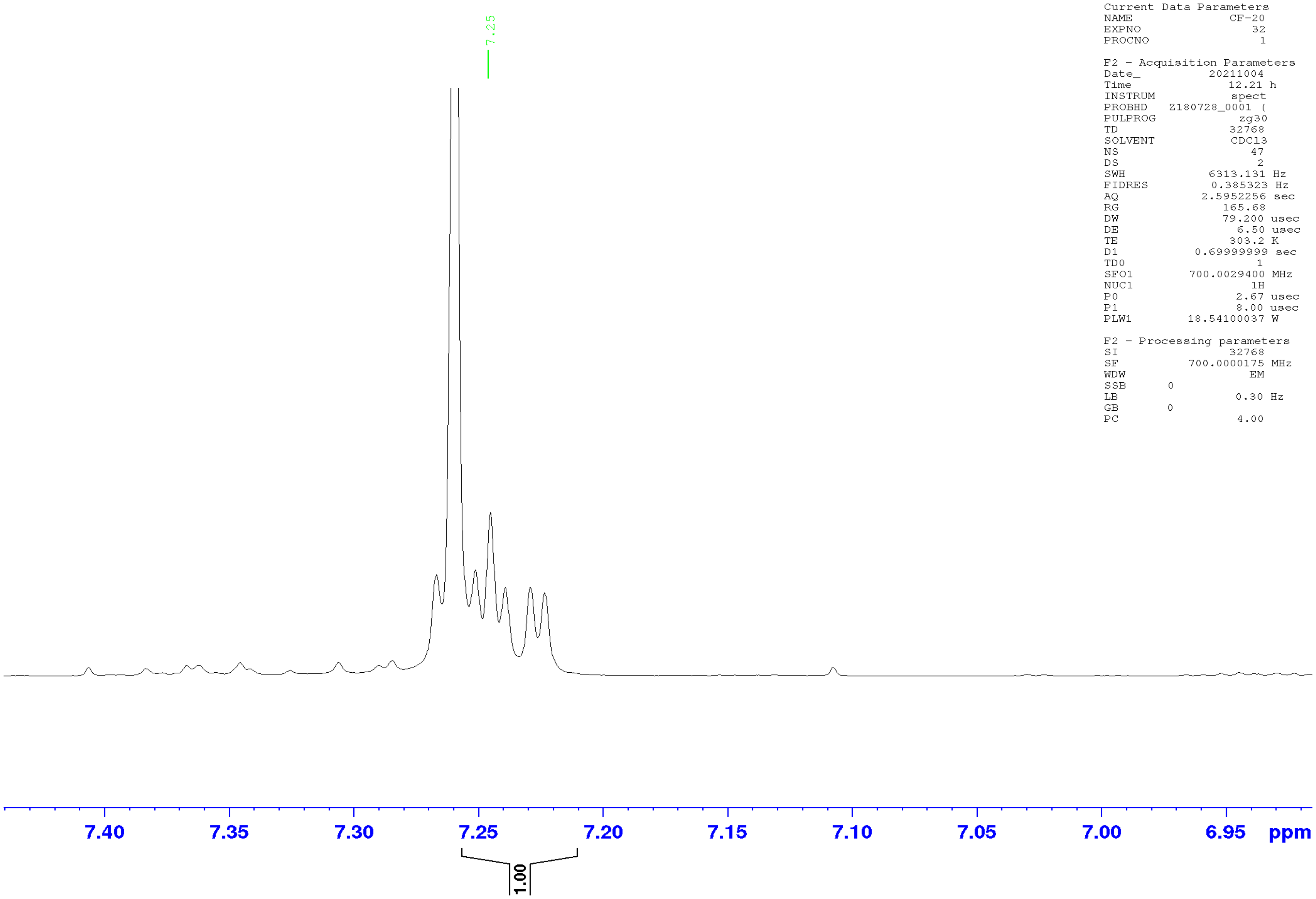

^{1}H NMR spectrum (700 MHz, $CDCl_3$) of **4** (expanded)

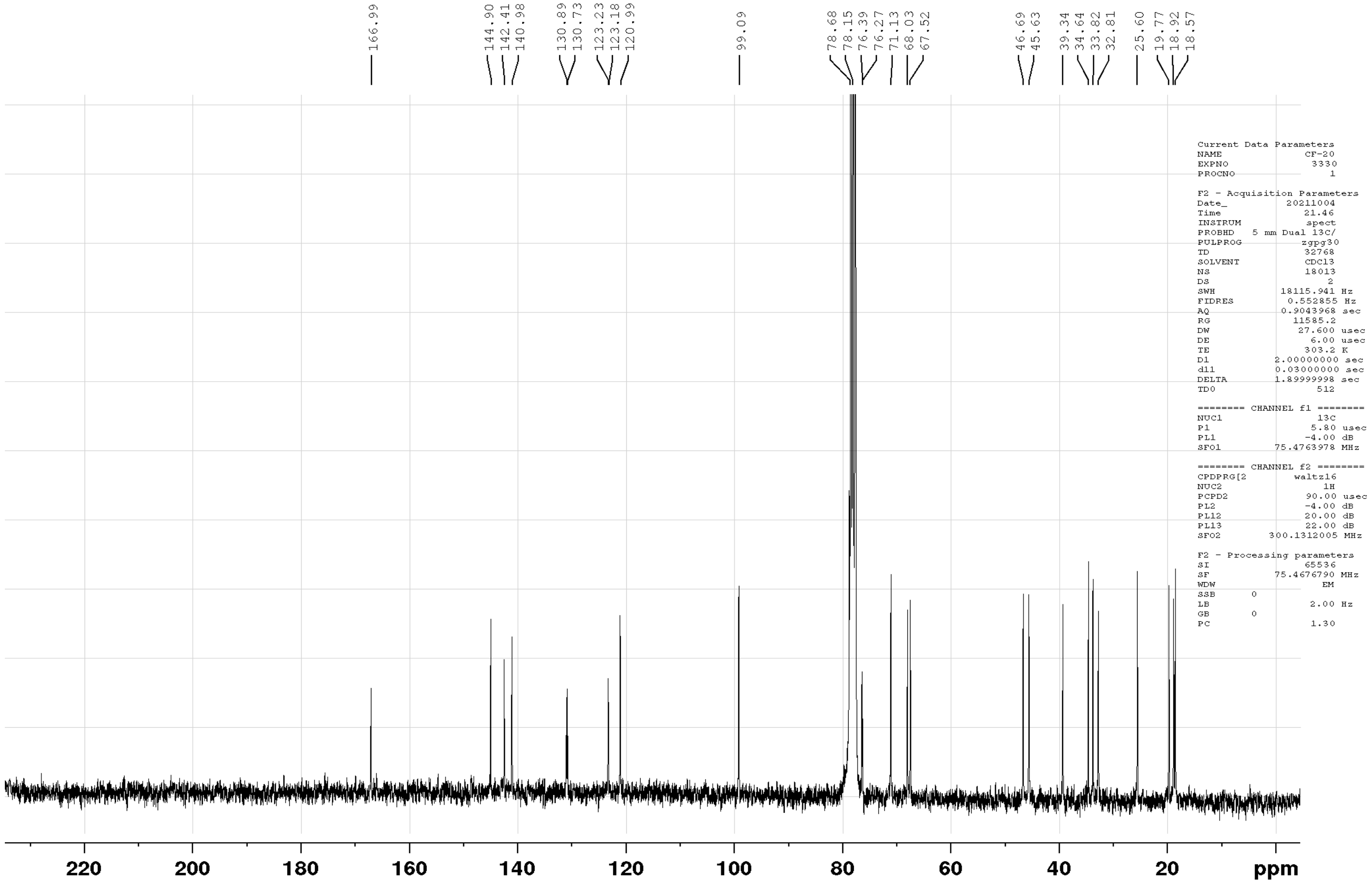

Figure S36. ^{13}C NMR spectrum (75 MHz, $CDCl_3$) of **4**

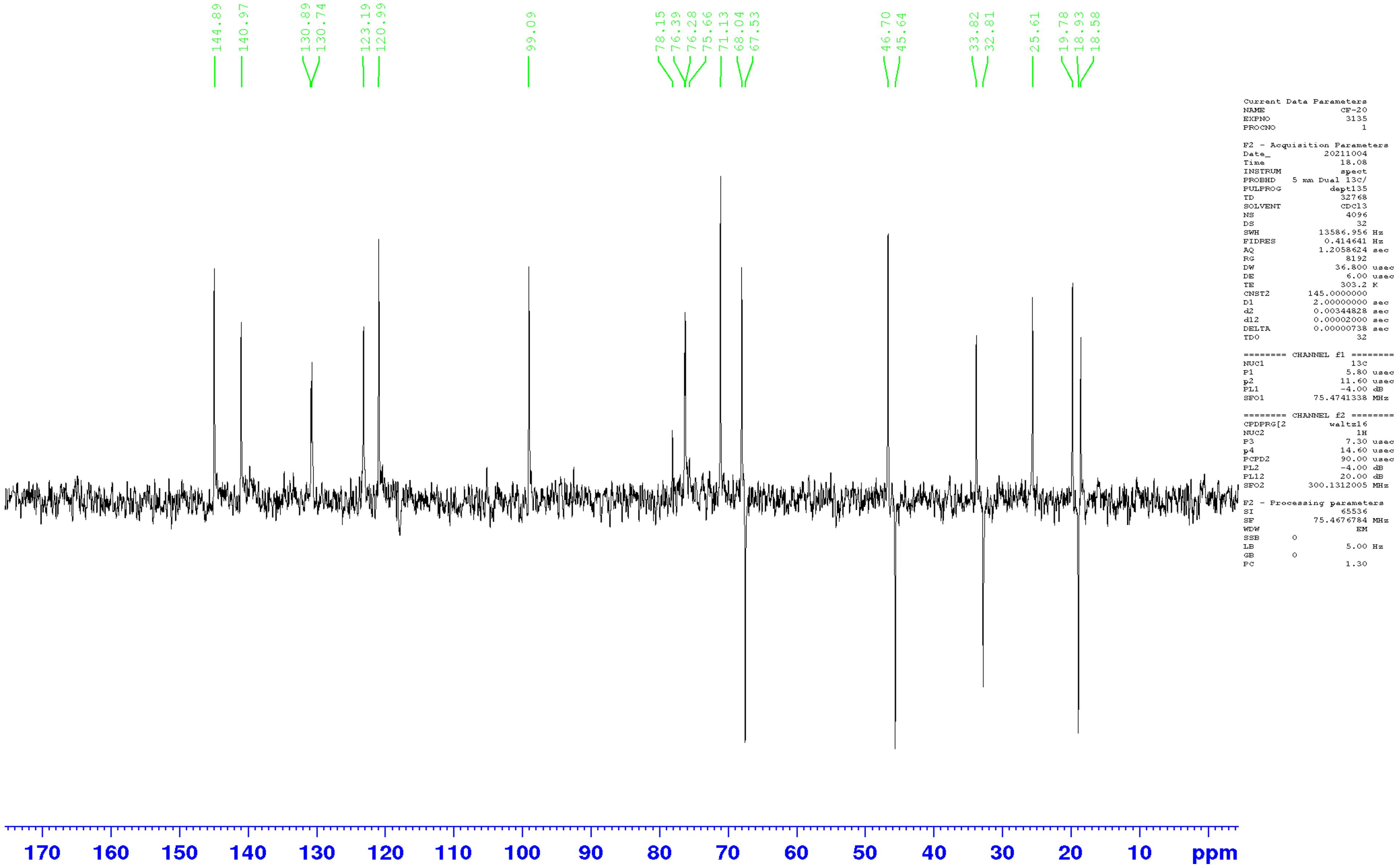

Figure S37. DEPT-135 NMR spectrum (75 MHz, $CDCl_3$) of **4**

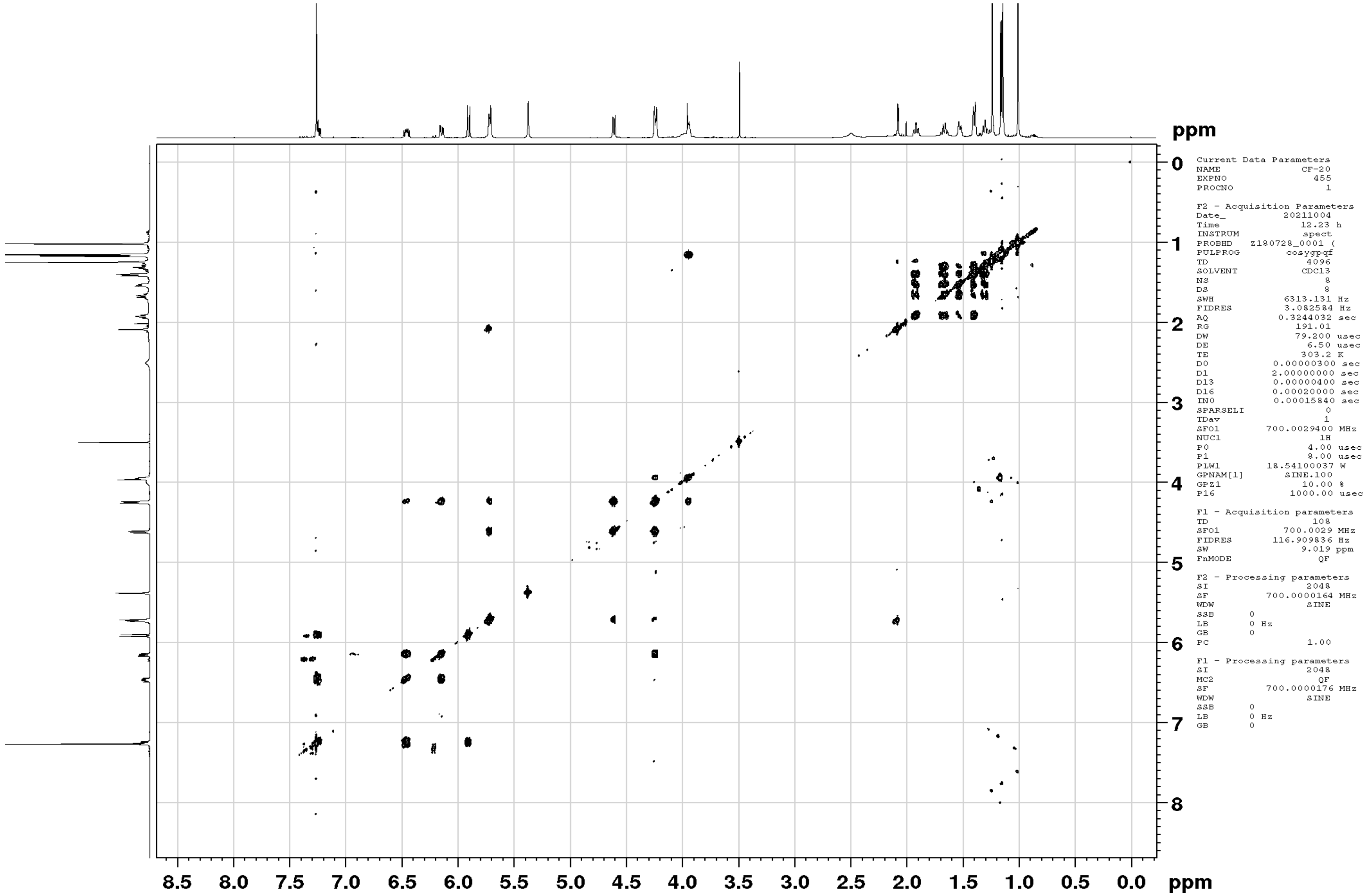

Figure S38. COSY-45 spectrum (700 MHz, $CDCl_3$) of **4**

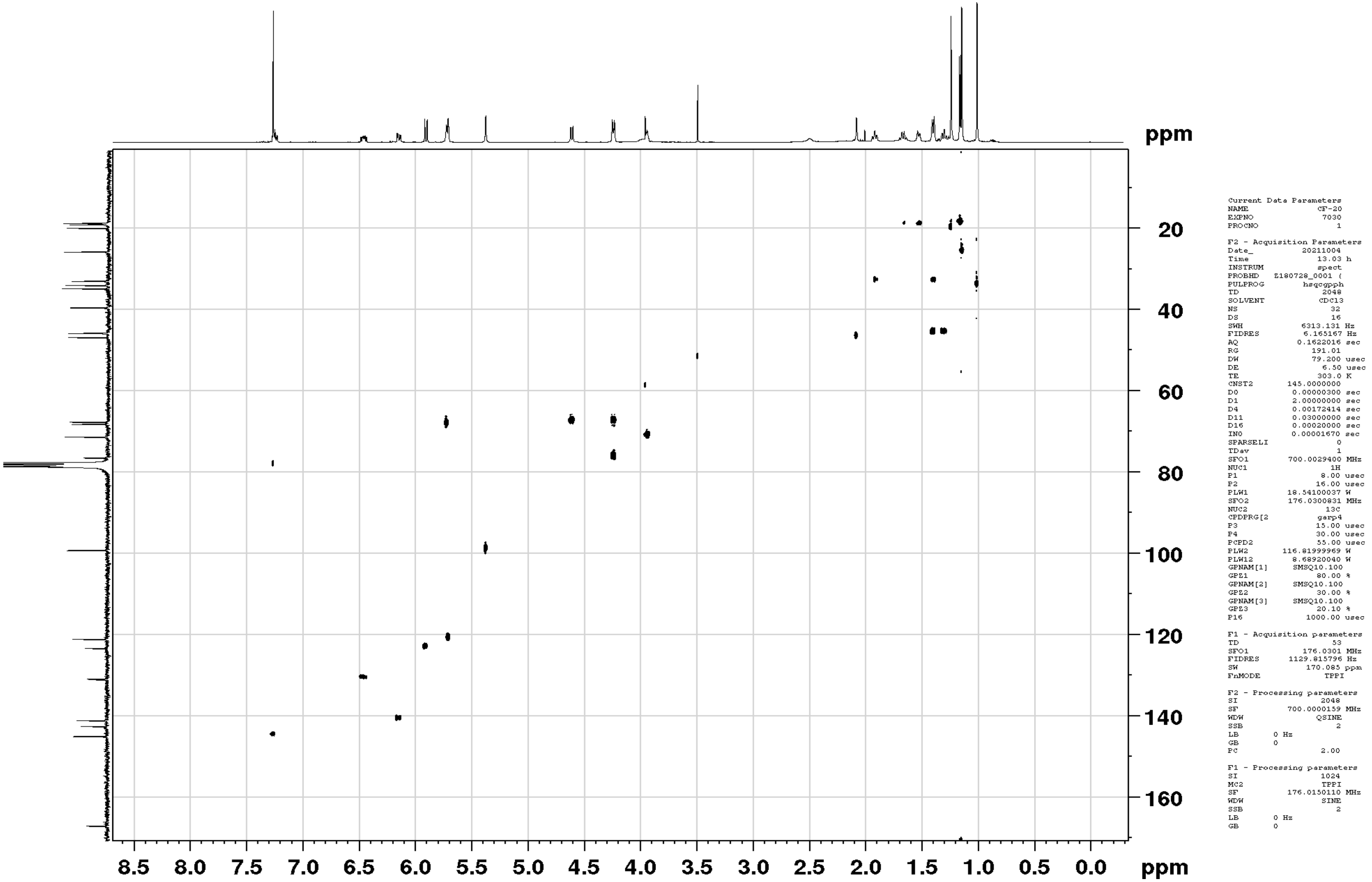

Figure S39. HSQC spectrum (700 MHz, $CDCl_3$) of **4**

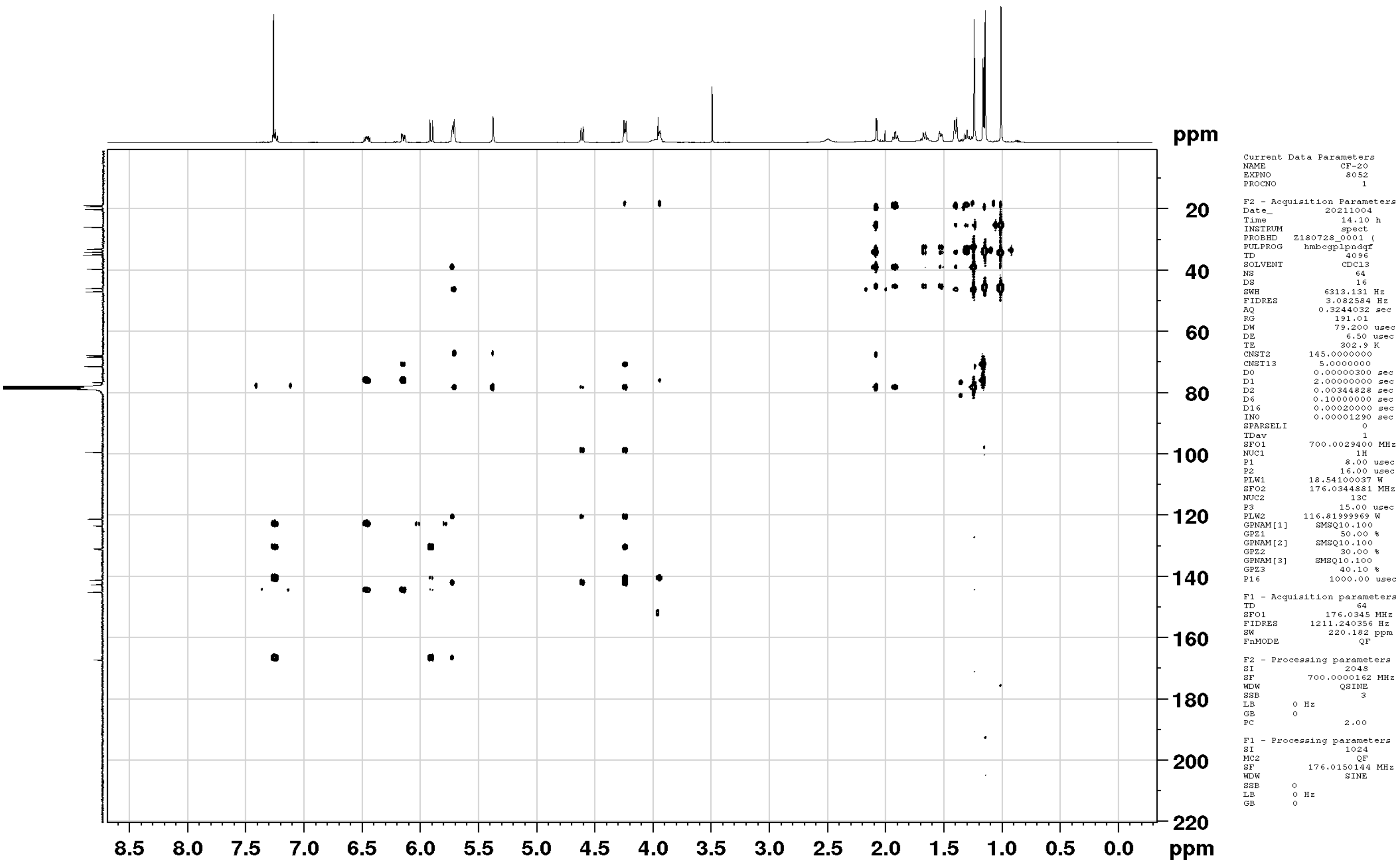

Figure S40. HMBC spectrum (700 MHz, $CDCl_3$) of **4**

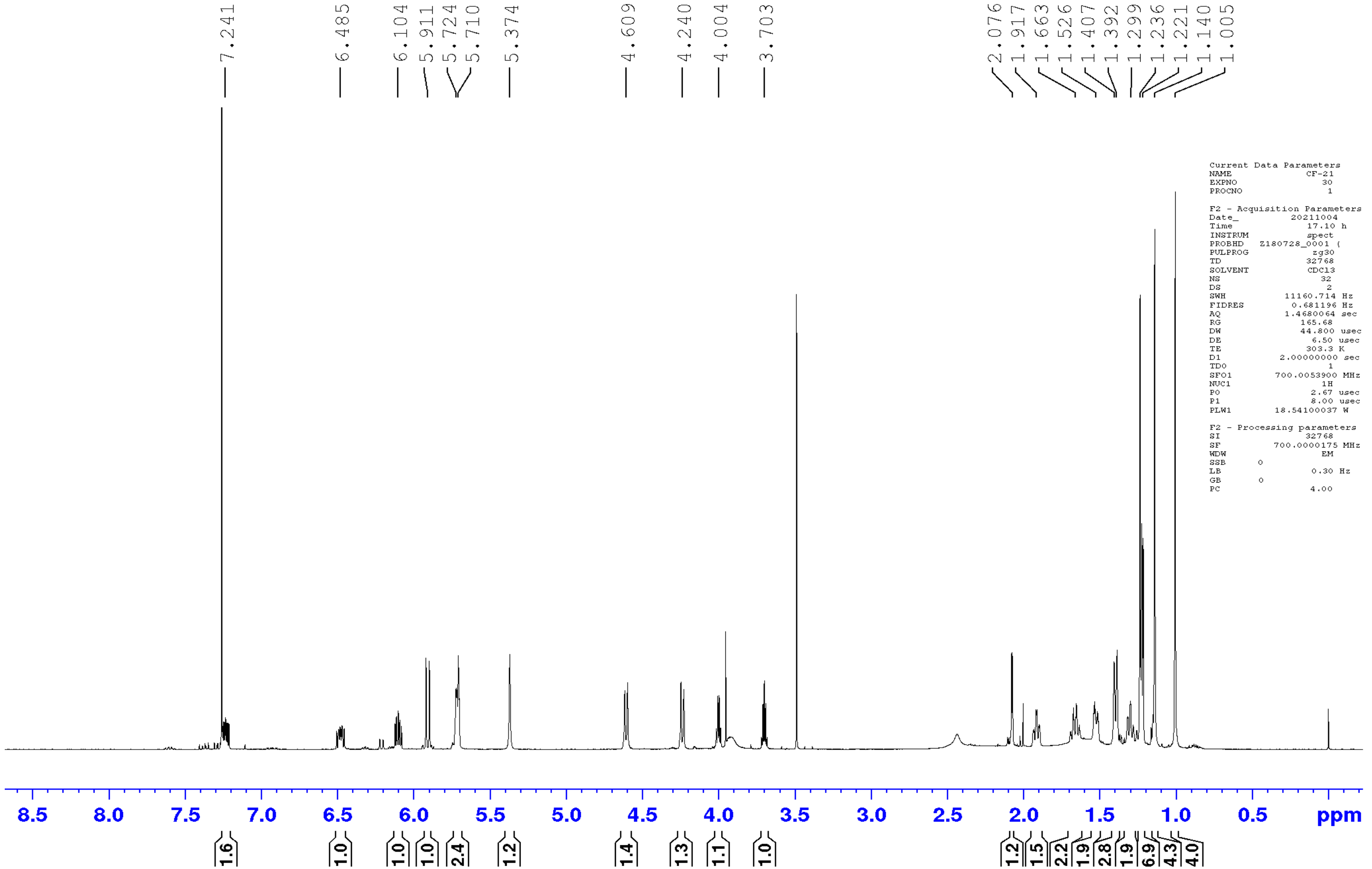

Figure S41. ^{1}H NMR spectrum (700 MHz, $CDCl_3$) of **5**

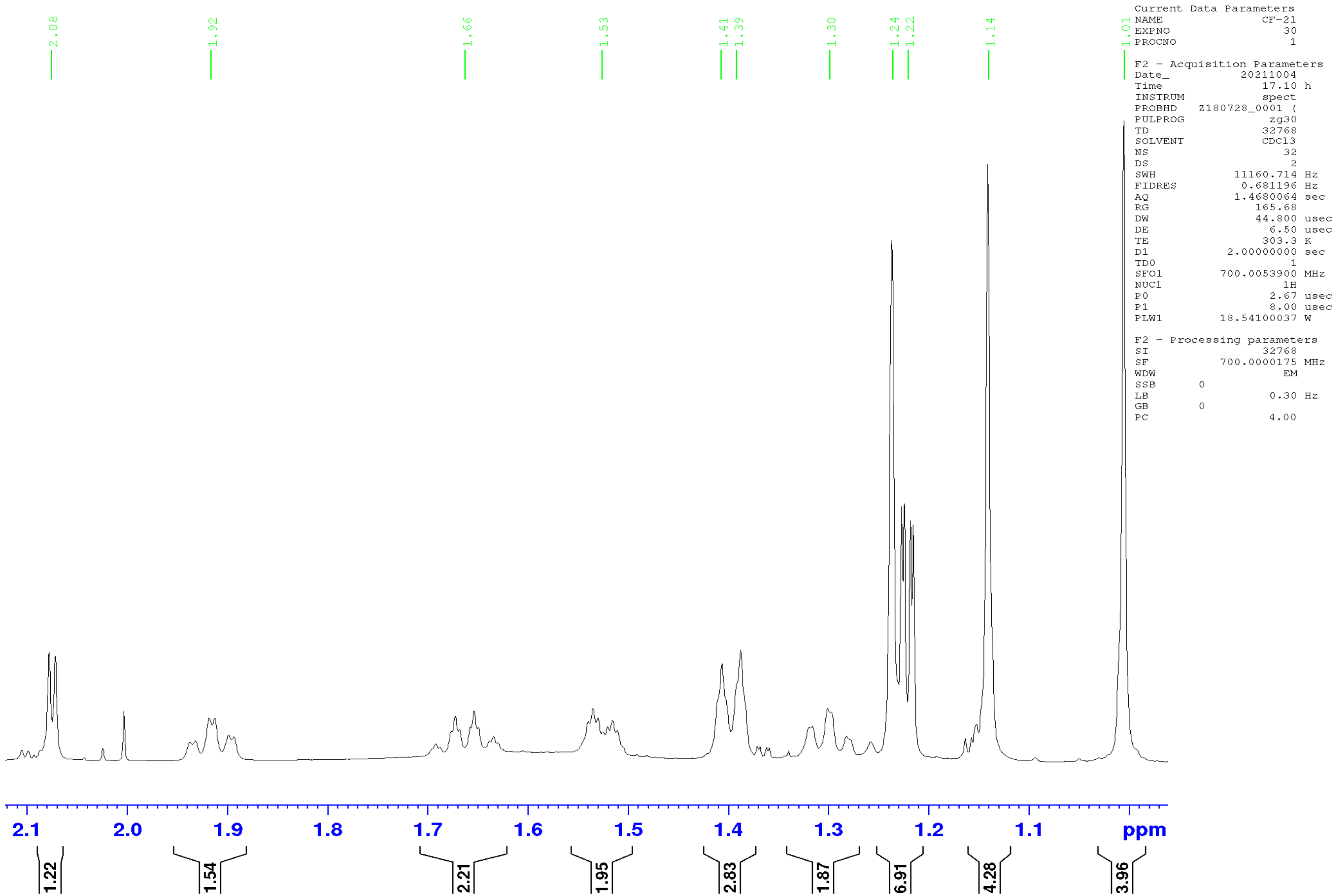

^{1}H NMR spectrum (700 MHz, $CDCl_3$) of **5** (expanded)

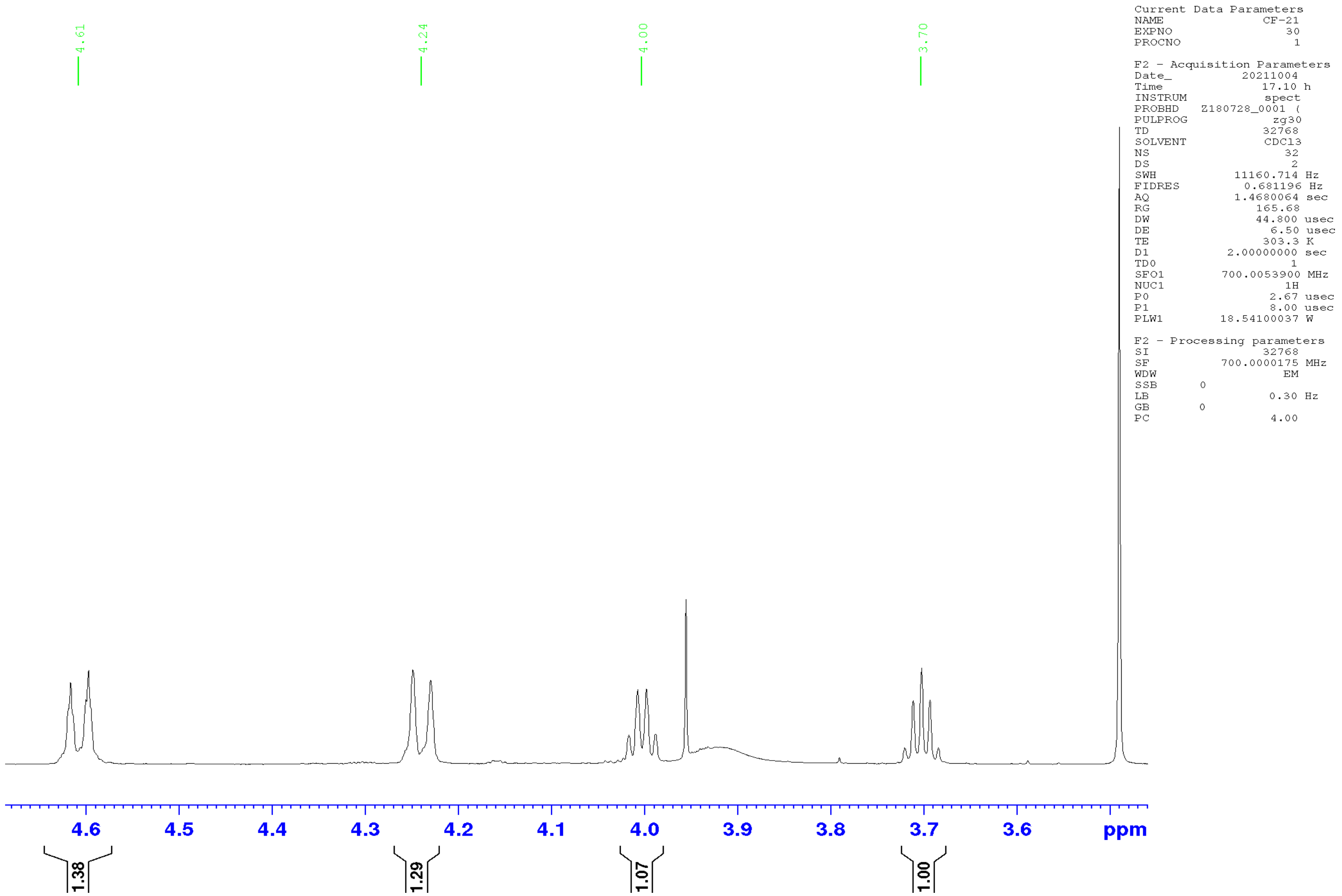

^{1}H NMR spectrum (700 MHz, $CDCl_3$) of **5** (expanded)

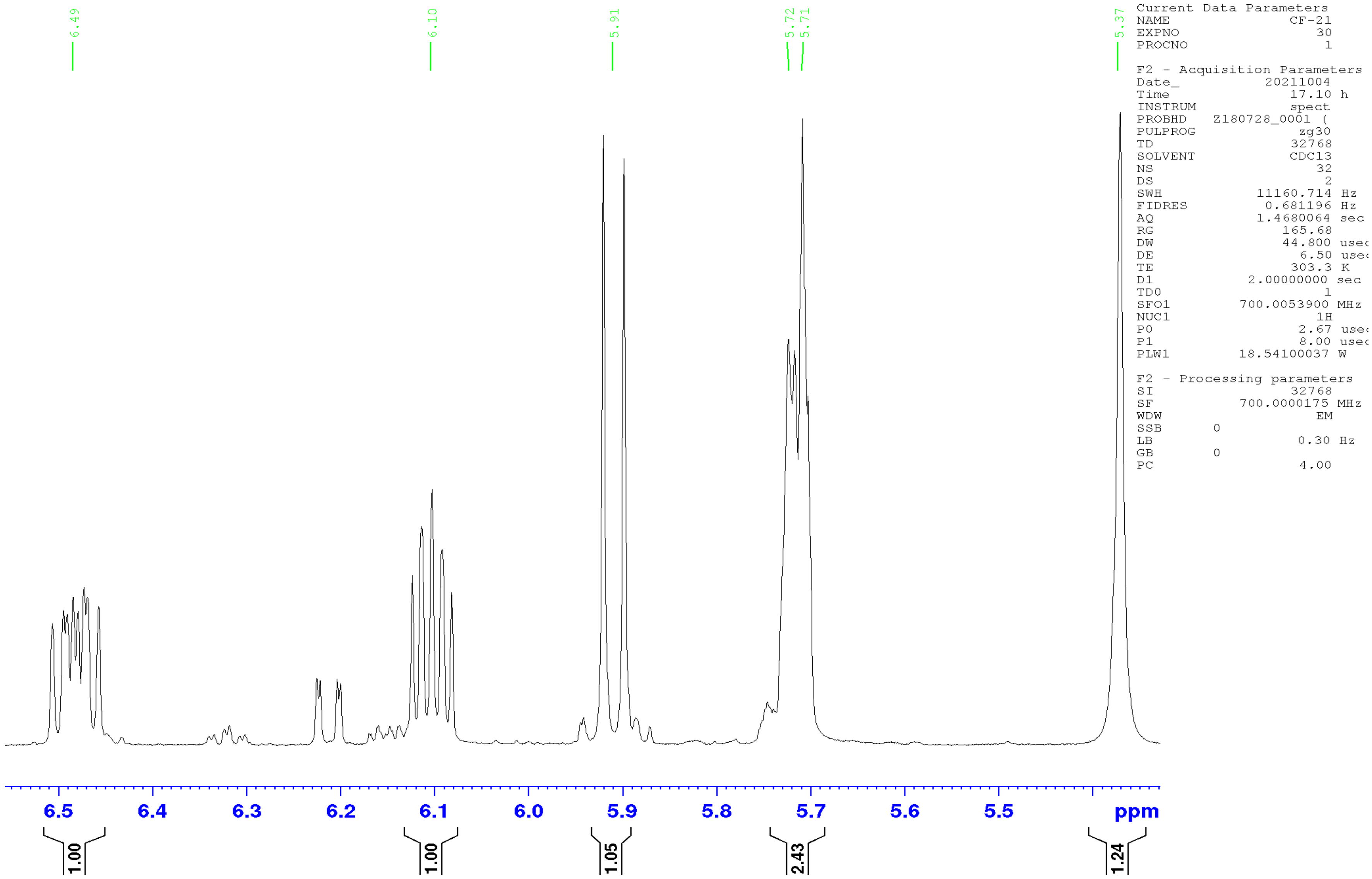

^{1}H NMR spectrum (700 MHz, $CDCl_3$) of **5** (expanded)

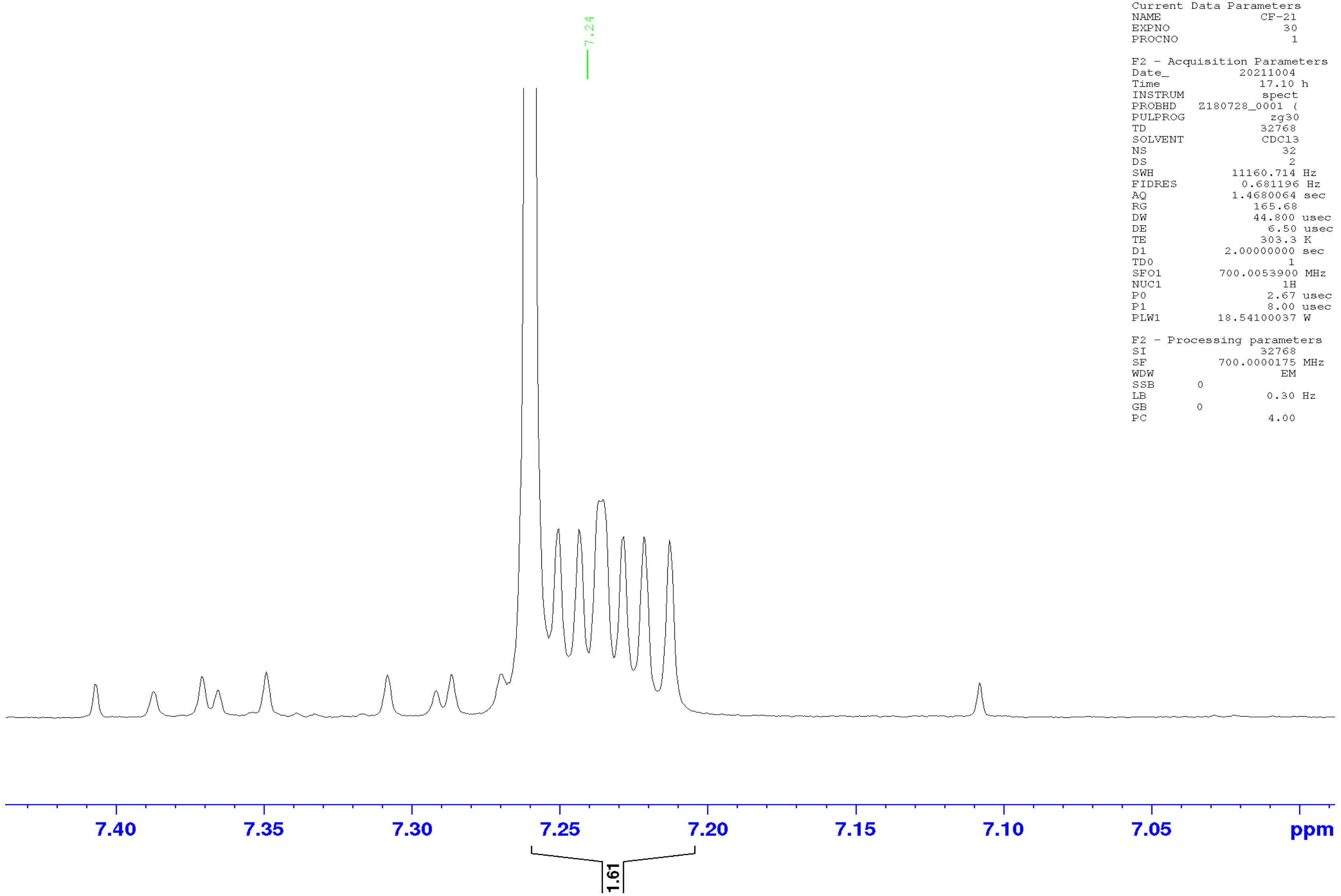

^{1}H NMR spectrum (700 MHz, $CDCl_3$) of **5** (expanded)

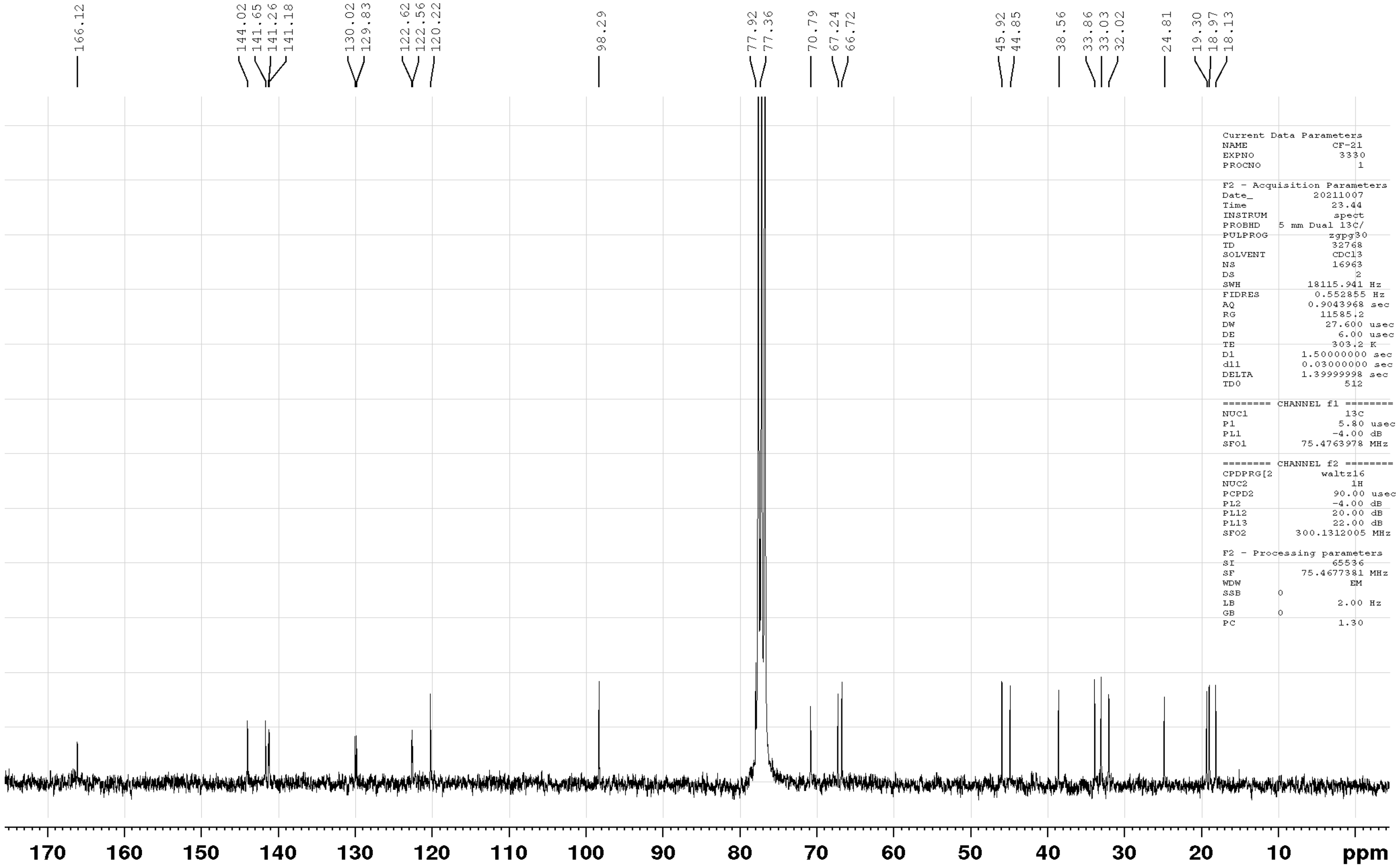

Figure S42. ^{13}C NMR spectrum (75 MHz, $CDCl_3$) of **5**

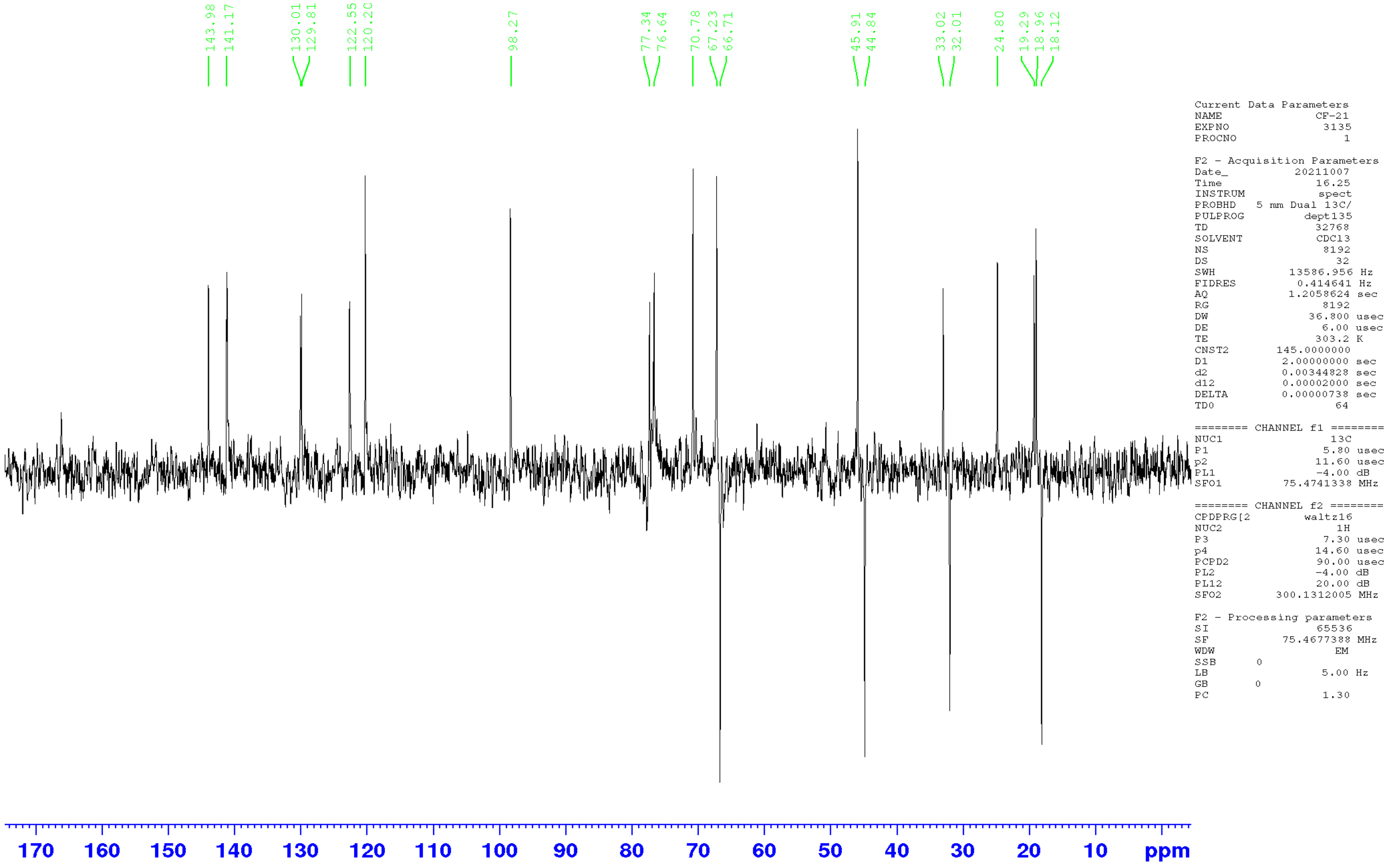

Figure S43. DEPT-135 NMR spectrum (75 MHz, $CDCl_3$) of **5**

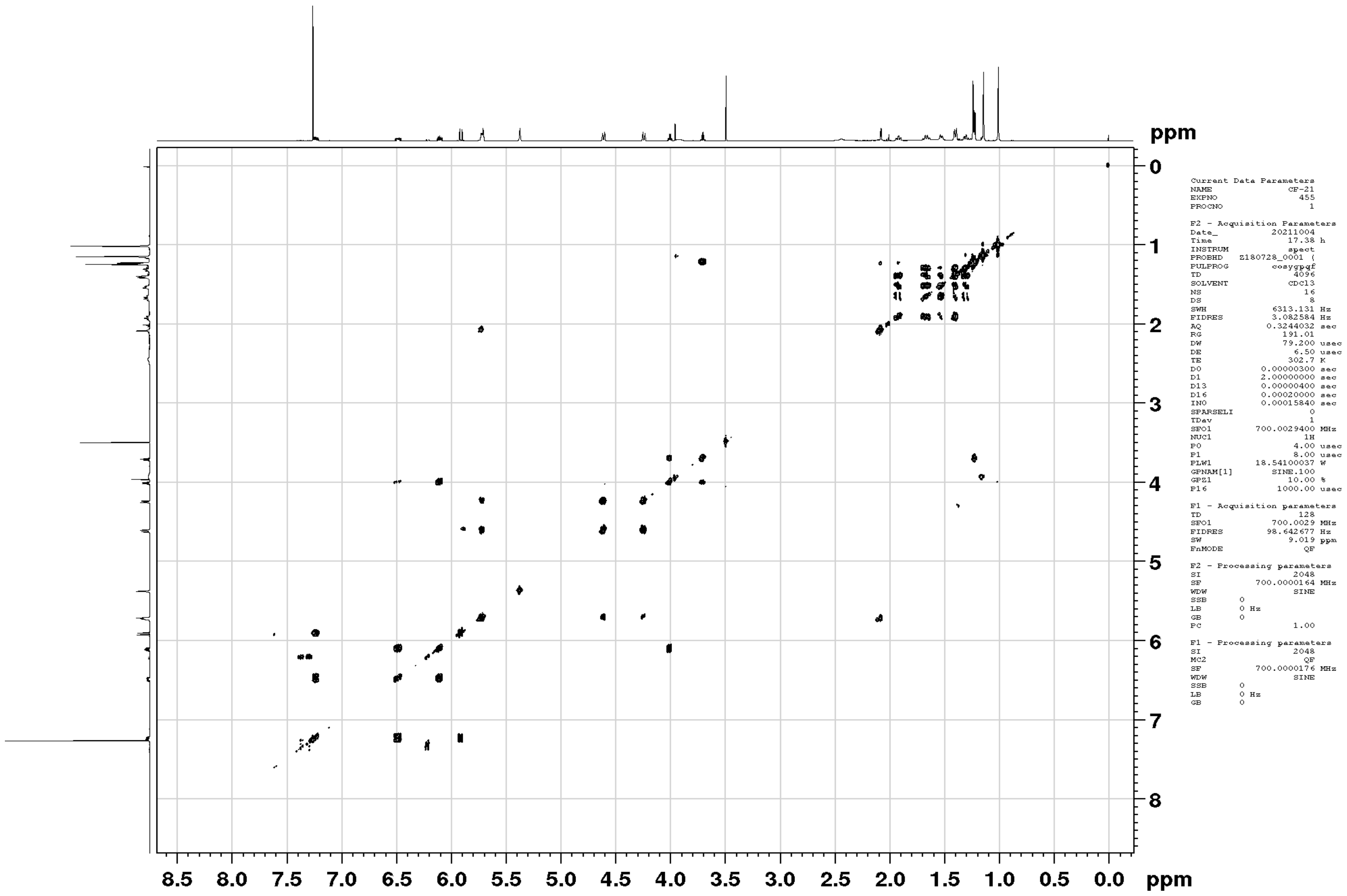

Figure S44. COSY-45 spectrum (700 MHz, $CDCl_3$) of **5**

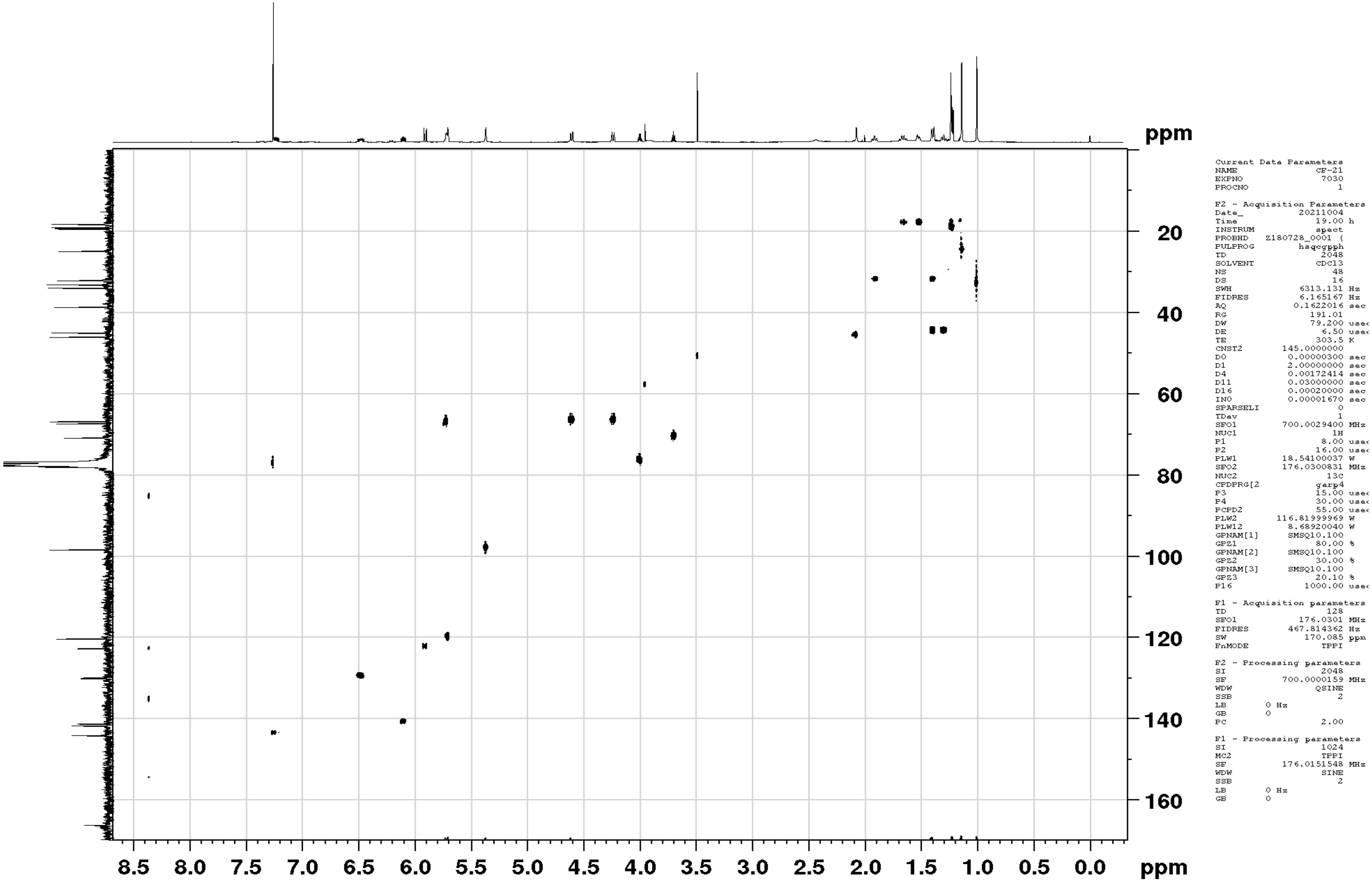

Figure S45. HSQC spectrum (700 MHz, $CDCl_3$) of **5**

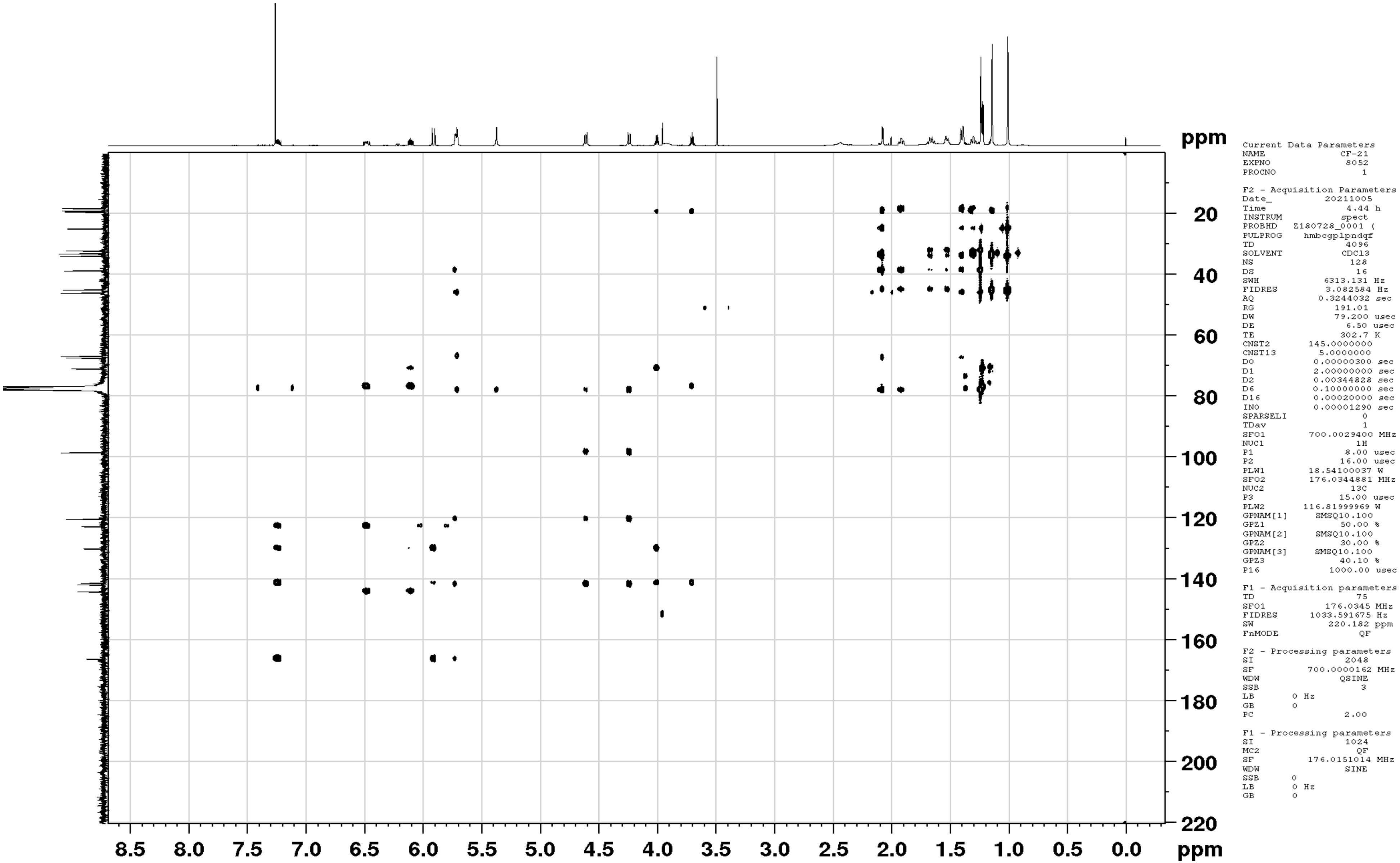

Figure S46. HMBC spectrum (700 MHz, $CDCl_3$) of **5**

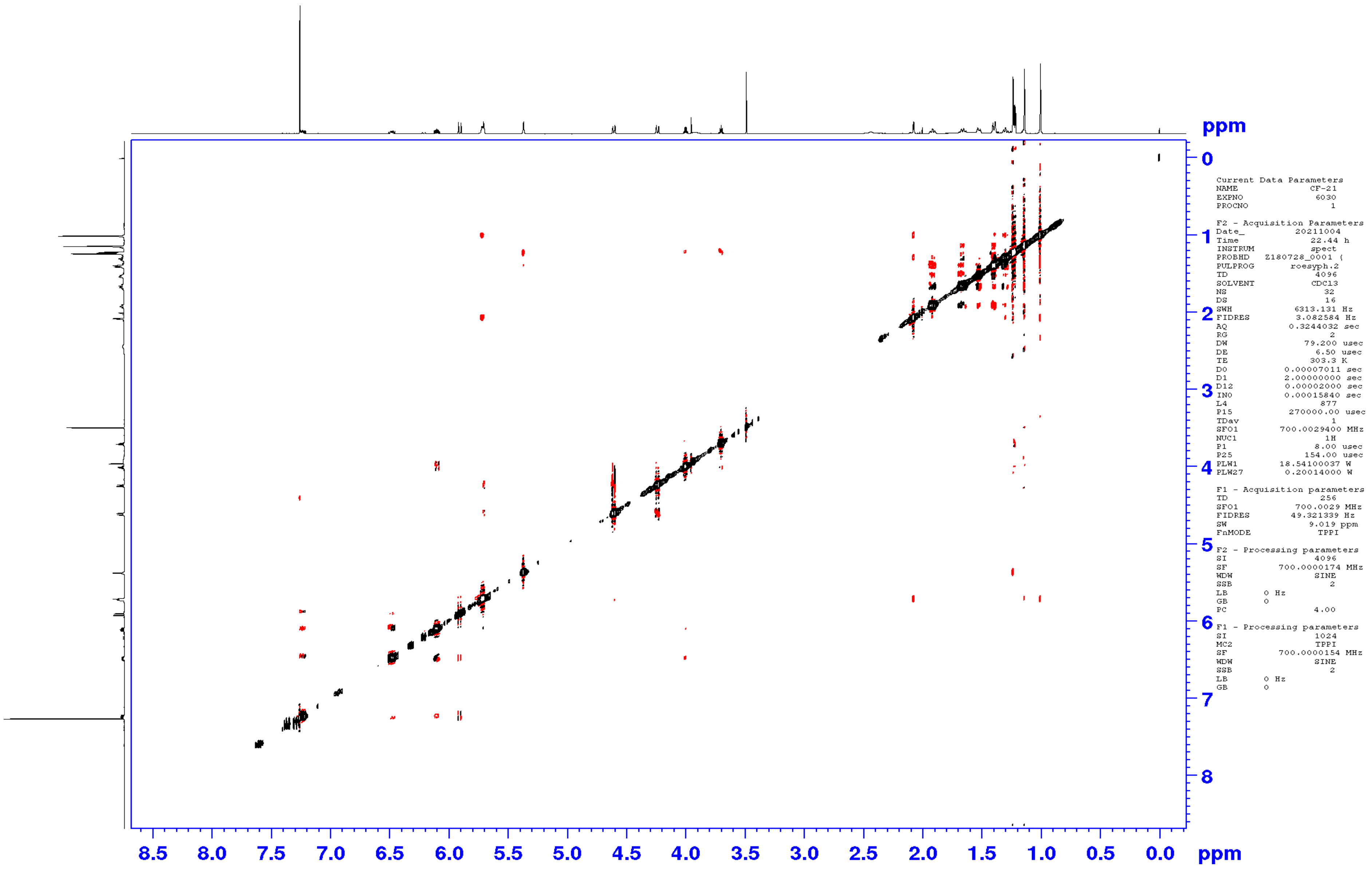

Figure S47. ROESY spectrum (700 MHz, $CDCl_3$) of **5**

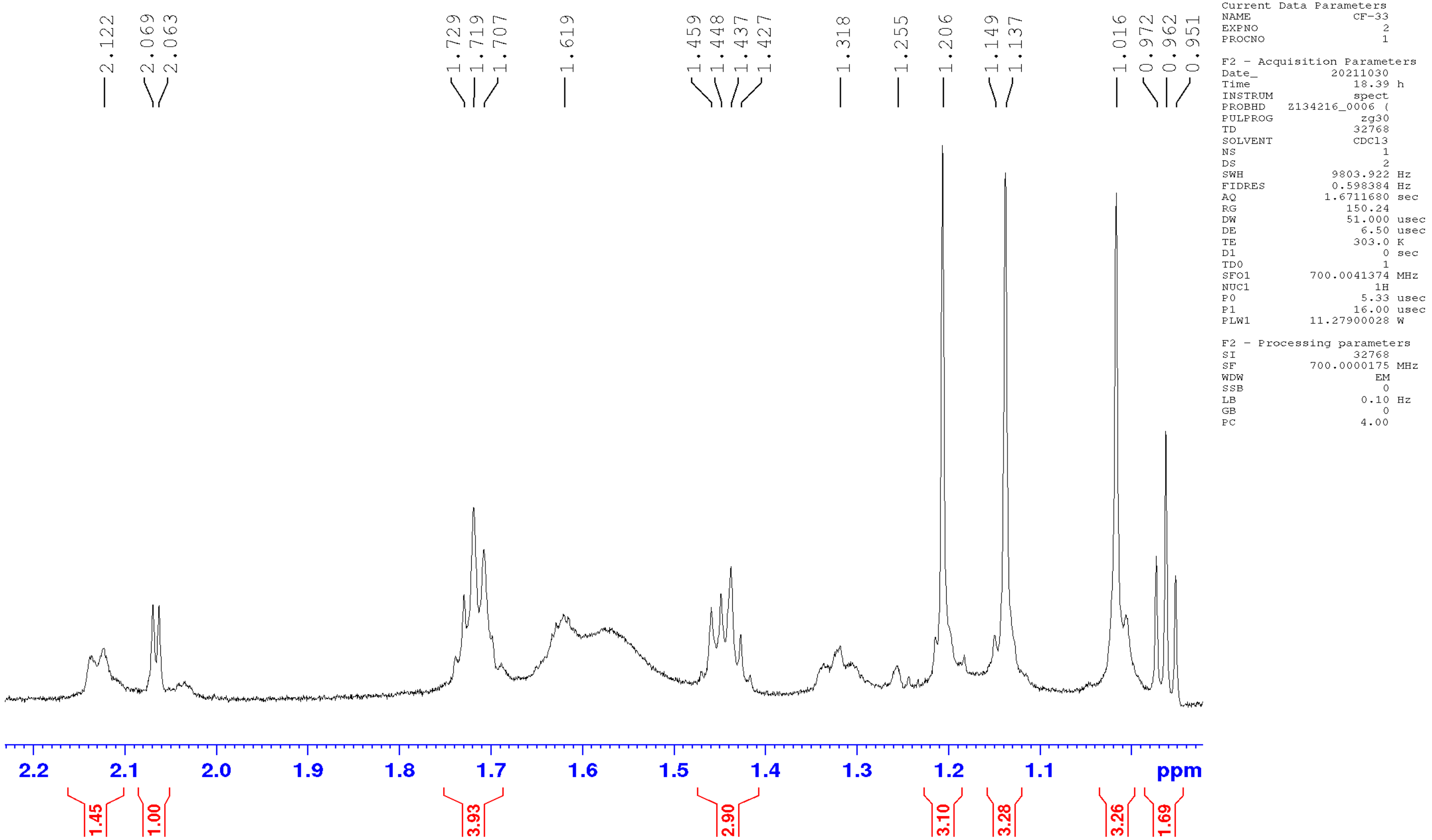

Figure S48. Expanded ^{1}H NMR spectrum (700 MHz, $CDCl_3$) of **9**

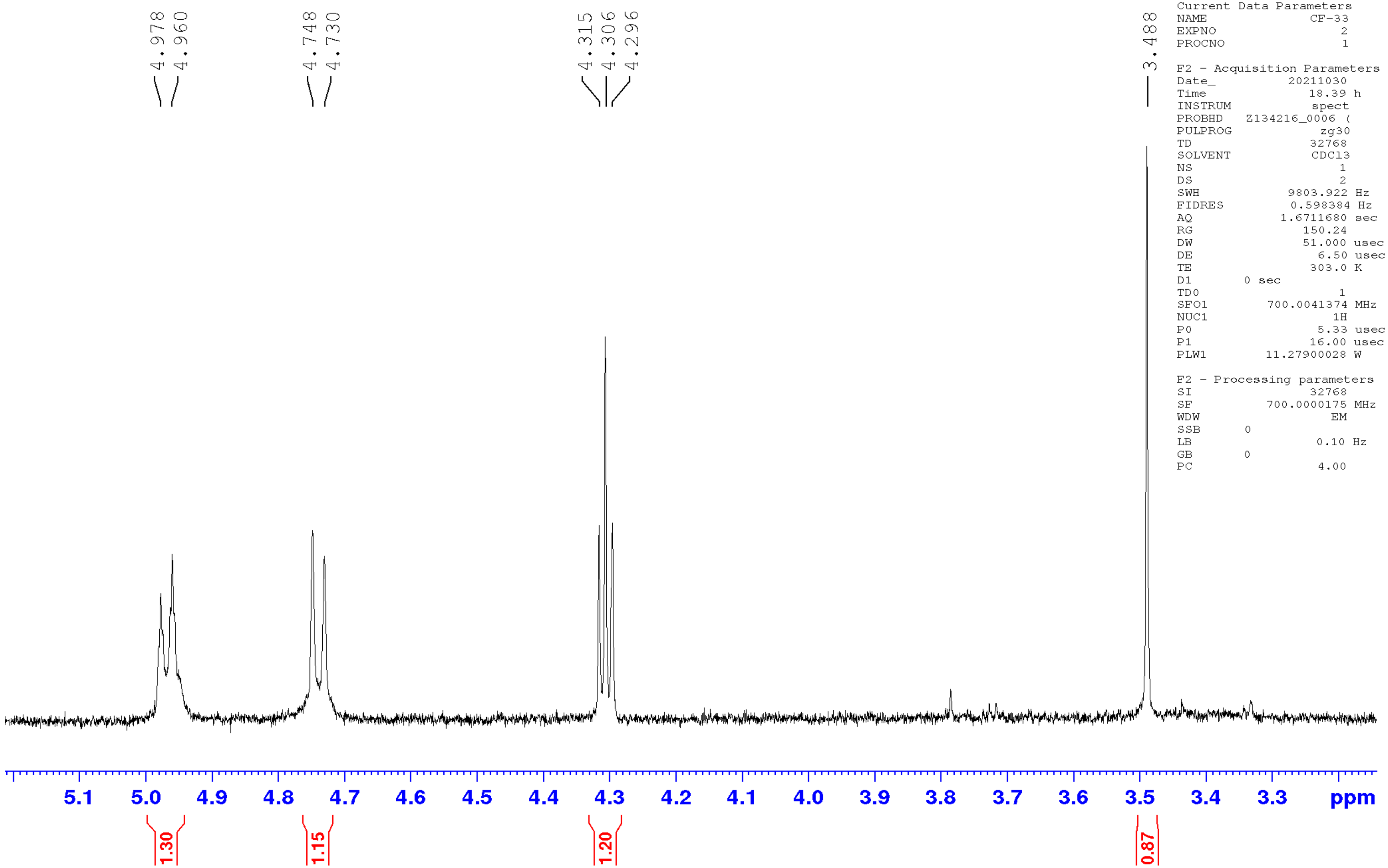

Expanded ^{1}H NMR spectrum (700 MHz, $CDCl_3$) of **9**

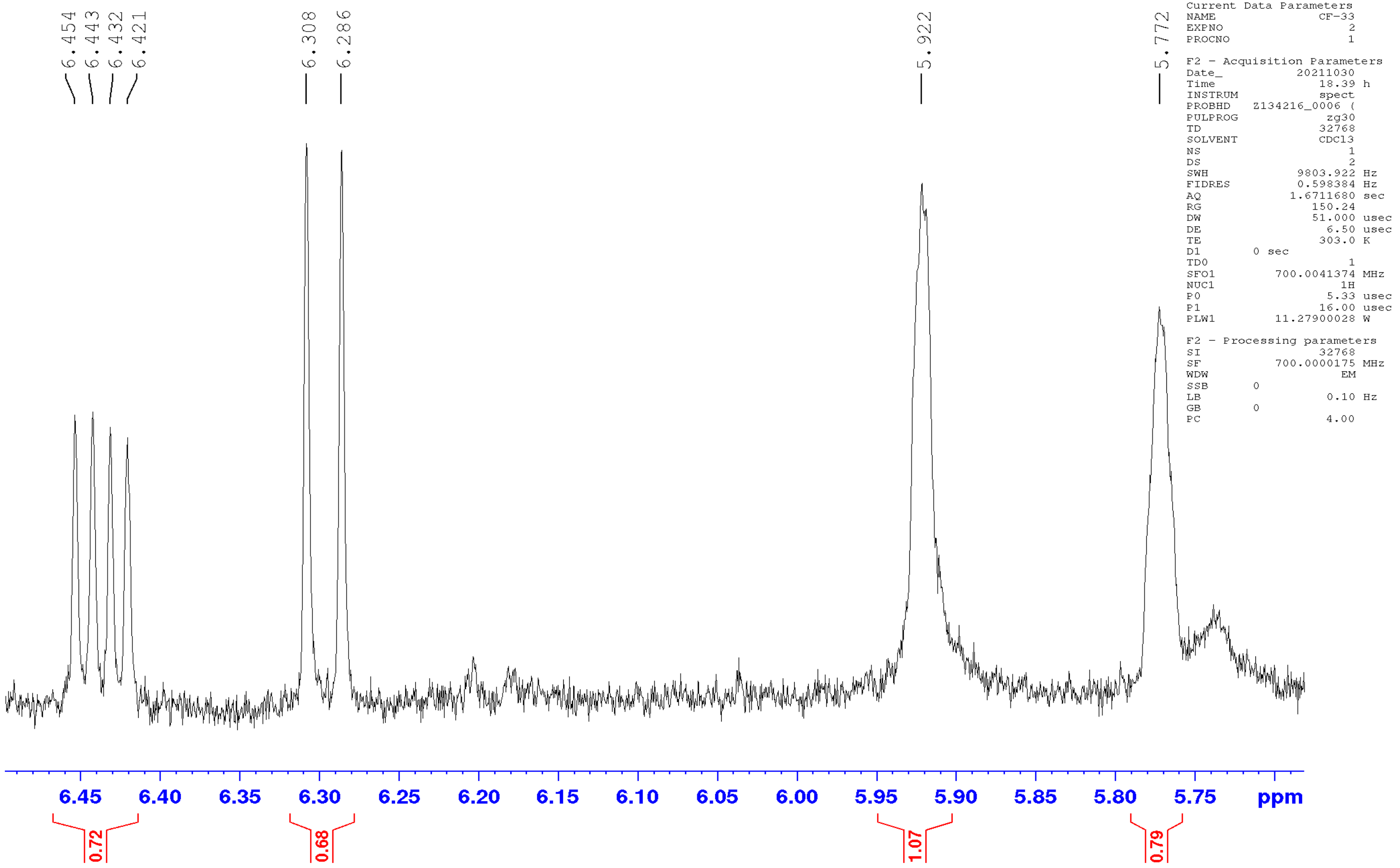

Expanded ^{1}H NMR spectrum (700 MHz, $CDCl_3$) of **9**

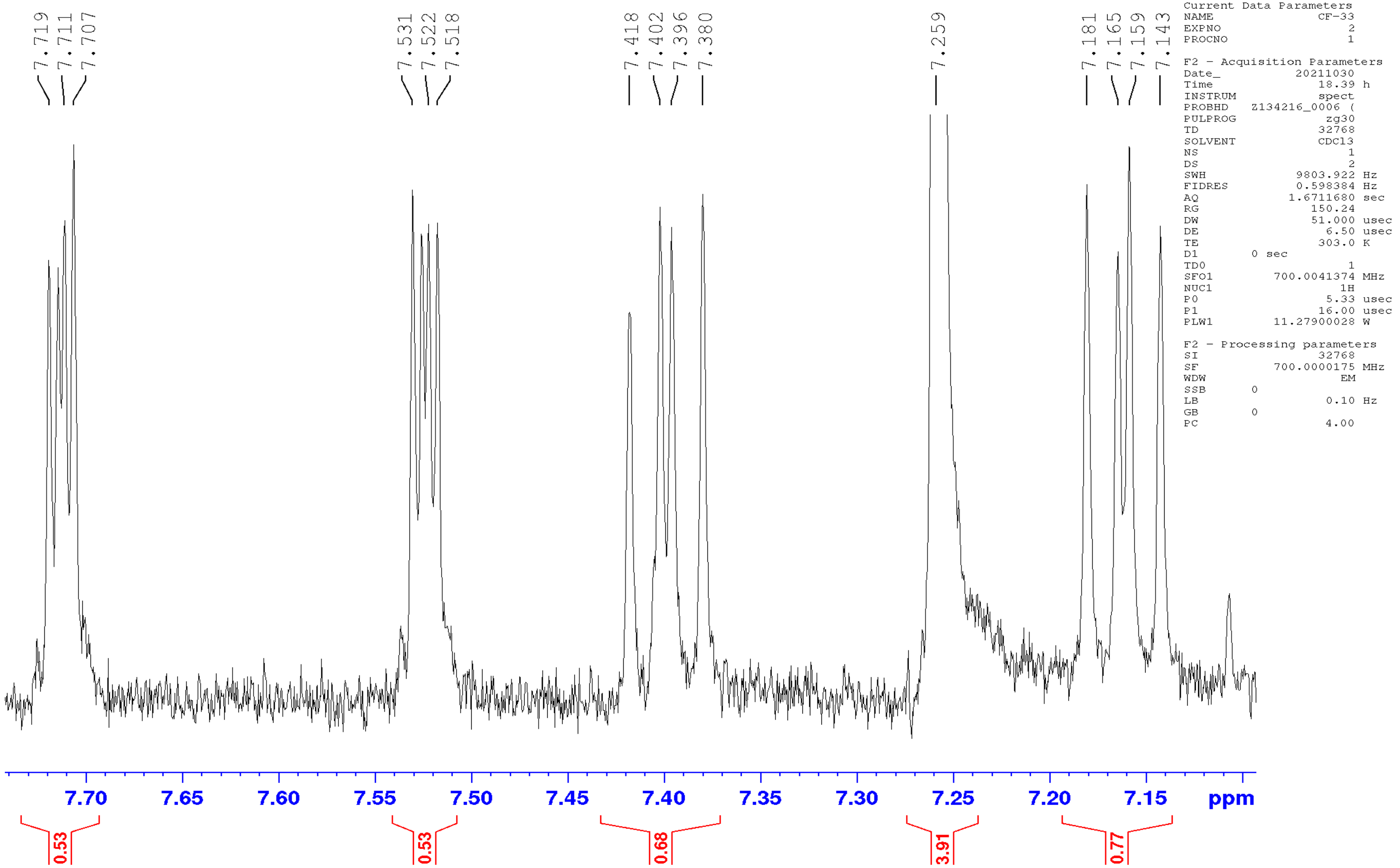

Expanded ^{1}H NMR spectrum (700 MHz, $CDCl_3$) of **9**

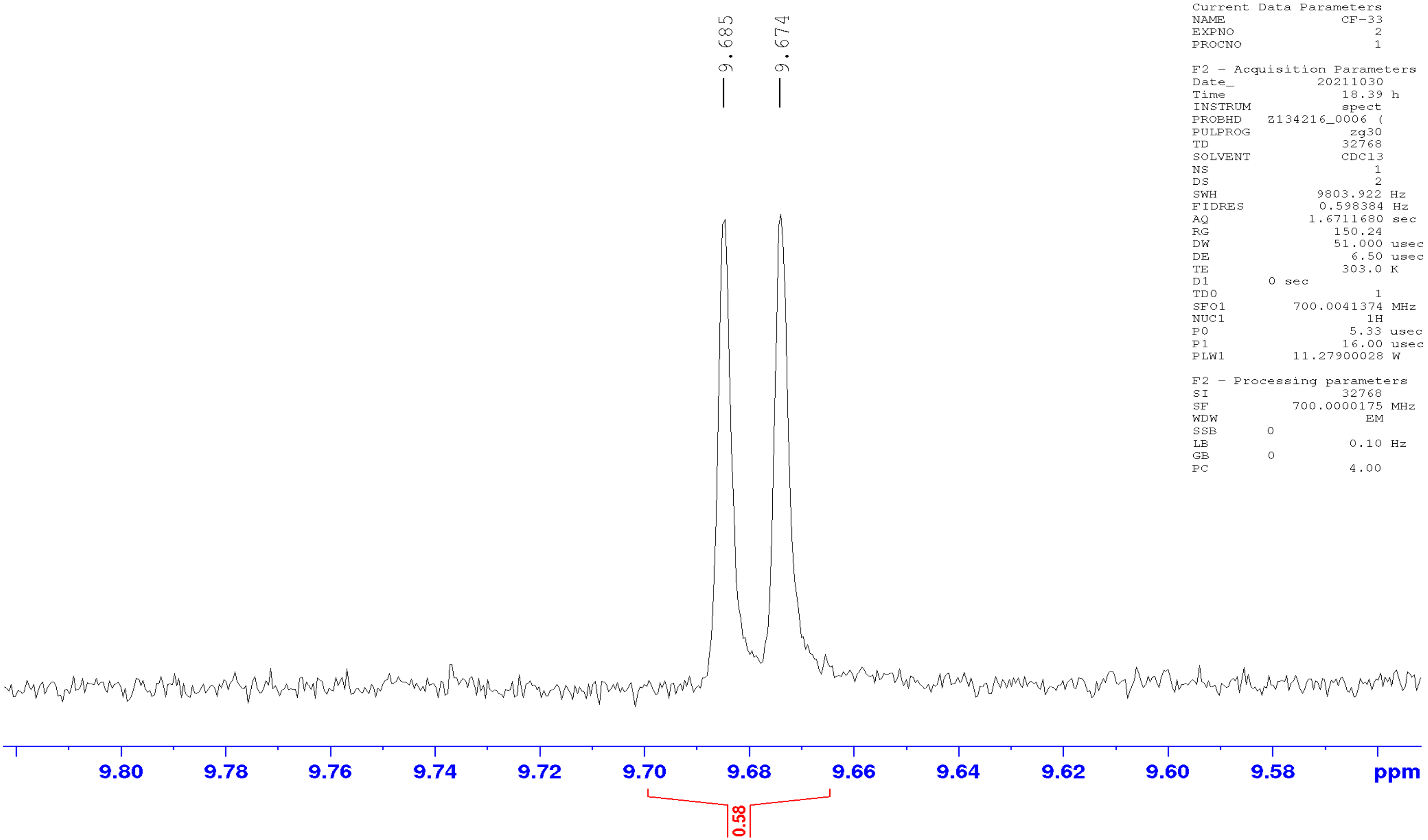

Expanded ^{1}H NMR spectrum (700 MHz, $CDCl_3$) of **9**

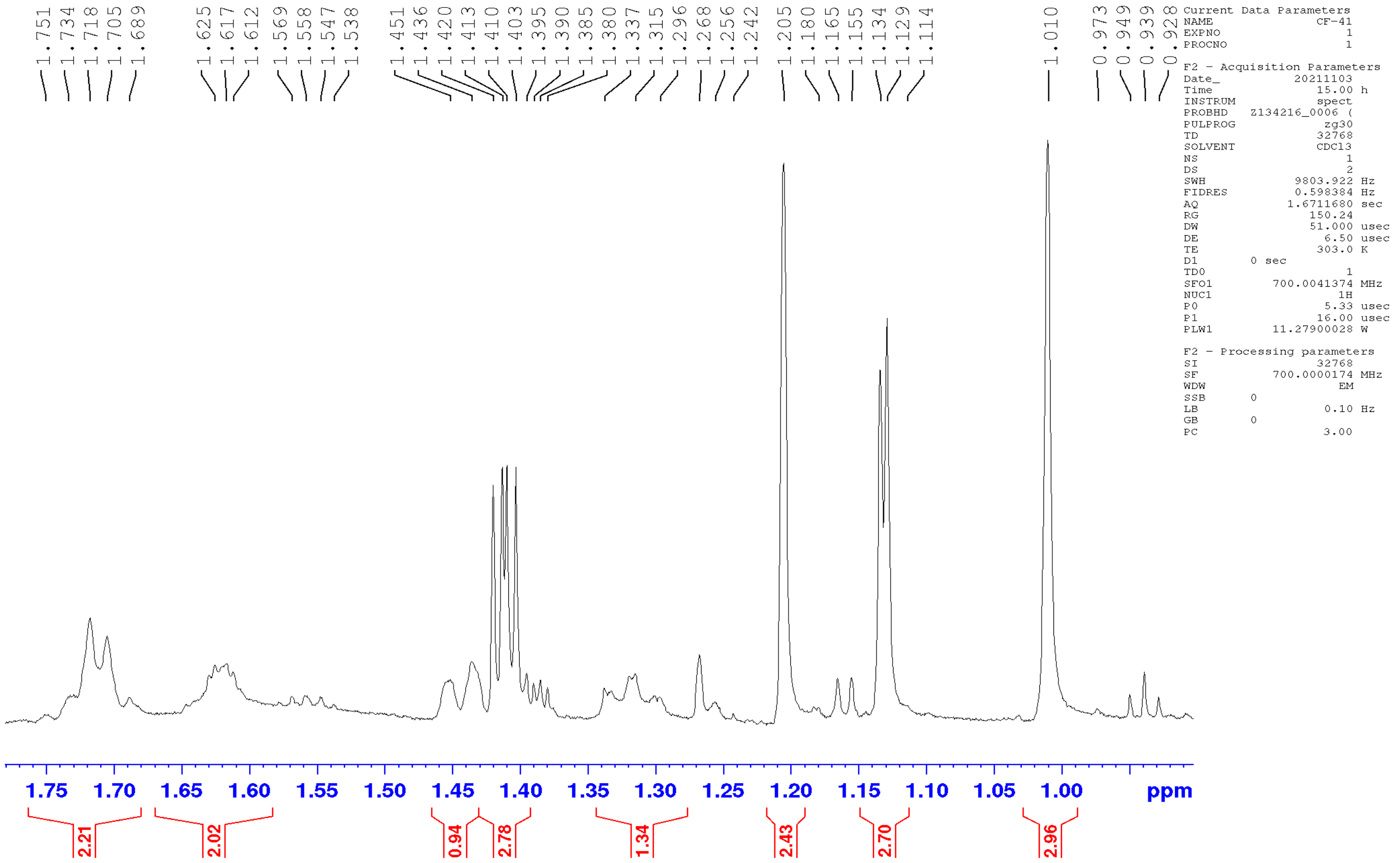

Figure S49. Expanded ^{1}H NMR spectrum (700 MHz, $CDCl_3$) of **10**

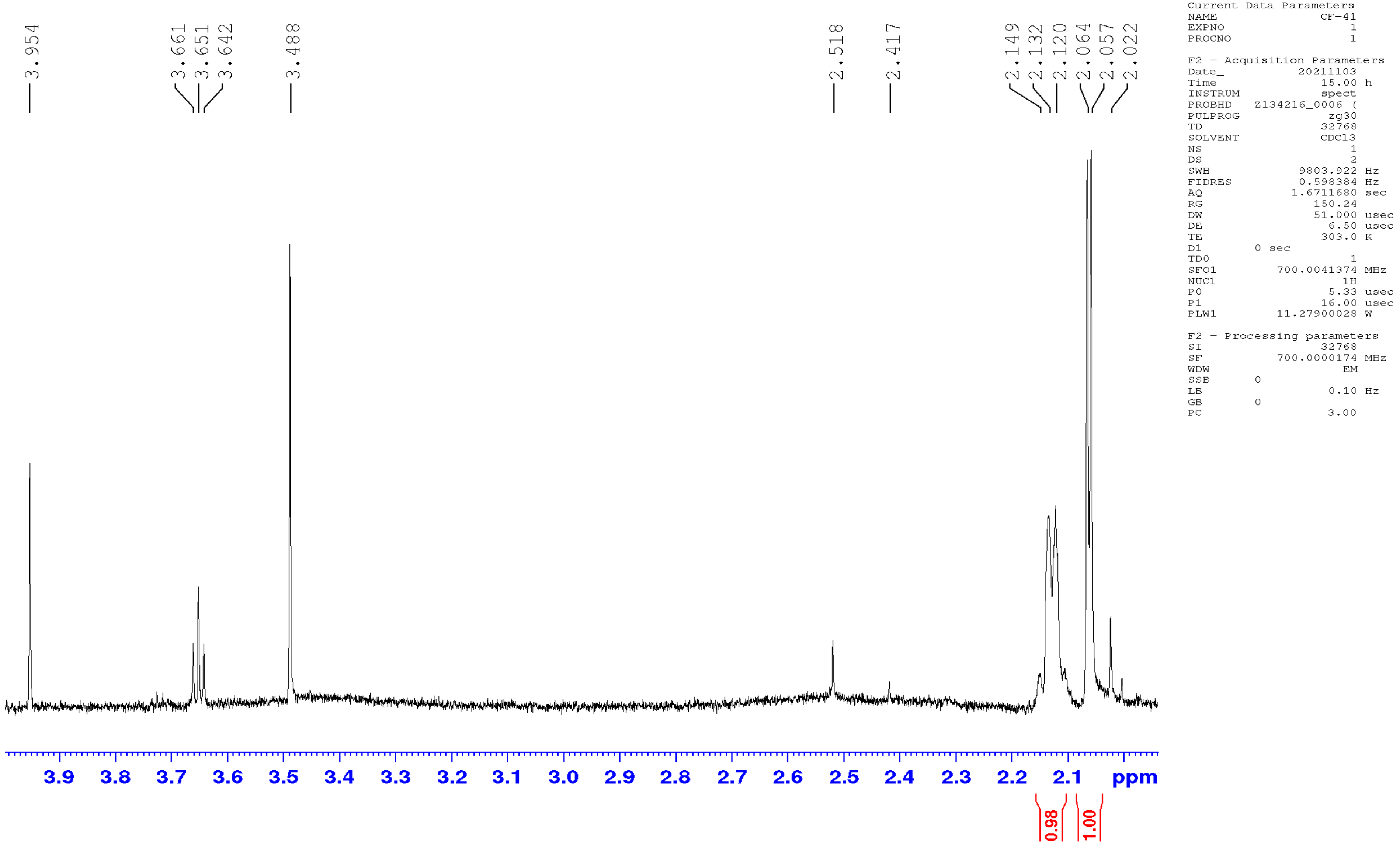

Expanded ^{1}H NMR spectrum (700 MHz, $CDCl_3$) of **10**

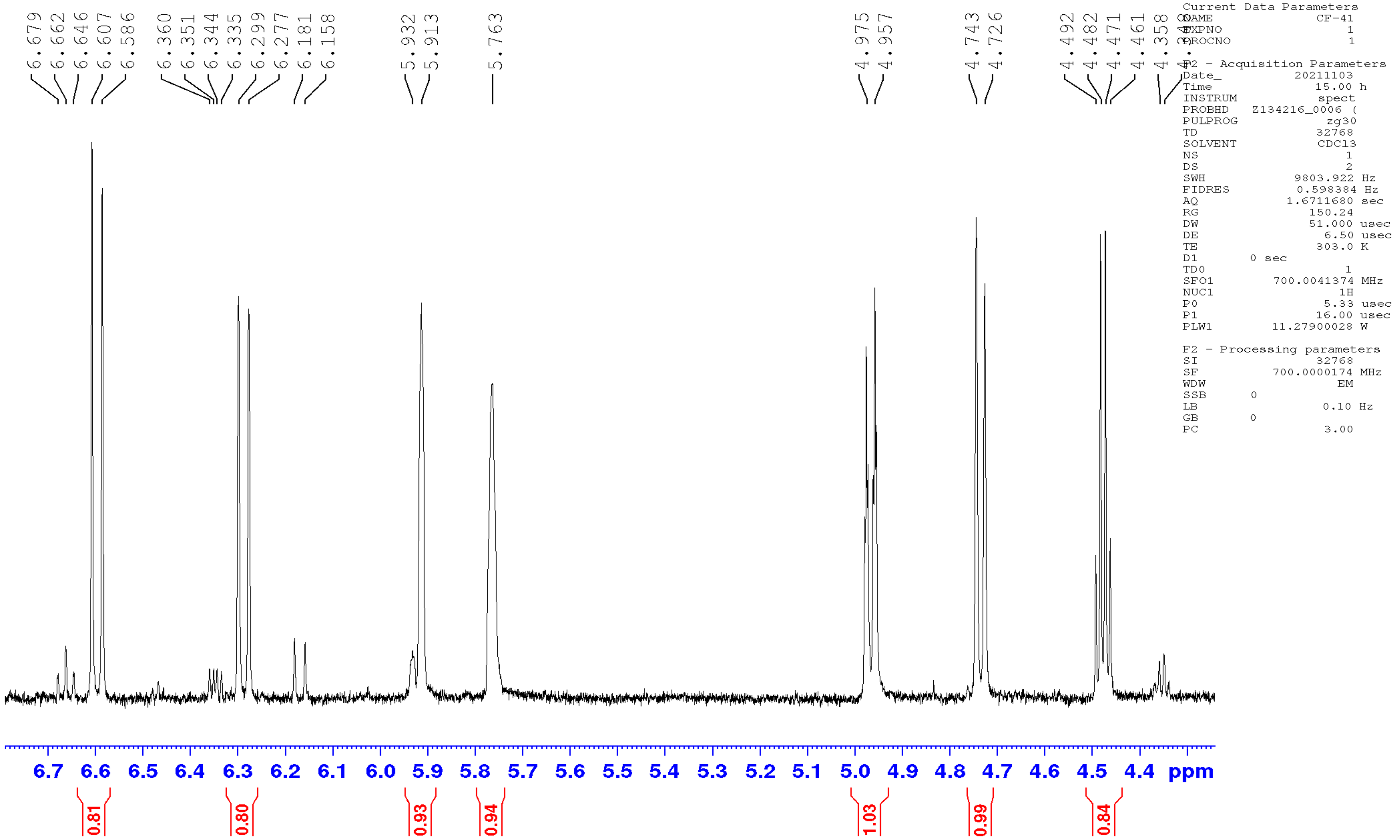

Expanded ^{1}H NMR spectrum (700 MHz, $CDCl_3$) of **10**

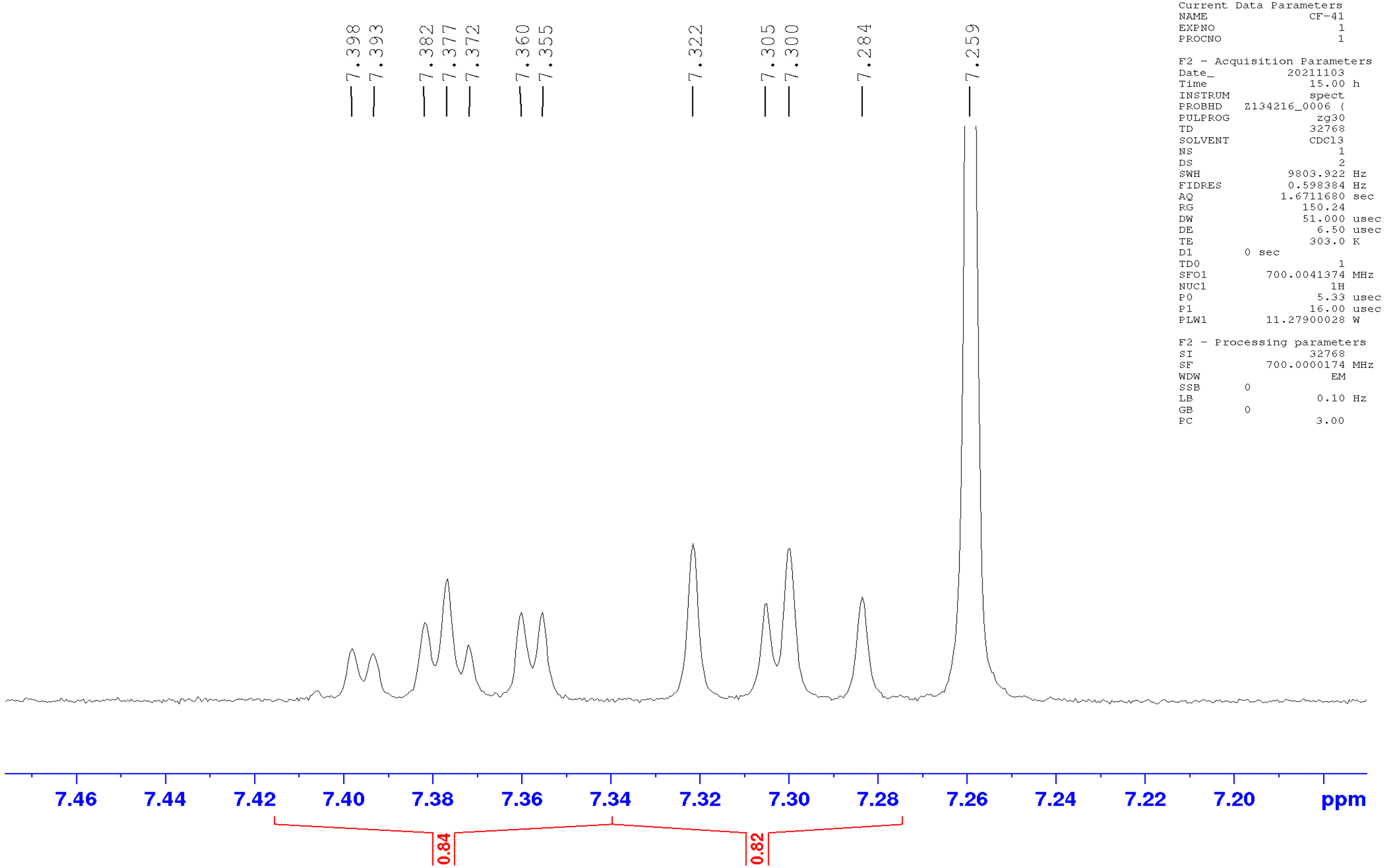

Expanded ^{1}H NMR spectrum (700 MHz, $CDCl_3$) of **10**

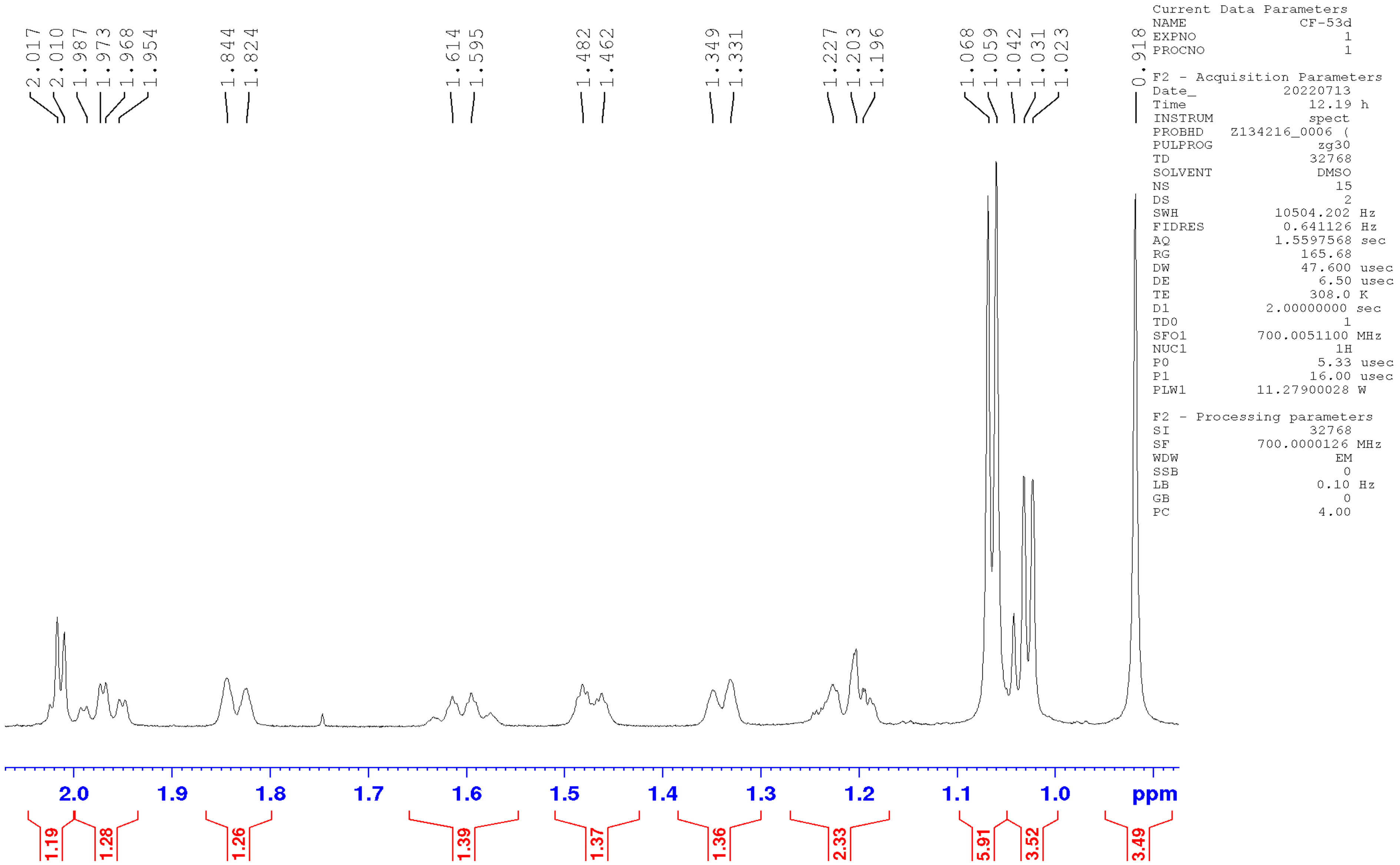

Figure S50. Expanded ^{1}H NMR spectrum (700 MHz, DMSO-d_6) of **11**

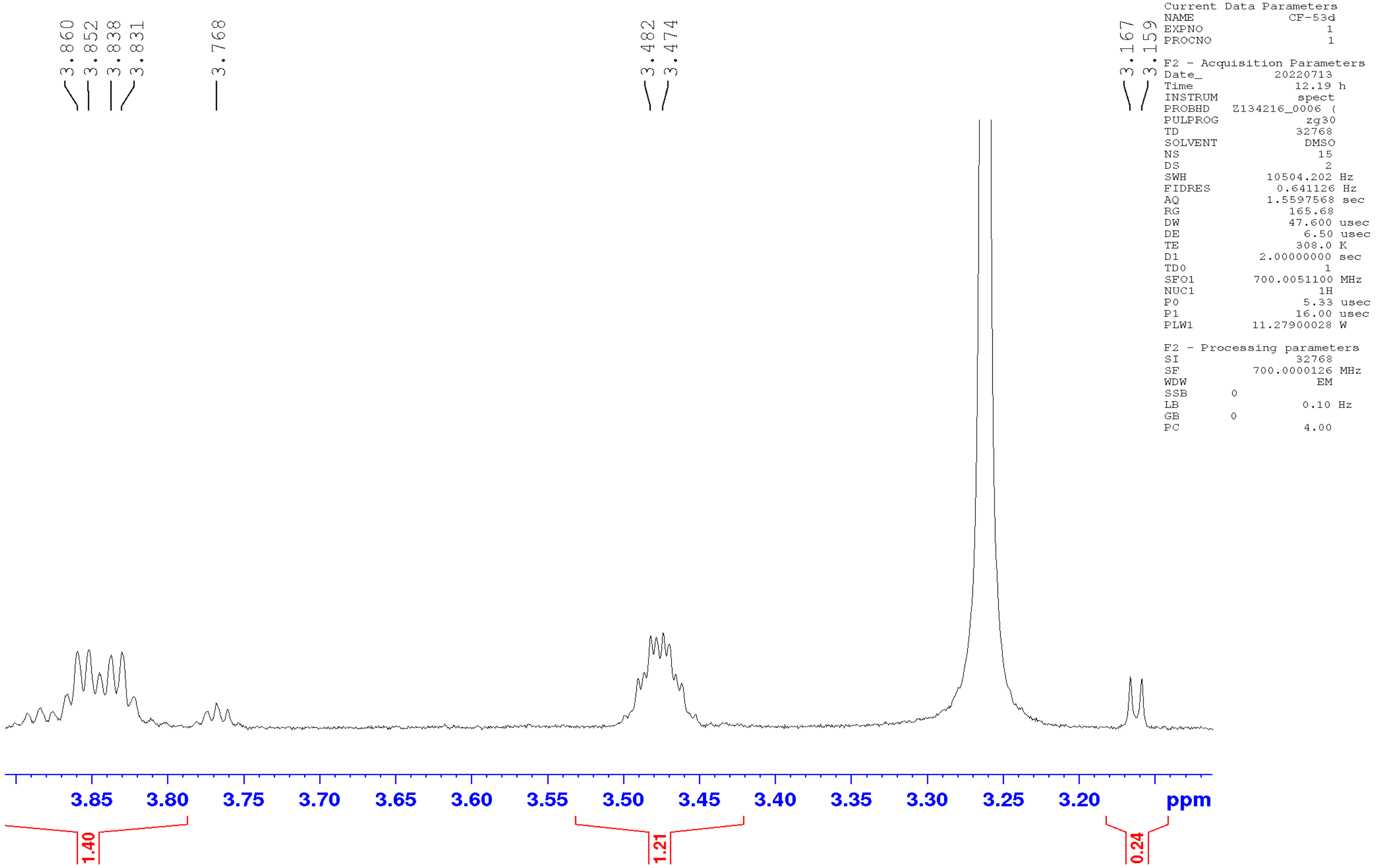

Expanded ^{1}H NMR spectrum (700 MHz, DMSO-d_6) of **11**

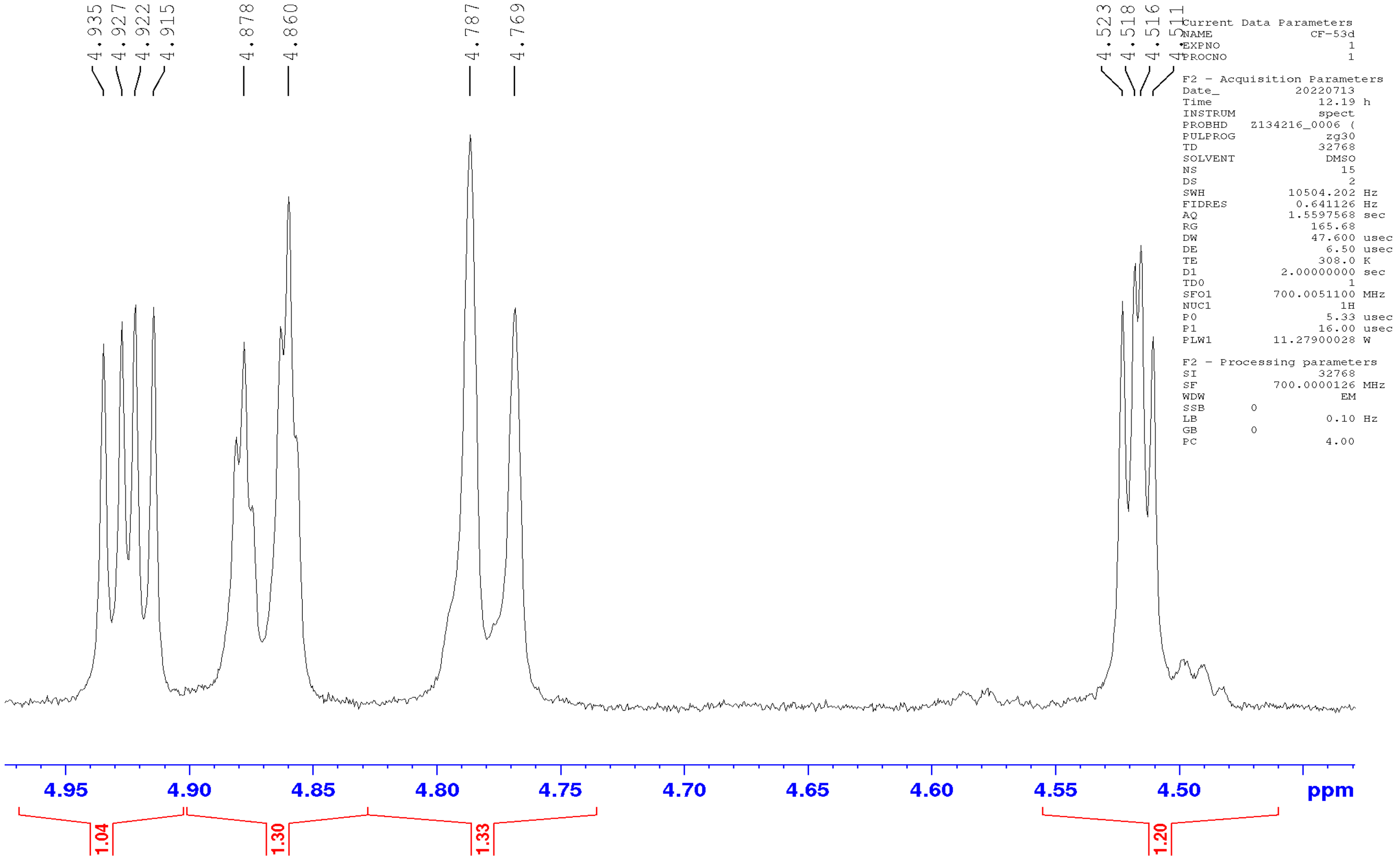

Expanded ^{1}H NMR spectrum (700 MHz, DMSO-d_6) of **11**

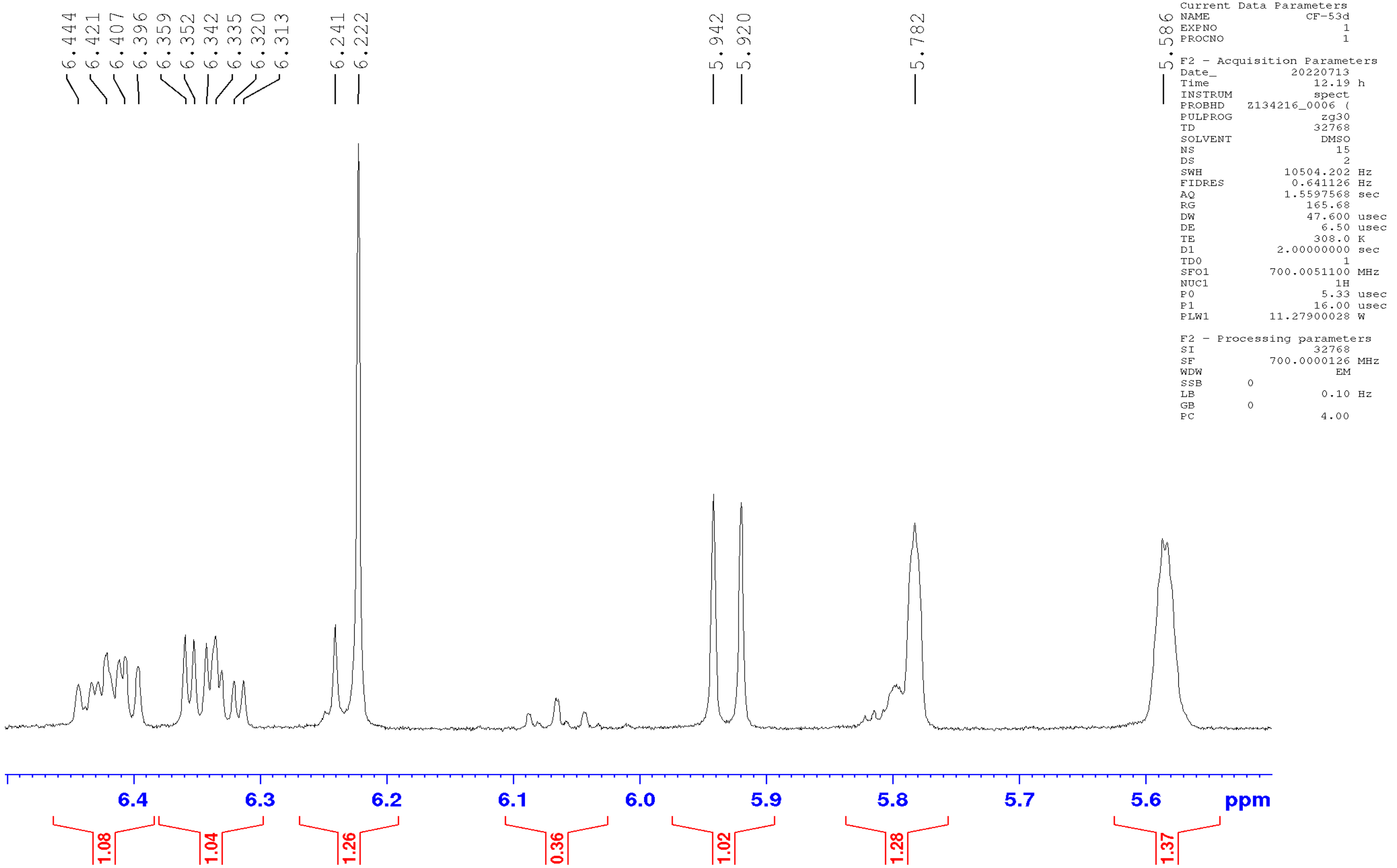

Expanded ^{1}H NMR spectrum (700 MHz, DMSO-d_6) of **11**

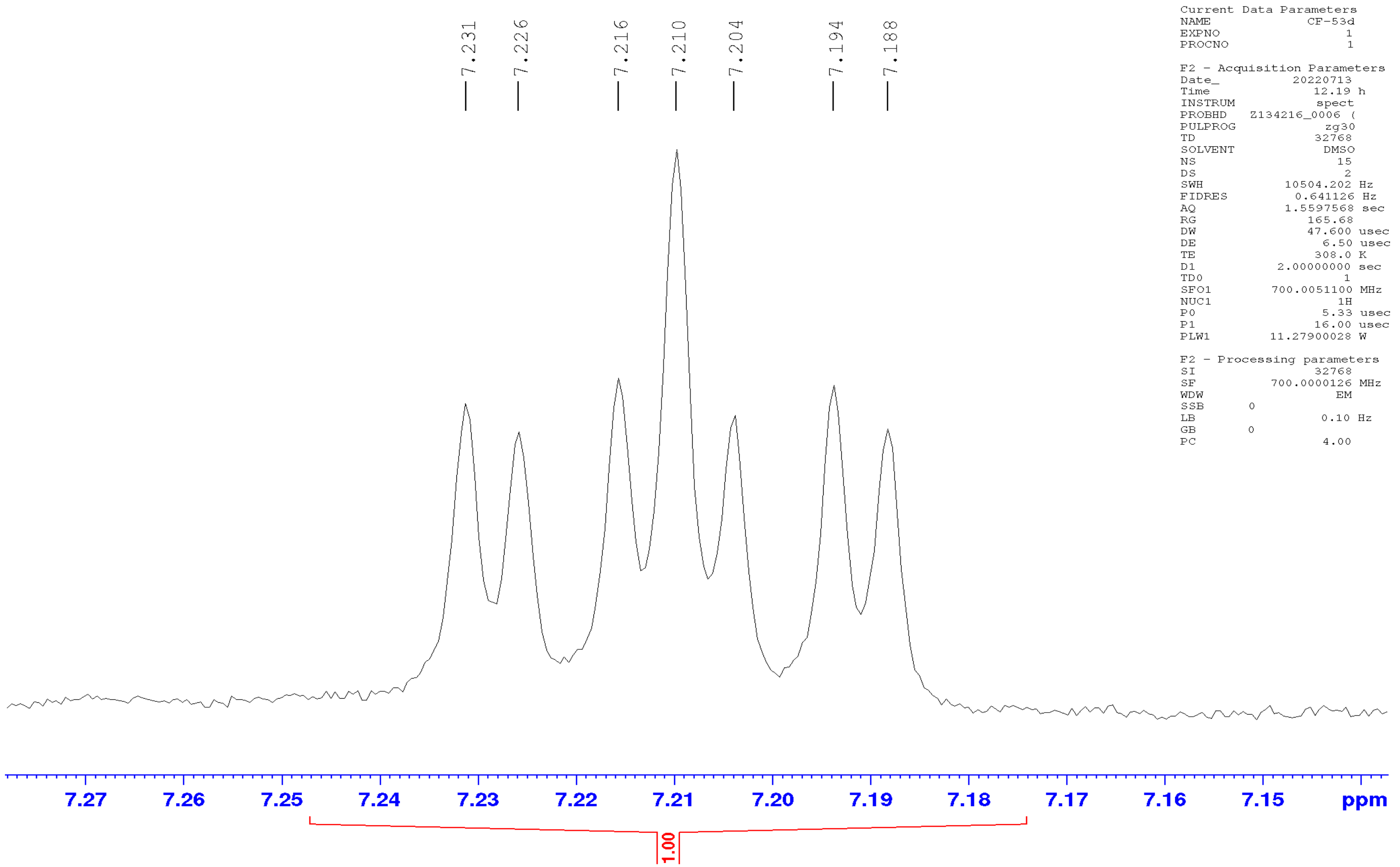

Expanded ^{1}H NMR spectrum (700 MHz, DMSO-d_6) of **11**

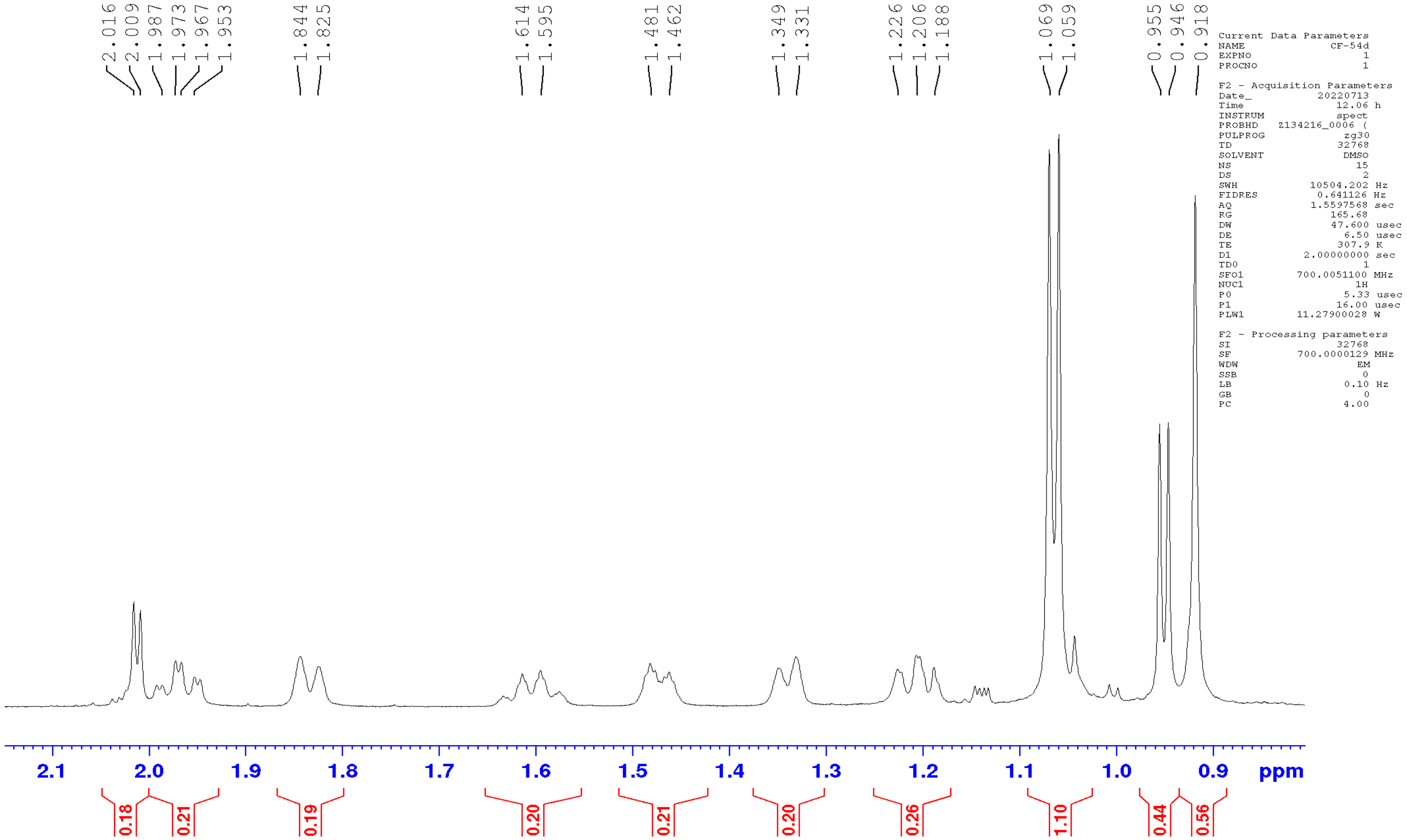

Figure S51. Expanded ^{1}H NMR spectrum (700 MHz, DMSO-d_6) of **12**

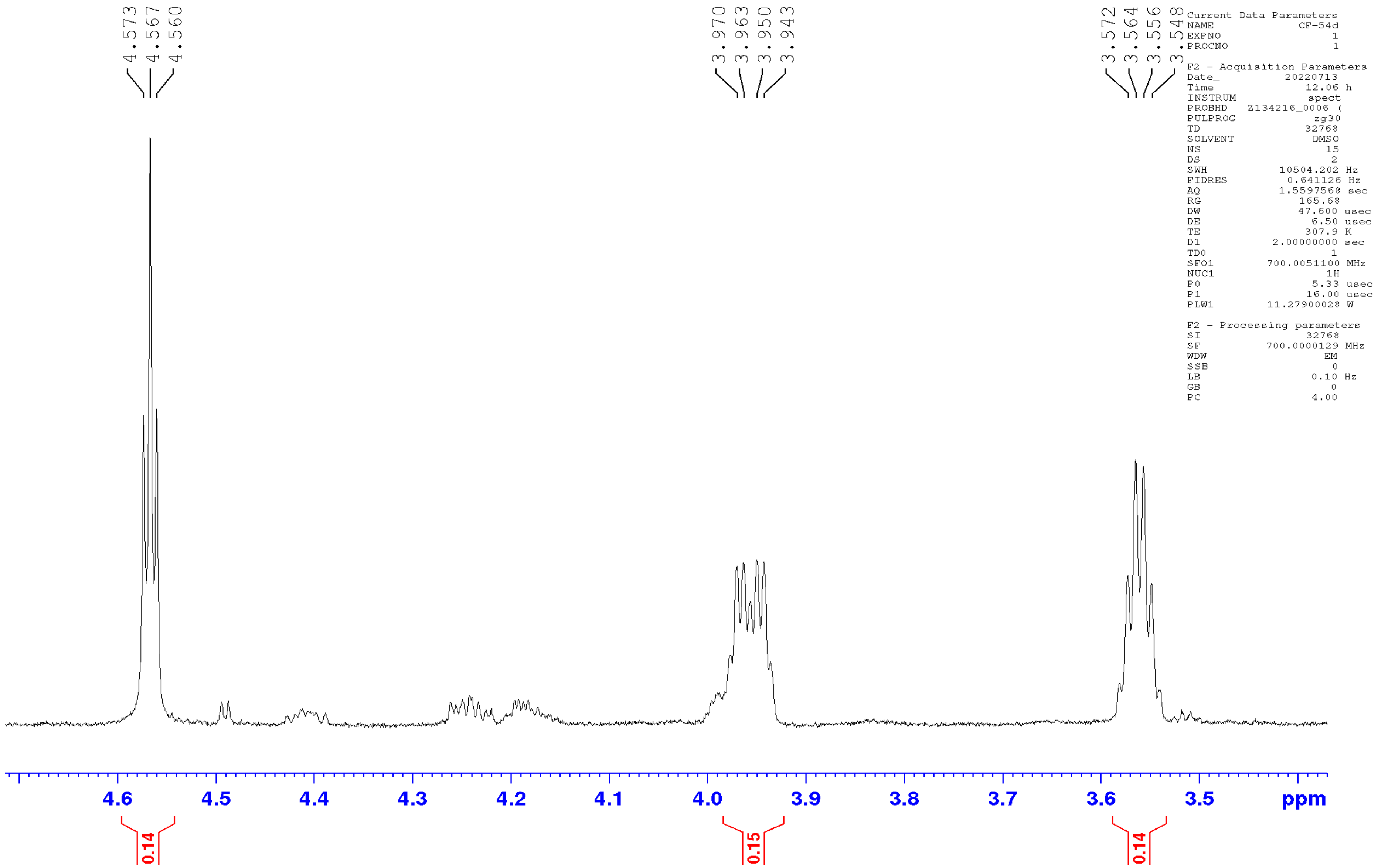

Expanded ^{1}H NMR spectrum (700 MHz, DMSO-d$_6$) of **12**

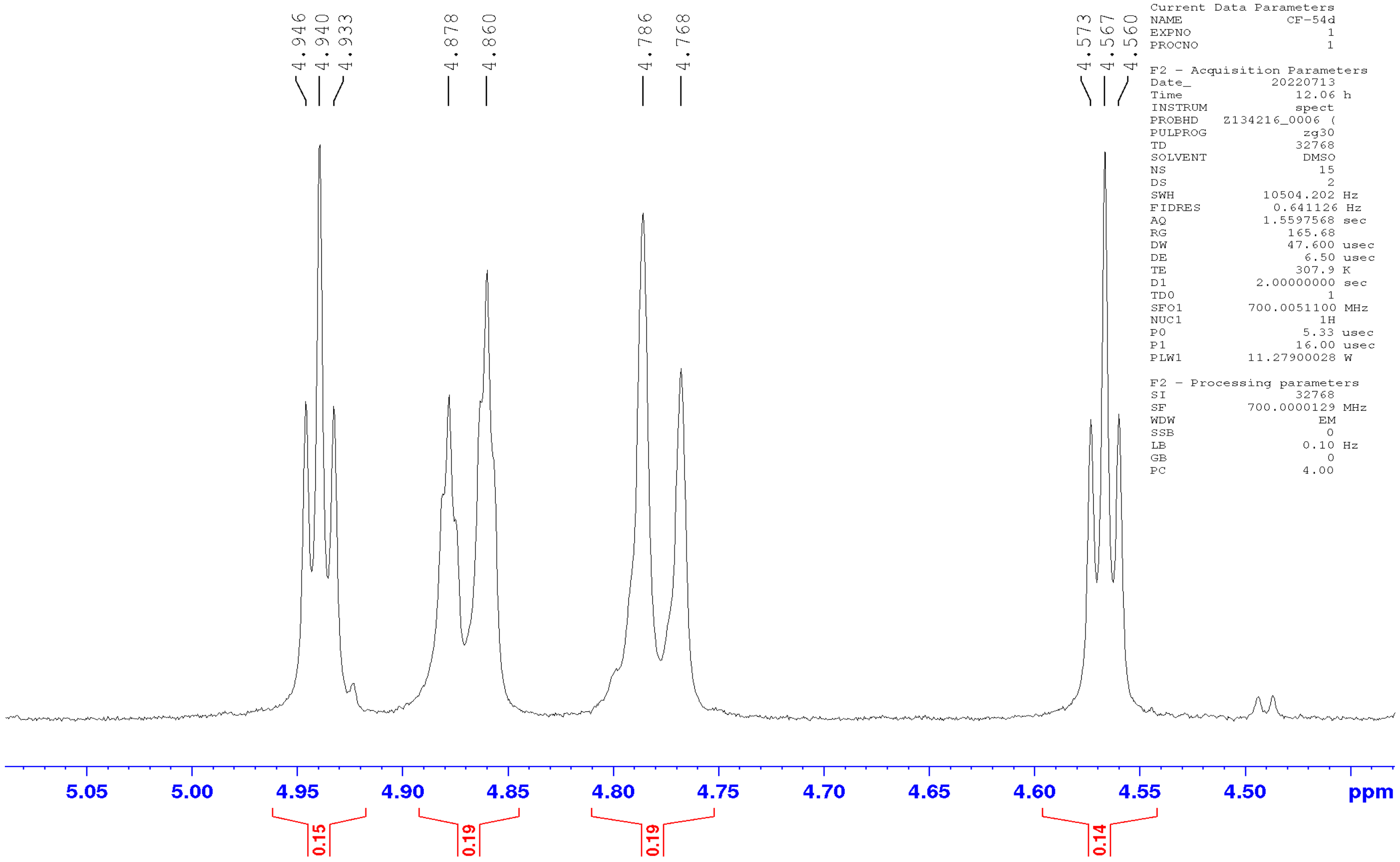

Expanded ^{1}H NMR spectrum (700 MHz, DMSO-d_6) of **12**

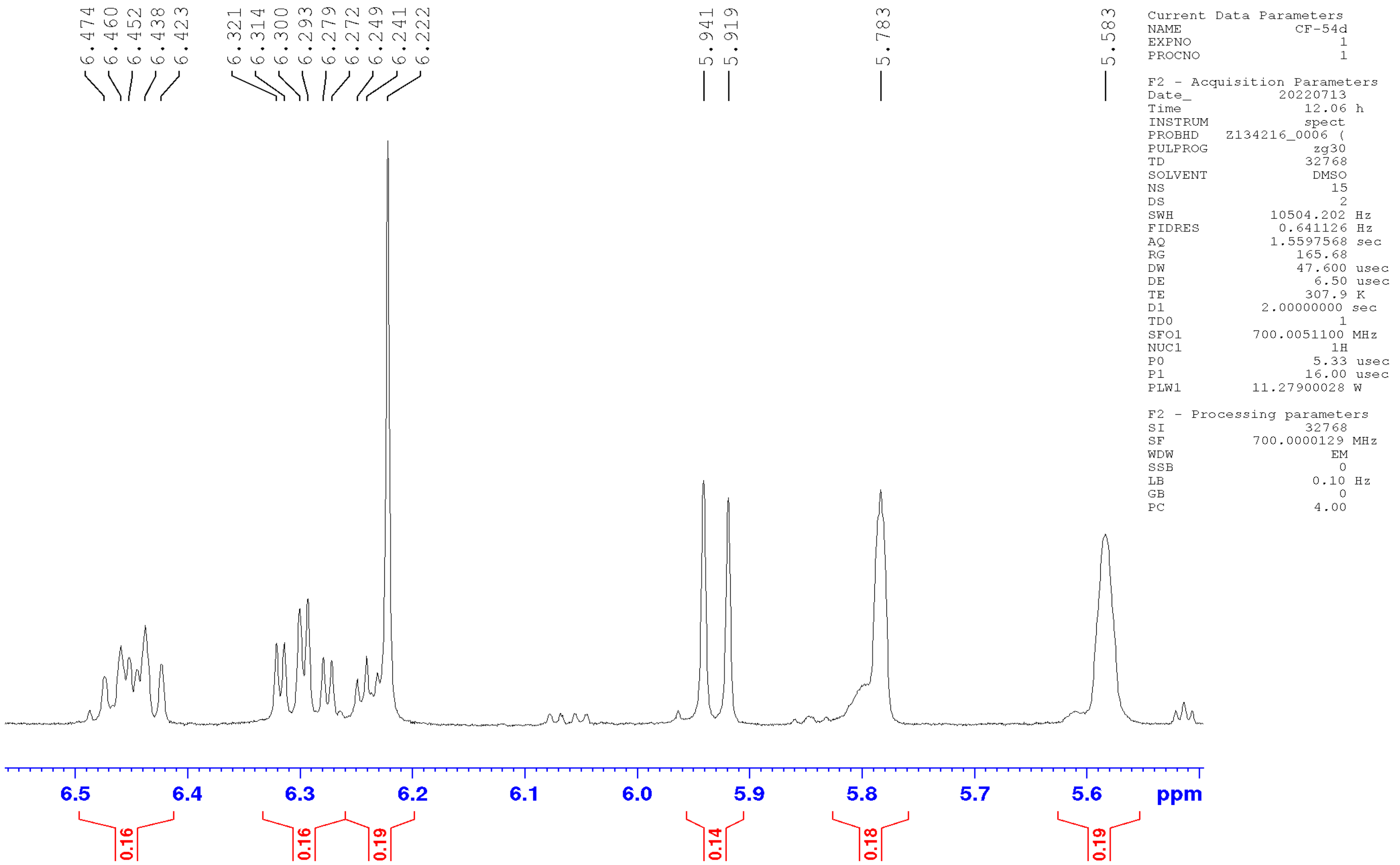

Expanded ^{1}H NMR spectrum (700 MHz, DMSO-d_6) of **12**

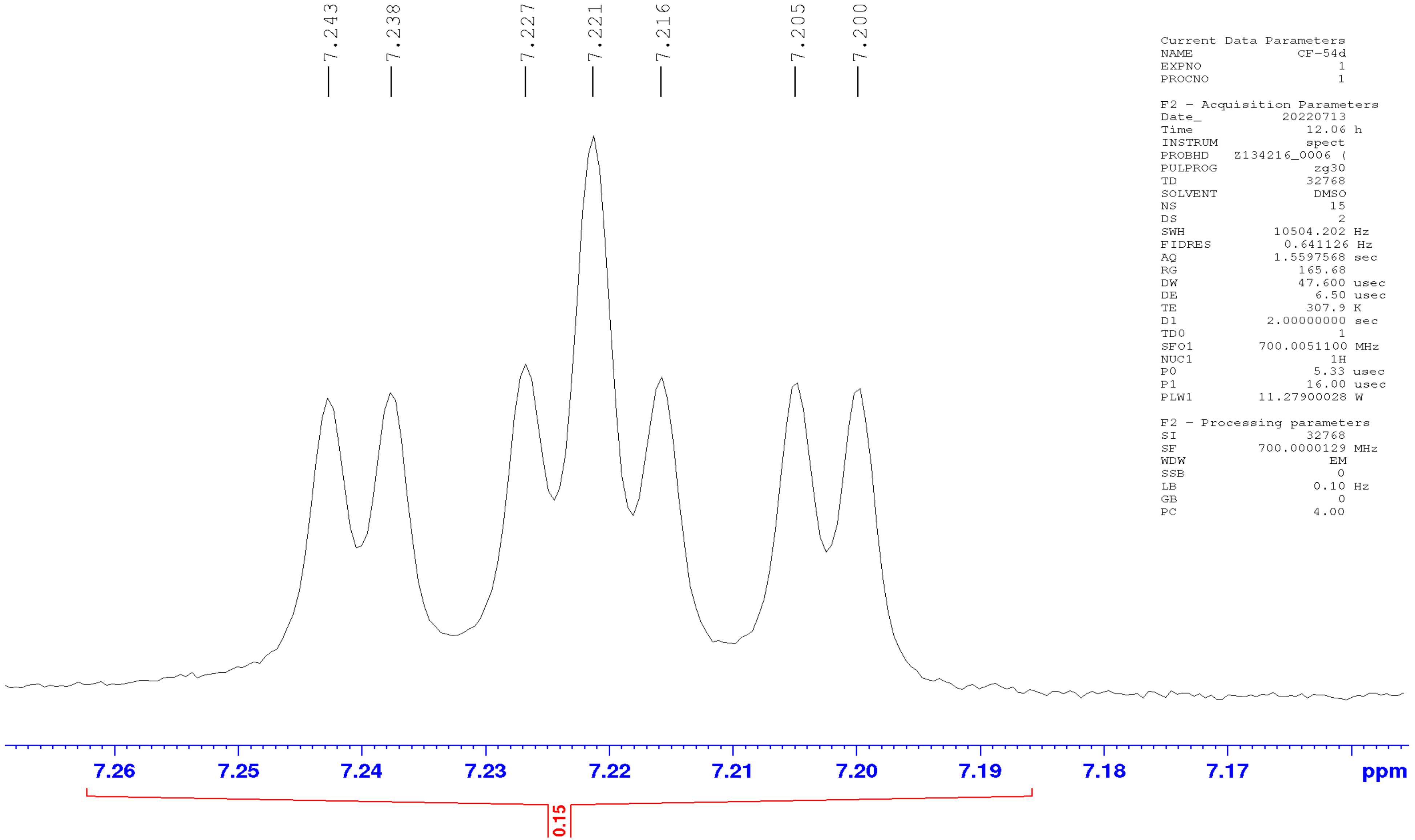

Expanded ^{1}H NMR spectrum (700 MHz, DMSO-d_6) of **12**

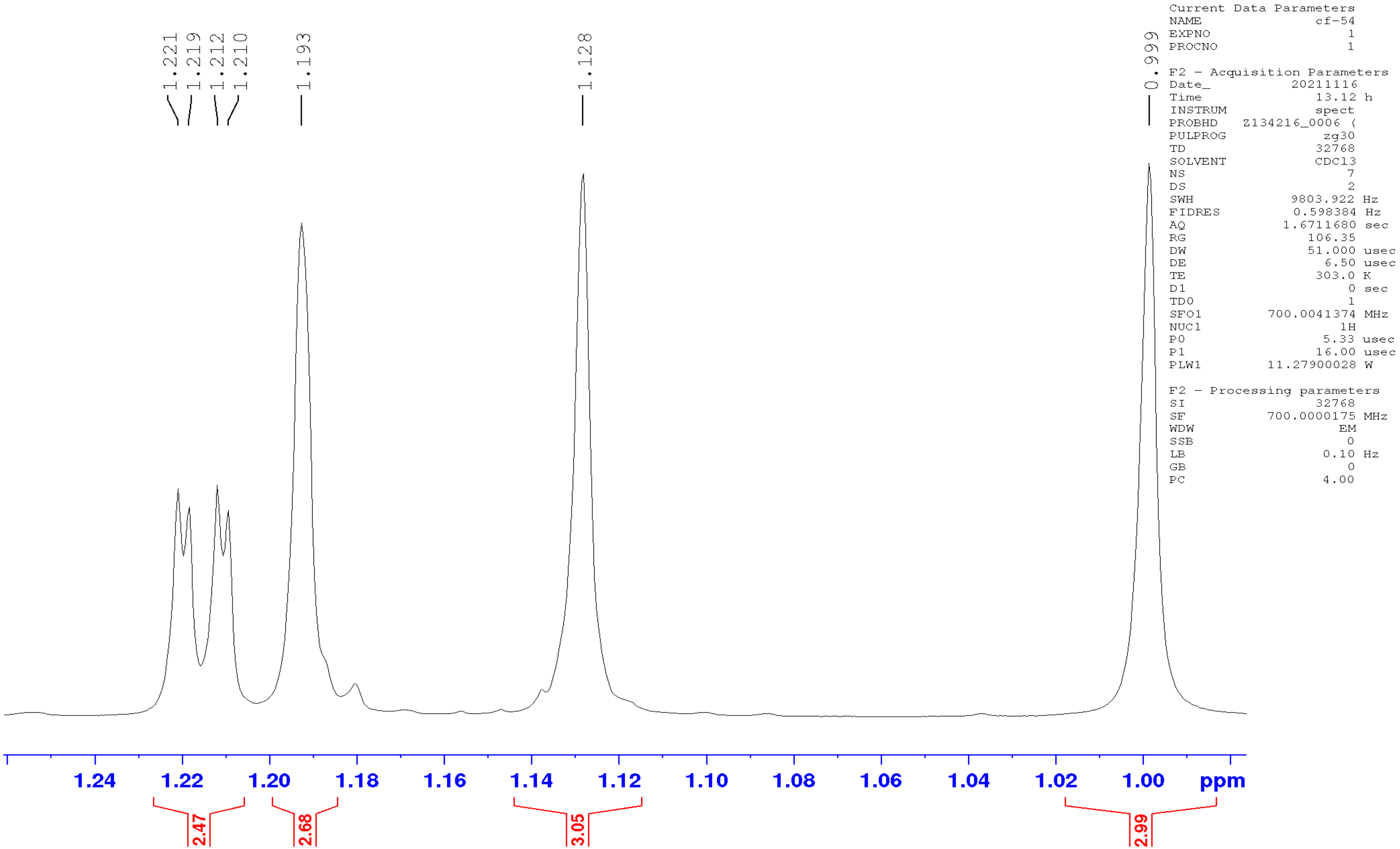

Figure S52. Expanded ^{1}H NMR spectrum (700 MHz, $CDCl_3$) of **12**

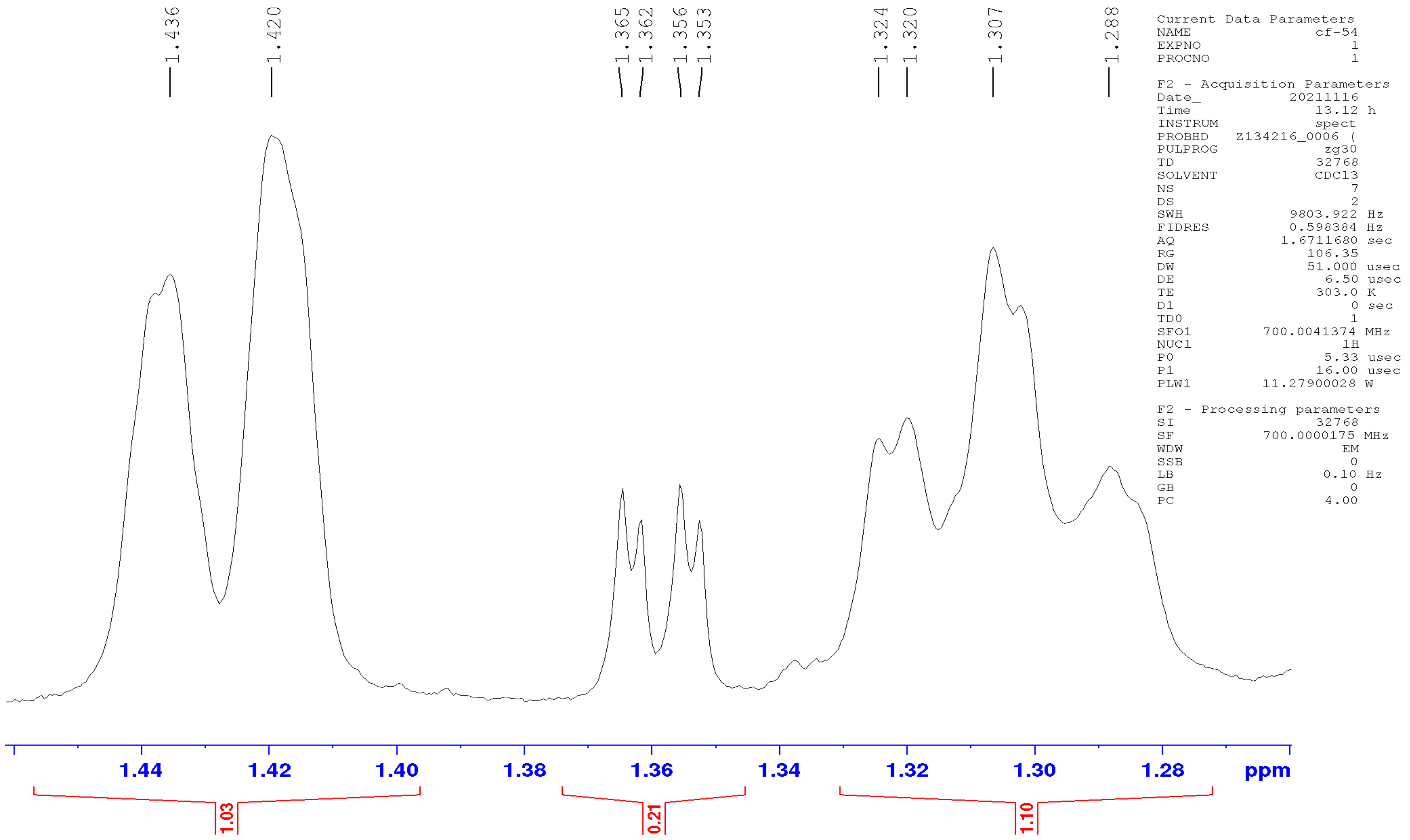

Expanded ^{1}H NMR spectrum (700 MHz, $CDCl_3$) of **12**

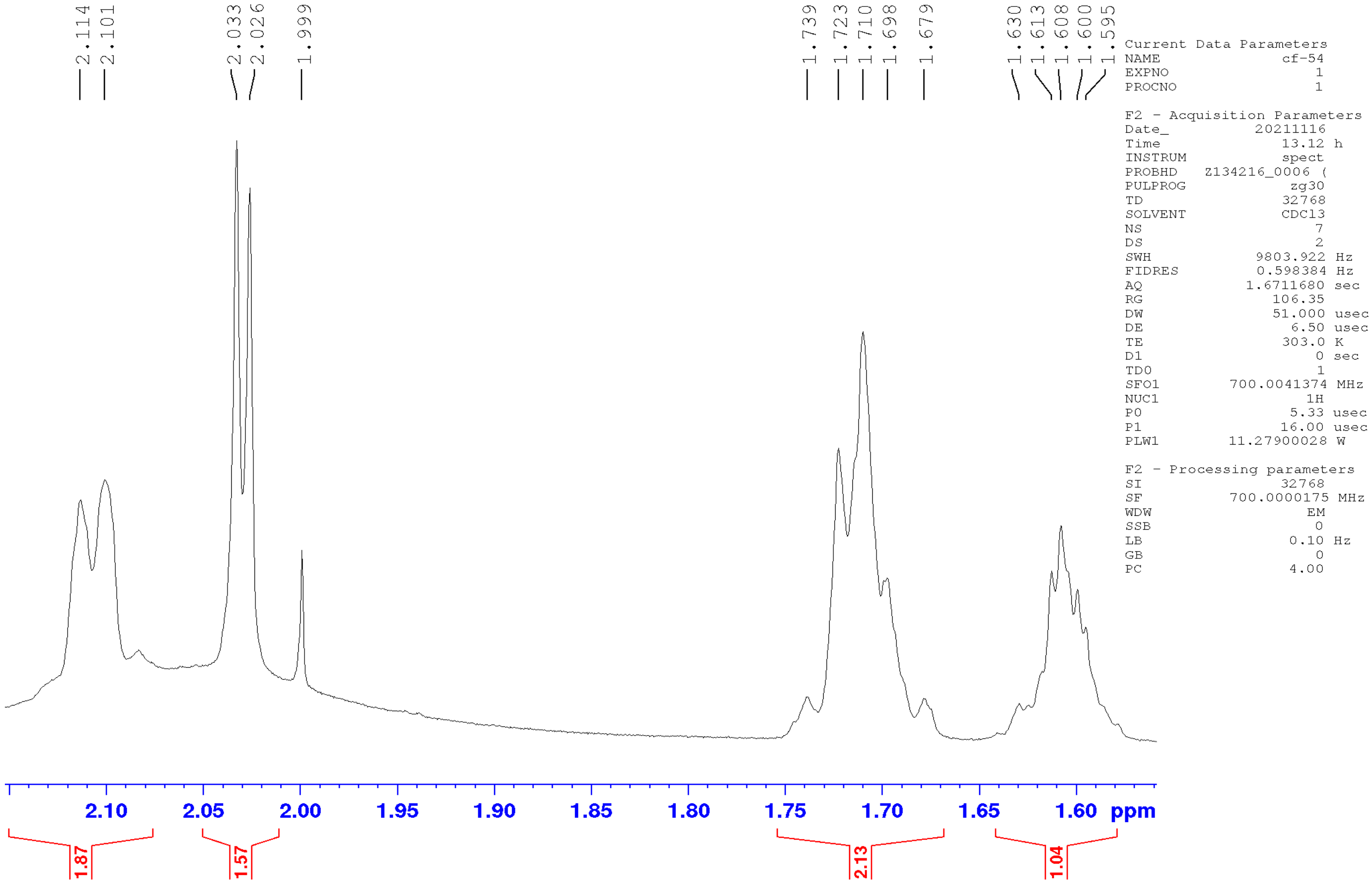

Expanded ^{1}H NMR spectrum (700 MHz, $CDCl_3$) of **12**

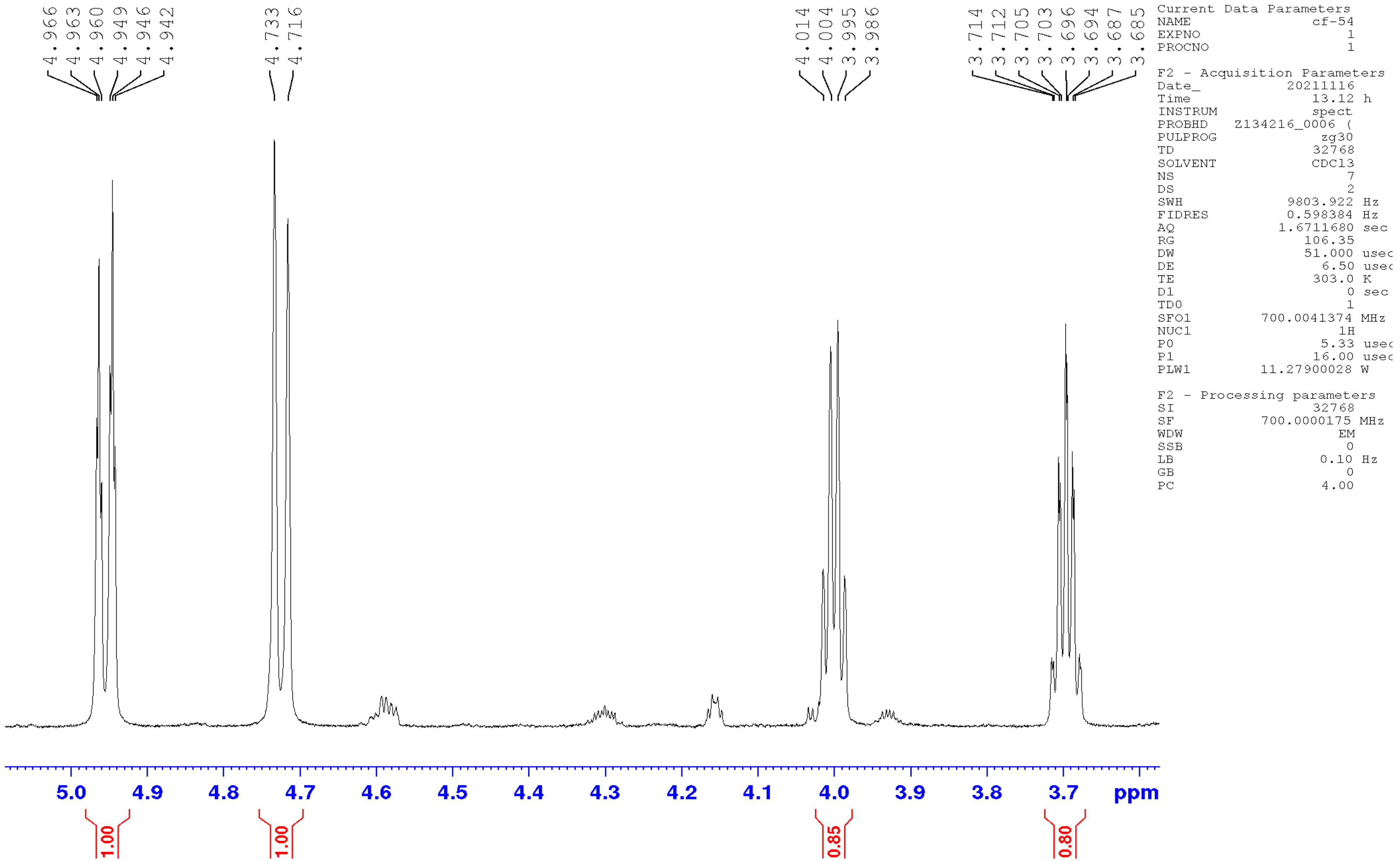

Expanded ^{1}H NMR spectrum (700 MHz, $CDCl_3$) of **12**

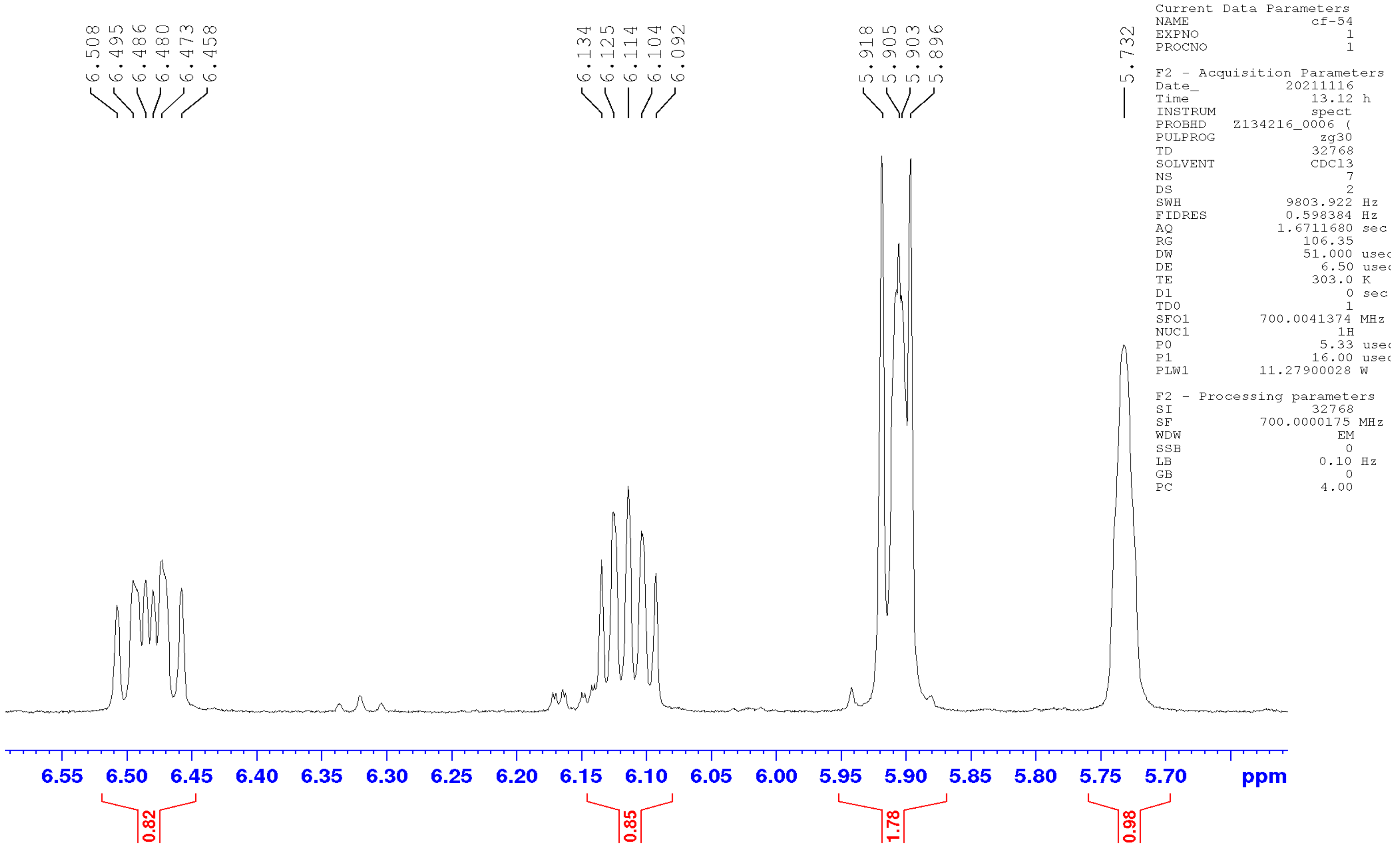

Expanded ^{1}H NMR spectrum (700 MHz, $CDCl_3$) of **12**

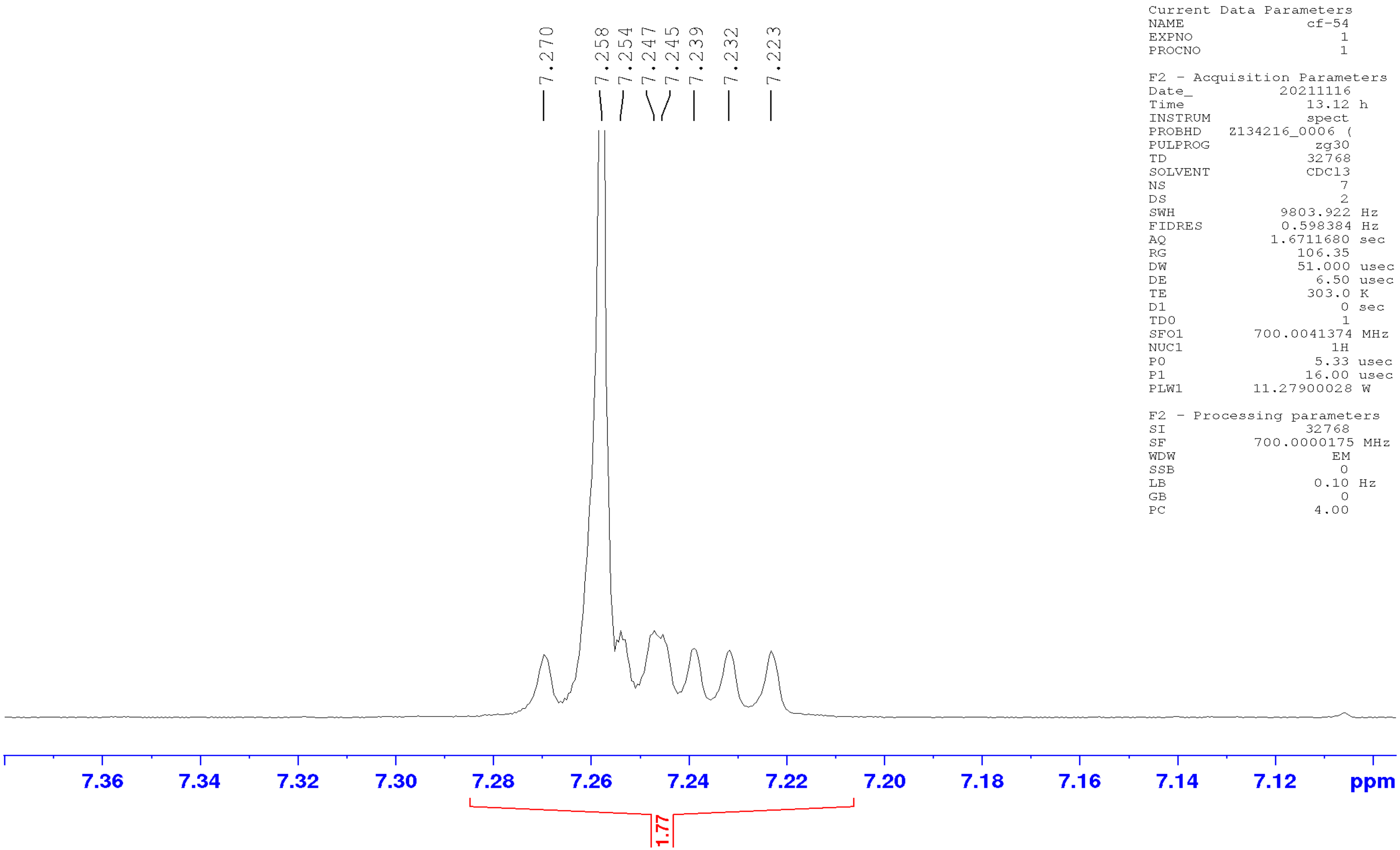

Expanded ^{1}H NMR spectrum (700 MHz, $CDCl_3$) of **12**

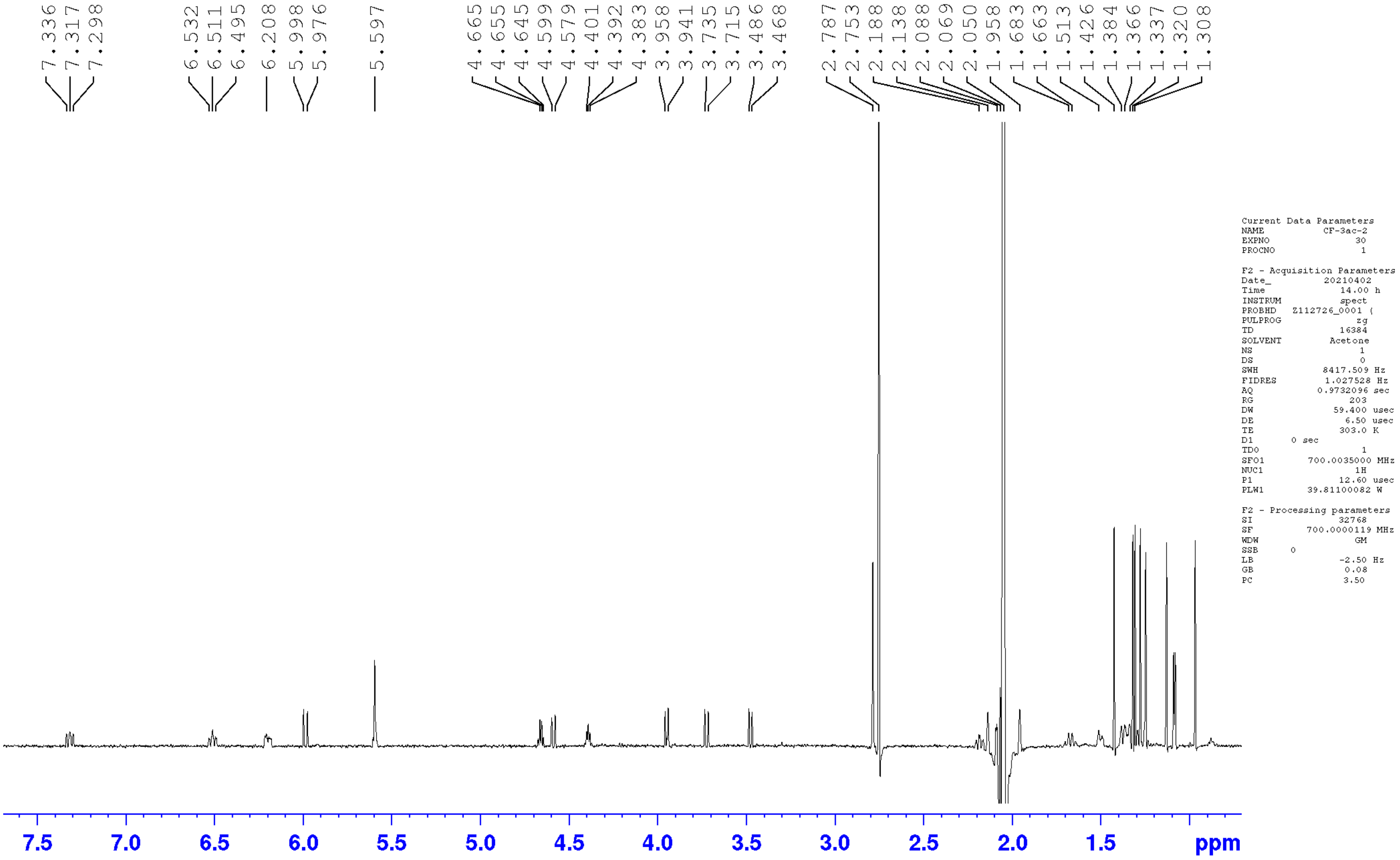

Figure S53. ^{1}H NMR spectrum (700 MHz, acetone-d_6) of **1a**

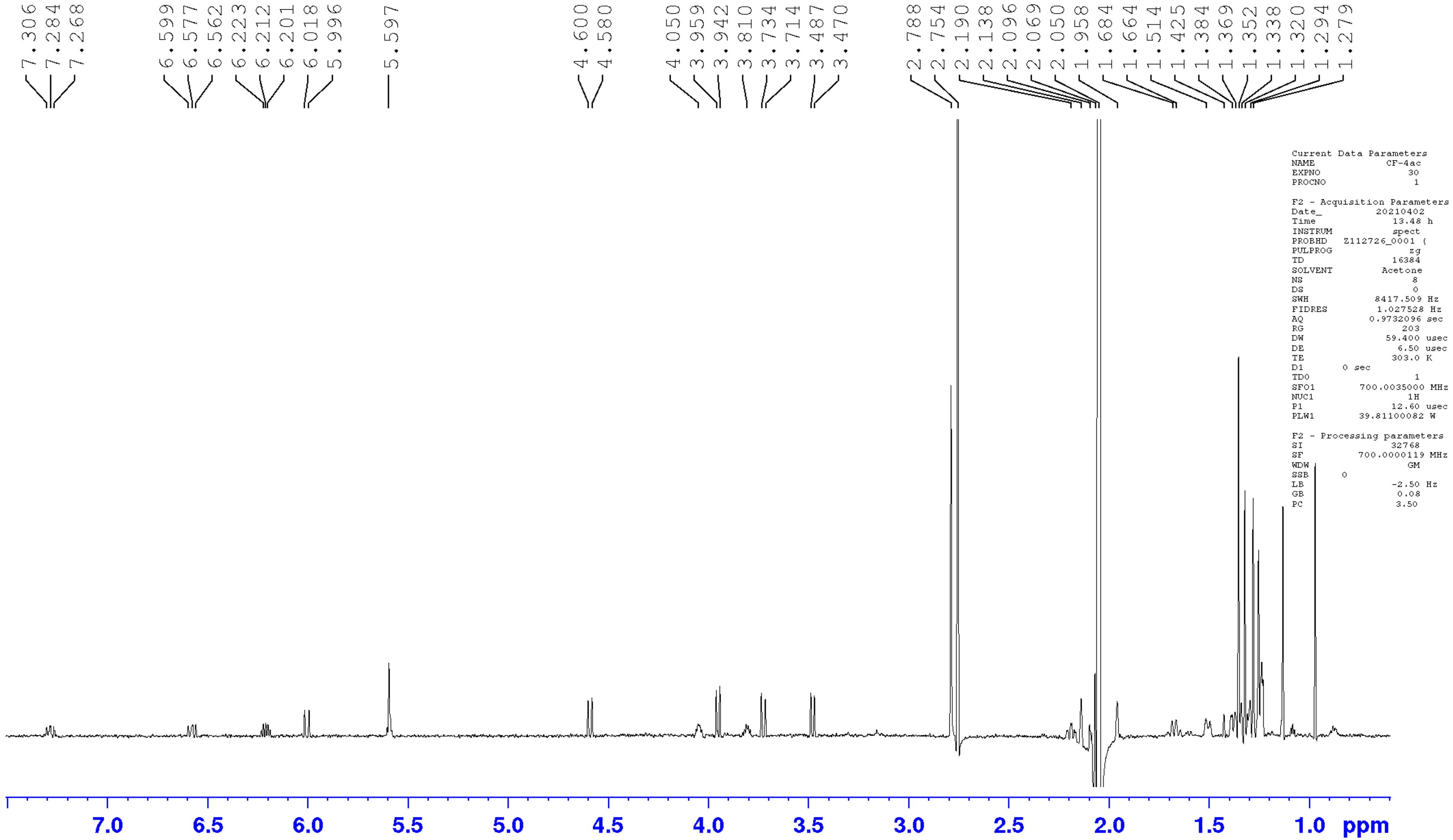

Figure S54. ^{1}H NMR spectrum (700 MHz, acetone-d_6) of **2a**

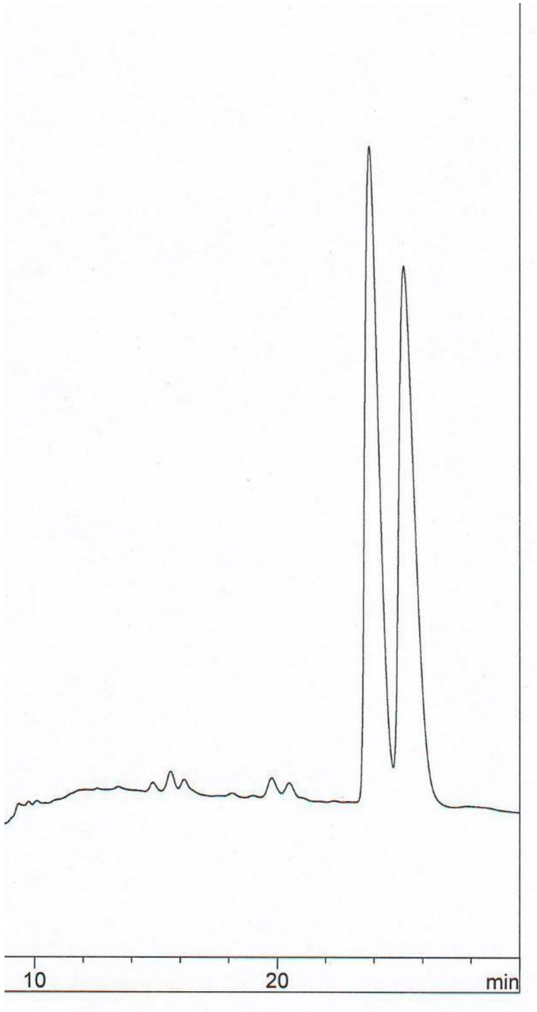

Figure S55. HPLC chart of compound **11** separation (Phenomenex CyperClone column, MeCN-water, 55:45, 0.7 ml/min).
